# CT-based AI system for quantitative and integrated management of acute respiratory distress syndrome in critical care

**DOI:** 10.1038/s41746-026-02648-9

**Published:** 2026-04-24

**Authors:** Yuetan Chu, Jianpeng Wang, Peiyao Luo, Hui Chen, Zhongheng Zhang, Jiannan Zhang, Yilan Zhang, Yingnan Ju, Yaxin Xiong, Xiqing Luo, Jiuyue Sun, Hongyu Shi, Mingbo Zhao, Tinghui Qiu, Yiqi Wang, Quankuan Gu, Ping Hang, Qiuyue Yang, Jiaman Guan, Yi Zhang, Renpei Lu, Ci Han, Yaoyu Gu, Changsong Wang, Kai Kang, Zhaowen Qiu, Xin Ge, Gongning Luo, Xin Gao, Kaijiang Yu, Mingyan Zhao, Xianglin Meng

**Affiliations:** 1 Department of Critical Care Medicine, the First Affiliated Hospital of Harbin Medical University, Harbin, Heilongjiang Province China; 2 Computer Science Program, Computer, Electrical and Mathematical Sciences and Engineering Division, King Abdullah University of Science and Technology (KAUST), Thuwal, Kingdom of Saudi Arabia; 3 Center of Excellence for Smart Health (KCSH), King Abdullah University of Science and Technology (KAUST), Thuwal, Kingdom of Saudi Arabia; 4 Department of Critical Care Medicine, Zhongda Hospital, School of Medicine, Southeast University, No. 87, Dingjiaqiao Road, Gulou District, Nanjing, China; 5 Department of Emergency Medicine, Sir Run Run Shaw Hospital, Zhejiang University School of Medicine, Hangzhou, Zhejiang Province China; 6 Department of Intensive Care Unit, Hainan General Hospital, Hainan Affiliated Hospital of Hainan Medical University, Haikou, China; 7 Northeast Forestry University, Harbin, China; 8 Department of Emergency and Critical Care Medicine, Wuxi Ninth People’s Hospital Affiliated to Soochow University, Wuxi, China; 9 Cancer Institute and Department of Nuclear Medicine, Fudan University Shanghai Cancer Center, Shanghai, China; 10 Key Laboratory of Hepatosplenic Surgery, Ministry of Education, The First Affiliated Hospital of Harbin Medical University, Harbin, China; 11 Heilongjiang Tuomeng Technology Co., Ltd., Harbin, China

**Keywords:** Biomarkers, Computational biology and bioinformatics, Diseases, Health care, Medical research

## Abstract

Acute respiratory distress syndrome (ARDS) remains a major challenge in critical care, with mortality exceeding 40%. Its diagnosis and management depend on multi-step procedures, invasive arterial blood gas analysis, and subjective CT interpretation, often leading to inconsistency, delayed intervention, and increased procedural burden. To address these limitations, we develop AutoARDS, an all-in-one foundation model that transforms routine chest CT into a quantitative platform, enabling integrated and reproducible assessment of diagnosis, progression, oxygenation, physiology, and prognosis within a single, non-invasive workflow, thereby supporting faster and more standardized critical-care decisions. Technically, AutoARDS proposes to employ a multi-task pretraining strategy with adversarial perturbation, distilling routine but unstructured clinical data into unified representations for fine-grained pathological learning. Trained on over 50,000 CT volumes and validated across six medical centers (6,153 individuals), AutoARDS (1) established a reproducible CT-derived biomarker linking morphological injury with disease severity, enabling standardized tracking of pulmonary progression; (2) accurately diagnosed acute respiratory failure and ARDS (AUCs = 0.97 and 0.87), facilitating early recognition and reducing diagnostic delay; (3) directly estimated the P/F ratio (PCC = 0.83), outperforming SpO_2_-based monitoring for noninvasive severity stratification and ventilation management; and (4) predicted 28-day outcomes (time-averaged AUC = 0.79), providing complementary risk assessment for clinical planning. Further analyses confirm generalizability to ARDS-associated right ventricular dysfunction (AUC = 0.76) and revealed a positive shift image-derived age residuals, reflecting disease-related imaging patterns that resemble pulmonary aging. By bridging visual information with quantitative physiology, AutoARDS exemplifies a scalable blueprint for transforming chest CT into an integrated, quantitative platform for precise and reproducible critical-care management.

## Introduction

Acute respiratory distress syndrome (ARDS) is a severe form of acute lung injury characterized by the rapid onset of hypoxemia, diffuse alveolar damage, and bilateral pulmonary infiltrates^1^. Despite advances in supportive care, mortality in intensive care units (ICUs) remains above 40%^2^. Effective diagnosis and management rely on synthesizing information from invasive and multi-step procedures, including arterial blood gas (ABG) analysis, ventilatory parameters, hemodynamic monitoring, and imaging interpretation^3,4^. This complexity delays timely decision-making, increases procedural burden, and hampers consistency across institutions. Moreover, while ABG provides the gold-standard measurement of oxygenation, its invasive and intermittent nature increases patient burden and limits accessibility and temporal resolution, particularly in unstable or resource-limited settings^5^. In parallel, radiological evaluation of ARDS, including chest X-ray and CT, is routinely employed as a non-invasive imaging modality. However, it remains largely qualitative and subjective, relying on visual recognition that varies across readers and institutions and lacks standardized quantitative benchmarks^6,7^. Together, these barriers highlight the need for a quantitative and reproducible paradigm that provides unified references and could support more timely, objective, and consistent ARDS management.

Recent advances in artificial intelligence (AI) have revealed that medical imaging can encode not only abnormal morphology but also rich pathological and physiological information beyond traditional, subjective interpretation^8–11^. Such information holds considerable promise for supporting a broad spectrum of ARDS-related tasks, including early diagnosis, physiological estimation, longitudinal monitoring, and treatment planning. However, most current AI applications remain narrowly focused on isolated objectives such as classification or segmentation^12–15^. This limited scope underutilizes the quantitative capacity of CT and constrains its role within integrated, data-driven ARDS management.

In this study, we present AutoARDS, a comprehensive framework that systematically transforms chest CT imaging into a quantitatively integrated platform for assisting the evaluation and management of ARDS within existing clinical workflows. By decoding pathological information from CT scans into standardized and reproducible metrics, AutoARDS extends routine imaging beyond traditional qualitative and subjective interpretation. Instead, it redefines the role of chest CT, enabling precise predictions across the spectrum of ARDS diagnosis and management, including diagnosis, oxygenation assessment, severity stratification, prognostic evaluation, and complication risk estimation, as well as providing complementary quantitative references to support longitudinal monitoring and clinical decision-making throughout intensive care. At imaging time points, AutoARDS can further provide a non-invasive estimate of oxygenation as a complementary quantitative reference, particularly when chest CT is already being performed, and ABG measurements are delayed, temporally unavailable, or not readily aligned with imaging. This may facilitate more consistent, objective, and data-driven interpretation of ARDS within the ICU.

To achieve these objectives, AutoARDS first enables lesion quantification through a self-supervised synthesis-contrast strategy that reconstructs healthy lung counterparts and identifies voxel-wise abnormalities, achieving a Dice score of 75.6% and outperforming the evaluated fully supervised baselines. This quantitative mapping reveals a reproducible CT-derived biomarker that links pulmonary morphological injury to oxygenation impairment, enabling consistent tracking of disease progress. Building on this, AutoARDS employs a multi-task vision-language pretraining framework that distills routinely collected but unstructured clinical information, including CT imaging, radiology reports, patient metadata, and physiological measurements, into unified, clinically interpretable representations. An adversarial perturbation strategy further enhances the sensitivity to fine-grained imaging features that correspond to physiological decline and patient outcomes, effectively aligning image semantics with clinical reasoning.

Clinically, AutoARDS first enables accurate identification for ARDS recognition within acute respiratory failure (AUCs of 0.85–0.92), thus supporting timely diagnosis and mechanical ventilation management. AutoARDS also supports non-invasive assessment of oxygenation when ABG sampling is delayed or infeasible, achieving direct CT-based estimation of the PaO_2_/FiO_2_ ratio (PCC = 0.83), supporting fast Berlin-defined severity stratification with 72–75% accuracy. These results demonstrate that quantitative imaging can provide continuous, standardized references for functional evaluation and decision-making in intensive care. AutoARDS also offers prognostic insights (time-averaged AUC = 0.79 for 28-day survival) and can be extended beyond ARDS to detect associated right ventricular dysfunction (AUC = 0.67–0.86), demonstrating its generalizability in pathological information representation. In addition, imaging-derived age analysis reveals a general positive shift in ARDS patients, reflecting disease-associated imaging phenotypes. By quantitatively decoding the physiological information embedded in CT data, AutoARDS bridges imaging morphology with real-world clinical decision-making, supporting earlier recognition, more consistent monitoring, and data-driven management of ARDS in critical care^16^.

## Results

### Overview of AutoARDS development and datasets

The development of AutoARDS follows three key stages: lesion quantification, task-unified multimodal pretraining, and task-specific fine-tuning. First, a synthesis-contrast strategy enables self-supervised lesion segmentation and quantitative characterization of ARDS, providing objective descriptors of disease burden. Second, multimodal pretraining aligns CT scans with radiological reports and incorporates auxiliary tasks to emphasize ARDS-specific abnormalities^17,18^, thereby learning fine-grained and clinically relevant representations. Finally, the pretrained encoder is adapted to downstream objectives. By integrating these components into a unified framework, AutoARDS delivers accurate, efficient, and clinically coherent support for ARDS management.

**Fig. 1:**
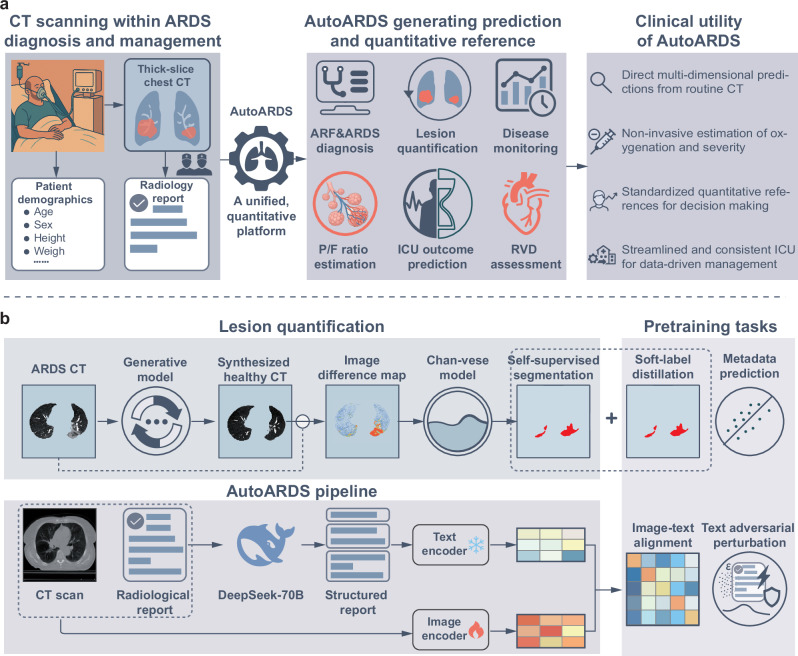
Overview of AutoARDS workflow and clinical integration. **a** Clinical workflow of AutoARDS. Routine chest CT within the current ARDS treatment pipeline is integrated through AutoARDS into a unified quantitative platform for ARDS assessment. The framework transforms conventional imaging interpretation into a stepwise, data-driven workflow: automatic identification of acute respiratory failure (ARF) and ARDS, lesion quantification and progress tracking, non-invasive estimation of the P/F ratio and severity, prediction of right-ventricular dysfunction (RVD), and ICU outcome forecasting. These multi-dimensional predictions also simultaneously serve as standardized quantitative references for streamlining decision-making and reducing reliance on serial invasive measurements. **b** AutoARDS pipeline and pretraining strategy. The pipeline combines lesion quantification with vision-language pretraining, in which CT volumes are aligned with radiology reports to capture fine-grained ARDS information. Text adversarial perturbation, soft-label distillation, and metadata prediction are incorporated to guide the model’s focus toward ARDS-specific abnormalities. The resulting pretrained encoders can be flexibly adapted to downstream tasks, providing a scalable and generalizable framework for clinical deployment.

To perform self-supervised lesion quantification, we propose a synthesis-contrast framework (Fig. 1b). Existing pneumonia segmentation methods developed for COVID-19 generalize poorly to ARDS, as ARDS lesions are far more diverse and heterogeneous, whereas COVID-19 segmentation models capture only a narrow subset of features (Supplementary Fig. 2a). To address this limitation, we design a framework that explicitly synthesizes healthy counterparts of diseased CT scans. Lung regions are first extracted from whole CT volumes to focus on the parenchyma, after which a generative model reconstructs the corresponding healthy lung structure^19^. Lesions are revealed as voxel-wise Hounsfield Unit (HU) deviations between the original and synthesized volumes, forming a difference map. By disentangling disease-induced alterations from intrinsic lung structure, the generative model mitigates over-reliance on specific lesion morphologies and thereby achieves robustness across heterogeneous lung pathologies. A variational model is then employed to refine the difference maps into accurate lesion masks. From these masks, eight quantitative indicators are extracted to provide a detailed characterization of lesion burden. Finally, both the refined segmentations and the derived indicators are integrated into downstream models, serving as informative priors tailored to ARDS-specific applications.

To capture fine-grained and diagnostically relevant information for ARDS, we design a task-unified multimodal pretraining framework (Fig. 1b). Given that ARDS diagnosis and management rely on diverse and multi-step information sources, this design aims to unify all routinely available clinical data into a single pretraining objective. This integration enables the model to learn how morphological patterns on CT scans correspond to textual and physiological semantics, thereby mirroring the multimodal reasoning process employed in real-world practice. The framework first incorporates the contrastive language-image pretraining (CLIP) paradigm^20^, aligning CT volumes with their corresponding radiological reports to learn joint representations. Beyond this baseline alignment, we introduce a text adversarial perturbation strategy^21,22^ to enhance sensitivity to detailed but clinically decisive characteristics. Specifically, for each structured radiology report, adversarial variants are generated by selectively altering critical descriptors, such as disease label, anatomical location, lesion distribution, severity, or texture, while leaving all non-lesion information unchanged. These targeted perturbations encourage the model to focus on the image features corresponding to the altered linguistic attributes, thereby improving its ability to capture ARDS-specific diagnostic signals. In addition, we extend the pretraining into a multimodal paradigm by jointly incorporating lesion soft-label distillation and metadata prediction. The former enforces explicit attention to lesion-derived features, anchoring the visual encoder to pathologically relevant signals, while the latter embeds demographic and acquisition factors to capture patient-specific information. This design not only disentangles fine-grained ARDS pathology from confounding context but also produces task-adaptive representations that remain robust across heterogeneous cohorts. Consequently, during downstream adaptation, AutoARDS leverages these pretrained embeddings to seamlessly support diverse objectives, from diagnosis and severity stratification to physiological estimation and outcome prediction, within a unified framework.

AutoARDS is pretrained on both the CT-RATE dataset^23^ and our multi-center ARDS cohort comprising 6,835 chest CT volumes from 6,153 individuals, with corresponding demographic information and radiology reports (Table 1). For subsequent analyses, all CT scans are categorized into four groups: healthy controls, localized lung disease (LLD, representing mild pulmonary abnormalities without respiratory failure), non-ARDS ARF (hereafter referred to as ARF), and ARDS. Built upon our datasets, AutoARDS supports a wide spectrum of downstream tasks, ranging from ARDS identification to ABG assessment, RVD estimation, and outcome prediction (Fig. 1a). By unifying these tasks within a single framework, AutoARDS not only enhances diagnostic accuracy but also improves efficiency in clinical management, thereby providing a foundational and scalable tool for timely analysis and decision-making in ARDS care.

**Table 1:** Detailed information of datasets for AutoARDS development

	Pretraining dataset	Internal dataset			External dataset		
Characteristics	CT-RATE	Center 1	Center 2	Center 3	Center 4	Center 5	Center 6
Number of CT volumes	47,149	2,502	1,105	1,528	741	451	508
Number of patients	21,304	2,366	875	1,528	741	274	369
Sex (M/F, %)	58.3/41.7	46.2/53.8	47.8/52.2	67.7/32.3	51.4/48.6	66.6/33.4	74.0/26.0
Age (years)	49.0 ± 17.4	53.2 ± 8.1	61.7 ± 14.5	76.5 ± 12.8	57.9 ± 9.8	67.4 ± 13.3	60.3 ± 17.9
Slice thickness (mm)	[0.50, 3.00]	[2.50, 5.00]	[1.25, 5.00]	[0.60, 5.00]	[2.00, 5.00]	[2.00, 5.00]	[1.00, 5.00]

### Self-supervised ARDS lesion segmentation

**Fig. 2:**
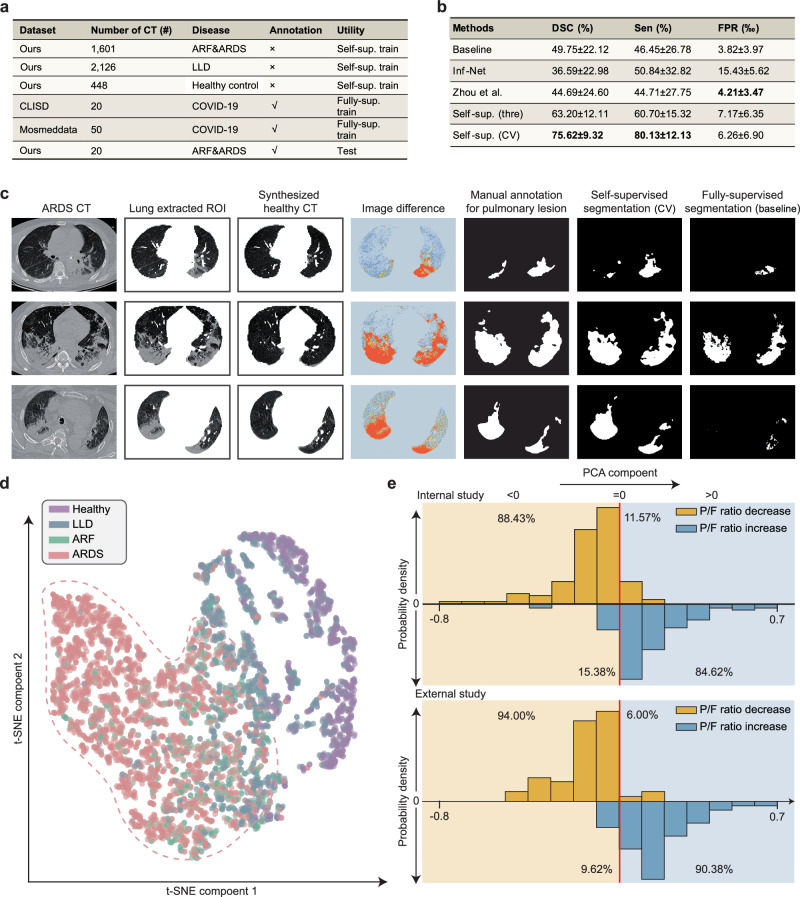
Quantitative CT-based assessment of pulmonary injury and disease progression in ARDS. **a** Summary of datasets used for training and evaluation of lesion quantification. **b** Quantitative performance of different segmentation approaches. On the annotated test set, the proposed self-supervised framework with Chan-Vese refinement achieved the highest Dice coefficient (75.6%) and sensitivity (80.1%) among the evaluated methods, while maintaining a low false-positive rate. **c** Representative workflow and visual results. AutoARDS synthesizes a virtual healthy lung from the diseased CT, computes voxel-wise image differences, and refines lesion masks, yielding segmentation results that showed encouraging agreement with expert annotations. **d** Two-dimensional t-SNE embedding of lesion-derived and demographic features demonstrates clear clustering of healthy, LLD, ARF, and ARDS cases, reflecting increasing disease burden along a quantitative axis. **e** Longitudinal tracking of ARDS progression using the first principal component (PC1) derived from lesion quantification. Changes in oxygenation status were defined by paired P/F ratio measurements obtained at two consecutive time points. The x-axis represents the change in PC1 values between paired scans (ΔPC1), where negative values indicate clinical improvement, and positive values indicate increasing lesion burden and disease deterioration. The red vertical line at 0 denotes no change. The y-axis represents the probability density of cases. In recovery cases (yellow, P/F ratio increase), PC1 values predominantly decrease (88.43% in the internal dataset and 94.00% in the external dataset). Conversely, in deteriorating cases (blue, P/F ratio decrease), PC1 values predominantly increase (84.62% in the internal dataset and 90.38% in the external dataset). The upper panel shows results from the internal dataset, and the lower panel shows results from the external datasets.

Accurate delineation of pulmonary lesions is clinically crucial for ARDS, as the extent and distribution of parenchymal involvement directly reflect disease burden and guide ventilatory management. We benchmark our proposed segmentation method against several state-of-the-art (SOTA) pneumonia segmentation approaches. These included two fully supervised baselines, Inf-Net^24^ and the method of Zhou et al.^25^, as well as a weakly supervised method, PointAnno^26^. To ensure fairness, we employ the publicly released models and checkpoints provided by the authors. In addition, we train a fully supervised baseline from scratch using nn-UNet^27^ on two public pneumonia datasets (CLISD^28^ and MosMedData^29^), comprising a total of 70 CT scans. Furthermore, we include experiments with the Chan-Vese (CV) model^30^ and direct thresholding as an ablation study. For evaluation on ARDS patients, we create an independent test set of 20 CT volumes, which is strictly excluded from training to avoid data leakage. Segmentation performance is assessed using the Dice similarity coefficient (DSC), sensitivity (Sen), and false positive rate (FPR). Both quantitative comparisons (Fig. 2b) and qualitative visualizations (Fig. 2c) are reported.

The results indicate that models trained exclusively on pneumonia datasets cannot be directly applied to ARDS lesion segmentation, resulting in poor performance and failed segmentation outcomes. Specifically, these models exhibit substantially lower sensitivity (below 60%), with a significant number of lesions left unidentified. This limitation can be attributed to the pronounced morphological differences between COVID-19 and ARDS lesions (Supplementary Fig. 2a). In contrast, our self-supervised method, learning from a diverse set of realistic ARDS examples, is capable of identifying the majority of lesions and demonstrates superior performance. It achieves a DSC of 75.62 ± 9.32% and a Sen of 80.13 ± 12.13%, which are markedly higher than those achieved by all other methods, while maintaining a competitive FPR (6.26 ± 6.90). Among the two refinement strategies evaluated, the CV-based method achieves better DSC and Sen values compared to thresholding. For subsequent studies, we utilize the segmentation results obtained through the CV-based refinement method. Collectively, these findings suggest that our self-supervised segmentation approach can identify heterogeneous ARDS lesions with promising performance on this limited evaluation set, thereby supporting downstream lesion characterization and physiological correlation in subsequent analyses.

### Lesion quantification enables clinically interpretable ARDS quantification and tracing

Quantitative assessment of pulmonary lesions on CT provides an objective basis for evaluating oxygenation impairment and for monitoring disease progression and treatment response. However, current radiological evaluations largely rely on subjective visual interpretation, resulting in limited reproducibility and substantial clinical workload. Drawing inspiration from the RALE score^31^, a clinical standard for assessing pulmonary edema based on lesion volume ratio, density, and location, we extract eight quantitative metrics from segmented lesions and lungs in CT scans^32^. Specifically, lesions are categorized into four groups based on their HU density: less than − 600 HU, between − 600 HU and − 400 HU, between − 400 HU and − 200 HU, and greater than − 200 HU. For each density category, the total lesion volume is calculated and normalized by the total lung volume to derive the volume ratio, which constitutes the first four metrics. Additionally, the average distance of lesions within each density category from the geometric center of the lung is computed to represent their spatial distribution, forming the remaining four metrics. Together, these eight metrics provide a comprehensive quantification of ARDS-associated lesions in terms of both volume and spatial distribution, thereby establishing the first reproducible CT-derived quantitative axis that overcomes the subjectivity of visual scoring systems and enables standardized comparison across patients and time points.

We compute the eight lesion-derived metrics for each CT volume and project them, together with the metadata including age and sex, into a low-dimensional space using the t-distributed stochastic neighbor embedding (t-SNE) algorithm. The clustering results are shown in Fig. 2c. The visualization reveals that four categories can be naturally distinguished and organized within the embedding space. Specifically, healthy controls cluster compactly on the right side, while LLD cases occupy the central transitional region. Non-ARDS ARF cases are distributed between the LLD and ARDS groups, reflecting their intermediate lesion burden. In contrast, ARDS cases form a distinct cluster on the left, characterized by markedly higher lesion involvement. This result highlights that ARDS can be directly characterized by its lesion features, while the quantification of lesions can also provide valuable insights to aid in ARDS diagnosis and differentiation.

We then conduct an analysis to evaluate whether lesion quantification can provide a universal and interpretable biomarker for monitoring ARDS progression. To this end, we employ principal component analysis (PCA) and identify the first principal component (PC1) as a compact yet physiologically meaningful biomarker. Unlike non-linear embeddings, PC1 offers both transparency and reproducibility, as its explicit linear formulation directly links lesion-derived metrics and metadata to disease severity:

1 $${\rm{PC}}1={\left(\begin{array}{c}0.1578\\ 0.6377\\ 0.6982\\ 0.2844\end{array}\right)}^{{\rm{T}}}\cdot \left(\begin{array}{c}{V}_{ < -600}\\ {V}_{ < -400}\\ {V}_{ < -200}\\ {V}_{\, > \,-200}\end{array}\right)+{\left(\begin{array}{c}0.0035\\ 0.0037\\ 0.0047\\ 0.0075\end{array}\right)}^{{\rm{T}}}\cdot \left(\begin{array}{c}{D}_{ < -600}\\ {D}_{ < -400}\\ {D}_{ < -200}\\ {D}_{\, > \,-200}\end{array}\right)+\left(\begin{array}{c}0.0005\\ 0.0069\end{array}\right)\cdot {\left(\begin{array}{c}{\rm{Age}}\\ {\rm{Sex}}\end{array}\right)}^{{\rm{T}}}$$

Here, $\left({V}_{ < -600},{V}_{ < -400},{V}_{ < -200},{V}_{\, > \,-200}\right)$ and $\left({D}_{ < -600},{D}_{ < -400},{D}_{ < -200},{D}_{\, > \,-200}\right)$ denote the normalized lesion volume ratios and centroid distances at different HU ranges as we aforementioned. This explicit formula ensures that any new CT scan can be directly projected onto the same progression axis, yielding a readily interpretable score that reflects the overall disease burden.

To validate this approach, we analyze paired CT scans and P/F ratios obtained from the same ARDS patients at different time points. If the P/F ratio increases at the second time point, the condition is considered improving; conversely, if the P/F ratio decreases, the condition is considered deteriorating. We then compare the changes in PC1 values between these paired time points. In the figure, the x-axis represents the change in PC1 values, with the red line at 0 indicating no change, while the y-axis represents the probability density of the paired CT scans, illustrating their distribution across varying changes in PC1 values. The results, presented in Fig. 2e for both internal and external datasets, reveal distinct patterns: in cases of ARDS recovery (P/F ratio increase, yellow), PC1 values predominantly decrease (88.43% in the internal dataset and 94.00% in the external dataset). Conversely, in deteriorating cases (P/F ratio decrease, blue), PC1 values predominantly increase (84.62% in the internal dataset and 90.38% in the external dataset). These findings demonstrate that PC1 serves as a consistent and reproducible quantitative axis and biomarker for monitoring ARDS progression or improvement with about 90% accuracy. Logistic regression analysis further confirms a significant correlation between PC1 values and changes in P/F ratio measurements (*p* =4.66 × 10^−15^). This longitudinal reproducibility highlights the potential of CT quantification not only as a diagnostic descriptor but also as a dynamic monitoring tool that supports ventilatory management and intervention timing.

SpO_2_ is currently the most commonly used metric to replace PaO_2_ when the ABG is not accessible. However, a recent study suggests that^33^, the SpO_2_/FiO_2_-ratio (S/F ratio) could lead to less precise results in identifying severity change categories between two consecutive datapoints. In contrast, AutoARDS may achieve higher accuracy when tracking clinical trajectories, representing a potentially more consistent monitoring. Such reproducible quantification establishes an imaging-based surrogate for physiological monitoring, enabling earlier recognition of deterioration and providing a quantitative foundation for subsequent oxygenation estimation and severity stratification.

### ARDS diagnosis

**Fig. 3:**
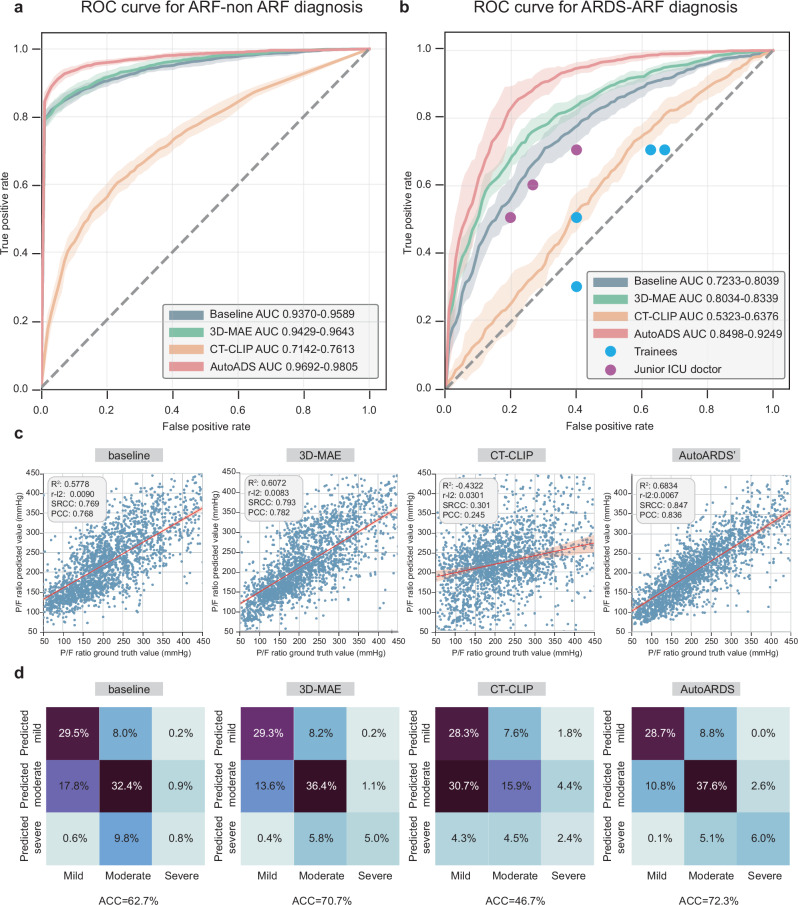
Comprehensive evaluation of ARDS diagnosis, P/F ratio estimation, and severity stratification on the internal dataset. **a** Receiver operating characteristic (ROC) curves for ARF diagnosis from all CT scans across different models. **b** ROC curves for ARDS detection from all ARF cases, with additional comparison to human performance (four trainees and three junior ICU doctors). **c** Scatter plots of predicted versus ground-truth P/F ratios, with correlation metrics reported for each method. **d** Confusion matrices for severity classification (mild, moderate, severe), with overall classification ACC reported for each method.

**Fig. 4:**
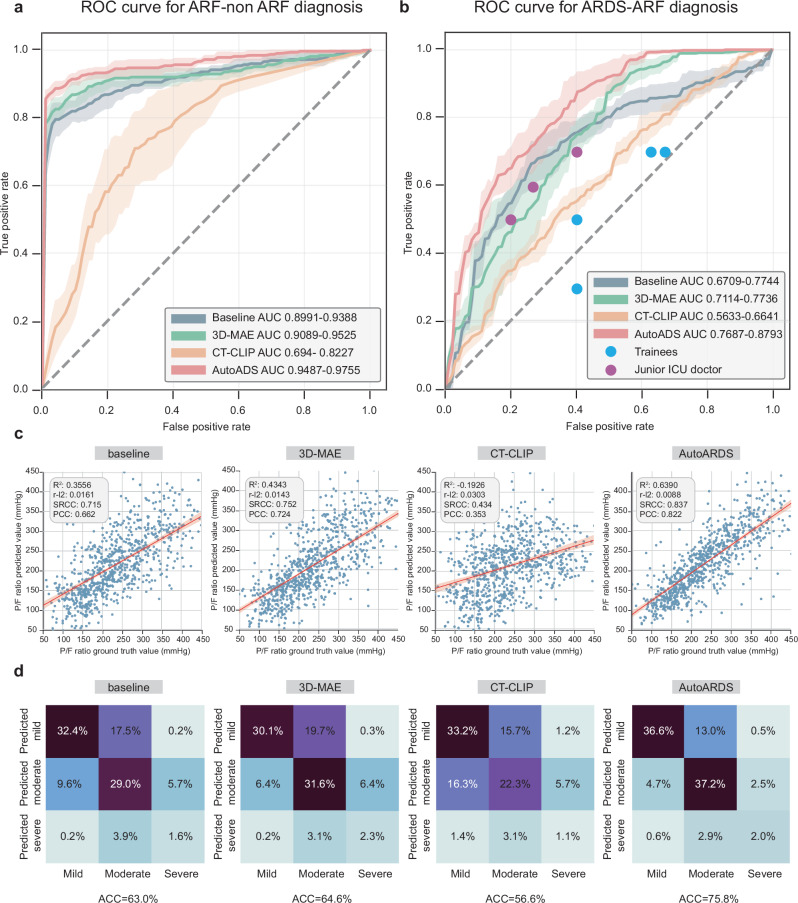
Comprehensive evaluation of ARDS diagnosis, P/F ratio estimation, and severity stratification on the external dataset. **a** Receiver operating characteristic (ROC) curves for ARF diagnosis from all CT scans across different models. **b** ROC curves for ARDS detection from all ARF cases, with additional comparison to human performance (four trainees and three junior ICU doctors). **c** Scatter plots of predicted versus ground-truth P/F ratios, with correlation metrics reported for each method. **d** Confusion matrices for severity classification (mild, moderate, severe), with overall classification ACC reported for each method.

Accurate CT-based recognition of ARDS is essential for clinical decision-making, enabling prompt differentiation from other acute pulmonary conditions and facilitating early, tailored intervention and mechanical ventilation strategy. Building on the reproducible CT-derived quantitative axis, we next evaluate whether AutoARDS can directly identify ARF and ARDS cases within the spectrum of lung parenchymal disease. To this end, we formulate the task as a two-stage supervised binary classification problem: (i) distinguishing ARF cases from all CT scans as an initial screening step, and (ii) detecting ARDS cases from the identified ARF cohort as a more refined assessment. Given the limited number of related studies on ARDS diagnosis, we compare AutoARDS with two representative pretrained CT foundation models, CT-CLIP^23^ and 3D MAE^34^. CT-CLIP employs an image-language contrastive learning strategy, representing a coarse-grained pretraining approach^23^, whereas 3D MAE is based on masked autoencoders, which have been shown to be more effective in capturing high-frequency information^35^. In addition, we include a baseline model with the same architecture as AutoARDS but without pretraining, to assess the contribution of pretraining to performance improvement. Only the decoder parameters of AutoARDS and the other pretrained models are optimized during fine-tuning, whereas the baseline model is trained from scratch. For internal datasets, we employ five-fold cross-validation and report the results across all folds. For external datasets, we report the averaged performance from five independently trained models. The corresponding results and receiver operating characteristic (ROC) curves are presented in Figs. 3 and 4.

In the internal dataset, for ARF classification (Fig. 3a), AutoARDS achieves a high AUC of 0.9748 (95% CI: 0.9692–0.9805), with an overall accuracy of 0.9275 (95% CI: 0.9216–0.9333) and an F1 score of 0.9282 (95% CI: 0.9224–0.9340). For ARDS detection among ARF cases (Fig. 3b), AutoARDS also reaches an optimal AUC of 0.8874 (95% CI: 0.8498–0.9249), with an overall accuracy of 0.8820 (95% CI: 0.8688–0.8952) and an F1 score of 0.9297 (95% CI: 0.9213-0.9381). The DeLong test^36^ confirms that AutoARDS achieves significantly superior classification performance compared with the second-best method (*p* = 3.55 × 10^−15^ for ARF and *p* = 4.00 × 10^−4^ for ARDS). Notably, CT-CLIP exhibits inferior performance, likely due to its coarse-grained training strategy that emphasizes global abnormality features^7,35^. When applied to ARDS-related tasks, which demand more fine-grained features, this limitation can substantially degrade performance. In the external dataset, AutoARDS also demonstrates robust performance, achieving an AUC of 0.9621 (95% CI: 0.9487–0.9755), with an overall accuracy of 0.9181 (95% CI: 0.9039–0.9322) and an F1 score of 0.9267 (95% CI: 0.9141–0.9393) for ARF (Fig. 4a), and an AUC of 0.8240 (95% CI: 0.7687–0.8793), with an overall accuracy of 0.8203 (95% CI: 0.8006-0.8399) and an F1 score of 0.8907 (95% CI: 0.8797–0.9017) for ARDS (Fig. 4b). Compared with the baseline model trained from scratch, AutoARDS exhibits less performance degradation, underscoring the effectiveness of pretraining in improving model generalizability across datasets.

We also conduct a reader study to compare AutoARDS with chest CT readers in ARDS detection from ARF cases, including three junior ICU doctors and four trainees, each of whom reviews 25 CT cases. Alongside the CT images, readers are provided with patient age and sex. For ARDS diagnosis, the performance of all readers falls below AutoARDS’s ROC curve (Fig. 3a, Fig. 4a). AutoARDS significantly outperforms all human readers, surpassing trainees by an average F1 score margin of 0.4463 and junior ICU doctors by 0.2618. Notably, the weakest-performing trainee achieves an F1 score as low as 0.2857, underscoring the difficulty of consistent interpretation in borderline ARDS cases without additional ABG results or prior imaging context.

To further contextualize human performance variability, we assessed inter-reader agreement within each reader group. Among the four trainees, inter-reader agreement was low (Fleiss’ *κ* = 0.188), with an overall agreement of 60.0%. Among the three junior ICU doctors, agreement was higher but remained moderate (Fleiss’ *κ* = 0.340), with an overall agreement of 68.0%. These findings indicate that, although human readers often struggle to interpret borderline ARDS cases using CT alone, particularly in the absence of ABG measurements, AutoARDS effectively captures implicit physiological characteristics embedded within the CT scans. This capability enables AutoARDS to emulate ABG-assisted interpretation, thereby supporting more flexible diagnostic decisions. Consequently, automated CT-based diagnosis may serve as a viable surrogate for invasive physiological assessments in routine clinical settings. Previous studies have reported that the S/F ratio can be affected by heterogeneous clinical or ventilatory settings, occasionally resulting in notable classification errors^33^. In contrast, AutoARDS establishes a CT-based, physiologically grounded alternative that enhances reliability and interpretability for ARDS identification when arterial blood gas analysis is unavailable.

### P/F ratio estimation and severity classification

Accurate oxygenation assessment is pivotal for ARDS diagnosis and management, yet the P/F ratio relies on invasive and often delayed ABG sampling. To address this limitation, we evaluate whether AutoARDS can serve as a non-invasive, CT-based surrogate for physiological oxygenation, directly inferring the P/F ratio from routine imaging to bridge morphology and physiology. In this regression task, ground-truth P/F ratios are obtained from matched ABG analyses and FiO_2_ records acquired within a 2-hour window of CT imaging. Regression performance is assessed using *R*^2^, mean absolute percentage error (MAPE), Spearman’s rank correlation coefficient (SRCC), and Pearson correlation coefficient (PCC). Higher values of *R*^2^, SRCC, and PCC indicate better estimation accuracy and stronger correlations. The MAPE metric, defined as

2 $${\rm{MAPE}}=\frac{1}{N}\mathop{\sum }\limits_{i=1}^{N}\left(\frac{| {y}_{i}-{\widehat{y}}_{i}| }{{y}_{i}}\right)\times 100 \% ,$$

is employed to measure relative errors with respect to the ground-truth P/F ratios, where *y*_*i*_ and ${\widehat{y}}_{i}$ denote the ground-truth and predicted values for the *i*-th sample, respectively. Lower MAPE values indicate better performance.

In the internal dataset (Fig. 3c), AutoARDS showed the strongest agreement with the ground-truth P/F ratio, achieving a SRCC of 0.848 (95% CI: 0.832–0.862), PCC of 0.833 (95% CI: 0.808–0.855), *R*^2^ of 0.694 (95% CI: 0.653–0.731), and MAPE of 20.221% (95% CI: 19.341%–21.109%). Its predicted P/F ratio distribution (217.5 ± 91.8 mmHg) was also close to the ground-truth distribution (229.8 ± 118.7 mmHg), with a slight bias of -12.3 mmHg. Among the comparison models, the 3D-MAE pretrained model outperformed the baseline, suggesting that task-relevant volumetric pretraining improves regression performance, whereas CT-CLIP showed substantially weaker estimation ability, indicating that coarse-grained cross-modal pretraining may be less suitable for this fine-grained physiological prediction task.

In the external dataset (Fig. 4c), AutoARDS maintained robust generalization, achieving a SRCC of 0.839 (95% CI: 0.810–0.864), PCC of 0.822 (95% CI: 0.778–0.858), *R*^2^ of 0.676 (95% CI: 0.606–0.737), and MAPE of 19.569% (95% CI: 17.215%–22.426%). The predicted distribution (223.9 ± 91.0 mmHg) remained close to the ground-truth value (243.0 ± 106.4 mmHg), with a slight bias of -19.1 mmHg, again indicating limited systematic deviation. By contrast, the baseline, 3D-MAE, and CT-CLIP models all showed lower concordance and higher relative error. Previous studies have estimated P/F ratios indirectly from S/F ratios^33,37^, typically reporting *R*^2^ values around 0.44–0.48 and correlations near 0.69. In comparison, AutoARDS provides a CT-based estimate with stronger agreement to directly measured oxygenation. Detailed quantitative results are reported in Supplementary Table 1(a).

P/F ratio estimation further enables direct severity stratification under the Berlin definition^38^. Specifically, ARDS severity was categorized as mild (200–300 mmHg), moderate (100–200 mmHg), and severe ( < 100 mmHg). Confusion matrices for the internal and external datasets are shown in Fig. 3d and Fig. 4d, respectively, and detailed class-level results are provided in Supplementary Table 1(b). AutoARDS achieved the best overall classification performance across datasets, with class-wise accuracies of 0.756/0.767/0.926 for mild/moderate/severe ARDS in the internal dataset and 0.744/0.815/0.951 in the external dataset. It also showed the most balanced class-level performance overall, with F1-scores of 0.624/0.716/0.494 internally and 0.656/0.752/0.382 externally, outperforming the comparison models in most severity categories. Notably, discrimination of severe ARDS remained more challenging across all methods, but AutoARDS still preserved substantially better sensitivity and precision than the alternatives. These findings indicate that AutoARDS not only estimates continuous oxygenation status with high fidelity, but also supports clinically meaningful non-invasive severity stratification from CT.

### ARDS prognosis prediction

Early risk stratification is crucial for guiding intervention and optimizing resource allocation in ARDS management, providing clinicians with actionable insights into disease trajectory, treatment responsiveness, and survival likelihood to inform timely and individualized care. Leveraging the unified imaging-physiology representations from AutoARDS, we further investigate whether it can predict clinical outcomes directly from CT, offering a rapid and non-invasive approach to survival estimation and triage support. Specifically, we apply AutoARDS to prognosis prediction, aiming to estimate survival likelihood and support early triage and critical care planning. The task is formulated as a discrete-time survival prediction problem, estimating the probability of survival for each patient up to 28 days post-scan. In addition to the embedded features described above, including lesion quantification and radiological report embeddings, the predicted P/F ratio is also incorporated as a reference. Model performance is evaluated using time-dependent AUC, which measures discrimination accuracy at each follow-up day, across both internal and external datasets.

**Fig. 5:**
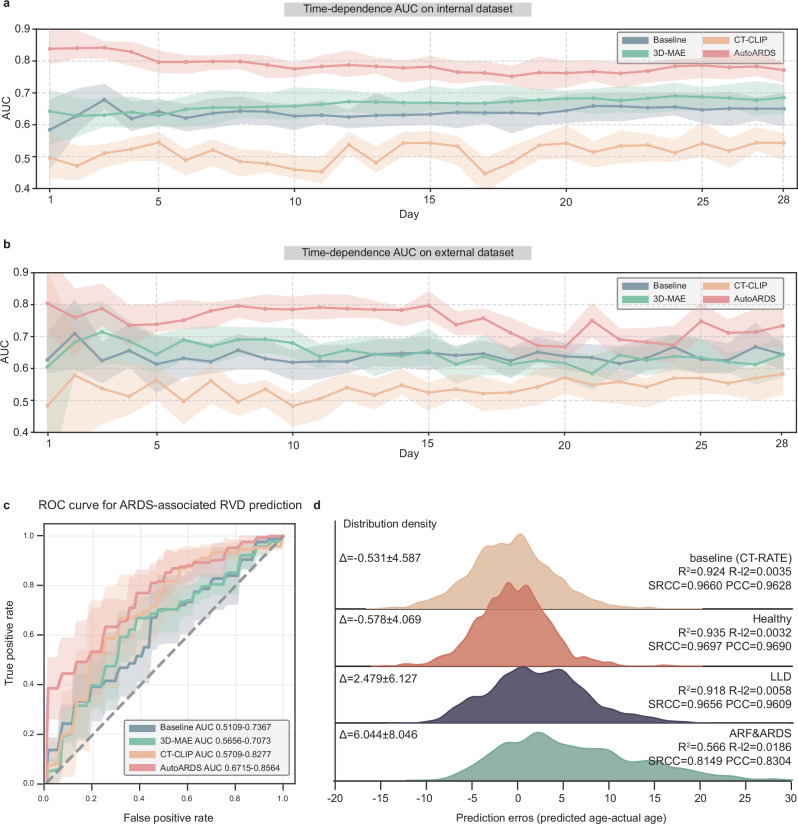
Comprehensive evaluation of ARDS prognosis, RVD prediction, and imaging-derived age. **a** Time-dependent AUC curves for 28-day survival prediction on the internal dataset. Shaded areas represent 95% confidence intervals. **b** Time-dependent AUC curves for 28-day survival prediction on the external dataset. Shaded areas represent 95% confidence intervals. **c** ROC curves for ARDS-associated RVD prediction among ARDS patients. **d** Imaging-derived age estimation across patient groups. ARDS patients exhibit a systematic positive shift in predicted age compared to healthy individuals.

The results on the internal dataset are shown in Fig. 5a, where AutoARDS consistently outperforms all baseline and pretrained models across the entire 28-day follow-up period. Specifically, AutoARDS achieves a stable AUC trajectory, maintaining performance around 0.80 from Day 1 to Day 28 (time-averaged AUC: 0.7872), presenting higher accuracy than previously reported results based on conventional APACHE-II indices^39^. In contrast, all other models exhibit substantially lower AUCs (time-averaged AUCs ranging from 0.5100 to 0.6642), with greater temporal variation and pronounced performance degradation over time. On the external dataset (Fig. 5b), AutoARDS again demonstrates a clear performance advantage, achieving a time-average AUC of 0.7477 across the follow-up period, exceeding other models by 0.10 to 0.21. Notably, performance degradation over time is more evident in the external setting for all models, likely reflecting differences in clinical decision pipelines and variations in patient management strategies across institutions. Nevertheless, AutoARDS exhibits more stable trajectories than all comparison methods, thereby underscoring its generalizability for outcome prediction. These robust results reinforce the potential of AutoARDS for real-world risk stratification at the time of imaging. Unlike APACHE-II, which requires extensive physiological and laboratory variables, AutoARDS achieves higher prognostic accuracy directly from CT, substantially lowering the barrier for large-scale deployment. These findings suggest that AutoARDS effectively captures prognostic markers and delivers survival estimates directly from routine CT imaging, enabling clinicians to assess patient risk without additional laboratory testing. Clinically, this capability provides actionable support for ICU triage, identification of high-risk patients, and optimization of resource allocation, particularly in settings where rapid, non-invasive, and reproducible prognostic assessment is critical.

### ARDS-associated RVD prediction

RVD represents a frequent and clinically significant complication of ARDS, reflecting the cardiopulmonary interaction between increased pulmonary vascular resistance and right heart strain. Early detection of RVD is critical, informing ventilatory adjustment, fluid management, and hemodynamic support. However, its diagnosis primarily relies on echocardiography, which could be operator-dependent and less routinely feasible for frequent or bedside assessment. To explore whether AutoARDS can provide a rapid alternative for identifying this complication, we evaluate its ability to predict RVD directly from chest CT scans and associated metadata.

This analysis is performed in 252 ARDS patients from Center 2, where echocardiographic labels are available. The task is formulated as a binary classification problem, differentiating ARDS patients with versus without RVD. AutoARDS achieves a moderate but clinically meaningful AUC of 0.7640 (95% CI: 0.6715–0.8564), outperforming all comparison models (Fig. 5c). The ROC curves further demonstrate consistently higher sensitivity across thresholds, with an average F1 score of 0.7708 (95% CI: 0.7226–0.8191). Notably, CT-CLIP shows partial improvement due to its exposure to cardiac features such as coronary calcification and pericardial effusion, suggesting that cardiac-relevant pretraining benefits RVD-related inference.

These findings highlight the feasibility of extending AutoARDS beyond pulmonary assessment toward integrated cardiopulmonary profiling. Although its performance remains constrained by limited training data and the heterogeneous presentation of RVD on CT, AutoARDS demonstrates that subtle imaging signatures of right heart strain can be captured within standard thoracic scans. With larger datasets and multimodal integration of echocardiographic or hemodynamic data, this framework may evolve into a practical tool for early, non-invasive detection of ARDS-associated cardiac dysfunction and more holistic critical-care decision support.

### AutoARDS enables CT-based age estimation

The metadata estimation module incorporated during pretraining enables direct estimation of CT-derived age without additional fine-tuning. We applied the pretrained age prediction to the ARDS cohort and defined the difference as the prediction residual, Δ = Age_pred_ − Age_chrono_, and examined its distribution across healthy individuals, patients with LLD, and patients with ARDS.

As shown in Fig. 5d, AutoARDS preserves high age prediction fidelity on the internal validation set and on healthy subjects within the ARDS cohort (PCC = 0.9690, SRCC = 0.9697), with residuals tightly centered around zero (Δ = − 0.531 ± 4.587 years and Δ = − 0.578 ± 4.069 years, respectively), indicating robust generalization of the pretrained age head. In contrast, in patients with lung pathology, the residual distributions exhibit systematic shifts and increased dispersion. Patients with LLD show a moderate positive shift in predicted age (Δ = + 2.479 ± 6.127), while ARDS patients demonstrate a markedly broadened and right-skewed residual distribution (Δ = + 6.044 ± 8.046). Notably, the majority of ARDS patients remain clustered near zero residuals, whereas a distinct subset exhibits substantially elevated age predictions, forming a pronounced positive tail. This pattern suggests that the image-derived age signal in ARDS captures heterogeneous disease-associated imaging phenotypes. Rather than indicating true biological aging per se, the observed positive residuals may reflect disease-related structural alterations, cumulative lung injury, and impaired pulmonary health status in a subset of patients. Accordingly, the age prediction residual may serve as an imaging-based marker associated with structural and physiological burden. Together with RVD estimation, these findings suggest that AutoARDS extends beyond ARDS diagnosis and severity assessment toward broader characterization of disease-associated phenotypes and potential systemic risk.

## Discussion

This study demonstrates that routinely acquired chest CT can be transformed into a quantitative platform for evaluating the physiological and pathological continuum of ARDS. By decoding physiological information embedded in imaging data, AutoARDS extends the role of CT beyond morphological visualization, assigning it an additional clinical value as a reproducible, quantitative, and physiologically interpretable reference within existing care workflows. Through integrated assessment of lesion burden, ARDS diagnosis, oxygenation-related severity, prognosis, and associated complications, AutoARDS contributes to greater consistency, transparency, and reproducibility in ARDS evaluation and management.

Importantly, CT-based P/F ratio estimation in AutoARDS is intended to play a complementary rather than replacement role in clinical practice. ABG analysis remains the clinical gold standard and routine bedside monitoring in the ICU. Within this context, the value of AutoARDS lies in providing an additional, non-invasive, and standardized physiological reference at imaging time points, particularly when chest CT is already being performed for diagnostic evaluation, reassessment after deterioration, follow-up of lung injury, or exclusion of complications. In such settings, AutoARDS can generate a simultaneous oxygenation-related estimate and Berlin-aligned severity reference from the same examination, thereby enriching the quantitative interpretation of that CT time point. This complementary function may be especially relevant when ABG quality, timeliness, or availability is suboptimal, such as when arterial sampling is delayed, technically difficult, poorly tolerated, relatively contraindicated, or not temporally aligned with imaging. Other practical scenarios include pain-sensitive patients, repeated puncture burden, early triage, inter-hospital transfer, incomplete physiological documentation, and resource-constrained or surge settings, while AutoARDS still operates within established ICU practices rather than replacing them.

Beyond its immediate application, AutoARDS illustrates a broader shift in the role of medical imaging, from a morphological tool to a platform capable of encoding physiologically meaningful information. Recent progress in AI-based representation learning has shown that medical images contain latent quantitative signals that reflect systemic physiological states^40–42^. Leveraging this potential, AutoARDS demonstrates how visual features can be integrated with physiological context to support a more comprehensive, system-level understanding of disease. Such integration aligns with the direction of multimodal diagnostics^43^, and lays the groundwork for data-driven critical care^44^, where imaging-derived metrics complement existing monitoring tools to guide timely and personalized decisions.

In particular, AutoARDS benefits from a pretraining strategy that incorporates imaging, radiology reports, patient metadata, and physiological measurements into unified representations. This design enables the model to capture clinically coherent patterns that mirror real-world diagnostic reasoning. Furthermore, adversarial perturbation learning enhances sensitivity to subtle yet meaningful variations in lesion characteristics and textual descriptions^45^, improving both quantitative accuracy and physiological interpretability. Together, these components highlight a generalizable framework for building physiologically grounded and data-efficient medical imaging models.

The observed age residual shift in ARDS patients is highly heterogeneous, characterized by a near-zero central mode and a pronounced right-skewed tail. This distribution indicates that the positive shift in mean ΔAge is not driven by a uniform effect across the cohort, but rather by a subset of patients with imaging patterns associated with more extensive lung injury and structural alteration. These disease-associated imaging features may partially overlap with patterns more commonly observed with advancing age, thereby contributing to the elevated group-level mean residual. Accordingly, the image-derived age signal in this context may be more cautiously interpreted as an imaging-based marker associated with disease burden and reduced pulmonary reserve, rather than a direct measure of true biological aging. Future work incorporating explicit calibration strategies based on well-defined healthy reference populations may further help disentangle disease-related imaging effects from physiological aging processes.

Notwithstanding its promise, this study remains retrospective in design. Prospective, multicenter validations will be essential to assess the real-world impact of AutoARDS on diagnostic efficiency, clinical decision-making, and patient outcomes^46^. Further investigations should clarify how CT-based physiological estimation interacts with conventional measurements, whether as a supplementary index when ABG is unavailable or as an integrative component within broader decision frameworks. Expanding AutoARDS to other forms of acute and chronic lung injury may further reveal how quantitative imaging can generalize across respiratory pathophysiology. In addition, lesion segmentation was evaluated on a limited set of 20 CT scans. Given the irregular and heterogeneous nature of ARDS-related pulmonary lesions, voxel-level annotation is particularly labor-intensive and requires substantial expert effort. Although the current segmentation performance is encouraging, it should still be regarded as preliminary. Future work should focus on establishing larger annotated cohorts to enable more comprehensive evaluation of segmentation robustness and generalizability across diverse scanners, institutions, and clinical settings. In summary, AutoARDS redefines the clinical and scientific scope of chest CT, transforming it from a subjective morphological record into quantitative information and establishing a scalable foundation for unified, standardized, and data-driven critical care.

## Methods

### Dataset establishment and disease determination

In this experimental framework, the diagnosis and severity assessment of ARF follows the common standard^47^, with the following two criteria: (1) a PaO_2_/FiO_2_ ratio (P/F ratio) below 300 mmHg, and (2) chest imaging (CT in our study) showing evident pulmonary parenchymal lesions, which cannot be fully attributed to effusions, lobar or lung collapse, or nodules, while the respiratory failure should not primarily result from cardiac failure or fluid overload. In addition, ARDS is regarded as a more severe subset of ARF, defined by the acute onset (within one week of a known clinical insult or new/worsening respiratory symptoms), the need for ventilatory support with at least 5 cmH_2_O positive end-expiratory pressure (PEEP) to maintain adequate gas exchange, and the presence of bilateral pulmonary opacities not fully explained by effusions, lobar collapse, or nodules, in accordance with the Berlin definition^38^. This definition further stratifies ARDS into mild (200–300 mmHg), moderate (100–200 mmHg), and severe ( < 100 mmHg) based on the degree of hypoxemia.

The diagnosis of right ventricular dysfunction (RVD) is established through a comprehensive clinical evaluation that includes assessment of clinical signs, echocardiography, chest CT imaging, and electrocardiography (ECG), with echocardiography serving as the primary diagnostic tool. Echocardiographic assessment provides direct visualization of right ventricular size and function, enabling quantitative evaluation of right ventricular fractional area change (FAC), tricuspid annular plane systolic excursion (TAPSE), right ventricular ejection fraction (RVEF), and interventricular septal motion abnormalities. Reduced TAPSE (<16 mm), FAC (<35%), or an increased RV/LV end-diastolic area ratio (>0.6), often accompanied by septal dyskinesia, can support the diagnosis of RVD^48^. The final diagnosis is ultimately determined by the comprehensive diagnostic report.

### Involved datasets

In the pretraining phase, we employ two large-scale datasets: the CT-RATE dataset and our multi-center ARDS cohort. The CT-RATE dataset^23^ comprises 21,304 patients collected in Istanbul. This dataset covers a broad spectrum of clinical abnormalities, including parenchymal diseases (e.g., consolidation, pulmonary fibrotic sequelae, pleural effusion) and other pulmonary conditions (e.g., hiatal hernia, lymphadenopathy). Each CT volume is accompanied by detailed patient metadata as well as paired radiological findings and impressions.

In addition, we also established a multi-center ARDS dataset consisting of 6,835 chest CT volumes from China, as detailed in Table 1. Specifically, the centers listed in the table correspond to the following institutions: Center 1—the First Affiliated Hospital of Harbin Medical University; Center 2-the Fourth Affiliated Hospital of Harbin Medical University; Center 3—OMIX, China National Center for Bioinformation/Beijing Institute of Genomics, Chinese Academy of Sciences (No.OMIX006496)^49^; Center 4—Mudanjiang First People’s Hospital; Center 5—Hainan General Hospital, and Center 6—Department of Critical Care Medicine, Zhongda Hospital, School of Medicine, Southeast University.

To ensure the integrity of the radiology reports included in our dataset, we initiated the process by anonymizing the reports, thereby removing all personal information pertaining to patients and medical professionals. Following this step, the anonymized reports were translated from Chinese to English using the GPT-4o API. Each translated report underwent meticulous review to verify coherence and to confirm the removal of any residual personal details. Ultimately, only the verified English translations were incorporated into our dataset. For the CT volumes, scans that did not focus on the chest region or originated from patients under the age of 18 were excluded. Additionally, CT volumes with suboptimal image quality, such as those exhibiting significant noise or artifacts, were removed (Supplementary Fig. 3). The metadata associated with the CT volumes was also rigorously cleaned to ensure the complete elimination of any identifying information related to patients or medical professionals.

### Thick-slice super-resolution

In clinical practice, thick-slice CT scanning is widely employed due to concerns about radiation exposure and equipment limitations, particularly in cases requiring multiple follow-up scans. However, the use of thick slices results in low resolution, which introduces sparse spatial information, while the increased slice thickness leads to severe spatial anisotropy. This anisotropy has been shown to cause significant performance degradation in deep learning-driven models^26,50^. Therefore, an appropriate thick-slice super-resolution process is essential before model development. Specifically, for any CT volume with an inter-slice thickness exceeding 2.50 mm, we utilize the CTHNet model^51^ to super-resolve the data to a resolution of 1.00 mm. Supplementary Fig. 1 presents two examples of the thick-slice super-resolution. Compared to interpolation, deep learning-based super-resolution methods can preserve fine-grained details and enhance spatial continuity while effectively reducing blurring and potential artifacts.

### Data normalization

To preserve long-contextual attention to the entire CT dataset, we introduce a novel data normalization method instead of cropping the entire volume into smaller cubes, as is commonly done in most 3D volume processing approaches^27^. Since the diagnosis and treatment of ARDS primarily focus on the lung parenchyma, our method aims to isolate the region containing the lung parenchyma from the full CT volume. This approach not only excludes irrelevant information within the CT volume, thereby improving the model focus, but also reduces the overall data size.

To achieve this, we first generate a lung mask from the CT volume data using the method described in “LungMask”^32^. Based on the segmented lung, we construct a bounding box and crop the entire CT volume accordingly. The cropped data is then resampled into a standardized space with dimensions 224 × 320 × 224 and a spatial resolution of 1 × 1 × 1.5mm^3^ (coronal, sagittal, and axial views, respectively). Regions exceeding the target size are cropped, while insufficient areas are padded with zeros. This ensures a lung region size of 22.4 × 32.0 × 33.6, which exceeds the typical lung dimensions of most healthy individuals. Our experiments also demonstrate that this configuration accommodates the entire lung region in over 99% of cases. Additionally, the reduced volume size is well-suited for model training, requiring approximately 10GB of VRAM per case on a GPU with half-precision training. Furthermore, the voxel intensity of all scans is truncated within a Hounsfield Unit (HU) range of [− 1000, 600] and normalized to a scale of [0, 1].

### Self-supervised lesion segmentation

Achieving lesion segmentation in ARDS provides substantial visual information and quantitative indicators that are critical for ARDS diagnosis and treatment monitoring. Deep learning-driven segmentation algorithms typically require large datasets with manual annotations serving as ground truth to ensure adequate model development and validation. However, manual annotation of lesions in a 3D context is exceedingly labor-intensive, and the establishment of sufficiently large datasets requires considerable costs and time. On the other hand, many cases of ARDS develop from pneumonia, and many studies have been developed to focus on pneumonia segmentation, particularly in the context of COVID-19^24–26,52^. Despite this, annotated 3D datasets remain generally scarce^28,29^, and the lesion characteristics of ARDS differ considerably from those of COVID-19 infections. Specifically, COVID-19 is typically characterized by predominantly peripheral ground-glass opacities and occasional crazy paving patterns, whereas ARDS exhibits much greater diversity, including both diffuse high-density consolidations and sub-visual lesions, accompanied by higher rates of pleural effusions^25,53^. These substantial differences in lesion characteristics, coupled with the scarcity of annotated COVID-19 datasets, frequently lead to poor or even failed segmentation performance when models are developed solely using existing COVID-19 datasets (Supplementary Fig. 4.b, c).

To address these challenges, we propose a self-supervised lesion segmentation approach, which achieves superior performance on ARDS cases (Fig. 2a, Supplementary Fig. 2b). The whole method can be divided into two parts: paired healthy data synthesis and segmentation refinement. The main idea is to synthesize CT data with healthy lung parenchyma from CT with pulmonary parenchymal lesions, while the differences between the raw and synthesized data can present the potential parenchyma lesions.

#### Paired healthy data synthesis

In our study, we utilize the Cycle-Consistent Generative Adversarial Network (CycleGAN)^19^ to synthesize healthy CT from ARF and ARDS cases by learning the mapping between unpaired abnormal and healthy cases. The CycleGAN framework comprises two generators (*G* and *F*) and two discriminators (*D*_*A*_ and *D*_*N*_), which operate adversarially to translate data between unpaired abnormal CT (*A*) and normal healthy CT (*N*). The generators, sharing the same architecture, are designed to map images between the two domains (*G*: *A* → *N* and *F*: *N* → *A*), while the two discriminators assess whether the generated images appear realistic within their respective domains.

Specifically, the generator network in our framework is built upon a 3D convolutional architecture to effectively process CT data. The architecture begins with a series of convolutional layers: an initial 3D convolution with a kernel size of 7 × 7, followed by two 3D convolutions with kernel sizes of 3 × 3 × 3. These layers progressively downsample the input spatial dimensions while preserving essential features. The core of the generator consists of six ResNet blocks, each incorporating reflection padding, instance normalization, and ReLU activation, which collectively enhance feature extraction and maintain spatial consistency. After the encoding phase, transposed convolutional layers are employed to upsample the data back to its original resolution, ensuring the synthesized output retains the input’s spatial dimensions. Dropout regularization is applied during this process to mitigate overfitting and improve generalization.

The discriminator network is composed of multiple 3D convolutional layers, each followed by instance normalization and LeakyReLU activation. These layers progressively reduce the spatial dimensions while increasing the number of feature map channels. A final 1 × 1 × 1 convolutional layer converts the channel dimension to 1. Subsequently, a pooling layer and a multi-layer perceptron (MLP) are applied to produce the final discrimination results.

The CycleGAN training pipeline is built upon two fundamental components: adversarial learning and cycle-consistency regularization. The objective is to simultaneously train the generator networks (*G* and *F*) and the discriminator networks (*D*_*A*_ and *D*_*H*_), ensuring that the generated images are both realistic and cycle-consistent. The training process alternates between updating the generators and the discriminators. Adversarial loss is used to guarantee that the generated images are indistinguishable from real images in their respective domains. For the generator *G*: *A* → *H*, the adversarial loss is defined as:

3 $${{\mathcal{L}}}_{GAN}(G,{D}_{H},A,N)={{\mathbb{E}}}_{n \sim {p}_{N}}[\log {D}_{N}(n)]+{{\mathbb{E}}}_{a \sim {p}_{A}}[\log (1-{D}_{N}(\widehat{a}))].$$

Here, *G* aims to generate images $\widehat{a}=G(a)$ that match the distribution of healthy CT, while *D*_*N*_ endeavors to differentiate these generated data from real healthy CT. Similarly, the adversarial loss ${{\mathcal{L}}}_{GAN}(F,{D}_{A},N,A)$ for *F*: *N* → *A* is employed as well. To enforce consistency and reversibility in the image translations, we employ the cycle-consistency loss^19^. The forward cycle-consistency loss ensures that if data *a* from disease CT *A* is translated to healthy CT *N* via *G*, and then mapped back to disease CT using *F*, the reconstructed image $F(\widehat{a})$ should remain close to the original input *a*. This constraint is formalized as:

4 $${{\mathcal{L}}}_{cyc}^{{\rm{forward}}}(G,F)={\mathbb{E}}a \sim {p}_{A}[| | F(\widehat{a})-a| {| }_{1}],$$

where ∣∣ ⋅ ∣∣_1_ denotes the *L*_1_ norm, which measures the pixel-wise difference between the reconstructed image and the original input. Similarly, the backward cycle-consistency loss ${{\mathcal{L}}}_{cyc}^{\,{\rm{backward}}}(F,G)$ is also employed to ensure the reversibility of healthy CT translations. The overall objective function is defined as the combination of the bidirectional adversarial losses and the cycle-consistency loss.

#### Segmentation refinement

Because the lesions associated with ARDS, such as pneumonia infection, consolidation, and fibrosis, typically exhibit higher HU values compared to healthy lung parenchyma (at least -600 HU for lesions versus approximately -850 HU for lung parenchyma)^26^, we can compare the HU value differences between the original ARDS CT and the corresponding healthy CT to locate ARDS lesions. Given the synthesized healthy CT, $\widehat{a}$, derived from ARDS CT *a*, the difference can be calculated as $d=\min \left(0,a-\widehat{a}\right)$. From the computed differences, we can obtain the segmentation mask *s* using both a thresholding strategy and a variational model.

For the thresholding strategy, the difference threshold is set as − 600 − ( − 850) = 250 HU^26^, and the segmentation mask is determined as follows:

5 $$s=\left\{\begin{array}{l}1\,{\rm{if}}\,d > 250\\ 0\,{\rm{otherwise}}\end{array}\right.$$

However, due to image noise and prediction errors, directly applying the thresholding strategy can produce numerous small, isolated false-positive regions as well as fragmented true-positive regions, thereby reducing the accuracy and quality of the final segmentation. To address this issue, we propose employing a variational model, which is a widely used approach in medical image segmentation^26^. The variational model minimizes an energy function to ensure that contours align with object boundaries, incorporating image features such as intensity, gradient, and texture. In our implementation, we first normalize the differences from the range [0, 600] to [0, 1] and then apply the CV method on the normalized differences to generate the segmentation mask. The Chan-Vese (CV) method, implemented via the scikit-image package, was configured with parameters *μ* = 0.05, *λ*_1_ = 5, and *λ*_2_ = 1, with a maximum of 200 iterations in our experiments. All the segmentation masks are then multiplied by the lung mask to avoid any false-positive regions outside the lung.

#### Ground truth annotation

To evaluate segmentation performance, we created a small subset of an ARDS dataset with manual annotations. First, we selected 20 CT scans from our internal dataset using a severity-stratified sampling strategy. Specifically, cases were categorized according to disease severity across the ARF spectrum, including non-ARDS ARF, mild ARDS, moderate ARDS, and severe ARDS based on the Berlin definition. Five representative cases were then randomly selected from each severity stratum, resulting in a total of 20 CT scans that collectively reflect the heterogeneity of ARDS-related pulmonary manifestations. Two experienced radiologists, each with over 10 years of experience, were invited to independently annotate the pulmonary lesions within the lungs. To ensure accuracy, a third radiologist reviewed the annotations and determined the final annotation by comparing and combining the two initial annotations. All manual annotations were performed using 3D Slicer^54^.

### Model pretraining

In this section, we propose a vision-language pretraining strategy specifically tailored for ARDS. Existing vision-language pretraining algorithms are generally designed to learn cross-modal representations between vision and language, thereby enhancing performance on downstream tasks. These methods can be broadly categorized into contrastive learning approaches (e.g., CLIP^20^, ALIGN^55^), generative learning approaches (e.g., SimVLM^56^, Flamingo^57^), and autoencoder-based methods (e.g., MAE^58^, BEiT-3^59^). However, these algorithms are predominantly developed for natural 2D images, and their image representations and semantic information often cannot generalize to medical imaging tasks^60^. Moreover, in the context of ARDS diagnosis, medical imaging requires precise and fine-grained representations of features such as the location, distribution, area, and severity of lung parenchymal lesions. Nonetheless, existing algorithms often exhibit a bias toward coarse-grained information^35,61^, rendering them insufficient for capturing the level of fine-grained information required in ARDS diagnosis. To address these limitations, we propose a novel approach that learns fine-grained descriptions from paired radiological reports by aligning cross-modal representations. Furthermore, we introduce a multi-task synchronous learning framework that incorporates ARDS segmentation and metadata information inference, enabling the model to focus specifically on lung parenchymal lesions.

#### Radiological report normalization

Although paired textual information offers valuable insights for the synchronous learning of image understanding and representation, directly applying radiological reports to vision-language pretraining poses several challenges: (1) Radiological reports are often authored by different radiologists across various institutions. The absence of unified writing conventions can result in inconsistencies and limitations in standardization. (2) ARDS-related imaging characteristics primarily focus on the lung parenchyma. However, radiological reports frequently include descriptions of less relevant anatomical structures, such as the esophagus, sternum, and thoracic vertebrae. This irrelevant information may interfere with network representations, ultimately degrading model performance. (3) In comparison to discriminative tasks, such as classification, the regression fine-grained diagnosis tasks demand a higher level of representation of fine-grained and high-frequency information^35^. Such information, including disease severity, location, and texture, allows the visual encoder to establish precise relationships between CT data and specific ARDS determinations. However, coarse-grained categories or redundant textual descriptions introduce significant challenges in achieving precise alignment between textual content and visual features.

To address these challenges, we propose a radiological report normalization technique that extracts and refines the most significant fine-grained information from radiological reports. This approach leverages a locally deployed large language model, DeepSeek-70B^62^, to process the data efficiently and effectively. Our method requires both the patient metadata and the corresponding radiological report. To achieve this, we designed a standardized prompt specifically tailored for DeepSeek, enabling the conversion of raw, unstructured information into structured annotations. These annotations retain essential metadata (sex and age), while capturing detailed information relevant to ARDS, including anatomical location, distribution, texture, and severity. Simultaneously, the process eliminates redundant or irrelevant details about unrelated anatomical structures. The prompts were first iteratively refined through validation on a subset of approximately 500 cases. Once optimized, the finalized prompts were applied to the entire dataset, resulting in structured and detailed descriptions for all CT scans. This process ensures more precise and uniform data extraction. An example of the report normalization and DeepSeek prompt can be found in the Supplementary Table 2.

#### Pretraining strategy

The proposed pretraining pipeline adopts the contrastive language-image pre-training (CLIP)^20^ framework, which has been extensively utilized for natural image-text understanding and representation learning in a self-supervised manner. The central concept of CLIP is to align images with their corresponding textual descriptions by maximizing their similarity within a shared embedding space, while simultaneously minimizing the similarity between unrelated image-text pairs. This is achieved through the use of contrastive learning. In our experimental setup, let *I* denote a CT volume and *T* represent the radiological report associated with *I*. The image and text encoders are employed to map *I* and *T* into image and text embeddings ${e}_{I},{e}_{T}\in {{\mathbb{R}}}^{d}$, respectively.

The similarity between a CT volume and its corresponding radiological report is quantified using the cosine similarity between their embeddings, defined as follows:

6 $$s\left(I,T\right)=\frac{{{e}_{I}}^{{\rm{T}}}{e}_{T}}{\left|{e}_{I}\right|\left|{e}_{T}\right|}.$$

For a batch containing *N* CT-radiological report pairs ${\left\{({I}_{i},{T}_{i})\right\}}_{i=1}^{N}$, the CLIP framework employs contrastive objectives to promote high similarity scores for positive pairs (e.g., (*I*_*i*_, *T*_*i*_)) while penalizing high similarity scores for negative pairs (e.g., (*I*_*i*_, *T*_*j*_) where *i* ≠ *j*). The contrastive loss function is formulated using a symmetric cross-entropy loss applied to the similarity scores:

7 $${{\mathcal{L}}}_{{\rm{CLIP}}}=\frac{1}{2N}\left(\mathop{\sum }\limits_{i=1}^{N}-\log \frac{\exp (s({I}_{i},{T}_{i})/\tau )}{{\sum }_{j=1}^{N}\exp (s({I}_{i},{T}_{j})/\tau )}+\mathop{\sum }\limits_{i=1}^{N}-\log \frac{\exp (s({I}_{i},{T}_{i})/\tau )}{{\sum }_{j=1}^{N}\exp (s({I}_{j},{T}_{i})/\tau )}\right).$$

Here, *τ* represents a learnable temperature parameter that scales the logits, allowing the model to adapt the sharpness of the similarity distribution.

Despite its effectiveness, CLIP-based pretraining is fundamentally a discriminative learning approach. Previous studies have demonstrated that while CLIP excels at capturing coarse-grained information, it exhibits limited capacity for fine-grained feature representation^35,61^. This limitation poses a challenge for the comprehensive diagnosis and assessment of ARDS, which depends on precise examination of fine-grained features, including the location, texture, density, and morphology of lung parenchyma lesions. To enhance the model’s ability to represent these critical fine-grained features, we propose two novel technical strategies, text adversarial perturbation learning and joint learning with lesion identification.

#### Text adversarial perturbation learning

To address the limitations of standard CLIP-based pretraining in capturing fine-grained but diagnostically critical image-text associations, we propose a strategy designed to enhance the model’s sensitivity to detailed yet meaningful differences in radiological descriptions. Specifically, for each paired CT volume (*I*) and its corresponding radiological report (*T*), we generate an adversarial radiological report sample, denoted as $\widehat{T}$, which introduces minimal but targeted modifications to the original text. These modifications focus exclusively on the substantial details of lung parenchymal lesions, including disease name, location, distribution, severity, and textures, while keeping the rest of the report unchanged. By making controlled changes to one or two lesion-specific features, such as altering “ground-glass opacity” to “honeycombing” or adjusting the lesion’s location from “bilateral upper lobes” to “right lower lobe”, we simulate detailed variations that, despite their small scale, can represent entirely different disease conditions due to their impact on key diagnostic features. The adversarial samples are generated by DeepSeek-70B, and Supplementary Table 2 presents an example of the prompt and output. To ensure medical plausibility, we randomly sampled 500 adversarial reports for manual review by two radiologists, confirming that over 95% of the generated perturbations were clinically coherent and free of anatomical contradictions. Given this high reliability, large-scale training proceeded without exhaustive manual checking. For a batch of size *N*, where each CT volume is paired with its raw report *T* and *M* adversarial text samples ${({I}_{i},{\widehat{T}}_{i}^{k})}_{k=0}^{M}$ (with ${\widehat{T}}_{i}^{0}={T}_{i}$ for convenience), we design an objective function to encourage the model to effectively separate these samples within the embedding space. The adversarial contrastive loss is defined as:

8 $${{\mathcal{L}}}_{{\rm{adv}}}=\frac{1}{N}\left(\mathop{\sum }\limits_{i=1}^{N}-\log \frac{\exp (s({I}_{i},{\widehat{T}}_{i}^{0})/\tau )}{{\sum }_{k=1}^{M}\exp (s({I}_{i},{\widehat{T}}_{i}^{k})/\tau )}\right).$$

*M* is set to 2-3 in our experiment. Training the model with these adversarial samples improves its capacity to detect, interpret, and distinguish detailed variations in lesion descriptions. This targeted approach ensures that the model focuses on diagnostically significant ARDS-related details while reducing its reliance on global patterns or superficial associations.

#### Task-unified multimodal pretraining

To enhance the encoder’s ability to capture ARDS-specific features, we propose a multimodal pretraining framework that jointly incorporates lesion supervision and metadata prediction in addition to vision-language alignment. This design explicitly enforces the model to attend not only to parenchymal lesions but also to patient-level attributes, thereby disentangling fine-grained pathological characteristics from broader clinical context and improving task adaptability.

First, for lesion-aware representation, we introduce soft label distillation^63^. Given a CT volume *T*, its soft label *S* is obtained from the normalized difference map generated in the self-supervised segmentation stage, which preserves voxel-wise lesion probability rather than a binarized mask. The encoder embeddings *e*^*I*^ are decoded into a predicted segmentation model $\widehat{s}$, supervised with soft Dice loss:

9 $${{\mathcal{L}}}_{\mathrm{seg}}=1-\frac{2{\sum }_{i}{s}_{i}{\hat{s}}_{i}+\varepsilon }{{\sum }_{i}{s}_{i}^{2}+{\sum }_{i}{\hat{s}}_{i}^{2}+\varepsilon },$$

where *ε* = 10^−4^ prevents division by zero.

Second, for metadata-guided representation, we incorporate patient demographic prediction, including sex and age prediction, as an auxiliary task. Age regression is supervised with an L1 loss, while sex classification is optimized using binary cross-entropy (BCE) loss.

By integrating lesion quantification and metadata inference with adversarial vision-language alignment, AutoARDS learns multimodal embeddings that remain sensitive to detailed disease features while robust to population-level heterogeneity. The overall pretraining objective is thus defined as:

10 $${{\mathcal{L}}}_{{\rm{overall}}}={{\mathcal{L}}}_{{\rm{CLIP}}}+{{\mathcal{L}}}_{{\rm{adv}}}+{\lambda }_{1}{{\mathcal{L}}}_{{\rm{seg}}}+{\lambda }_{2}{{\mathcal{L}}}_{{\rm{sex}}}+{\lambda }_{3}{{\mathcal{L}}}_{{\rm{age}}},$$

where *λ*_1_, *λ*_2_, *λ*_3_ balance the contributions of lesion supervision and metadata prediction, set as (1, 0.1, 0.1) in our experiments.

#### Network architecture

We utilized the CXR-BERT^64^, a language transformer pre-trained specifically on chest X-ray reports, as our text encoder. During the pretraining phase, the encoder processes the normalized radiological reports and converts them into a consistent 512-token format. Each token is embedded in a 768-dimensional space. These token embeddings are aggregated by summing across all 512 tokens, followed by a linear transformation^23^. This step compresses the information into a 512-dimensional representation. The parameters of the text encoder remain fixed throughout the pretraining phase.

We employed the Swin Transformer^65^, a hierarchical network architecture, as the image encoder. Prior studies have demonstrated its superior capability in capturing fine-grained information compared to pure Vision Transformer (ViT) architectures^66^. To preserve the complete receptive field and enable long-range attention, the entire CT volume was utilized as input without cropping. The model was configured with a patch size of 4, a feature dimension of 24, a window size of 7, and layer depths of (2, 2, 2, 2), with the number of attention heads set to (3, 6, 12, 24).

### Fine-tuning training for ARDS tasks

To apply our pretrained encoder to specific ARDS tasks, including P/F ratio regression, diagnosis, and prognosis prediction, we design a lightweight 3D convolutional network as the decoder. The embeddings from the encoder are tokenized and used as inputs to this decoder, which consists of two main components: a convolutional block and a task-specific header. The convolutional block employs 3D convolutions for feature extraction, with InstanceNorm for normalization and GELU as the activation function. The features extracted by the convolutional block are then passed to the header, which flattens them and processes them through two fully connected layers. During fine-tuning, the encoder parameters remain fixed, while only the decoder parameters are updated through optimization. Data augmentation strategies, including random zooming, flipping, and adding Gaussian noise, are utilized.

For each task, the following objective functions were employed: ARDS and ARDS diagnosis-binary cross entropy loss; P/F ratio regression-L1 Loss; RVD prediction-binary cross entropy loss; and survival prediction-NLLLogisticHazardLoss. For all internal datasets, we conducted 5-fold cross-validation, with the training, validation, and testing sets divided in a 7:1:2 ratio. The final results are reported across all datasets. For external datasets, we report the averaged performance obtained from five independently trained models.

For all task-specific applications, image embeddings were first obtained using the image encoder, followed by lesion segmentation with the segmentation model. From the segmented lesions, eight quantitative metrics were extracted (see subsection “Lesion quantification enables ARDS differentiation and tracing”) as representative descriptors of lesion burden. These eight lesion-derived metrics and patient metadata were first projected into a compact representation via a linear transformation and spatially expanded to match the encoder feature dimensions. This representation was then concatenated with the main image embedding along the channel axis. Together with the residual embeddings from multiple encoder stages, the fused features were processed by corresponding decoder blocks to produce the final predictions.

### Reader study design

The reader study included seven readers: three junior ICU physicians with 4–5 years of clinical experience in critical care and four trainees in the early stage of ICU training. All readers independently reviewed the same set of 25 chest CT cases. To ensure a standardized imaging-only evaluation setting, arterial blood gas results and prior imaging were not provided during assessment. The test cases were randomly sampled from the full dataset to preserve a realistic case composition and better reflect routine clinical scenarios.

### Analysis for healthy CT synthesis and ablation study

To assess the fidelity of the synthesized healthy CT images, we first evaluated their distributional similarity to real CT scans using the Kernel Inception Distance (KID)^67^, where lower values indicate better alignment between synthesized and real image distributions. As shown in Supplementary Fig. 4a, the KID score consistently decreased as training progressed, dropping from approximately 0.07 at 50,000 iterations to below 0.05 after 300,000 iterations. This steady decline demonstrates that the synthesized images progressively converged toward the distribution of real healthy CTs, with the final model achieving high-fidelity generation.

Beyond quantitative assessment, we further examined perceptual realism through a reader study involving 20 medical trainees. Each participant was asked to discriminate between real and synthesized CT images. The ROC analysis in Supplementary Fig. 4b shows that most participants performed close to the diagonal reference line of random guessing, with true positive rates fluctuating around 0.5 at moderate false positive rates. This indicates that, despite medical training, the synthesized images were visually indistinguishable from authentic CTs for most observers. Together, these findings confirm that the proposed synthesis approach achieves both distributional fidelity and perceptual plausibility, producing images that are quantitatively aligned with real data and qualitatively convincing to human readers.

### Ablation study

To investigate the contribution of individual components in AutoARDS, we conducted an ablation study on the internal dataset (Supplementary Fig. 4c). Removing any of the four modules, including soft label distillation (SL), metadata prediction (MP), adversarial pretraining (AP), and lesion segmentation/quantification (LS), led to performance degradation across ARF, ARDS, and RVD diagnosis, prognosis prediction, and P/F ratio prediction tasks.

In particular, eliminating both SL and LS substantially reduced the ARDS diagnostic AUC from 0.8874 to 0.8065 and the P/F ratio prediction PCC from 0.8360 to 0.7291. This pronounced decline can be attributed to the complete loss of lesion information and attention guidance, which are critical for ARDS-related tasks that heavily rely on lesion characteristics. By contrast, removing either SL or LS alone resulted in more modest degradation, reflecting their complementary contributions in capturing lesion-specific features. The removal of AP also led to considerable performance deterioration, highlighting its central role in AutoARDS. AP encourages the model to emphasize ARDS-related features, whereas removing it effectively can reduce AutoARDS to a conventional CLIP-based pretraining model. As discussed previously, such coarse-grained pretraining struggles to capture the fine-grained diagnostic signals required for ARDS-related tasks. Collectively, these results underscore the complementary and indispensable roles of SL, LS, and AP, confirming that the integration of lesion-focused representation learning and adversarial pretraining is essential for achieving robust performance in AutoARDS.

### Experimental settings

All proposed methods are implemented using Python 3.10 and PyTorch 2.7.0. The Adam optimizer is employed for optimization, with an initial learning rate of 1 × 10^−4^. The decay rates for the first-order and second-order momentum gradients are set to 0.9 and 0.999, respectively. All experiments are conducted on a Linux workstation equipped with two NVIDIA RTX A100 GPUs. We employed image augmentation including random zooming, Gaussian noise (*σ* = 0.1), random axis flipping, and random rotation, which were all implemented with MONAI^68^. The model pretraining process requires approximately 15 days with 20 epochs, while fine-tuning for each downstream task takes roughly 5 days with 50 epochs.

Segmentation results are reported as mean ± std, while classification results are presented as mean ± 95%CI. The Wilcoxon signed-rank test is used to evaluate the distributions of two paired groups, whereas the Mann-Whitney U test is applied for unpaired groups. For the comparison of ROC curves and corresponding AUC values, the DeLong test was additionally applied, which can provide a non-parametric approach specifically designed for correlated ROC curves. A *p*-value of less than 0.05 (*p* < 0.05) is considered statistically significant in this study. The *p*-values are indicated in figures and tables using the following notation: *p < 0.05, **p < 0.01, ***p < 0.001, ****p < 0.0001, and NS (not significant). All statistical analyses are conducted using Python and R (version 4.3.0).

In our project, two large language models, DeepSeek-70B and GPT-4o, were used exclusively for radiological report normalization and adversarial text perturbation. Specifically, DeepSeek-70B refers to the DeepSeek-R1-Distill-Llama-70B model, which was locally deployed on our institutional server. The model was used in accordance with the official usage guidelines provided by the developers (https://huggingface.co/deepseek-ai/DeepSeek-R1-Distill-Llama-70B). In addition, GPT-4o (OpenAI) was accessed via the official OpenAI cloud-based API. All interactions with GPT-4o were conducted between January and August 2025, using the then-current production version of the gpt-4o model.

### Ethics Approval

This multicenter retrospective study included chest CT data from six sources. Ethical approval was obtained from the local institutional review boards of the participating institutions. Specifically, approval was granted by the Ethics Committee of the First Affiliated Hospital of Harbin Medical University (approval no. 2025196), the Ethics Committee of the Fourth Affiliated Hospital of Harbin Medical University, the Ethics Committee of Mudanjiang First People’s Hospital, the IEC for Clinical Research of Zhongda Hospital, Southeast University (approval no. 2025ZDSYLL524-P01), and the Ethics Committee of Hainan General Hospital (approval no. EC-LC-2025-51-01). For these centers, the requirement for informed consent was waived due to the retrospective nature of the study and the use of anonymized data. Data from OMIX, China National Center for Bioinformation / Beijing Institute of Genomics, Chinese Academy of Sciences (accession no. OMIX006496) were obtained from a publicly available database and were exempt from further ethical review. Pre-experiments were conducted under the oversight of the Institutional Biosafety and Bioethics Committee at King Abdullah University of Science and Technology (approval no. 25IBEC021). All data used in this study were de-identified prior to analysis.

## Supplementary information

Supplementary Information

Supplementary Data 1

Supplementary Data 2

Supplementary Data 3

## Data Availability

The raw data and echocardiography reports supporting the findings of this study are provided as Supplementary Data 1 and Supplementary Data 2. The dataset from Center 3^49^ and CT-CLIP^23^ can be accessed according to the instructions released in the referenced manuscript. Additional datasets generated and/or analyzed during the current study are not publicly available due to ethical and legal restrictions associated with patient privacy and institutional data-sharing agreements, but are available from the corresponding authors upon reasonable request, subject to scientific review, external Institutional Review Board (IRB) approval, and a data use agreement.

## Code availability

An open-source version of the code base is available on GitHub at https://github.com/FAHHMU-Meng/AutoARDS.
