# Supplementary material for: CT-based AI system for quantitative and integrated management of acute respiratory distress syndrome in critical care
Source: NPJ Digit Med. 2026 Apr 24;9:493. doi: 10.1038/s41746-026-02648-9 (PMC13315241; doi:10.1038/s41746-026-02648-9)
Supplement: Supplementary file 1 — Supplementary Information [file 41746_2026_2648_MOESM1_ESM.pdf]

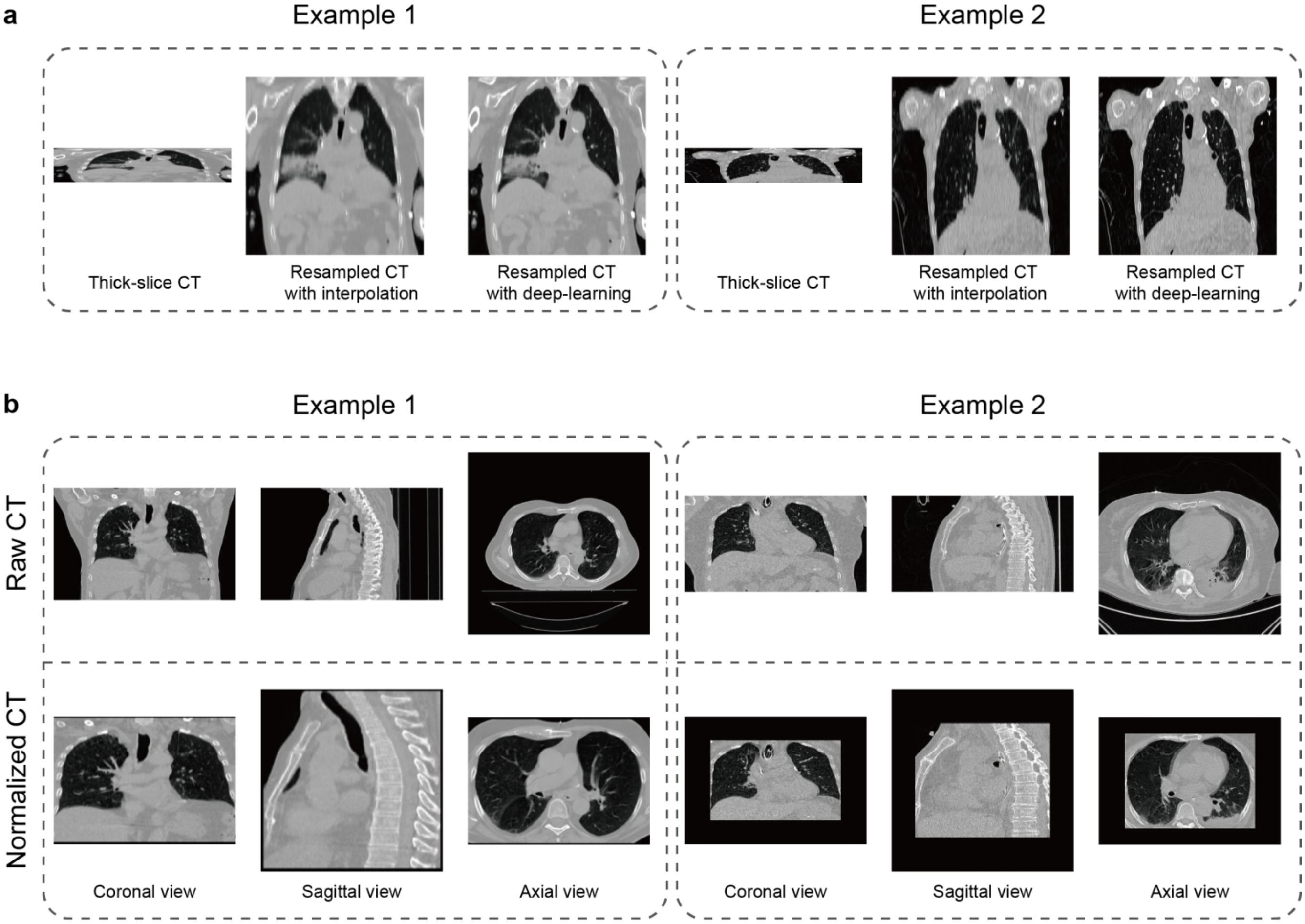

Supplementary Figure 1. Illustration for data pre-processing. (a) Thick-slice super-resolution. (b) Data normalization to guide the model to focus more on the lung area.

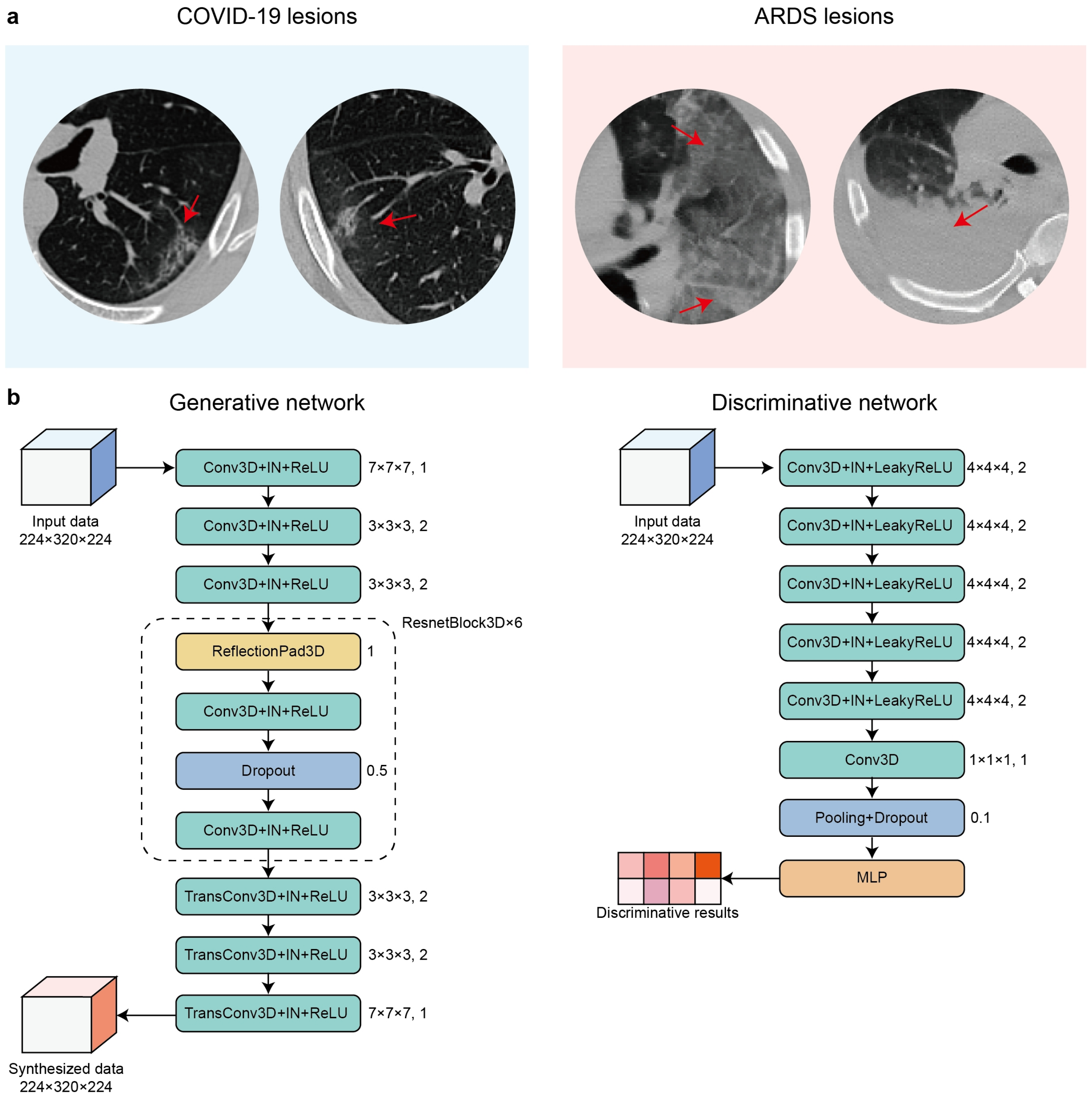

Supplementary Figure 2. Self-supervised ARDS lesion segmentation. (a) Visualization comparison between lesions of COVID-19 and ARDS. The former presents peripheral ground-glass opacities, while ARDS presents diffuse lesions and high-density consolidations. (b) Network architectures of the generation and discrimination model in the CycleGAN.

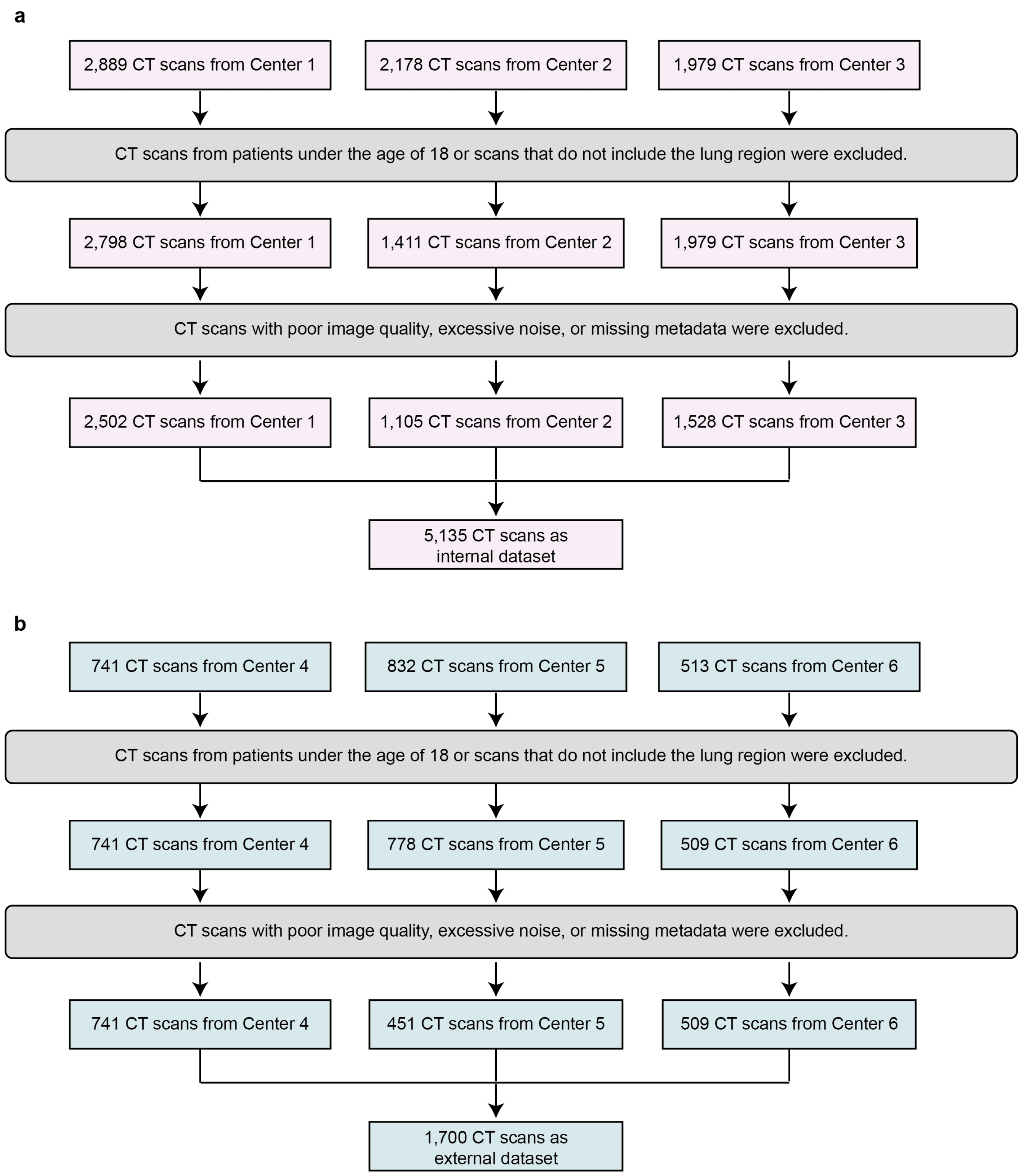

Supplementary Figure 3. The flowchart of the internal (a) and external (b) dataset setup.

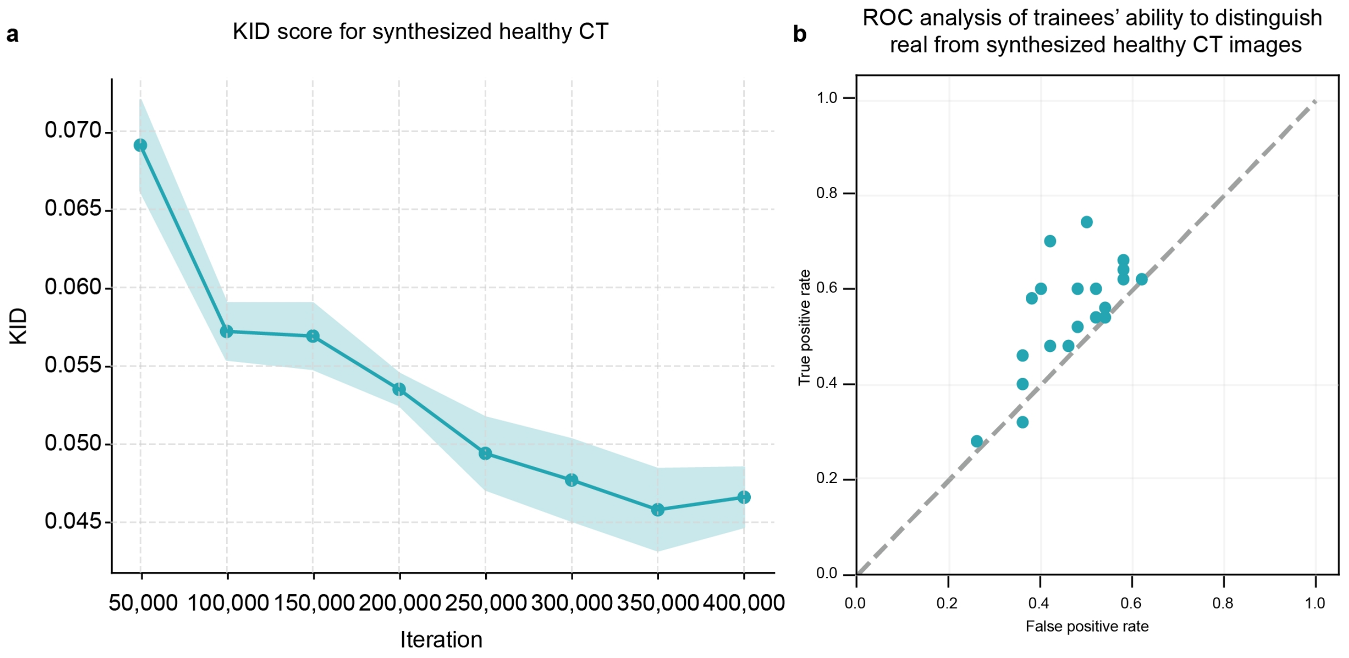

c

Ablation study for AutoARDS

	ARF diagnosis	ARDS diagnosis	RVD diagnosis	Prognosis prediction	P/F ratio prediction
Methods	AUC	AUC	AUC	AUC	PCC
AutoARDS	0.9748	0.8874	0.7640	0.7872	0.8360
w/o SL	0.9432	0.8509	0.7627	0.7635	0.8021
w/o MP	0.9552	0.8662	0.7441	0.7844	0.8182
w/o AP	0.8145	0.7597	0.7033	0.6919	0.6729
w/o LS	0.9390	0.8455	0.7409	0.7548	0.7687
w/o SL+LS	0.9307	0.8065	0.7433	0.7116	0.7291

Supplementary Figure 4. Analysis for healthy CT synthesis and ablation study. (a) Kernel Inception Distance (KID) score of synthesized healthy CT images across different training iterations. The shaded area denotes the 95% confidence interval. (b) Receiver operating characteristic (ROC) analysis of a reader study, in which 20 medical trainees were asked to distinguish between real and synthesized healthy CT images. The scattered points represent individual reader performance. (c) Ablation study of AutoARDS conducted on the internal dataset. Removal of individual modules—including soft label distillation (SL), metadata prediction (MP), adversarial pretraining (AP), and lesion segmentation/quantification (LS)—consistently degraded performance across multiple tasks (ARF, ARDS, and RVD diagnosis, prognosis prediction, and P/F ratio prediction).

a

		Ground truth	Baseline	3D-MAE	CT-CLIP	AutoARDS
Internel dataset	R^2	-	0.588[0.557, 0.618]	0.611[0.583, 0.640]	0.054[0.015, 0.110]	0.694[0.653, 0.731]
	MAPE%	-	28.792[27.494, 30.156]	24.983[23.831, 26.117]	50.222[47.513, 53.039]	20.221[19.341, 21.109]
	PCC	-	0.767[0.746, 0.786]	0.782[0.764, 0.800]	0.233[0.121, 0.332]	0.833[0.808, 0.855]
	SRCC	-	0.770[0.750, 0.789]	0.795[0.777, 0.813]	0.301[0.260, 0.340]	0.848[0.832, 0.862]
	Mean±STD (mmHg)	229.8±118.7	234.6±90.0	228.8±93.2	226.4±110.8	217.5±91.8
	Bias (mmHg)	-	4.8	-10.0	-3.4	-12.3
Externel dataset	R^2	-	0.439[0.378, 0.503]	0.529[0.466, 0.590]	0.137[0.102, 0.178]	0.676[0.606, 0.737]
	MAPE%	-	27.508[25.306, 29.932]	25.313[23.317, 27.613]	37.173[34.596, 39.990]	19.569[17.215, 22.426]
	PCC	-	0.663[0.615, 0.709]	0.727[0.683, 0.768]	0.370[0.319, 0.422]	0.822[0.778, 0.858]
	SRCC	-	0.717[0.678, 0.752]	0.759[0.724, 0.790]	0.437[0.383, 0.488]	0.839[0.810, 0.864]
	Mean±STD (mmHg)	243.0±106.4	221.0±90.0	215.6±90.0	215.1±88.0	223.9±91.0
	Bias (mmHg)	-	-22.0	-27.4	-27.9	-19.1

b

		Baseline	3D-MAE	CT-CLIP	AutoARDS
Internel dataset	Accuracy	0.680;0.708;0.910	0.713;0.767;0.942	0.568;0.602;0.859	0.756;0.767;0.926
	Sensitivity	0.572;0.634;0.072	0.542;0.713;0.444	0.605;0.312;0.217	0.688;0.737;0.411
	Specificity	0.726;0.757;0.991	0.785;0.802;0.989	0.552;0.795;0.921	0.784;0.786;0.976
	Precision	0.465;0.634;0.429	0.513;0.705;0.800	0.361;0.503;0.208	0.571;0.696;0.620
	F1-score	0.513;0.634;0.124	0.527;0.709;0.571	0.452;0.385;0.213	0.624;0.716;0.494
Externel dataset	Accuracy	0.651;0.702;0.925	0.657;0.715;0.925	0.540;0.647;0.913	0.744;0.815;0.951
	Sensitivity	0.495;0.654;0.278	0.449;0.713;0.417	0.495;0.503;0.194	0.644;0.839;0.361
	Specificity	0.745;0.727;0.953	0.783;0.716;0.947	0.568;0.720;0.945	0.806;0.802;0.977
	Precision	0.542;0.547;0.208	0.558;0.559;0.259	0.411;0.475;0.135	0.669;0.682;0.406
	F1-score	0.518;0.596;0.238	0.497;0.627;0.319	0.449;0.489;0.159	0.656;0.752;0.382

Supplementary Table 1. Performance of P/F ratio estimation and P/F-derived ARDS severity stratification on the internal and external datasets. (a) Regression performance for CT-based P/F ratio estimation, including R^2, MAPE, PCC, SRCC, Mean±STD of measured and predicted values, and prediction bias. For R^2, MAPE, PCC, and SRCC, the corresponding 95% confidence intervals are additionally reported. (b) Class-level performance of ARDS severity stratification derived from the predicted P/F ratio according to the Berlin definition, including accuracy, sensitivity, specificity, precision, and F1-score for the mild, moderate, and severe categories.

**Meta information:**	PatientAge: 49 years; PatientSex: M; Spatial resolution: [0.837, 0.837, 5.0]; Manufacturer: Philips; KVP: 120, XrayTubeCurrent: 266
**Radiological report (findings):**	The diameters and courses of the main mediastinal vascular structures are normal, with no aneurysmal dilatation or localized stenosis observed. The proportions of the heart chambers are harmonious, myocardial thickness is within normal limits, the pericardium is smooth and intact, and no signs of effusion were detected. The mid-section of the trachea and the lumens of both main bronchi are unobstructed, with smooth mucosal surfaces. Diffuse patchy ground-glass opacities and consolidation shadows are distributed across both lung fields, more prominent in the middle and lower lobes of the right lung. Air bronchograms are visible within the consolidation areas. The lesions exhibit a multi-lobar and multi-segmental distribution pattern. Linear fibrotic proliferative lesions are observed in the apical segment of the right upper lobe and the lingular segment of the left lung. No definite solitary spherical lesions or cavitary lesions were detected. No fluid-density shadows were observed in either pleural cavity. Mild localized adhesion of the pleura was noted along the right oblique fissure, with a pleural thickness of less than 3 mm. The cortex of the sternum, ribs, and thoracic vertebrae is continuous, with uniform bone marrow density, and no osteolytic or osteoblastic destructive lesions were observed. Upper abdominal sections show a well-defined liver contour with uniform parenchymal density, and the diameter of the main portal vein is 12 mm.
**DeepSeek prompt 1:**	Please standardize the provided radiology report and patient metadata into a structured clinical narrative. Format the patient's age as "[X] years-old" and gender as "[male/female/other]." Retain exclusively pulmonary parenchymal and cardiac functional findings, omitting descriptions of other anatomical regions. Preserve lung abnormalities, such as nodules, infiltrates, consolidation, and fibrosis, using standardized terms. For each abnormality, describe its anatomical location (e.g., right upper lobe), distribution (focal/difuse/segmental), texture (e.g., ground-glass, solid), and severity (mild/moderate/severe). Do not include any information about the inference or diagnosis of ARDS. Present observations in one continuous paragraph without item icons, preserving ambiguous phrasing from the original report (e.g., "possible," "cannot exclude"). Align terminology with the RadLex lexicon where applicable, retain original measurement units (e.g., mm, cm), and avoid speculative interpretations beyond explicitly documented data.
**DeepSeek response 1:**	The patient is a 49-year-old male. The proportions of the heart chambers are harmonious, myocardial thickness is within normal limits, and the pericardium is smooth and intact, with no signs of effusion detected. The lung parenchyma demonstrates diffuse patchy ground-glass opacities and consolidation shadows distributed bilaterally, predominantly in the middle and lower lobes of the right lung. Air bronchograms are visible within the areas of consolidation. The lesions exhibit a multi-lobar and multi-segmental distribution pattern. Linear fibrotic proliferative changes are noted in the apical segment of the right upper lobe and the lingular segment of the left lung. No solitary spherical lesions, cavitary lesions, or pleural effusions are identified. Mild localized pleural adhesion is observed along the right oblique fissure, with pleural thickness measuring less than 3 mm.
**DeepSeek prompt 2:**	You are now tasked with modifying this radiological report. Specifically: 1) Focus on the lung parenchymal lesion details, including disease name, location, distribution, severity, and textures, while leaving all other parts of the report unchanged; 2) Make minimal changes by randomly selecting two or three features related to the lung parenchymal lesions and altering their context. Avoid adding new information or overly changing the style or structure; 3) Ensure the modifications maintain medical plausibility and logical coherence. Changes should not conflict with the rest of the report.
**DeepSeek response 2:**	The patient is a 49-year-old male. The proportions of the heart chambers are harmonious, myocardial thickness is within normal limits, and the pericardium is smooth and intact, with no signs of effusion detected. The lung parenchyma demonstrates diffuse patchy ground-glass opacities and consolidation shadows distributed bilaterally, **with a slight predominance in the lower lobes bilaterally**. Air bronchograms are visible within the areas of consolidation. The lesions exhibit a **patchy and asymmetrical** distribution pattern. Linear fibrotic proliferative changes are noted in the apical segment of the right upper lobe and the lingular segment of the left lung. No solitary spherical lesions, cavitary lesions, or pleural effusions are identified. Mild localized pleural adhesion is observed along the right oblique fissure, with pleural thickness measuring less than 3 mm.

Supplementary Table 2. An example of structuring and standardizing radiological reports.
