# Supplementary figures and images for: CT-based AI system for quantitative and integrated management of acute respiratory distress syndrome in critical care (part 2 of 2)
Source: NPJ Digit Med. 2026 Apr 24;9:493. doi: 10.1038/s41746-026-02648-9 (PMC13315241; doi:10.1038/s41746-026-02648-9)

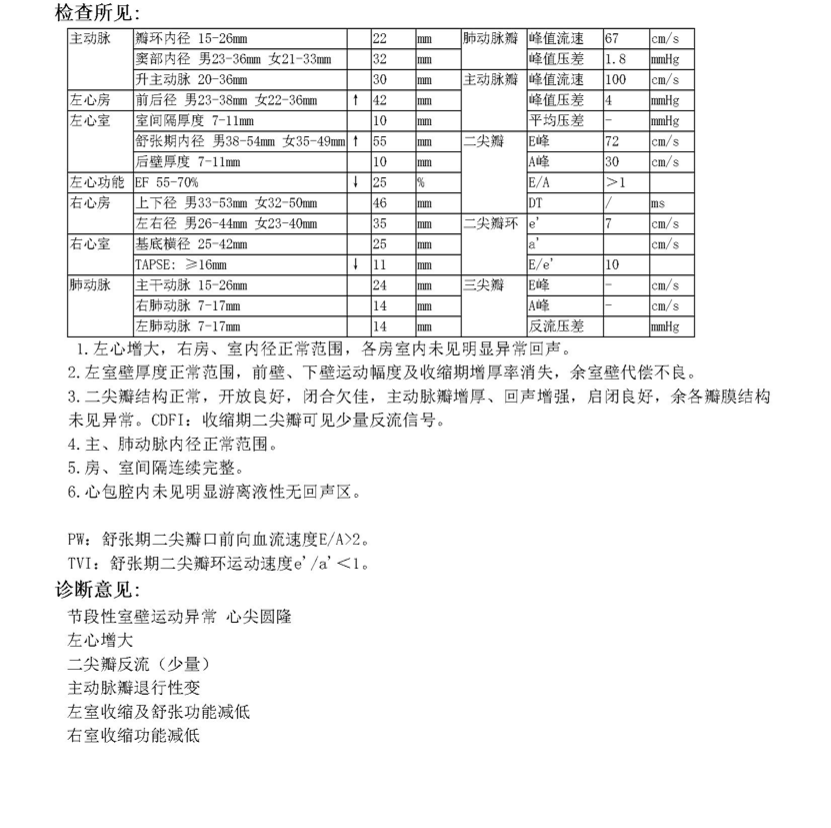

Supplement: Supplementary file 3 — Supplementary Data 2 [file 41746_2026_2648_MOESM3_ESM.zip › echocardiography_reports/220.png]

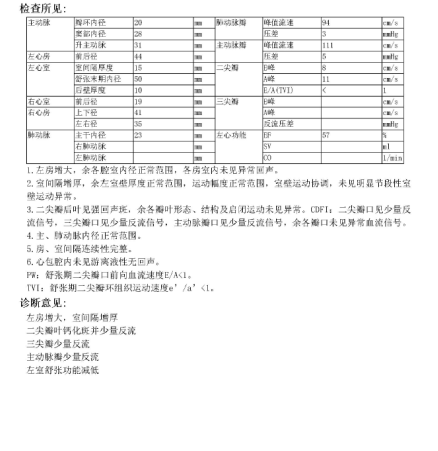

Supplement: Supplementary file 3 — Supplementary Data 2 [file 41746_2026_2648_MOESM3_ESM.zip › echocardiography_reports/221.png]

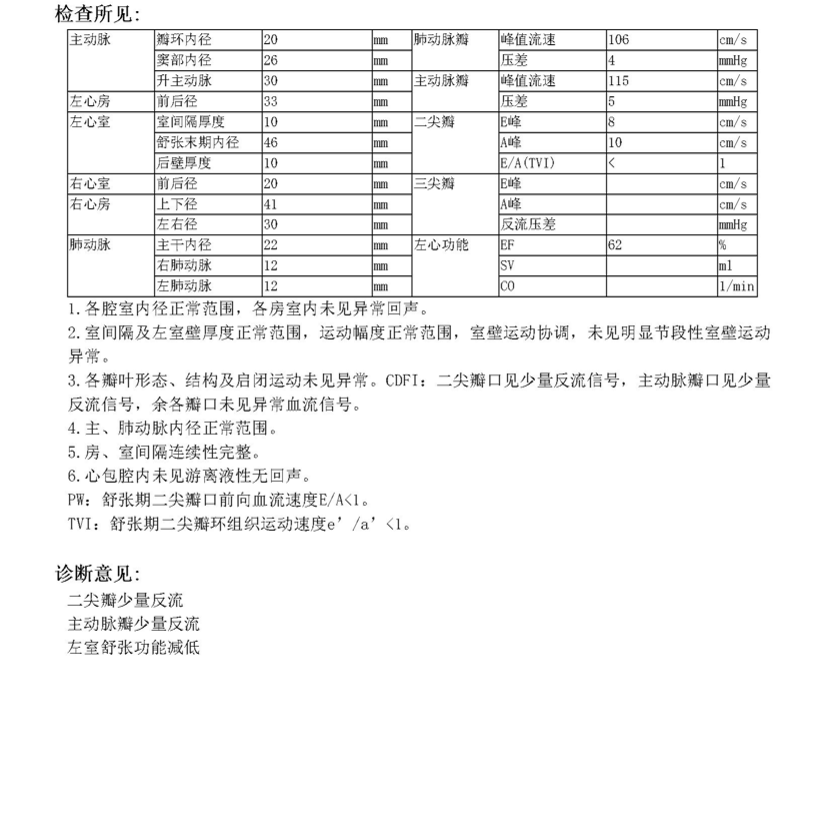

Supplement: Supplementary file 3 — Supplementary Data 2 [file 41746_2026_2648_MOESM3_ESM.zip › echocardiography_reports/222.png]

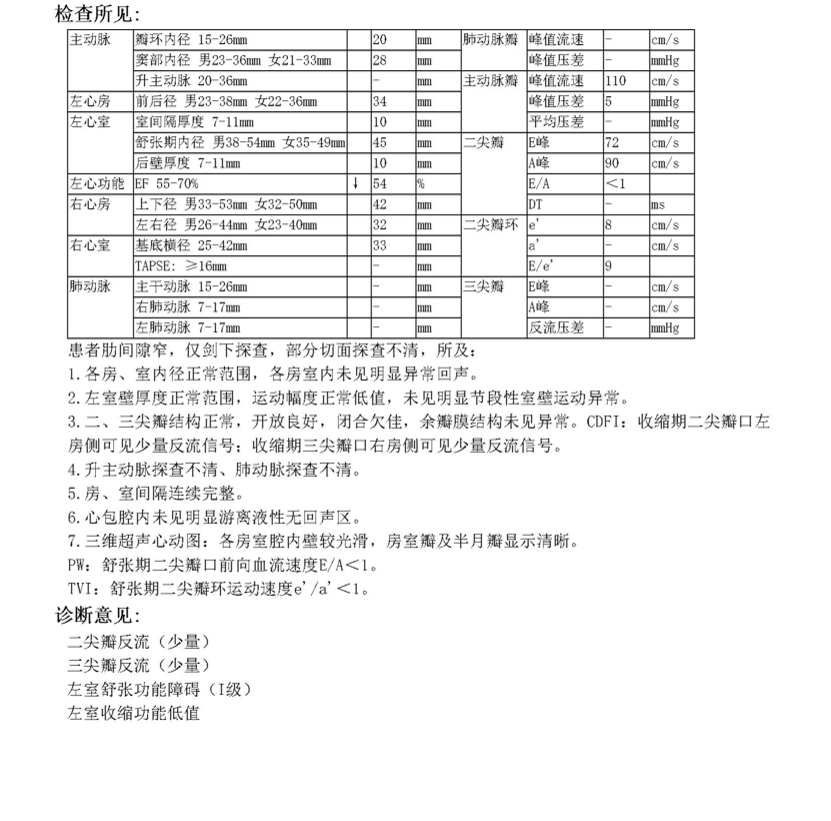

Supplement: Supplementary file 3 — Supplementary Data 2 [file 41746_2026_2648_MOESM3_ESM.zip › echocardiography_reports/223.png]

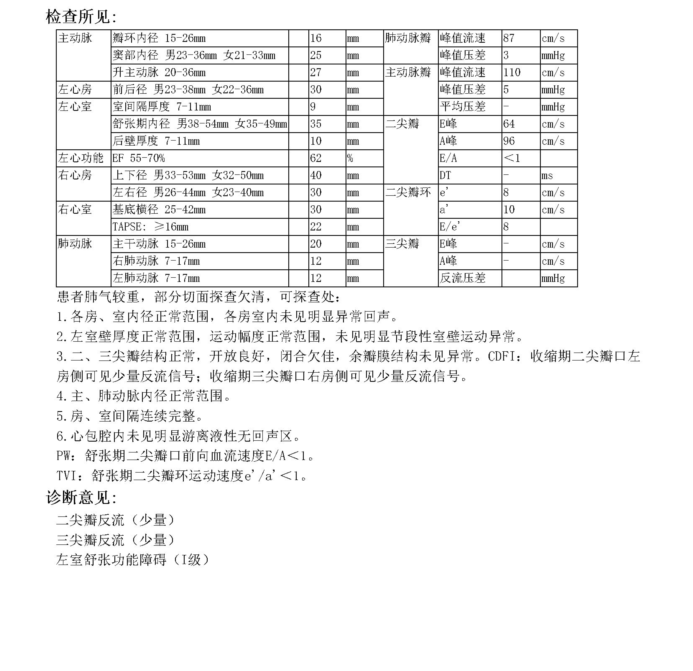

Supplement: Supplementary file 3 — Supplementary Data 2 [file 41746_2026_2648_MOESM3_ESM.zip › echocardiography_reports/224.png]

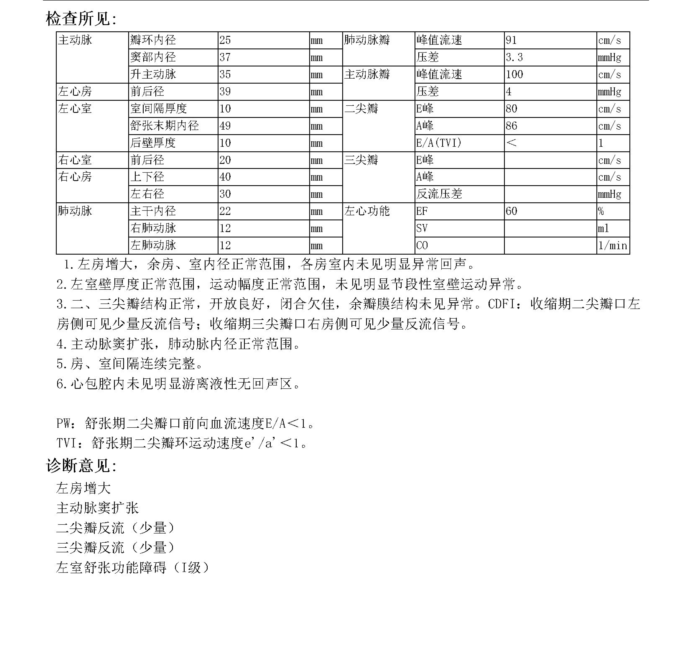

Supplement: Supplementary file 3 — Supplementary Data 2 [file 41746_2026_2648_MOESM3_ESM.zip › echocardiography_reports/225.png]

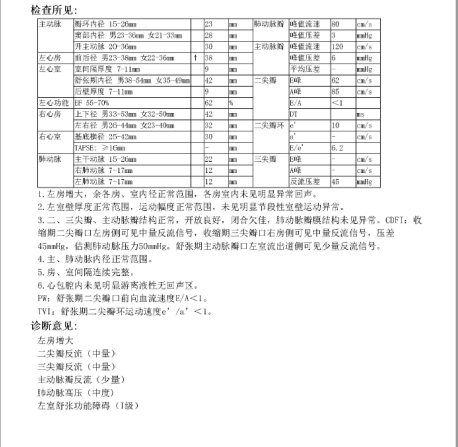

Supplement: Supplementary file 3 — Supplementary Data 2 [file 41746_2026_2648_MOESM3_ESM.zip › echocardiography_reports/226.png]

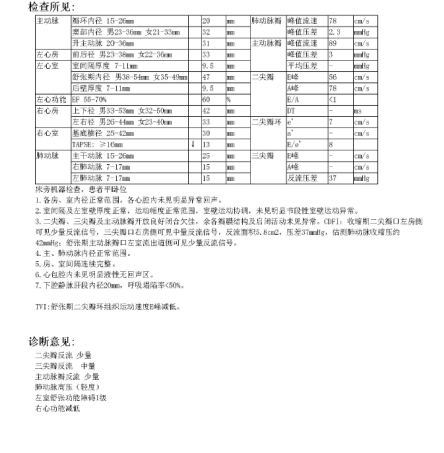

Supplement: Supplementary file 3 — Supplementary Data 2 [file 41746_2026_2648_MOESM3_ESM.zip › echocardiography_reports/227.png]

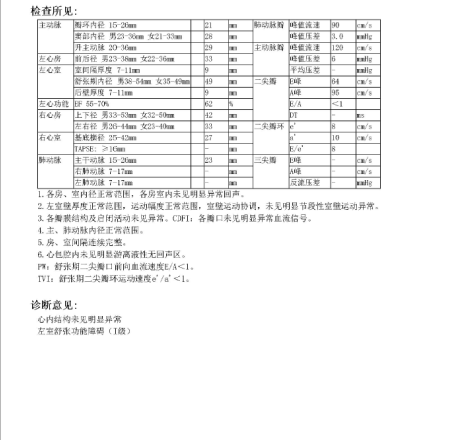

Supplement: Supplementary file 3 — Supplementary Data 2 [file 41746_2026_2648_MOESM3_ESM.zip › echocardiography_reports/228.png]

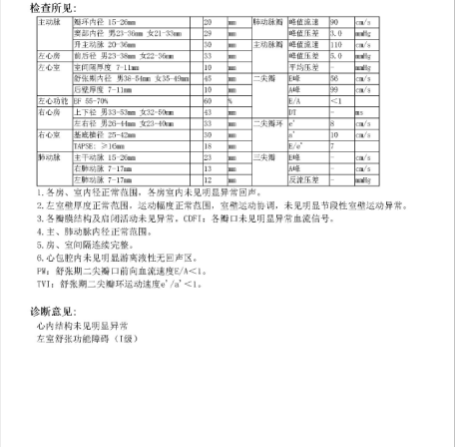

Supplement: Supplementary file 3 — Supplementary Data 2 [file 41746_2026_2648_MOESM3_ESM.zip › echocardiography_reports/229.png]

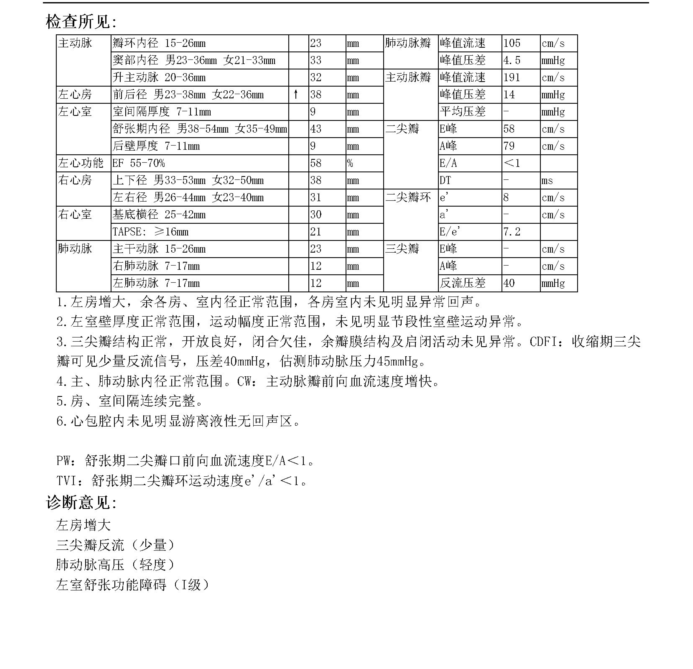

Supplement: Supplementary file 3 — Supplementary Data 2 [file 41746_2026_2648_MOESM3_ESM.zip › echocardiography_reports/230.png]

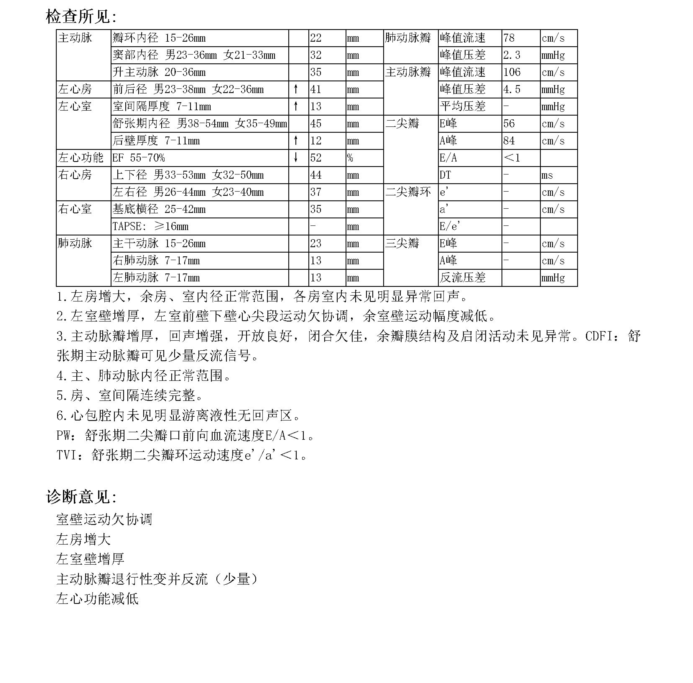

Supplement: Supplementary file 3 — Supplementary Data 2 [file 41746_2026_2648_MOESM3_ESM.zip › echocardiography_reports/231.png]

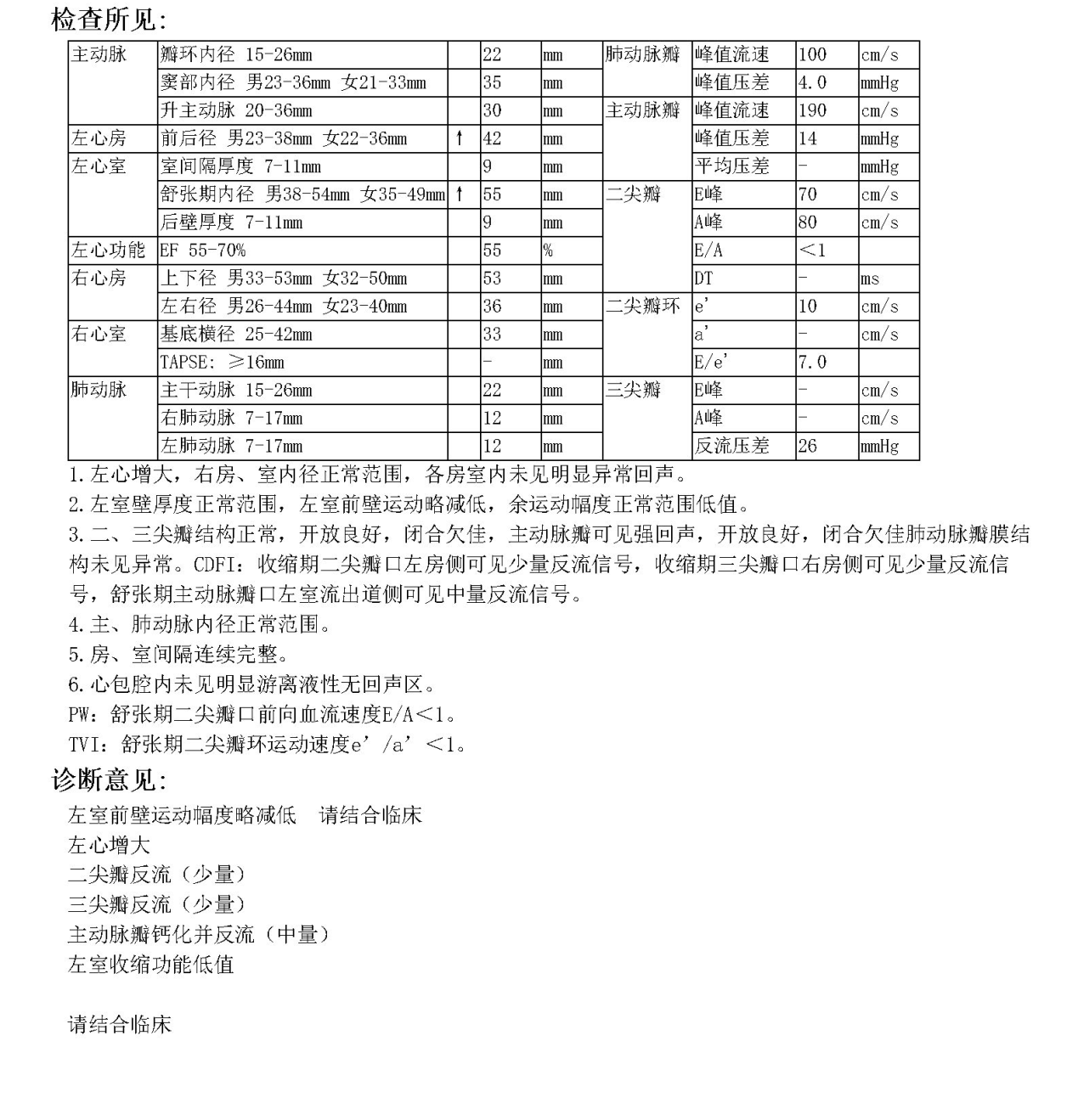

Supplement: Supplementary file 3 — Supplementary Data 2 [file 41746_2026_2648_MOESM3_ESM.zip › echocardiography_reports/232.png]

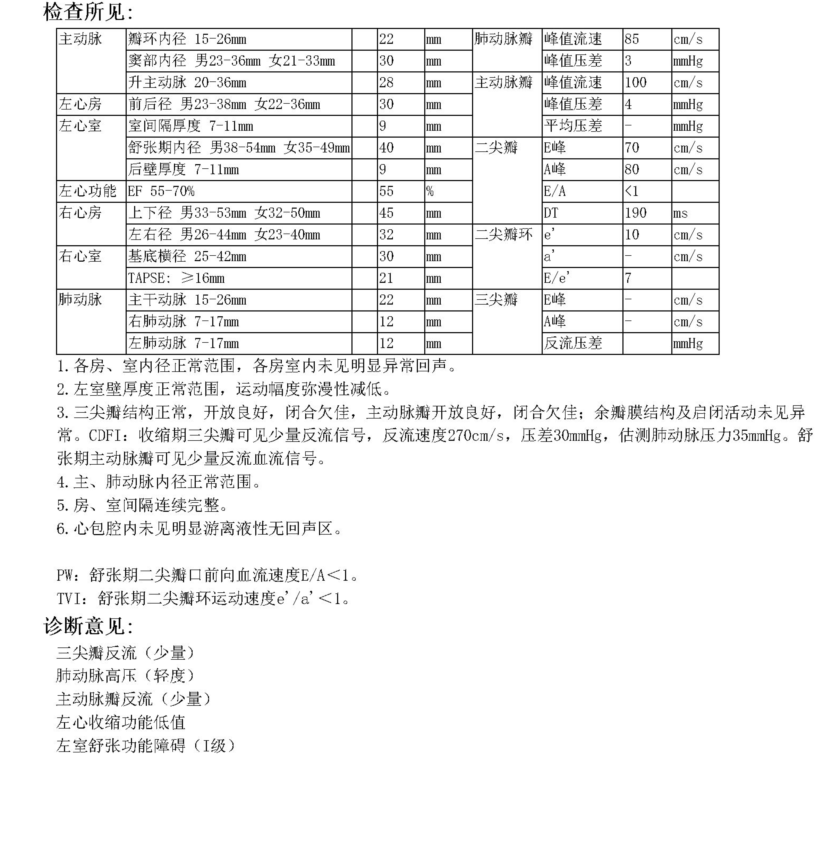

Supplement: Supplementary file 3 — Supplementary Data 2 [file 41746_2026_2648_MOESM3_ESM.zip › echocardiography_reports/233.png]

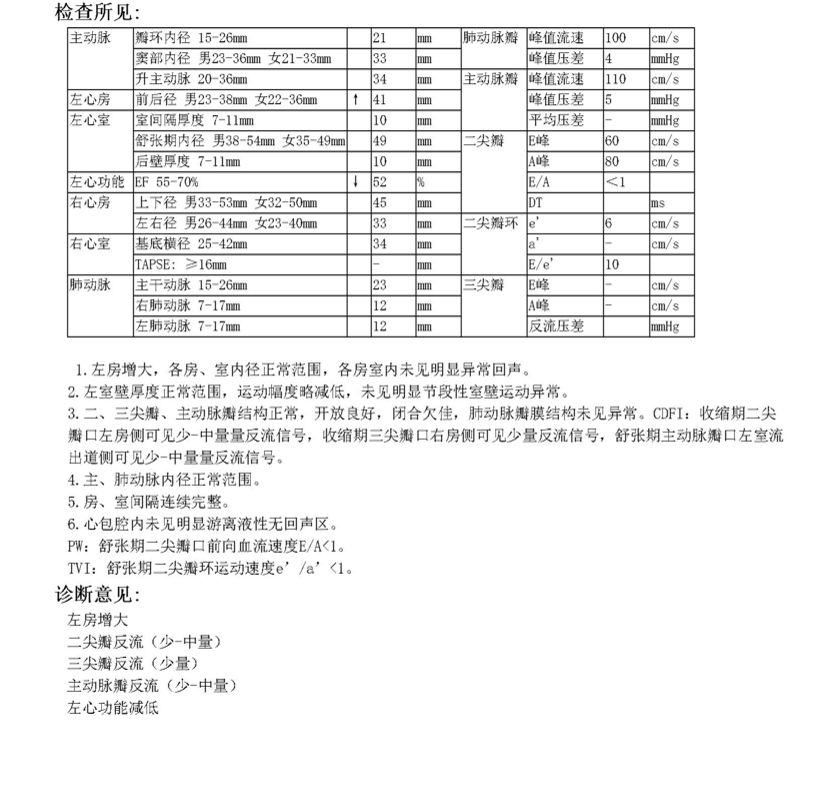

Supplement: Supplementary file 3 — Supplementary Data 2 [file 41746_2026_2648_MOESM3_ESM.zip › echocardiography_reports/234.png]

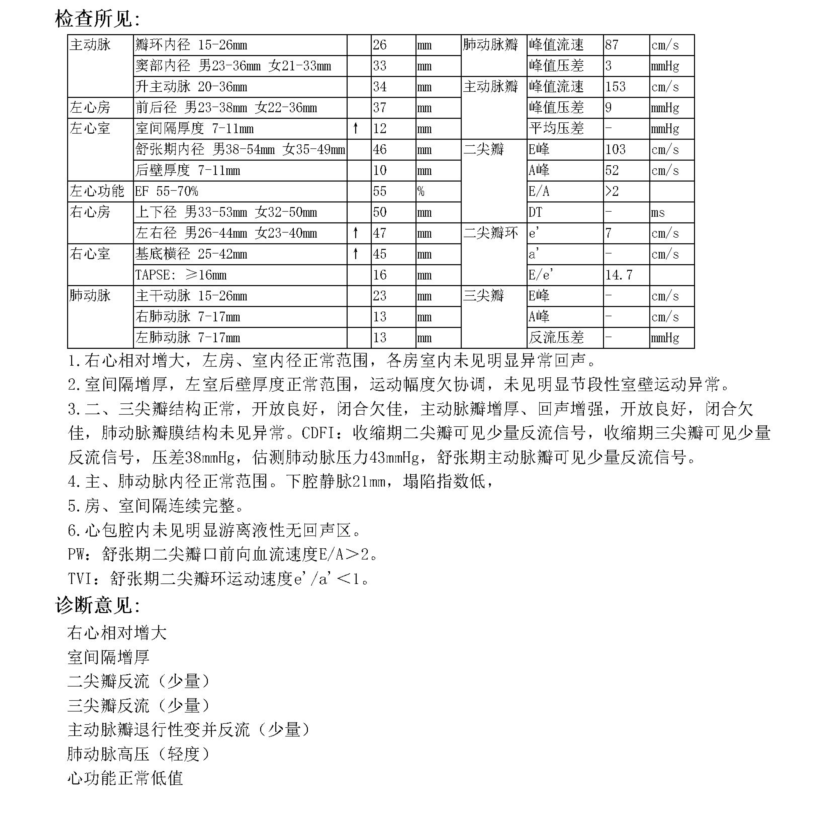

Supplement: Supplementary file 3 — Supplementary Data 2 [file 41746_2026_2648_MOESM3_ESM.zip › echocardiography_reports/235.png]

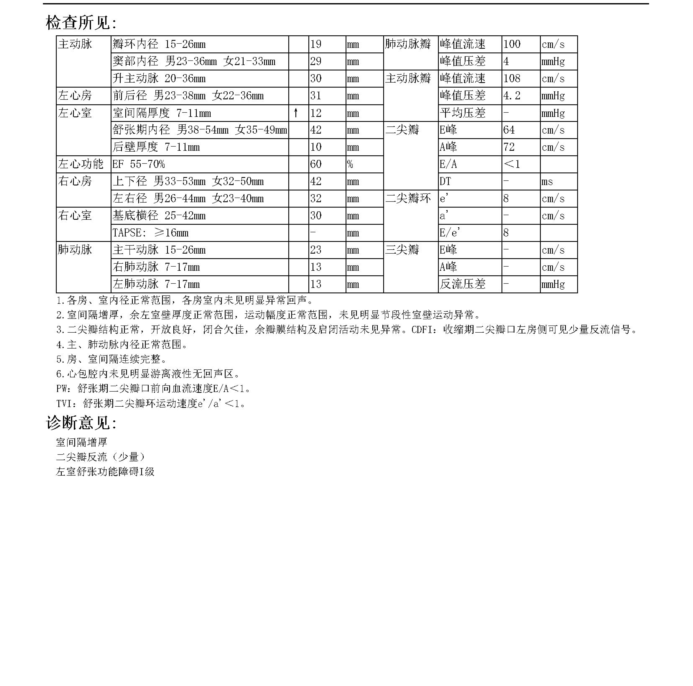

Supplement: Supplementary file 3 — Supplementary Data 2 [file 41746_2026_2648_MOESM3_ESM.zip › echocardiography_reports/236.png]

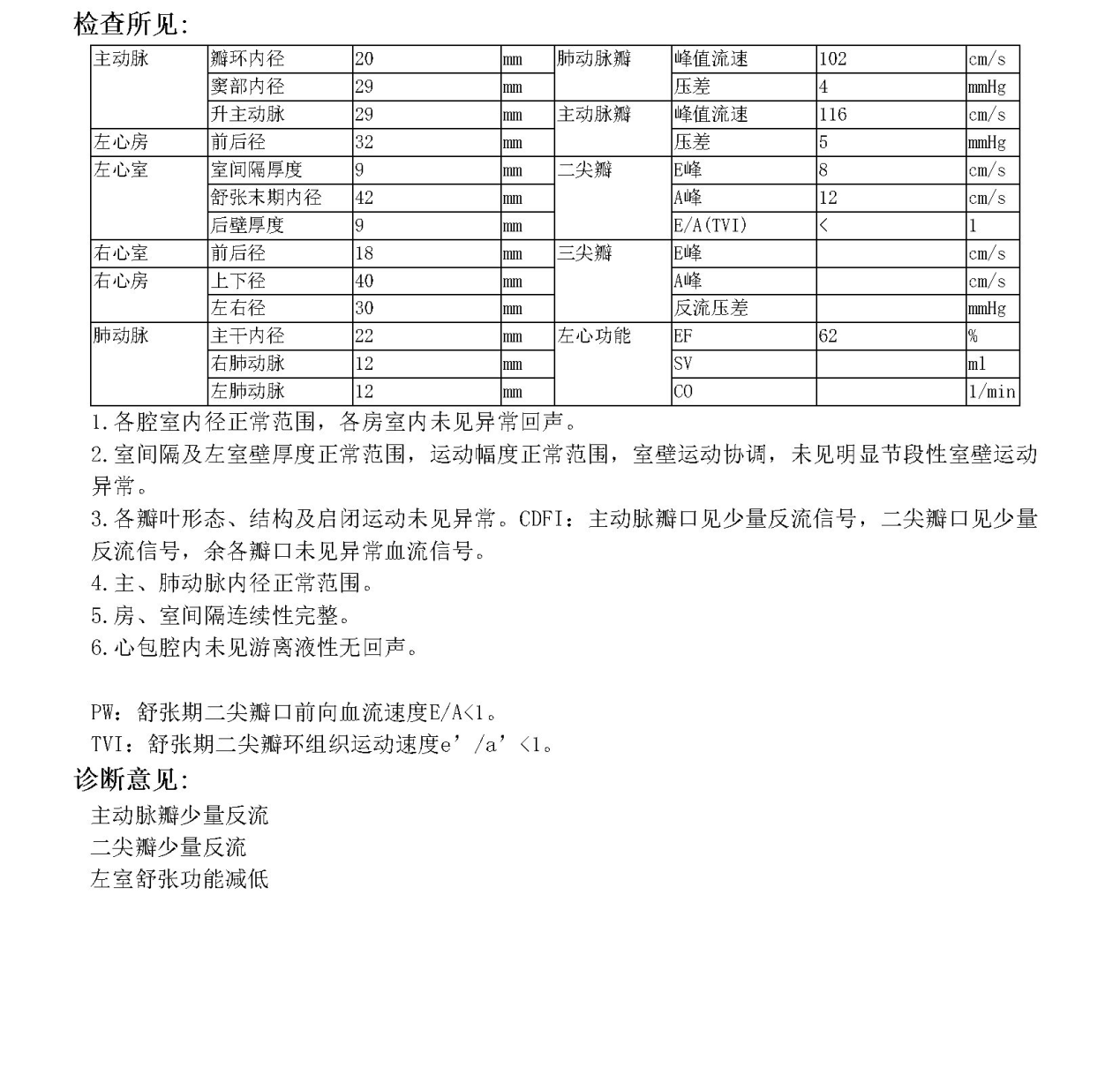

Supplement: Supplementary file 3 — Supplementary Data 2 [file 41746_2026_2648_MOESM3_ESM.zip › echocardiography_reports/237.png]

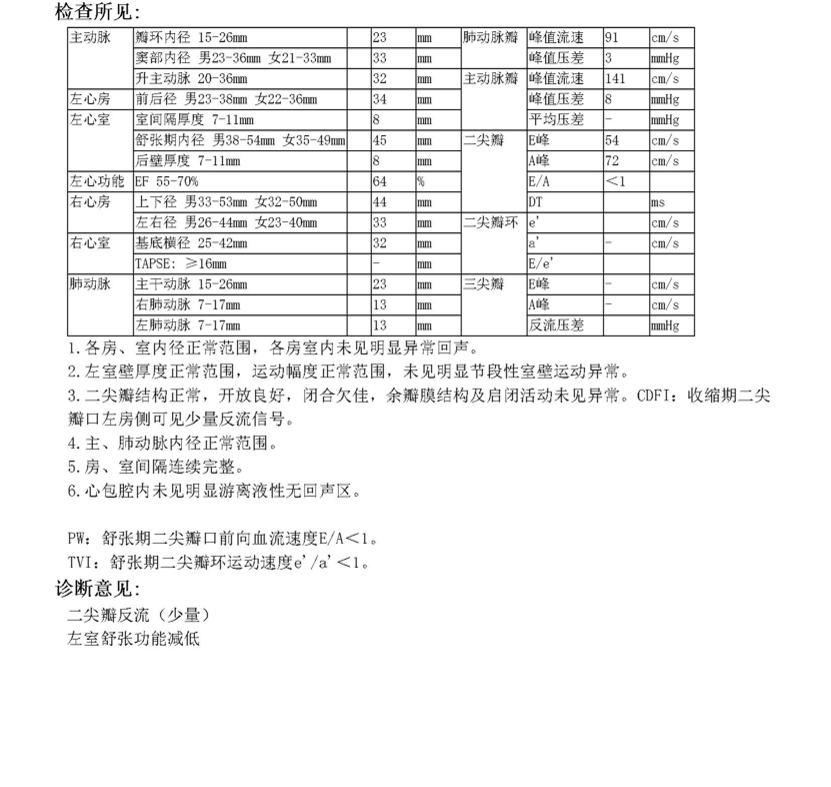

Supplement: Supplementary file 3 — Supplementary Data 2 [file 41746_2026_2648_MOESM3_ESM.zip › echocardiography_reports/238.png]

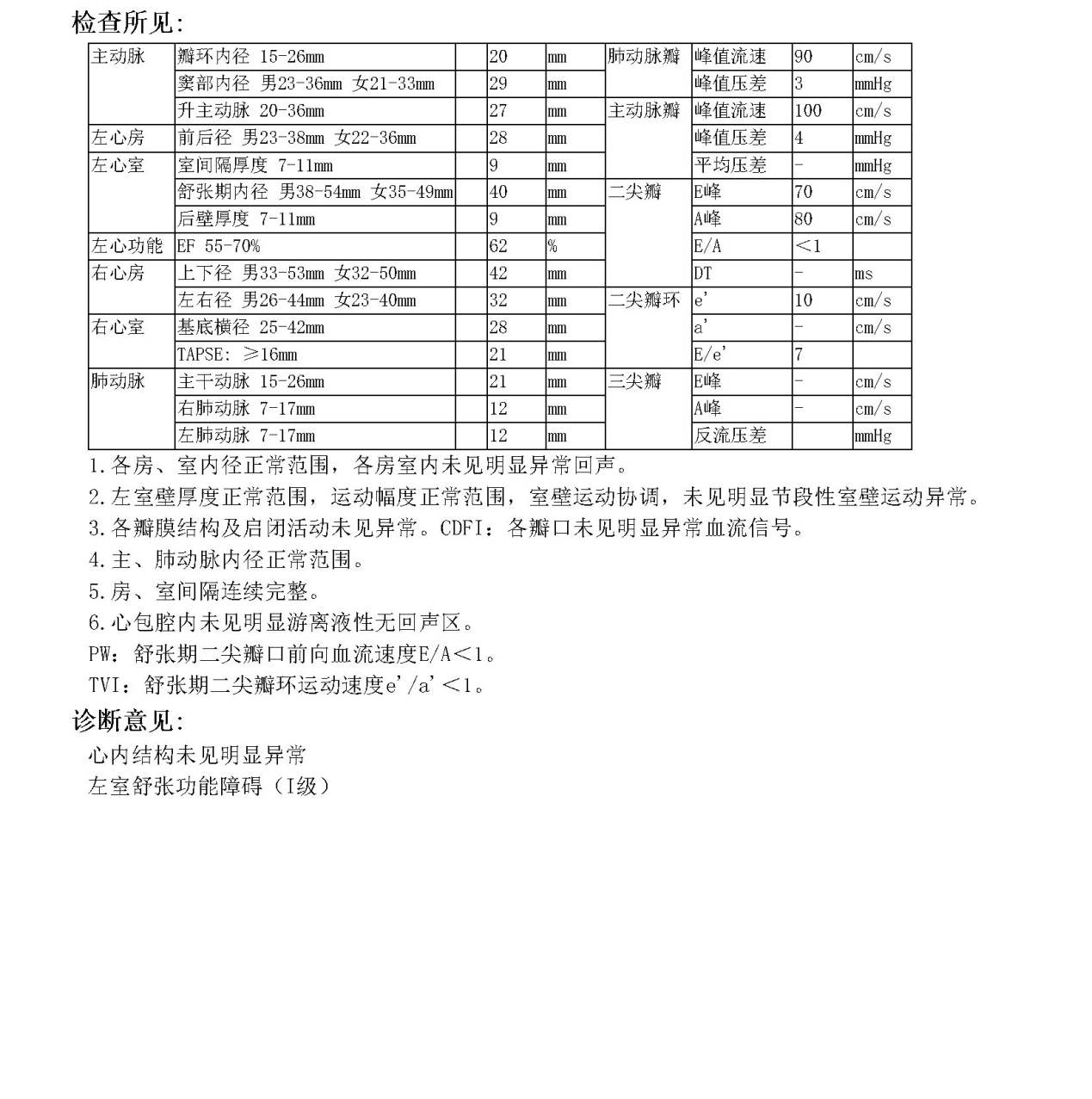

Supplement: Supplementary file 3 — Supplementary Data 2 [file 41746_2026_2648_MOESM3_ESM.zip › echocardiography_reports/239.png]

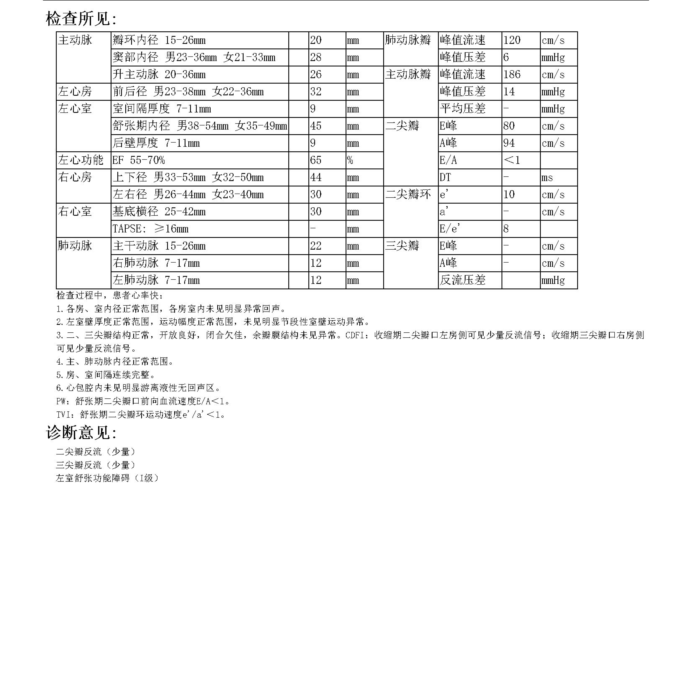

Supplement: Supplementary file 3 — Supplementary Data 2 [file 41746_2026_2648_MOESM3_ESM.zip › echocardiography_reports/240.png]

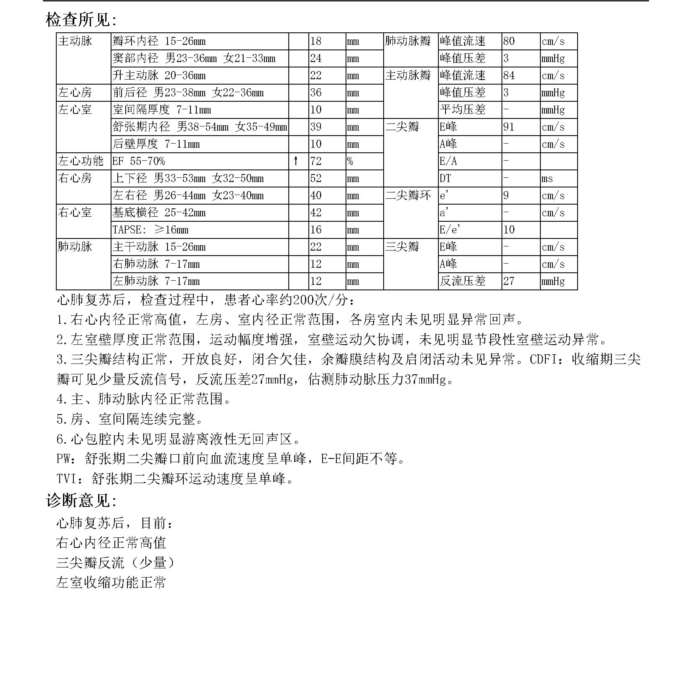

Supplement: Supplementary file 3 — Supplementary Data 2 [file 41746_2026_2648_MOESM3_ESM.zip › echocardiography_reports/241.png]

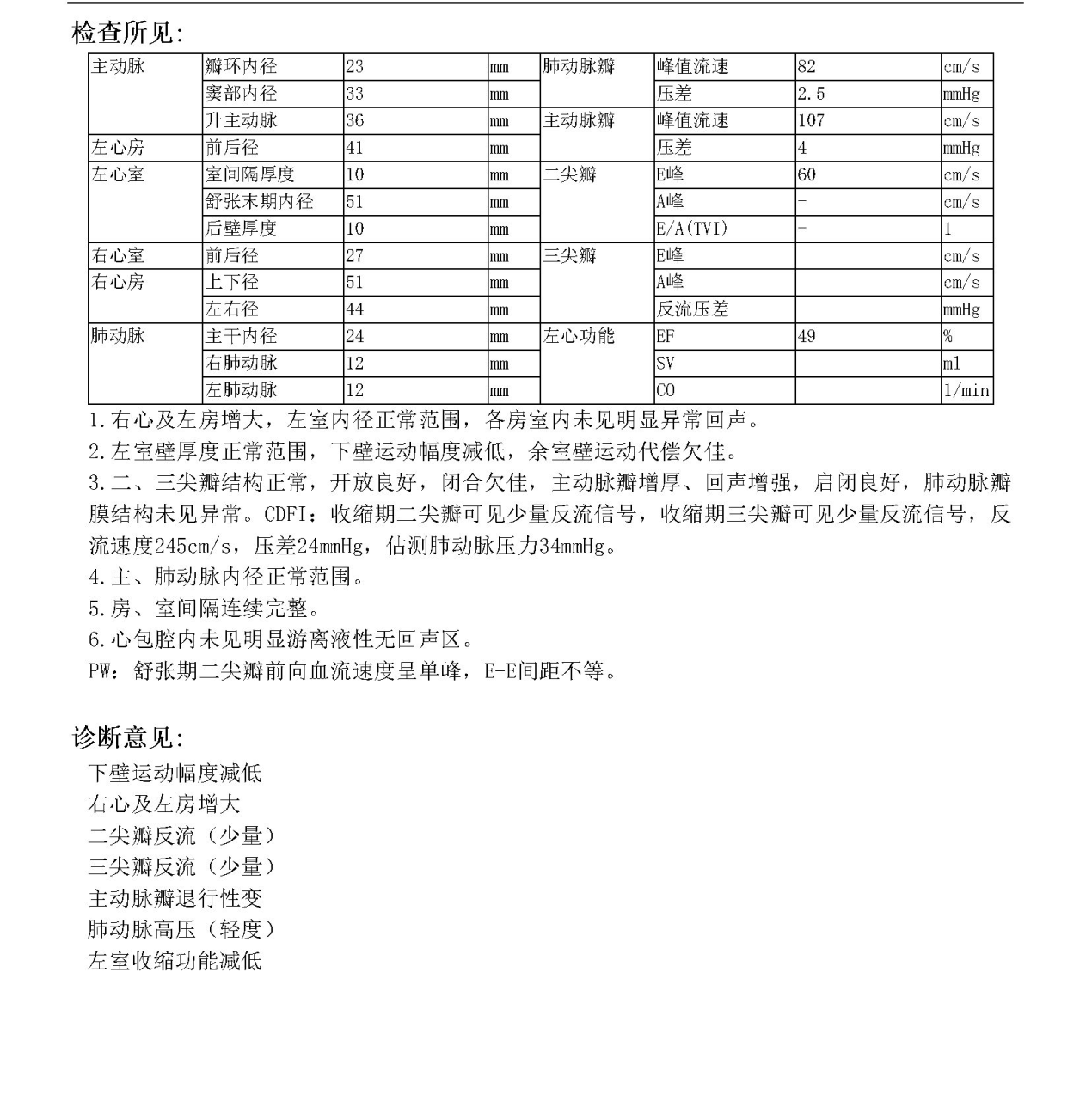

Supplement: Supplementary file 3 — Supplementary Data 2 [file 41746_2026_2648_MOESM3_ESM.zip › echocardiography_reports/242.png]

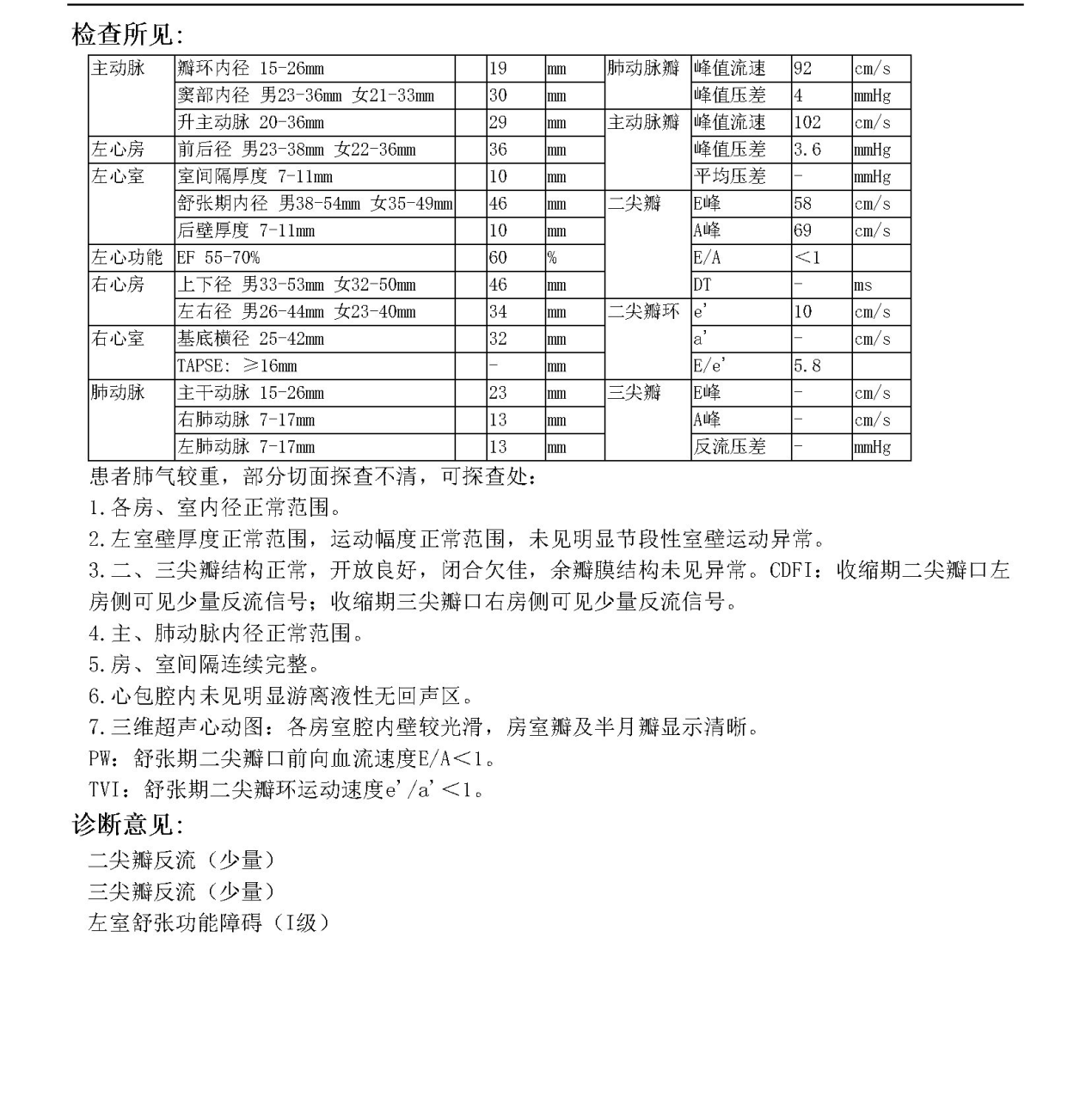

Supplement: Supplementary file 3 — Supplementary Data 2 [file 41746_2026_2648_MOESM3_ESM.zip › echocardiography_reports/243.png]

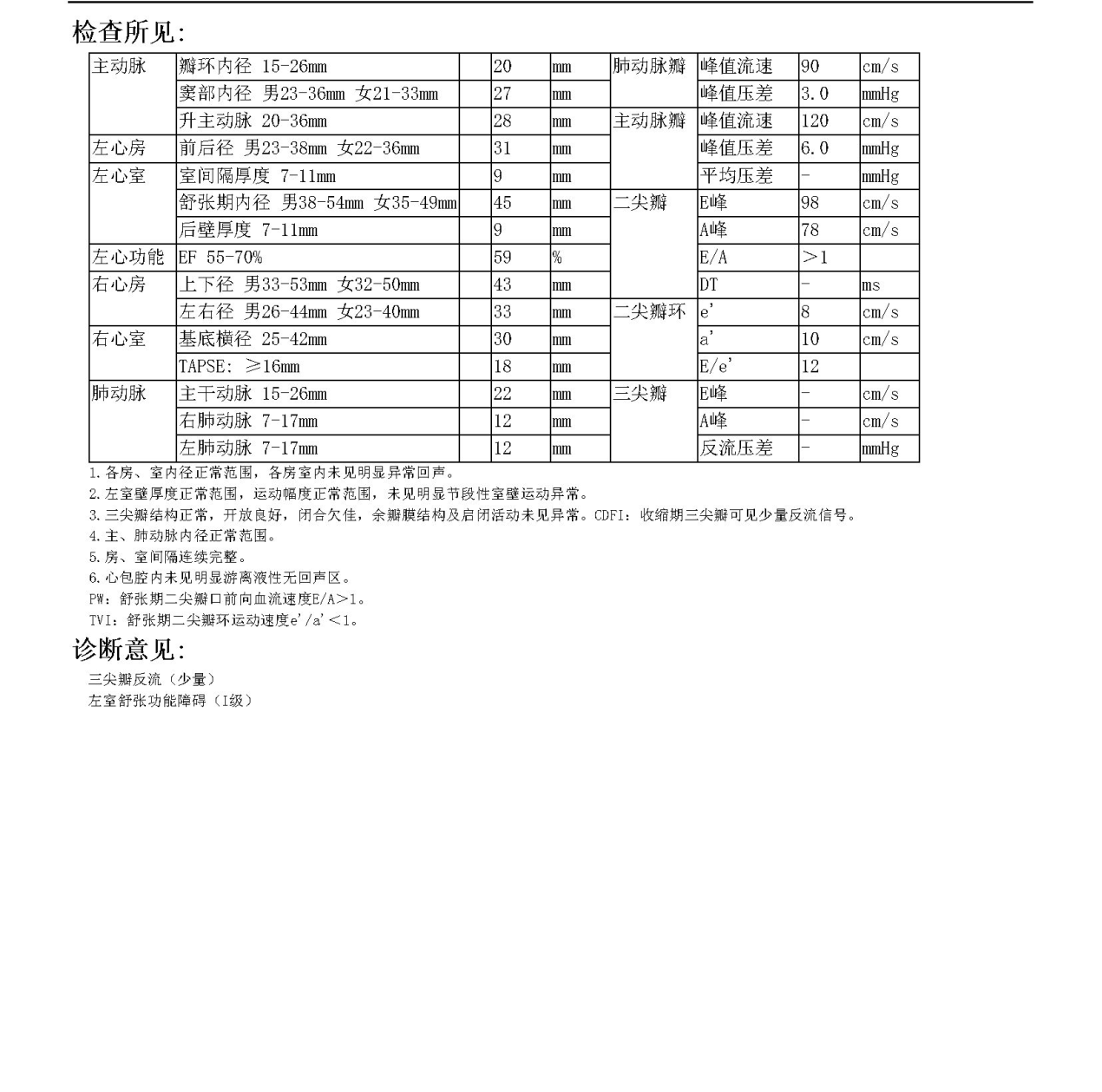

Supplement: Supplementary file 3 — Supplementary Data 2 [file 41746_2026_2648_MOESM3_ESM.zip › echocardiography_reports/244.png]

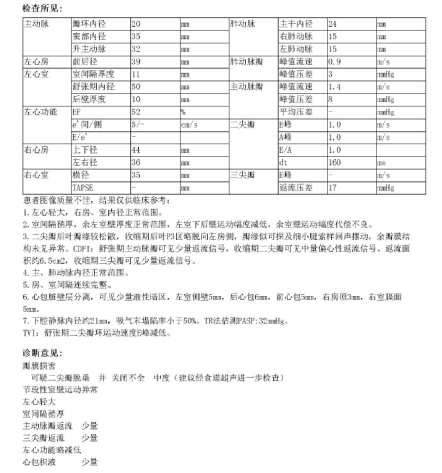

Supplement: Supplementary file 3 — Supplementary Data 2 [file 41746_2026_2648_MOESM3_ESM.zip › echocardiography_reports/245.png]

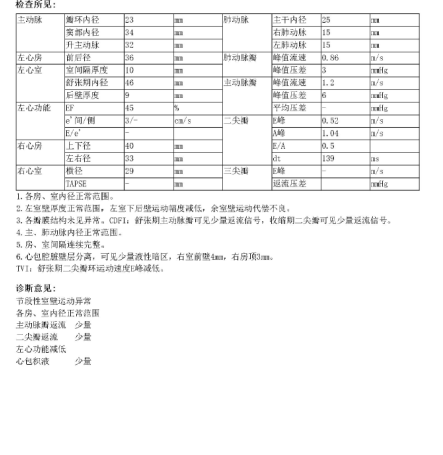

Supplement: Supplementary file 3 — Supplementary Data 2 [file 41746_2026_2648_MOESM3_ESM.zip › echocardiography_reports/246.png]

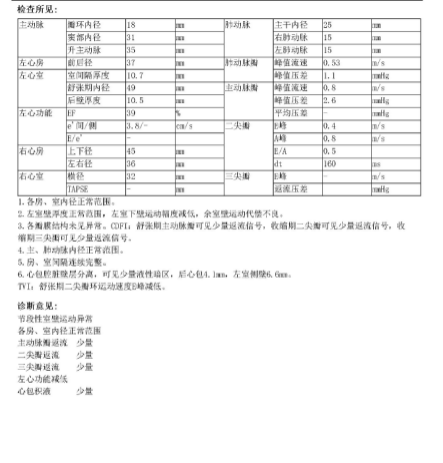

Supplement: Supplementary file 3 — Supplementary Data 2 [file 41746_2026_2648_MOESM3_ESM.zip › echocardiography_reports/247.png]

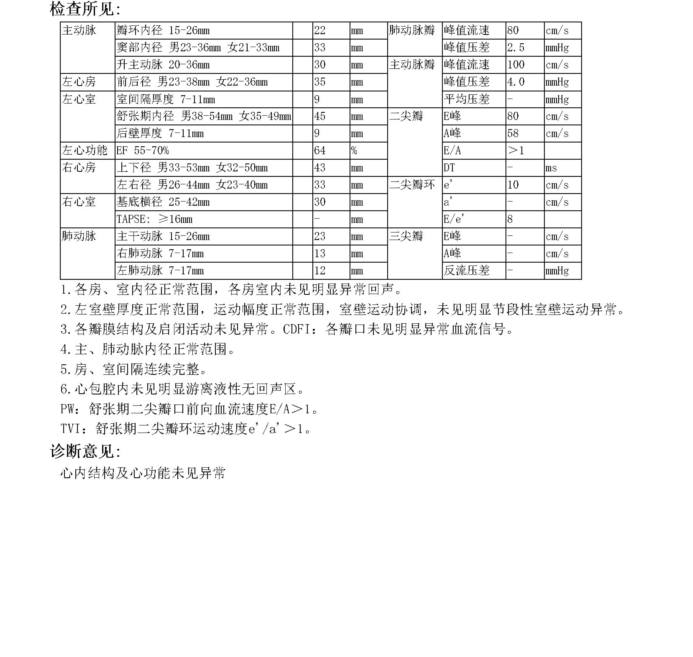

Supplement: Supplementary file 3 — Supplementary Data 2 [file 41746_2026_2648_MOESM3_ESM.zip › echocardiography_reports/248.png]

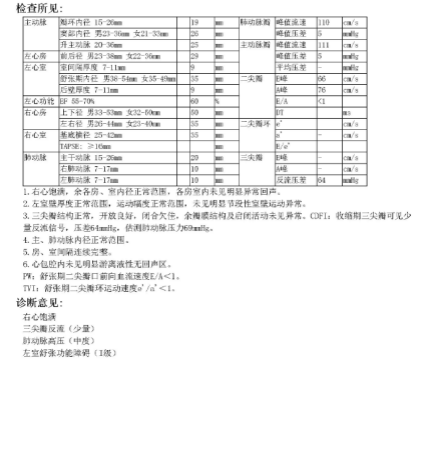

Supplement: Supplementary file 3 — Supplementary Data 2 [file 41746_2026_2648_MOESM3_ESM.zip › echocardiography_reports/249.png]

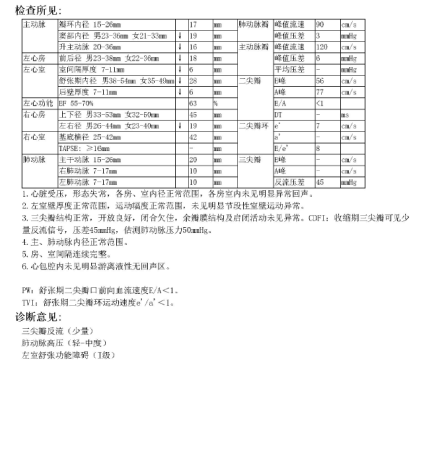

Supplement: Supplementary file 3 — Supplementary Data 2 [file 41746_2026_2648_MOESM3_ESM.zip › echocardiography_reports/250.png]

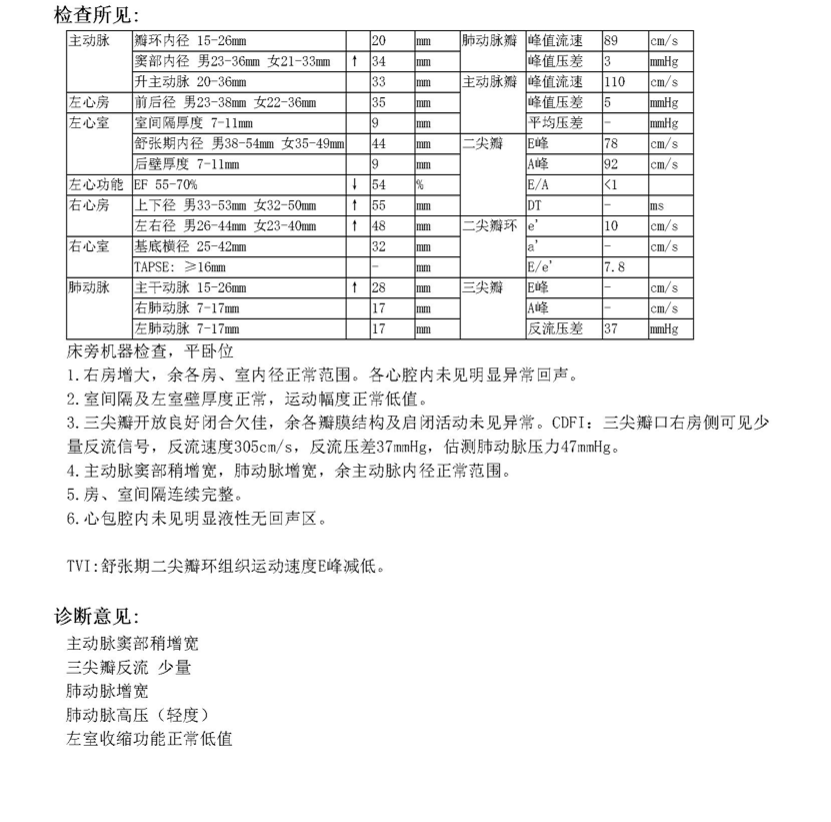

Supplement: Supplementary file 3 — Supplementary Data 2 [file 41746_2026_2648_MOESM3_ESM.zip › echocardiography_reports/251.png]

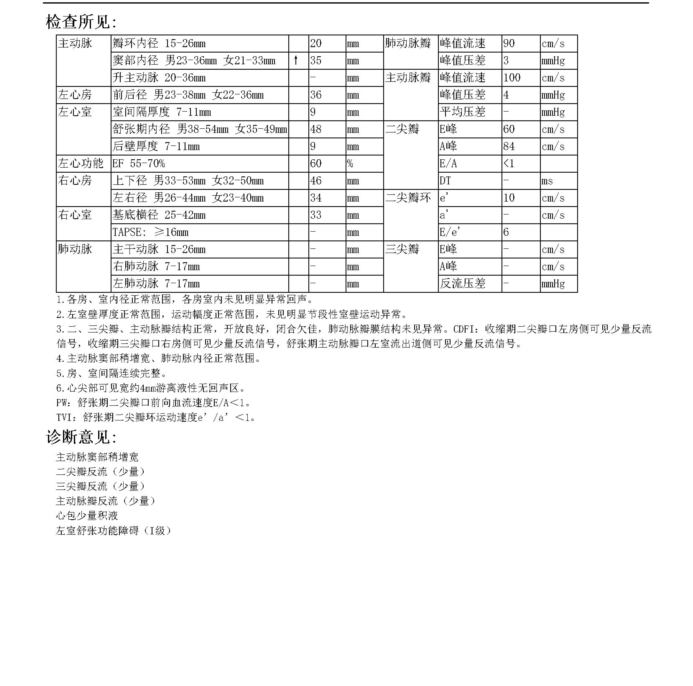

Supplement: Supplementary file 3 — Supplementary Data 2 [file 41746_2026_2648_MOESM3_ESM.zip › echocardiography_reports/252.png]

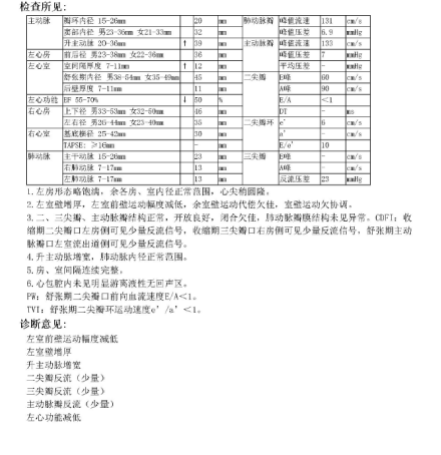

Supplement: Supplementary file 3 — Supplementary Data 2 [file 41746_2026_2648_MOESM3_ESM.zip › echocardiography_reports/253.png]

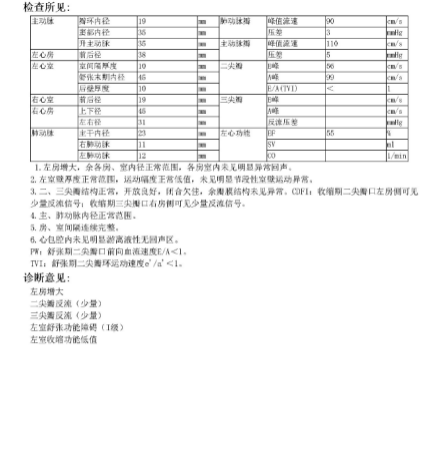

Supplement: Supplementary file 3 — Supplementary Data 2 [file 41746_2026_2648_MOESM3_ESM.zip › echocardiography_reports/254.png]

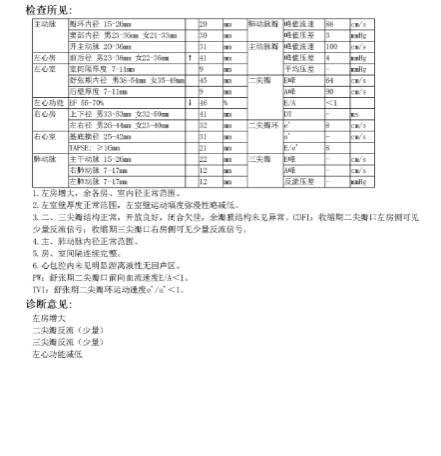

Supplement: Supplementary file 3 — Supplementary Data 2 [file 41746_2026_2648_MOESM3_ESM.zip › echocardiography_reports/255.png]

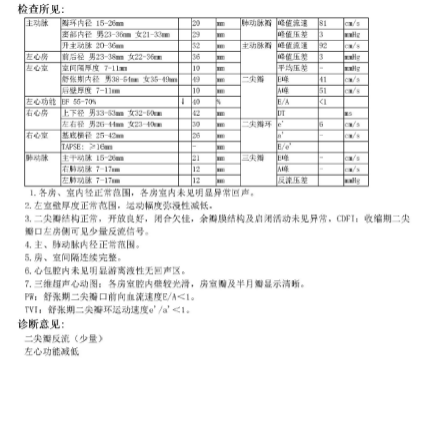

Supplement: Supplementary file 3 — Supplementary Data 2 [file 41746_2026_2648_MOESM3_ESM.zip › echocardiography_reports/256.png]

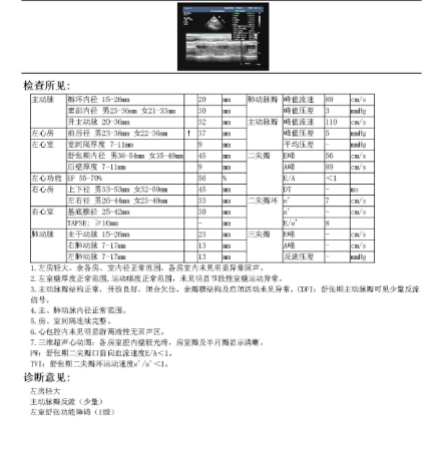

Supplement: Supplementary file 3 — Supplementary Data 2 [file 41746_2026_2648_MOESM3_ESM.zip › echocardiography_reports/257.png]

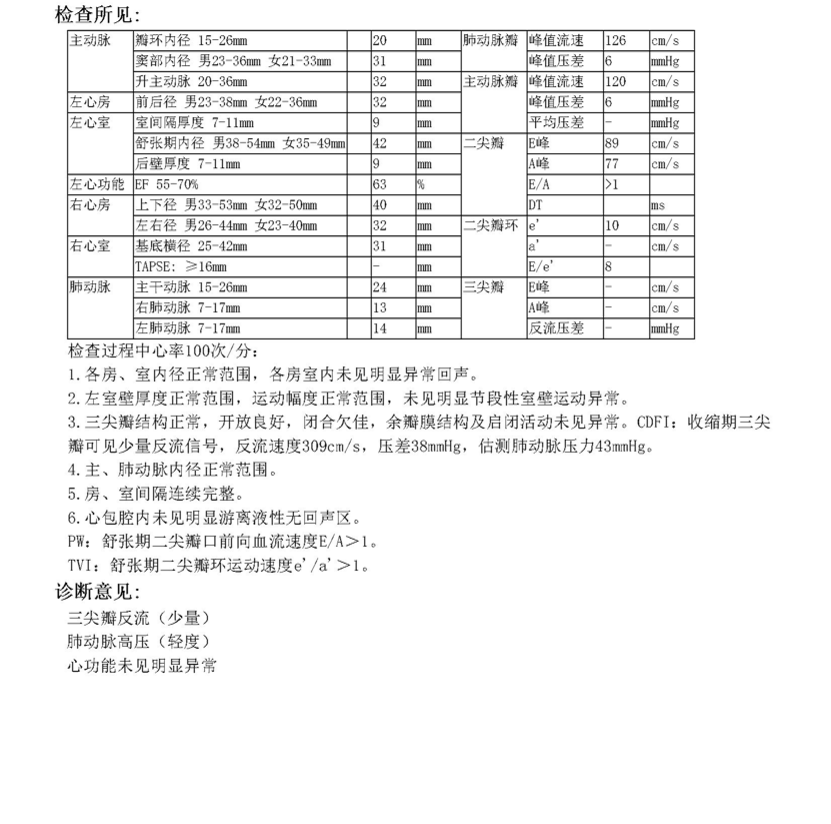

Supplement: Supplementary file 3 — Supplementary Data 2 [file 41746_2026_2648_MOESM3_ESM.zip › echocardiography_reports/258.png]

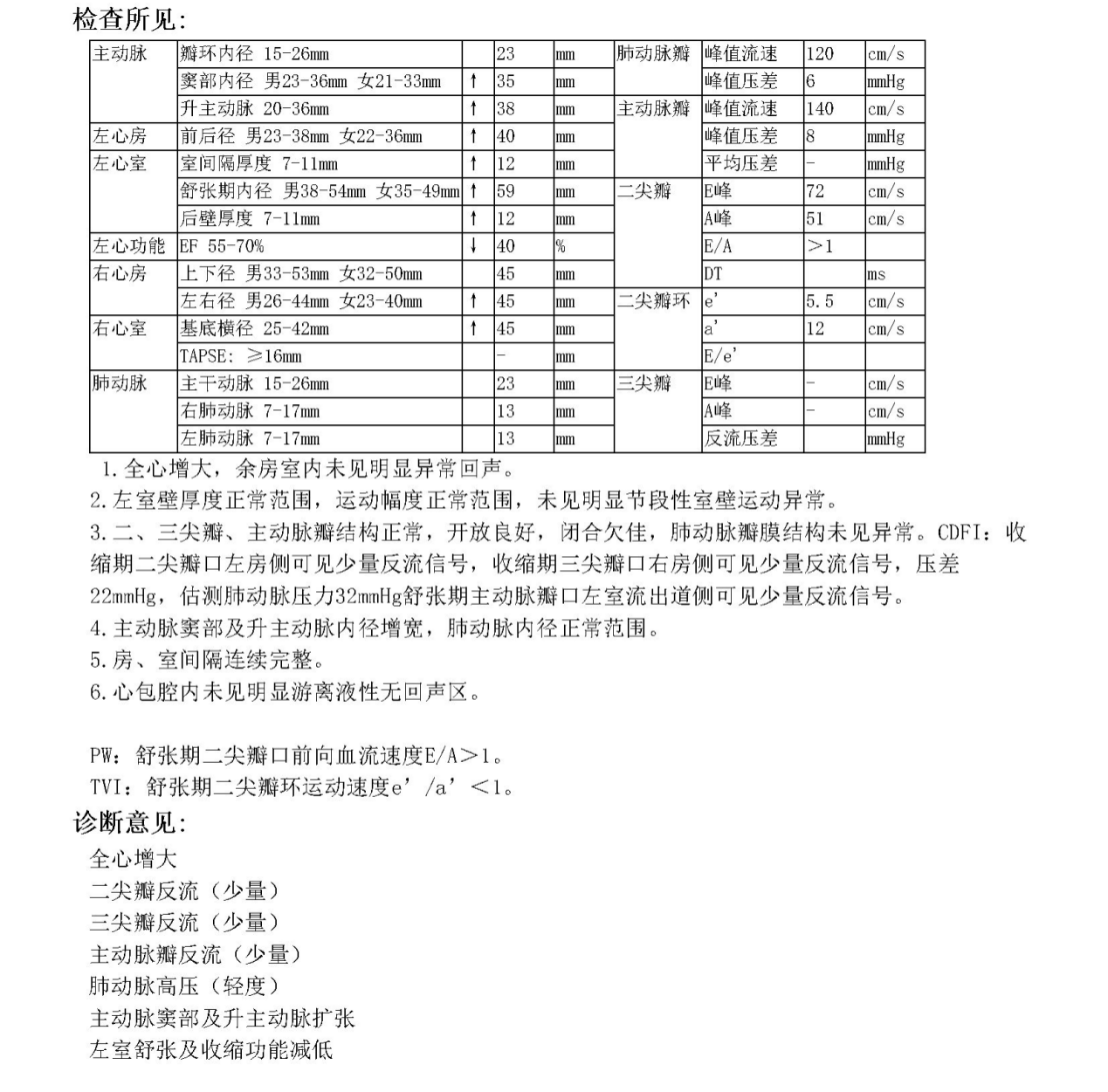

Supplement: Supplementary file 3 — Supplementary Data 2 [file 41746_2026_2648_MOESM3_ESM.zip › echocardiography_reports/259.png]

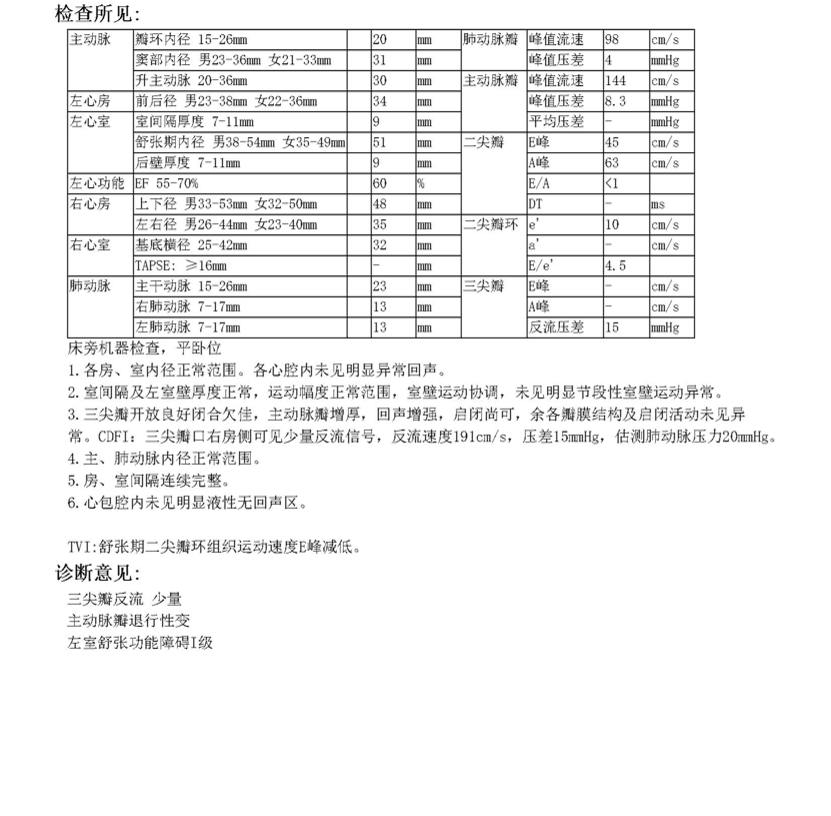

Supplement: Supplementary file 3 — Supplementary Data 2 [file 41746_2026_2648_MOESM3_ESM.zip › echocardiography_reports/260.png]

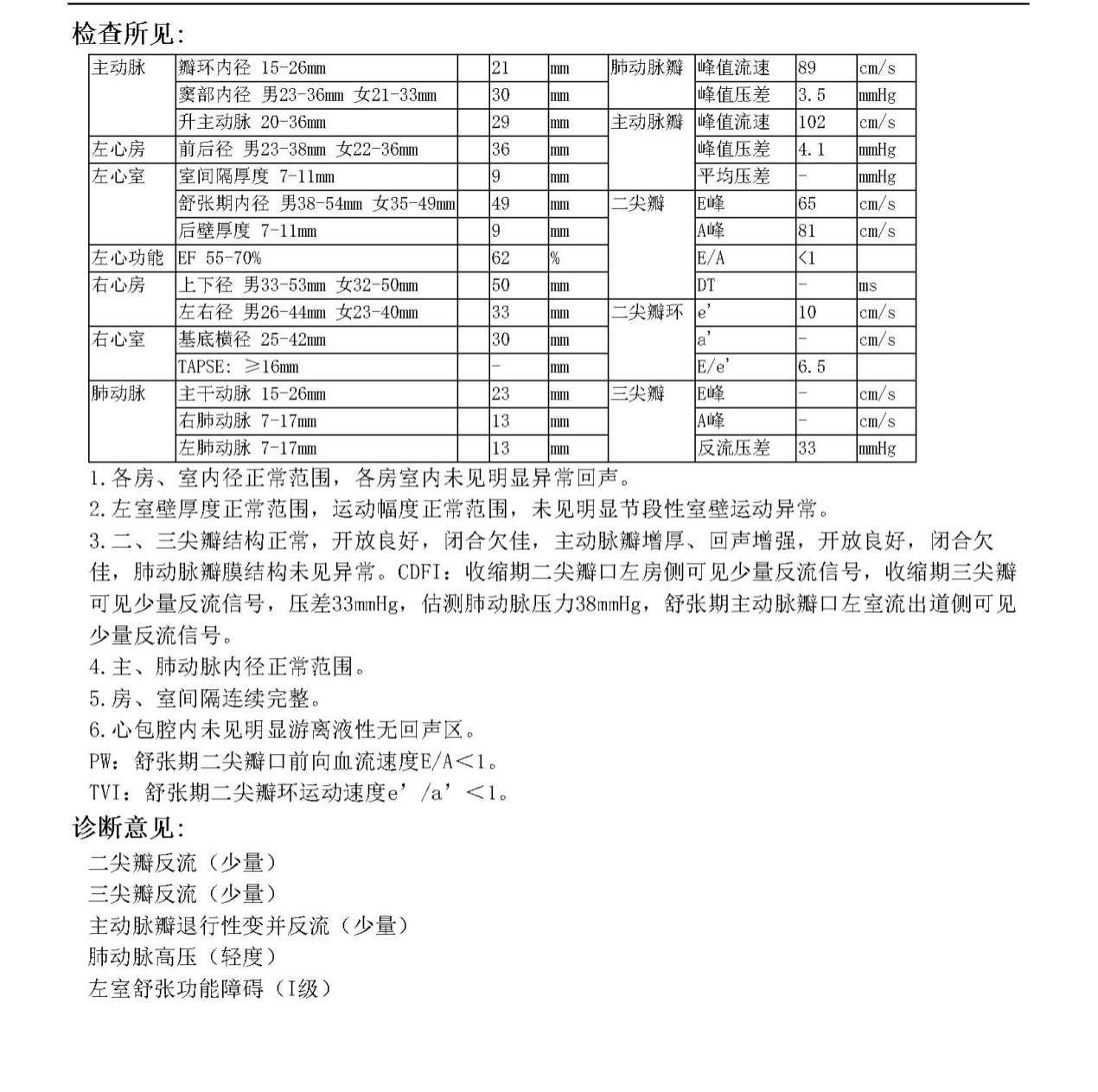

Supplement: Supplementary file 3 — Supplementary Data 2 [file 41746_2026_2648_MOESM3_ESM.zip › echocardiography_reports/261.png]

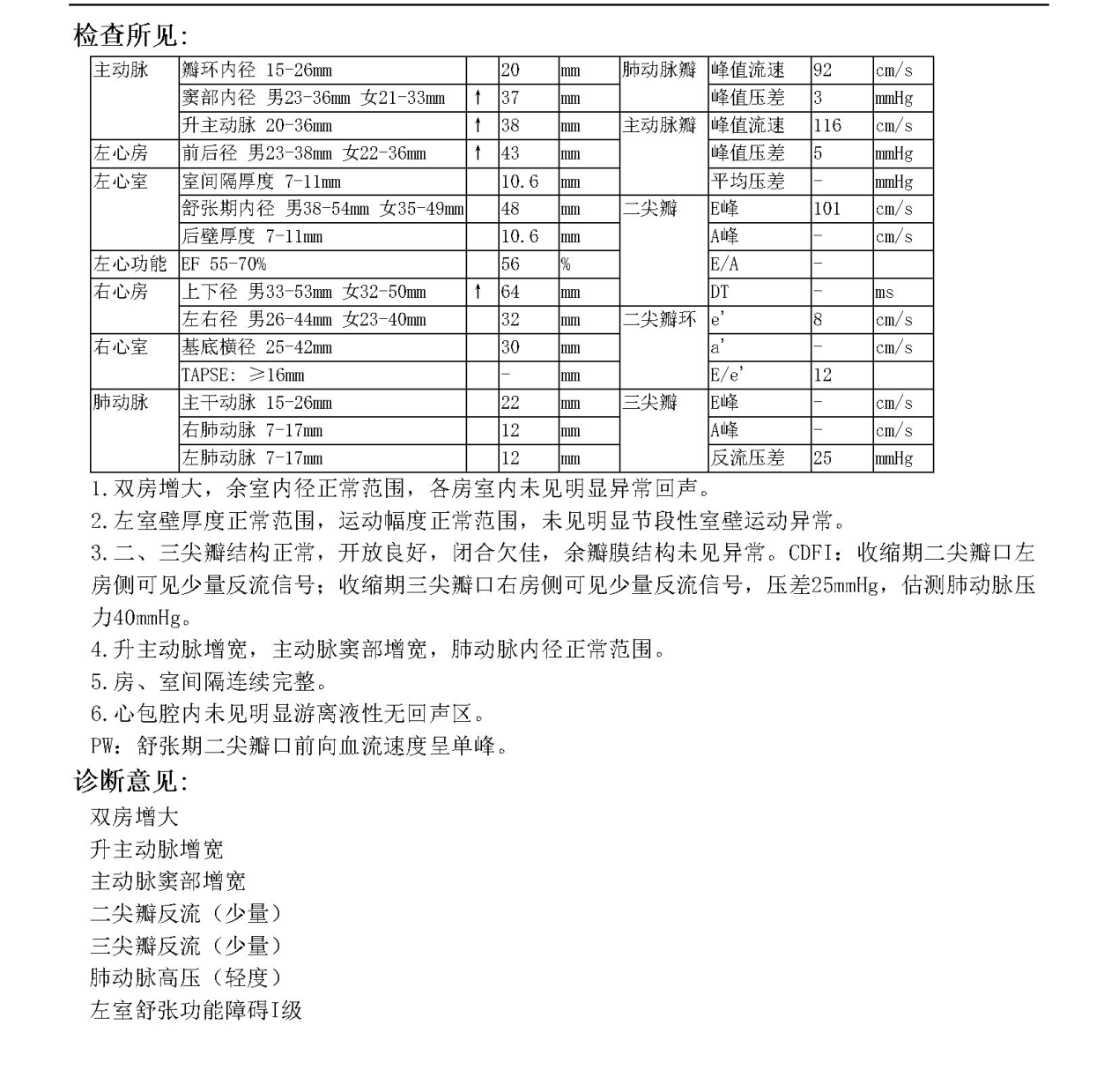

Supplement: Supplementary file 3 — Supplementary Data 2 [file 41746_2026_2648_MOESM3_ESM.zip › echocardiography_reports/262.png]

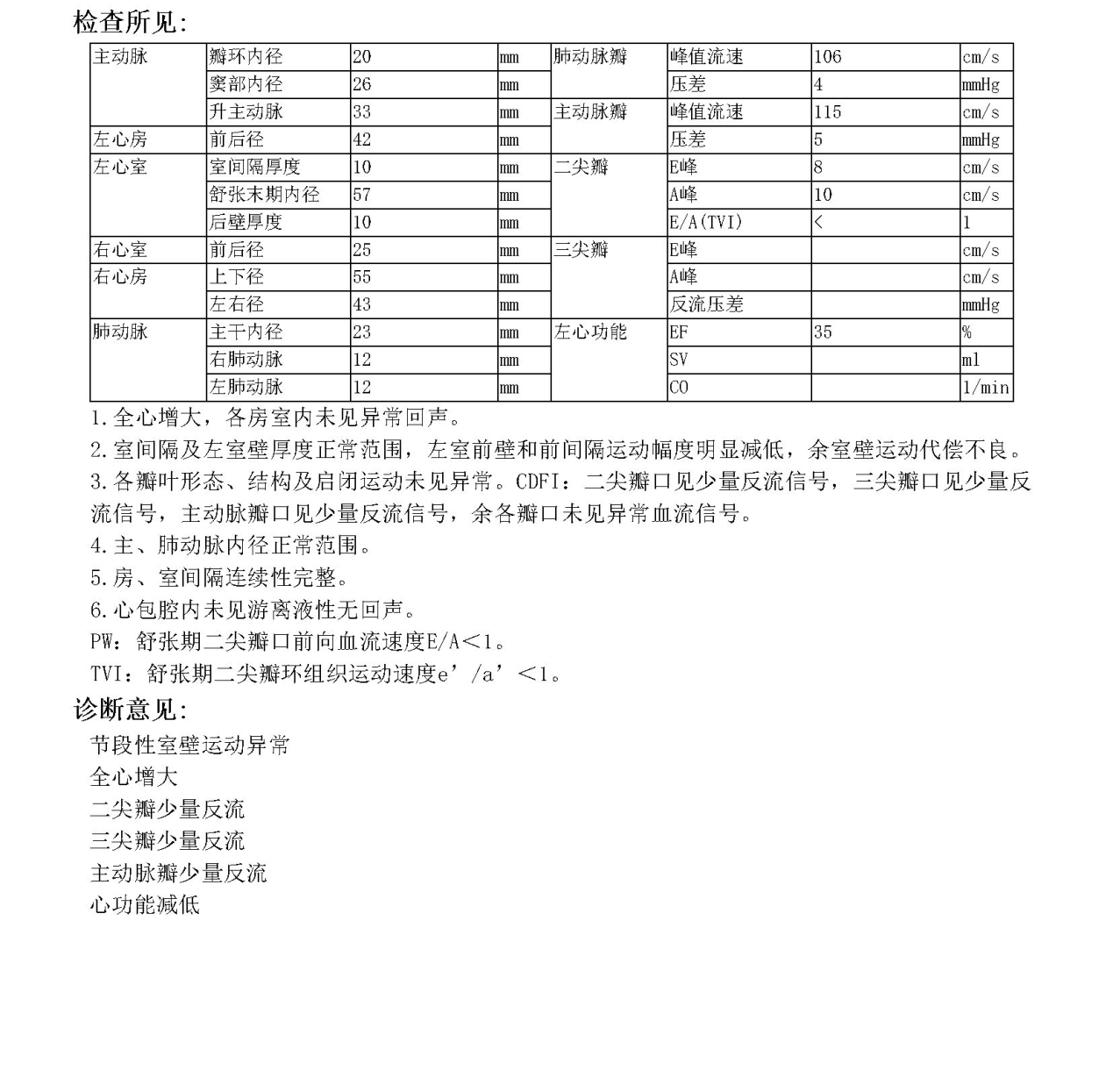

Supplement: Supplementary file 3 — Supplementary Data 2 [file 41746_2026_2648_MOESM3_ESM.zip › echocardiography_reports/263.png]

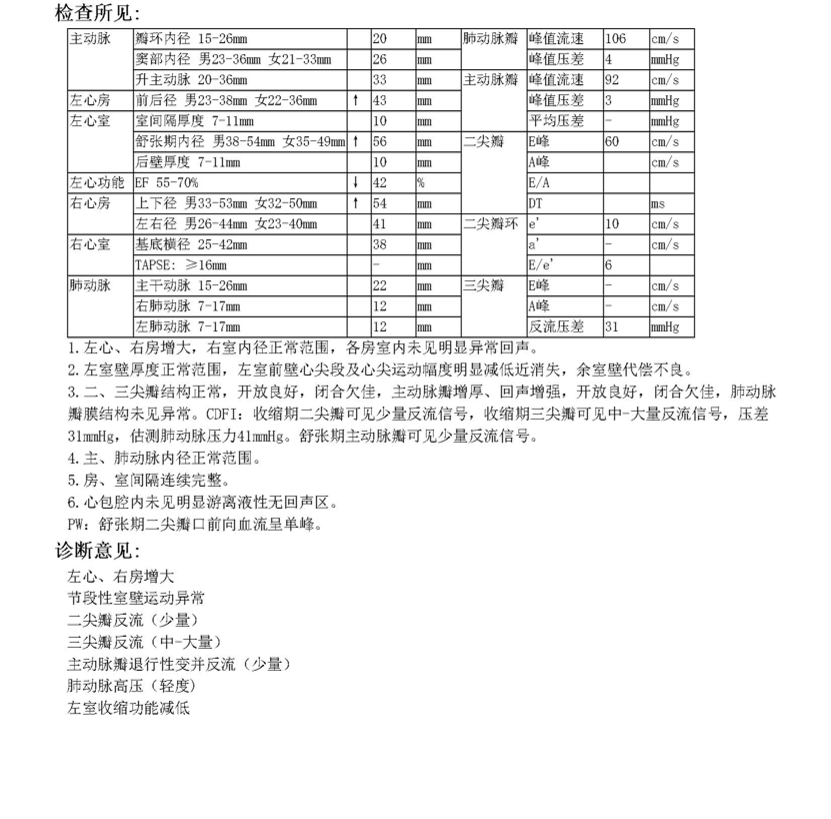

Supplement: Supplementary file 3 — Supplementary Data 2 [file 41746_2026_2648_MOESM3_ESM.zip › echocardiography_reports/264.png]

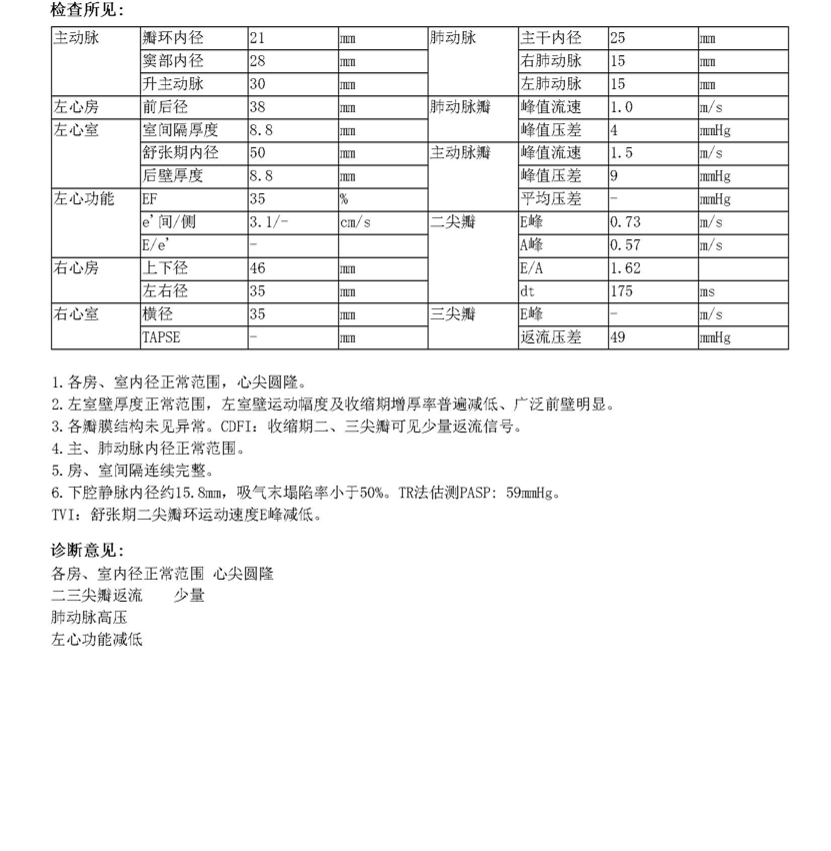

Supplement: Supplementary file 3 — Supplementary Data 2 [file 41746_2026_2648_MOESM3_ESM.zip › echocardiography_reports/265.png]

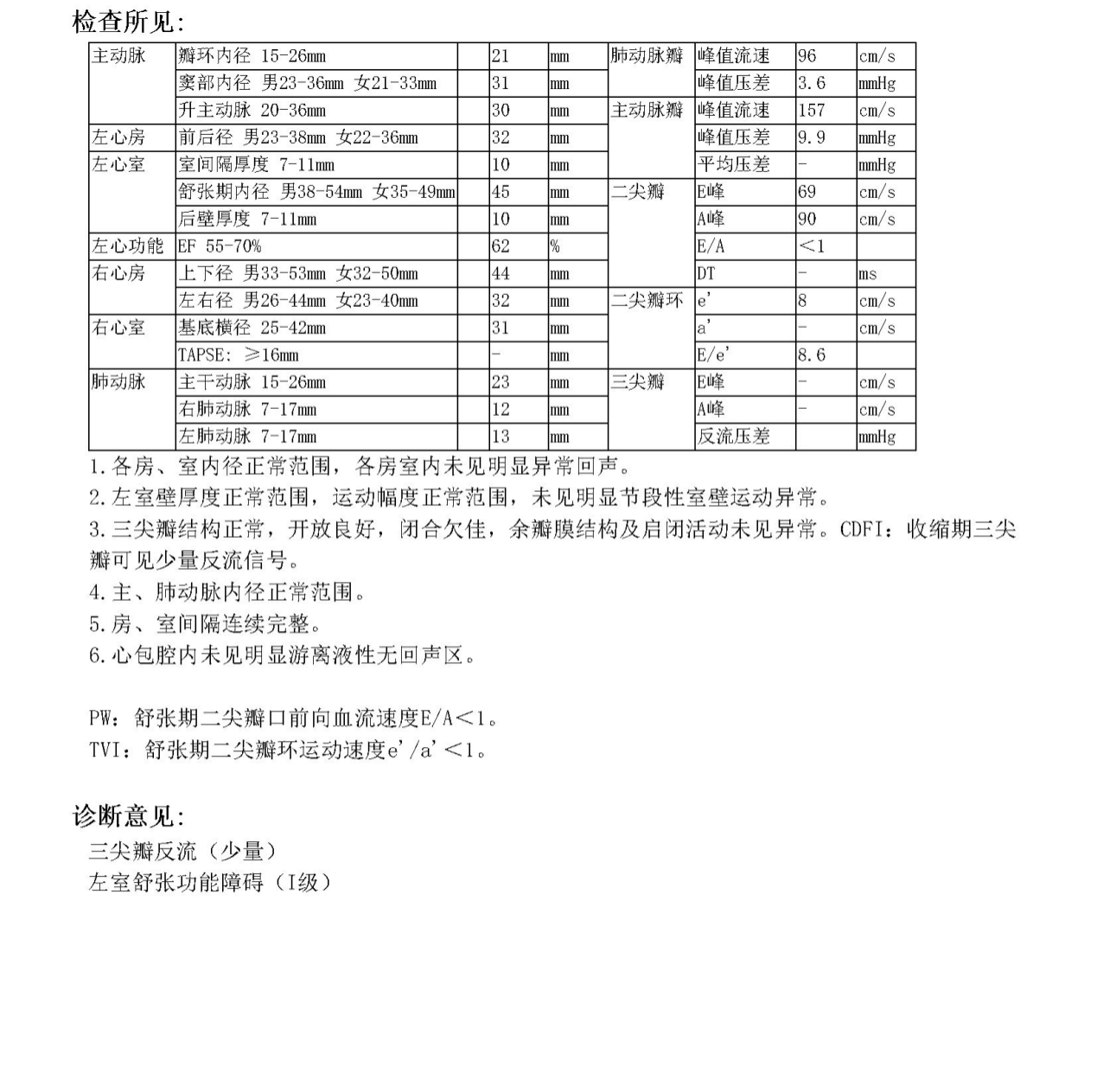

Supplement: Supplementary file 3 — Supplementary Data 2 [file 41746_2026_2648_MOESM3_ESM.zip › echocardiography_reports/266.png]

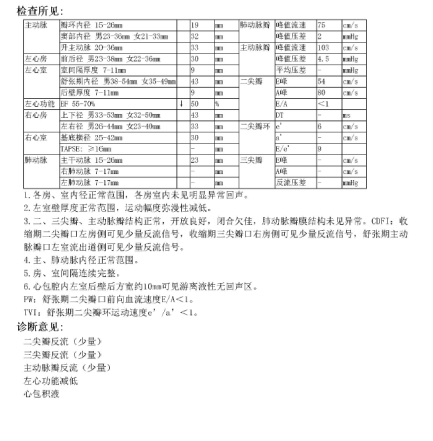

Supplement: Supplementary file 3 — Supplementary Data 2 [file 41746_2026_2648_MOESM3_ESM.zip › echocardiography_reports/267.png]

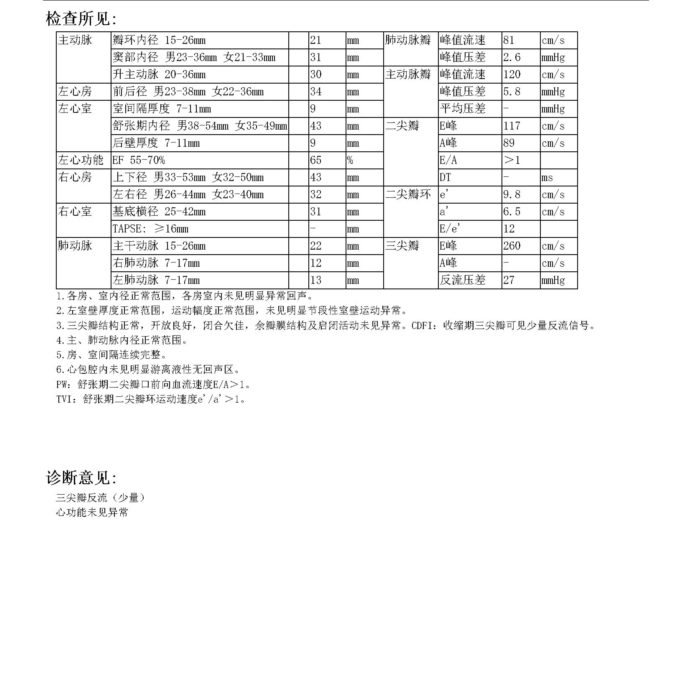

Supplement: Supplementary file 3 — Supplementary Data 2 [file 41746_2026_2648_MOESM3_ESM.zip › echocardiography_reports/268.png]

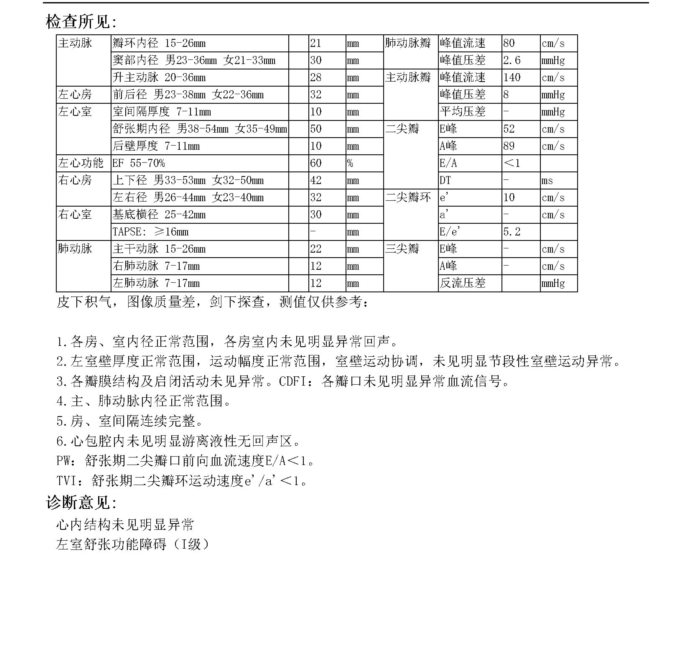

Supplement: Supplementary file 3 — Supplementary Data 2 [file 41746_2026_2648_MOESM3_ESM.zip › echocardiography_reports/269.png]

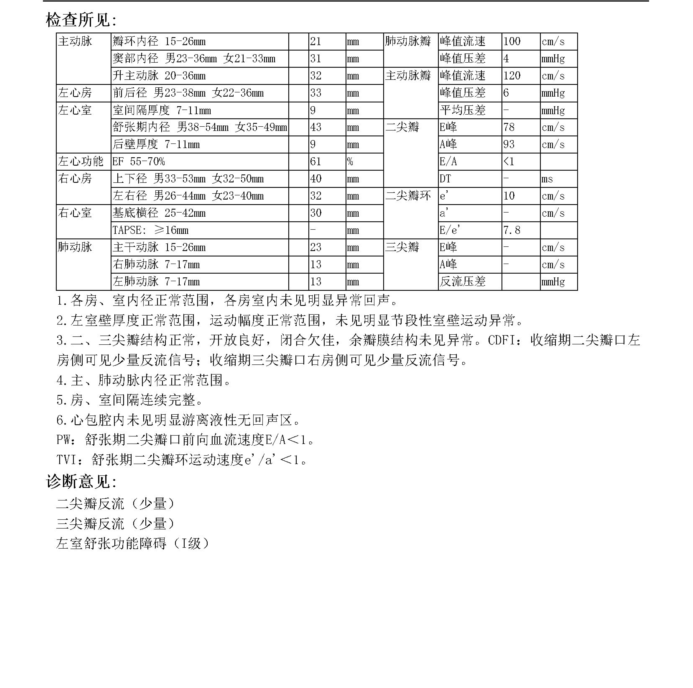

Supplement: Supplementary file 3 — Supplementary Data 2 [file 41746_2026_2648_MOESM3_ESM.zip › echocardiography_reports/270.png]

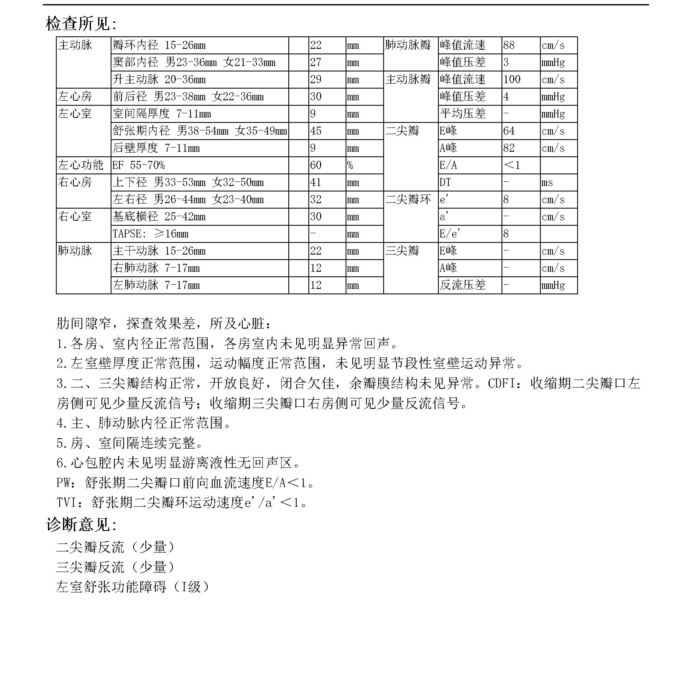

Supplement: Supplementary file 3 — Supplementary Data 2 [file 41746_2026_2648_MOESM3_ESM.zip › echocardiography_reports/271.png]

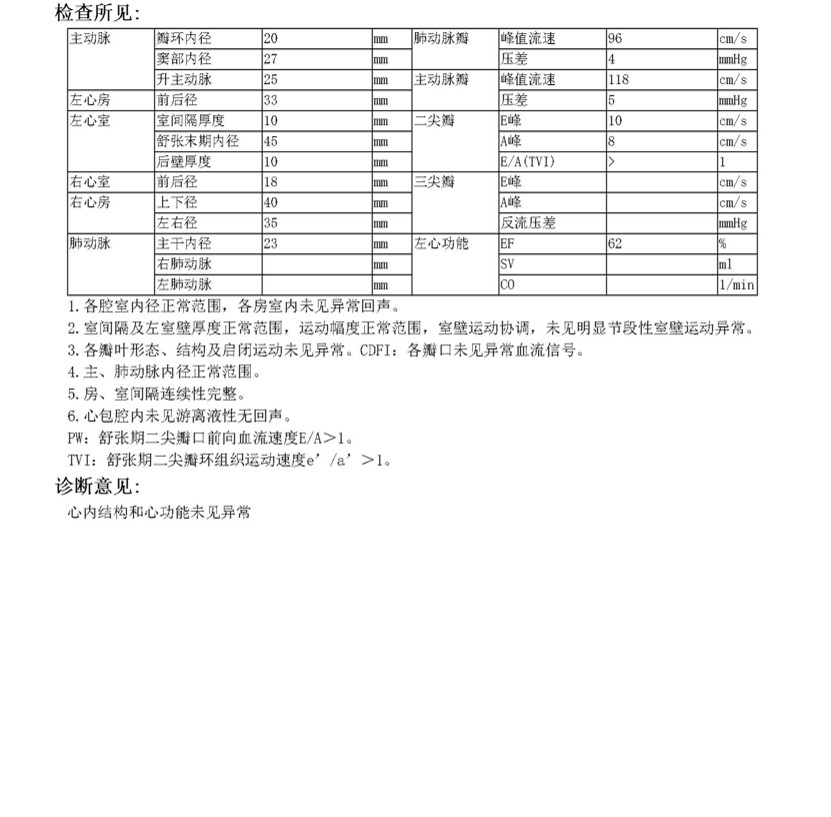

Supplement: Supplementary file 3 — Supplementary Data 2 [file 41746_2026_2648_MOESM3_ESM.zip › echocardiography_reports/272.png]

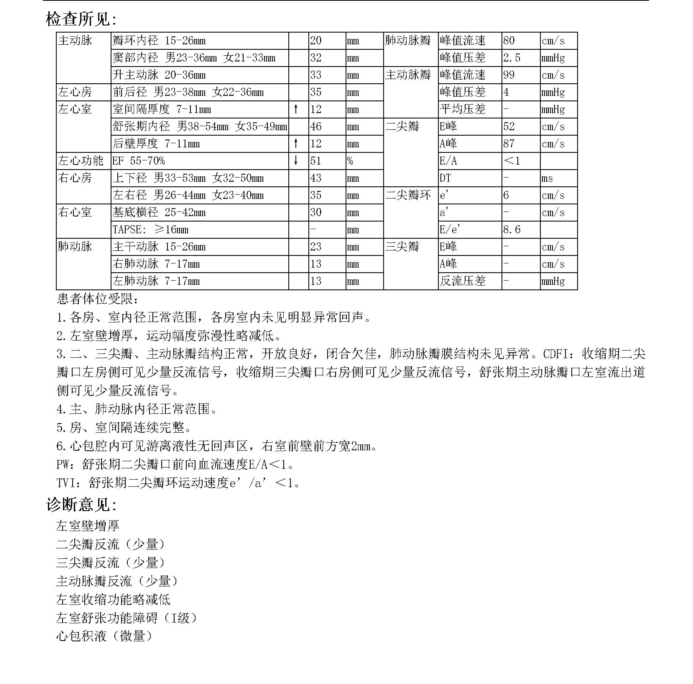

Supplement: Supplementary file 3 — Supplementary Data 2 [file 41746_2026_2648_MOESM3_ESM.zip › echocardiography_reports/273.png]

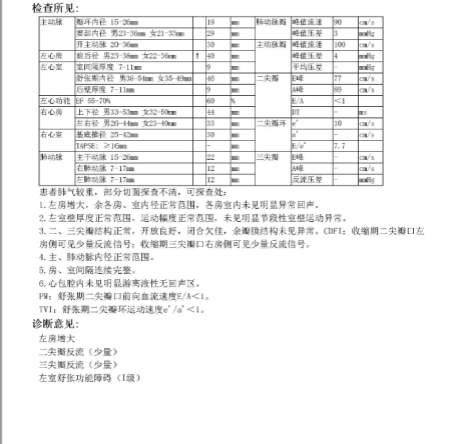

Supplement: Supplementary file 3 — Supplementary Data 2 [file 41746_2026_2648_MOESM3_ESM.zip › echocardiography_reports/274.png]

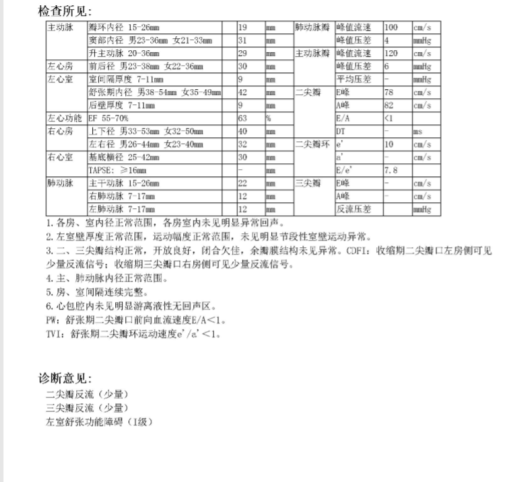

Supplement: Supplementary file 3 — Supplementary Data 2 [file 41746_2026_2648_MOESM3_ESM.zip › echocardiography_reports/275.png]

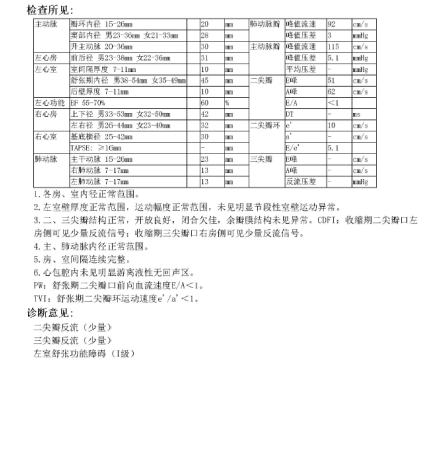

Supplement: Supplementary file 3 — Supplementary Data 2 [file 41746_2026_2648_MOESM3_ESM.zip › echocardiography_reports/276.png]

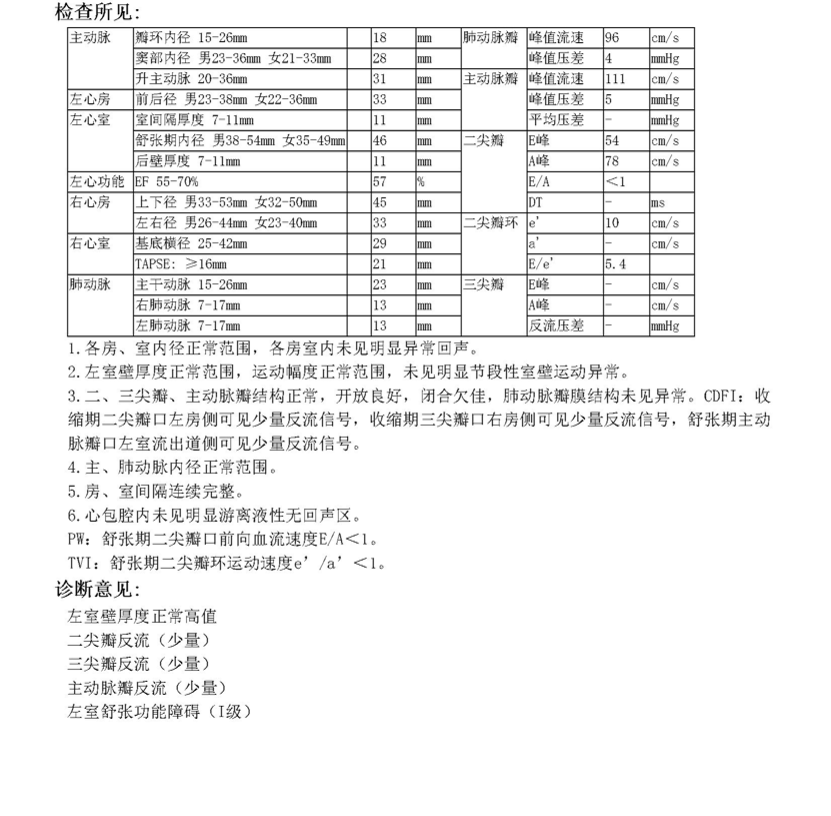

Supplement: Supplementary file 3 — Supplementary Data 2 [file 41746_2026_2648_MOESM3_ESM.zip › echocardiography_reports/277.png]

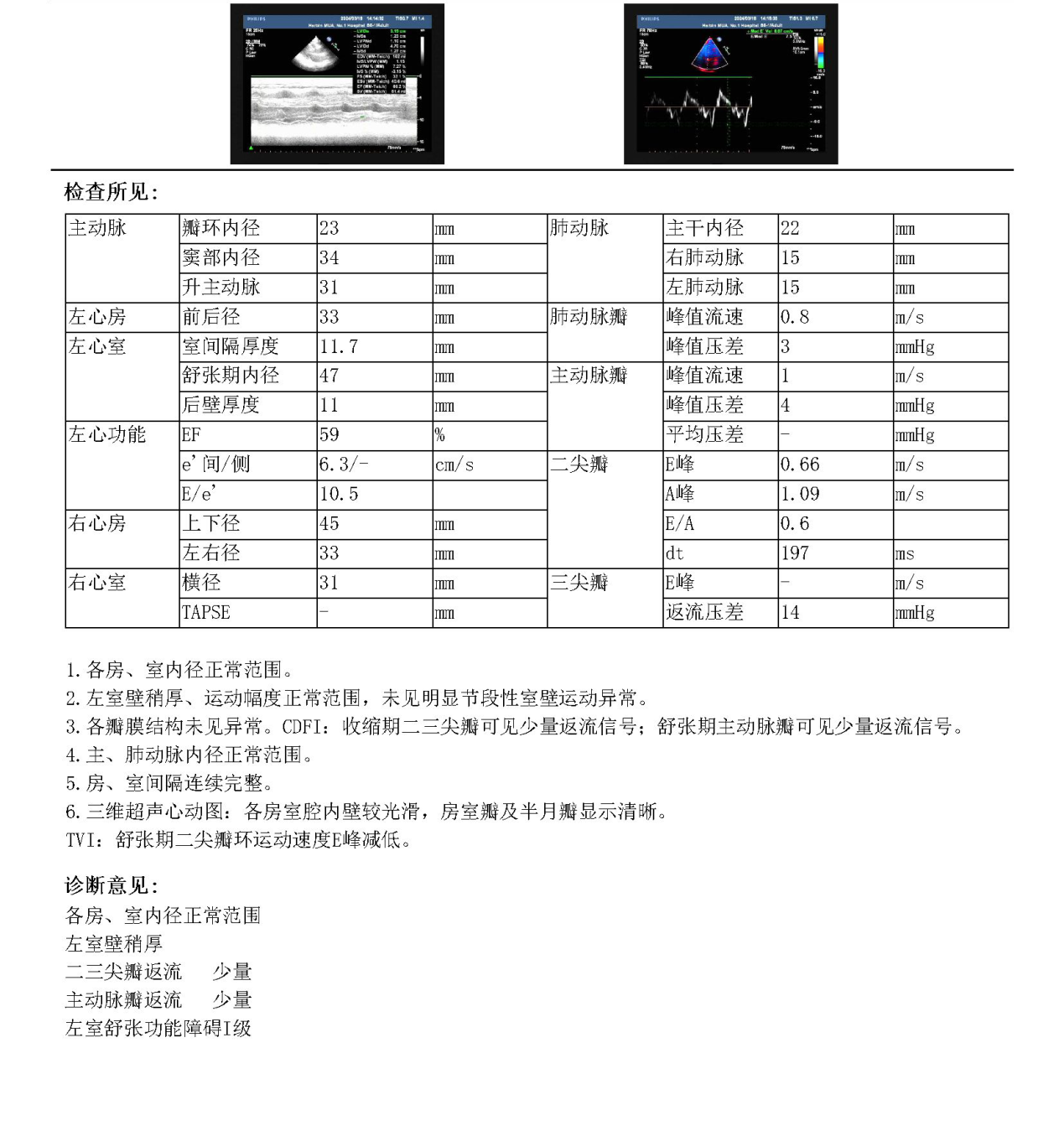

Supplement: Supplementary file 3 — Supplementary Data 2 [file 41746_2026_2648_MOESM3_ESM.zip › echocardiography_reports/278.png]

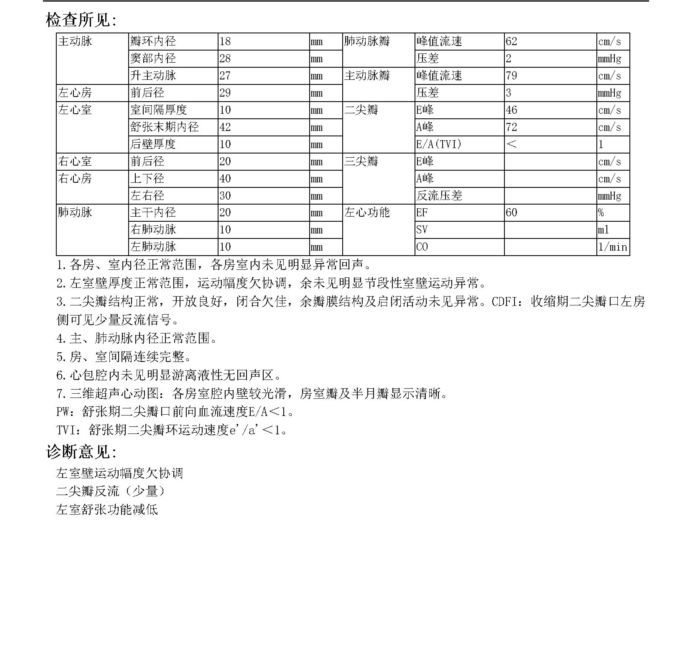

Supplement: Supplementary file 3 — Supplementary Data 2 [file 41746_2026_2648_MOESM3_ESM.zip › echocardiography_reports/279.png]

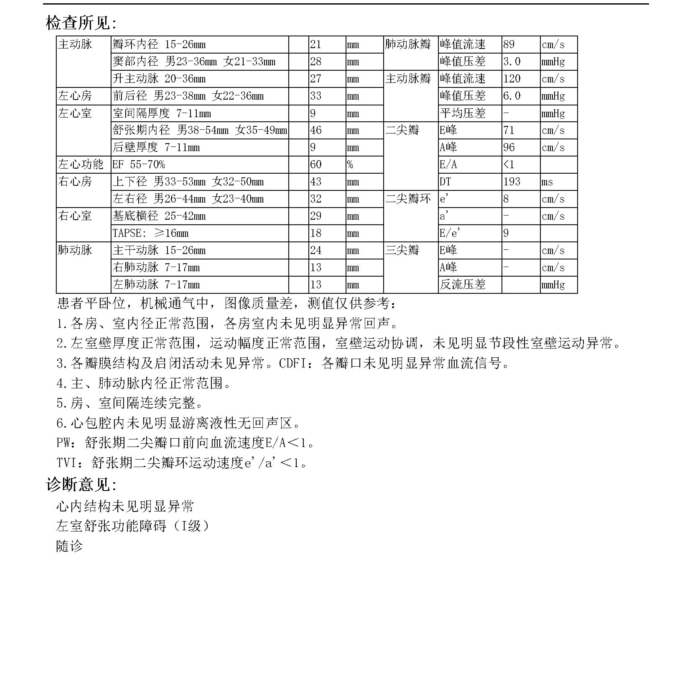

Supplement: Supplementary file 3 — Supplementary Data 2 [file 41746_2026_2648_MOESM3_ESM.zip › echocardiography_reports/280.png]

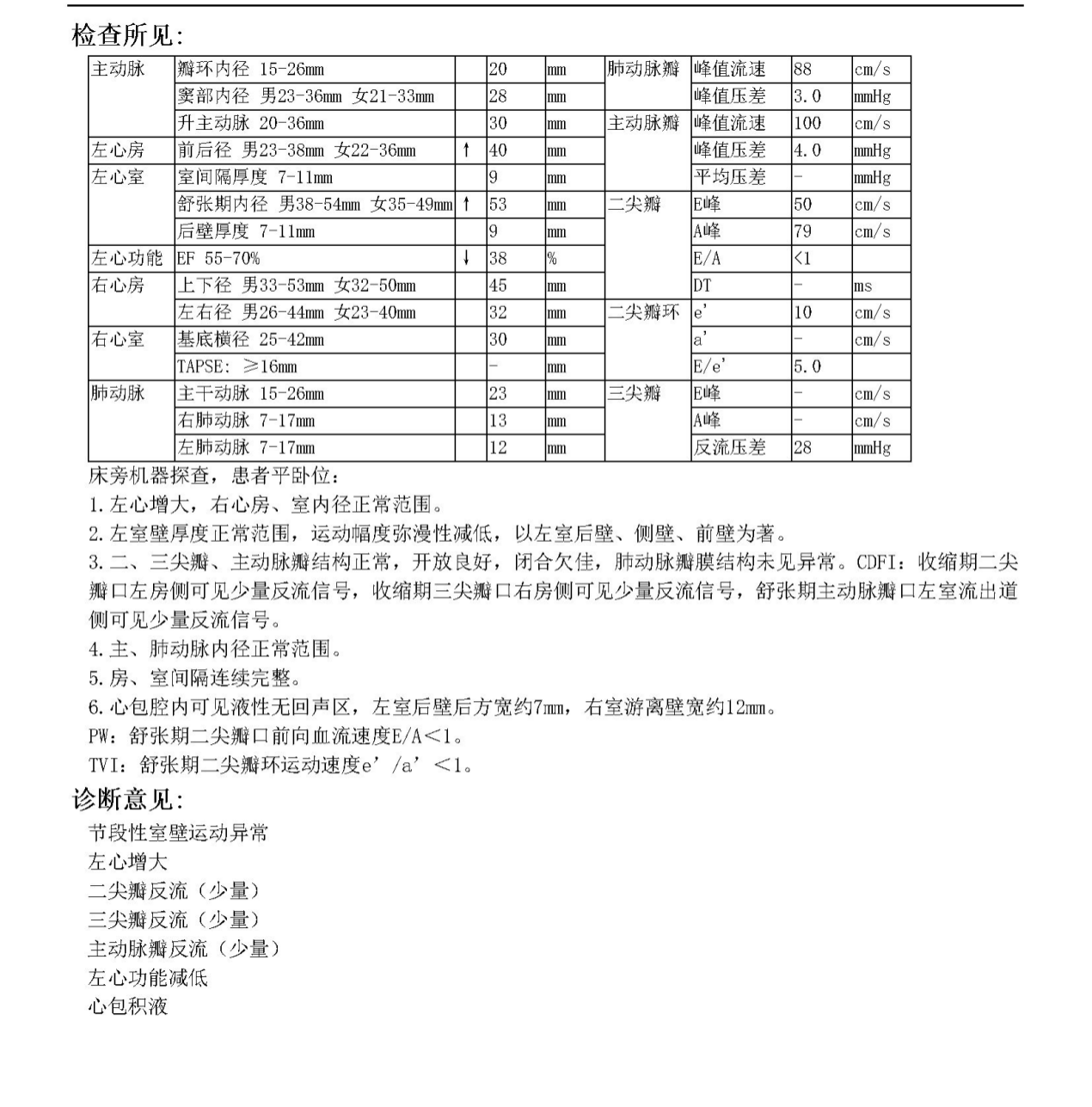

Supplement: Supplementary file 3 — Supplementary Data 2 [file 41746_2026_2648_MOESM3_ESM.zip › echocardiography_reports/281.png]

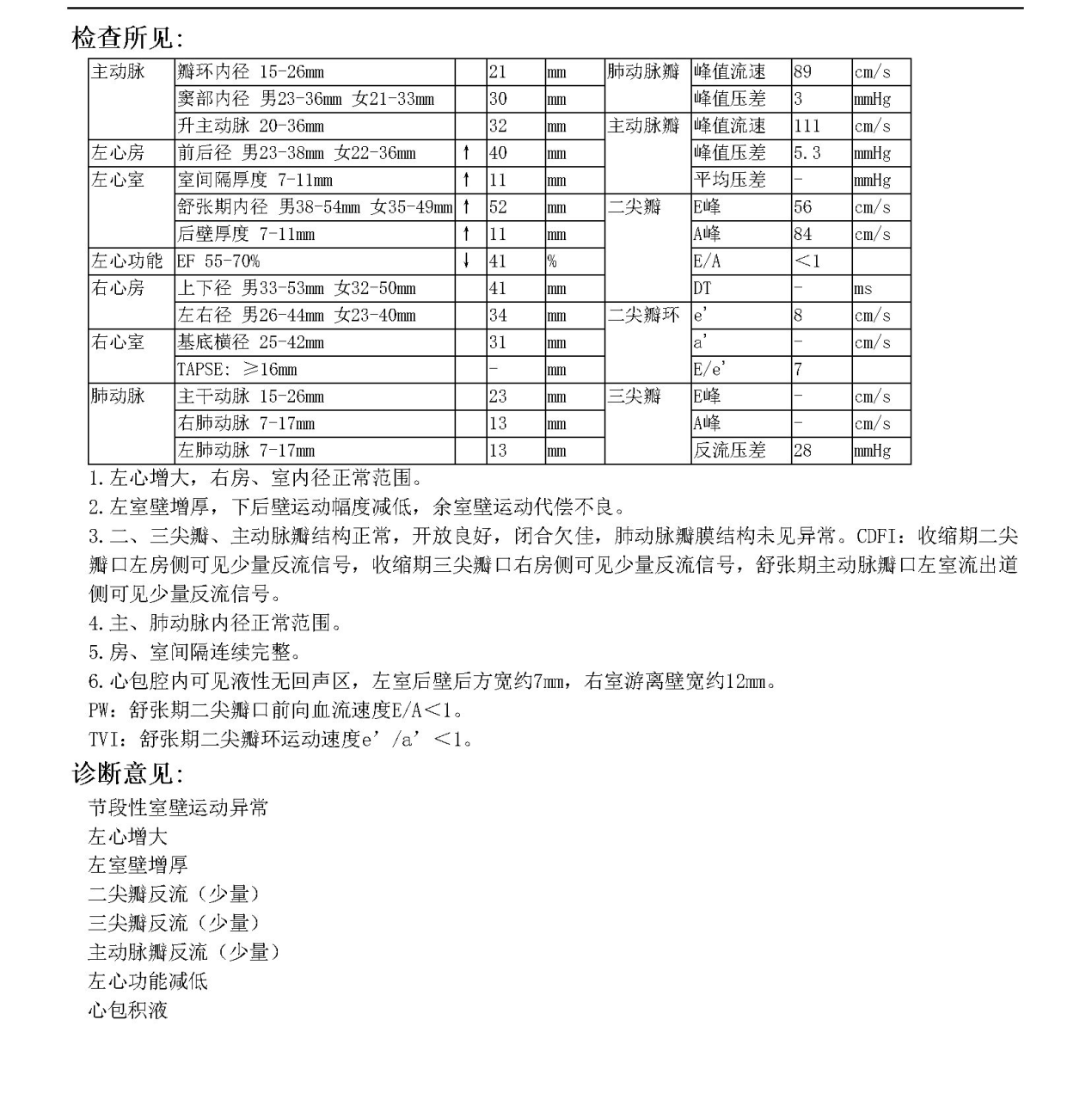

Supplement: Supplementary file 3 — Supplementary Data 2 [file 41746_2026_2648_MOESM3_ESM.zip › echocardiography_reports/282.png]

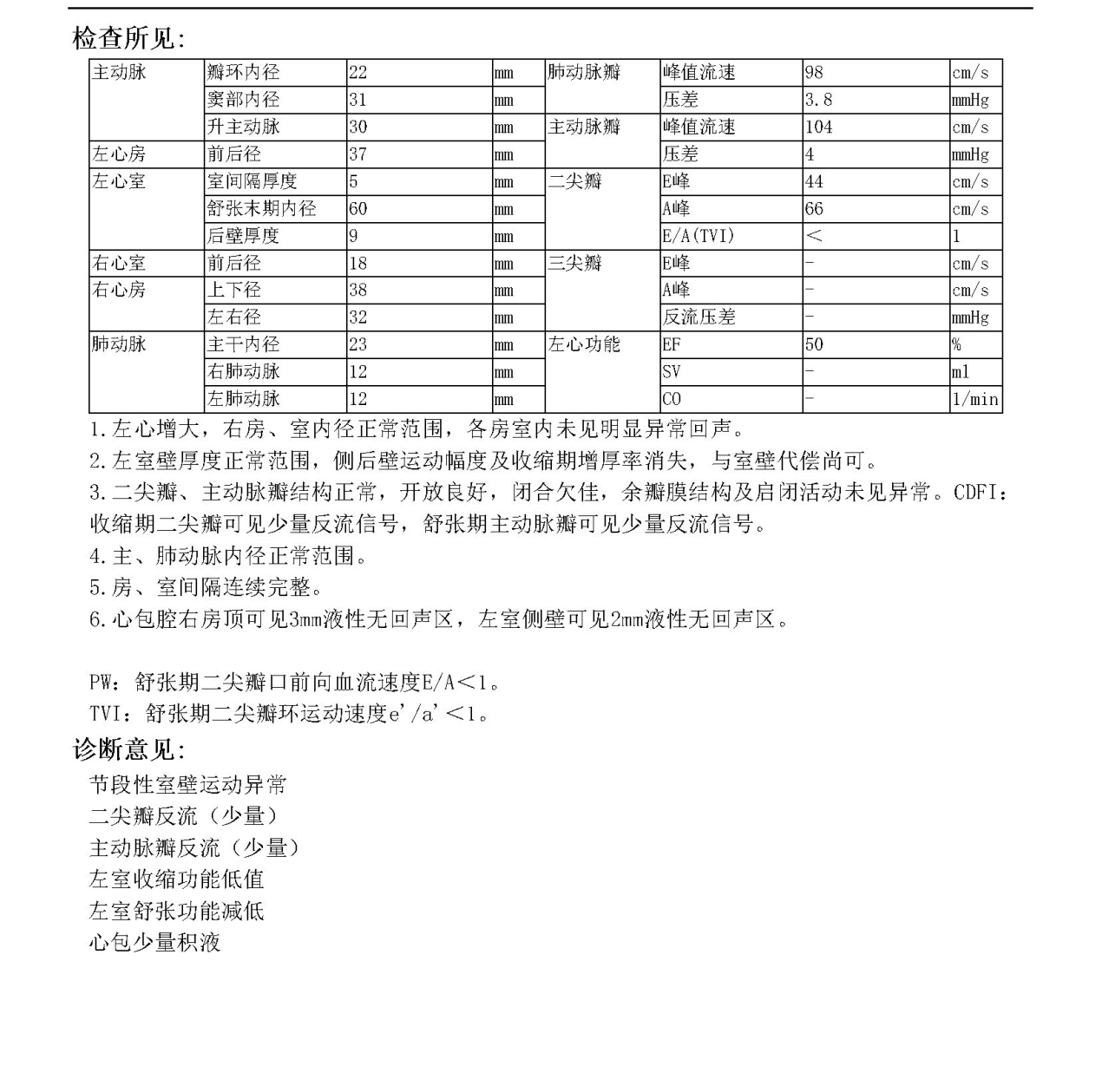

Supplement: Supplementary file 3 — Supplementary Data 2 [file 41746_2026_2648_MOESM3_ESM.zip › echocardiography_reports/283.png]

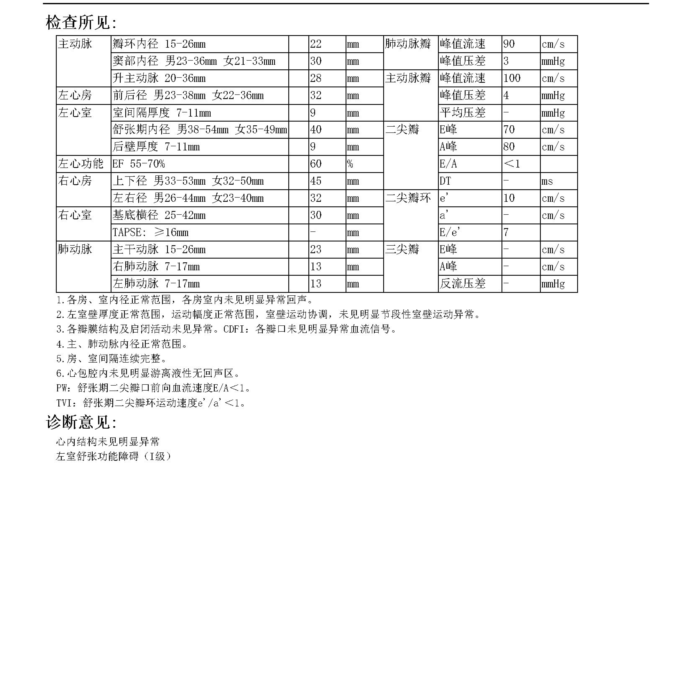

Supplement: Supplementary file 3 — Supplementary Data 2 [file 41746_2026_2648_MOESM3_ESM.zip › echocardiography_reports/284.png]

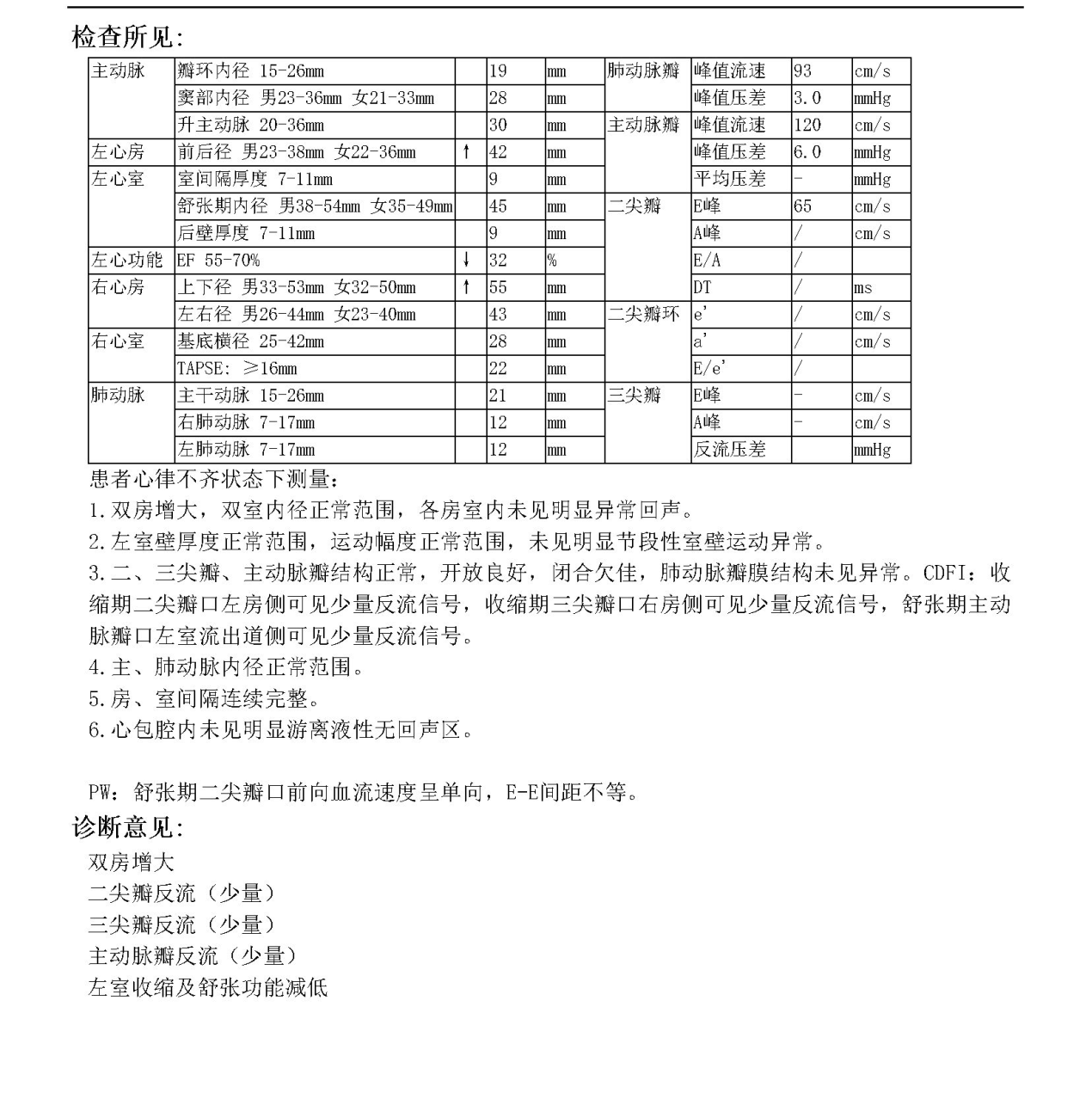

Supplement: Supplementary file 3 — Supplementary Data 2 [file 41746_2026_2648_MOESM3_ESM.zip › echocardiography_reports/285.png]

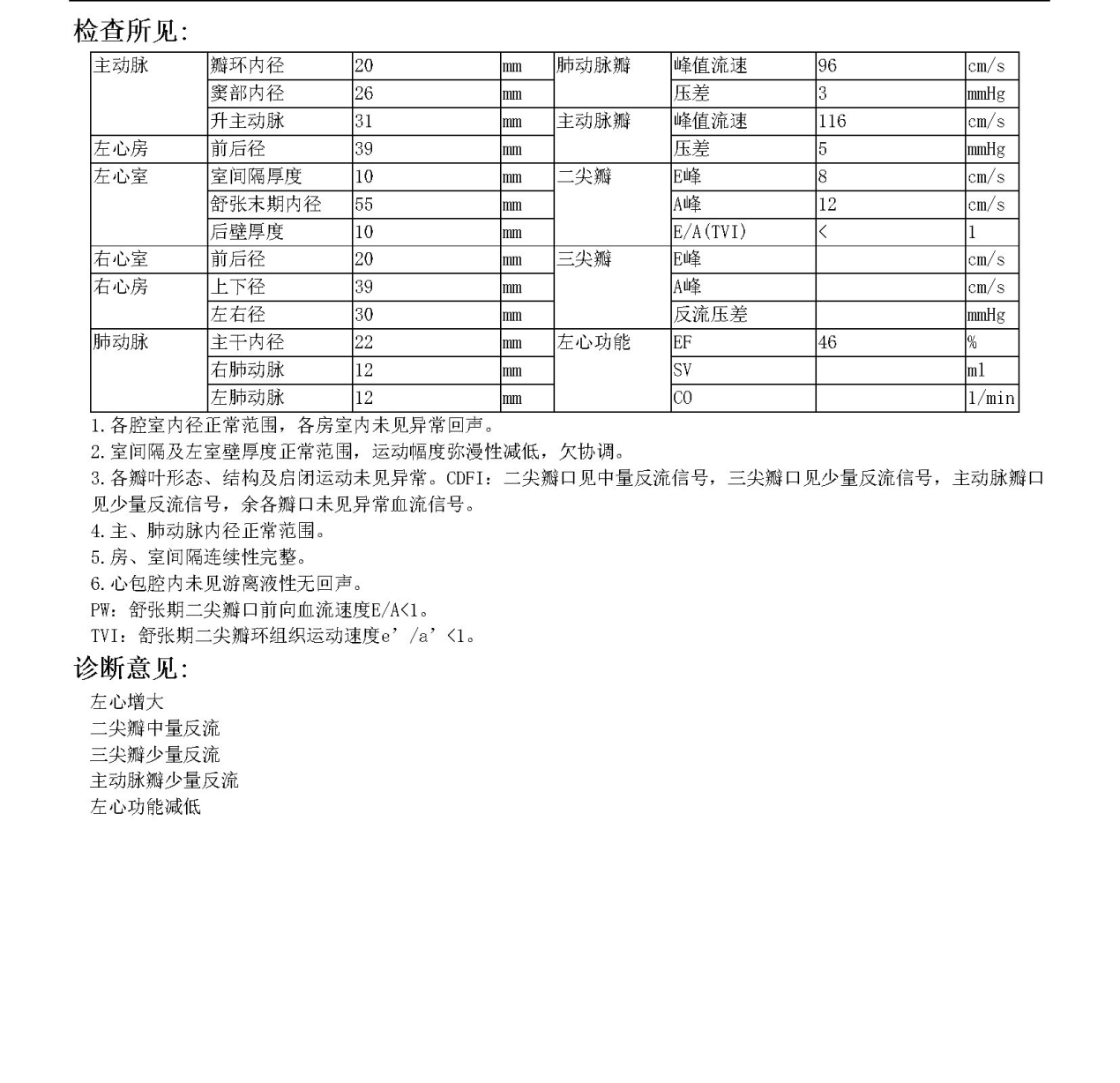

Supplement: Supplementary file 3 — Supplementary Data 2 [file 41746_2026_2648_MOESM3_ESM.zip › echocardiography_reports/286.png]

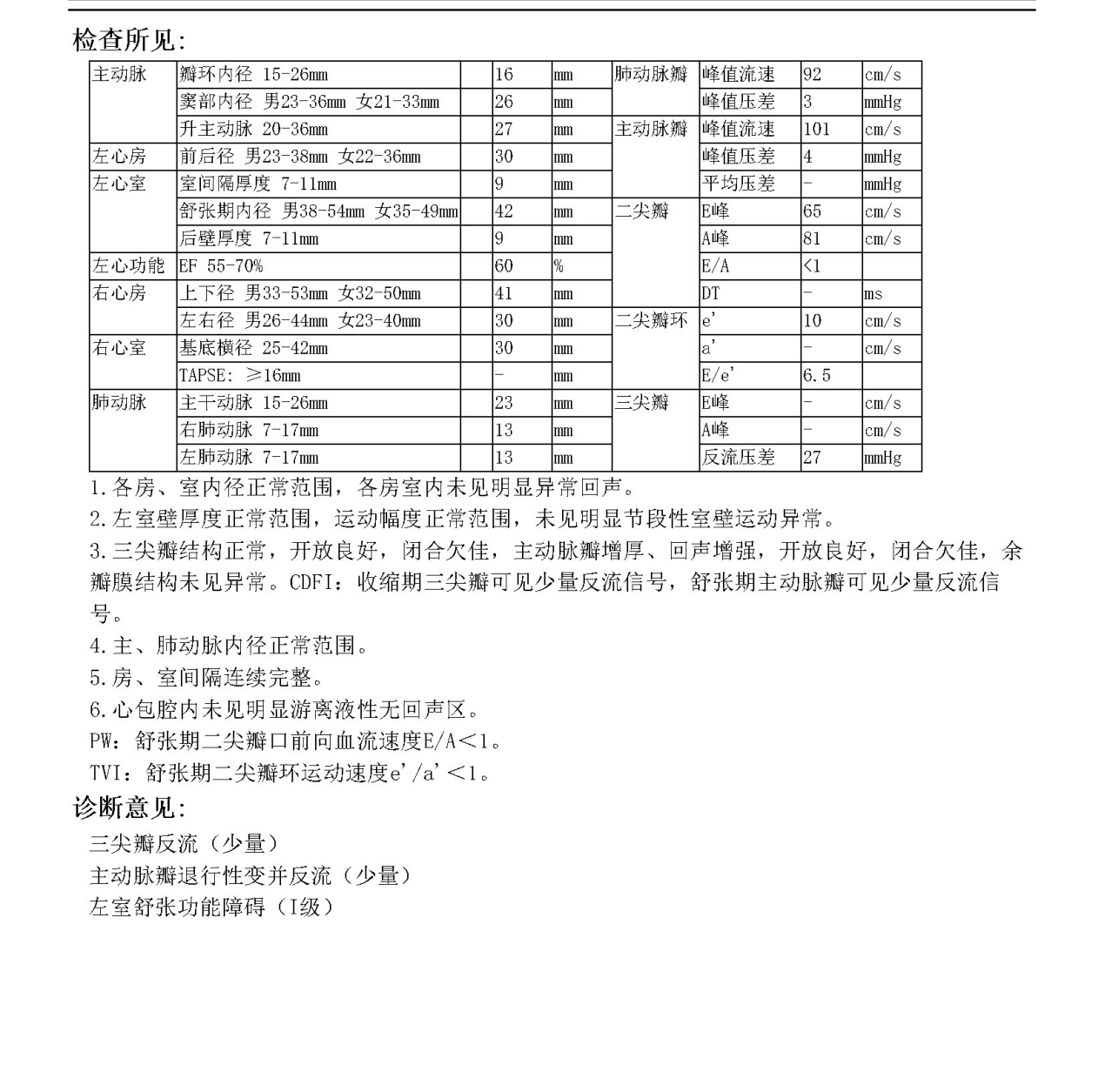

Supplement: Supplementary file 3 — Supplementary Data 2 [file 41746_2026_2648_MOESM3_ESM.zip › echocardiography_reports/287.png]

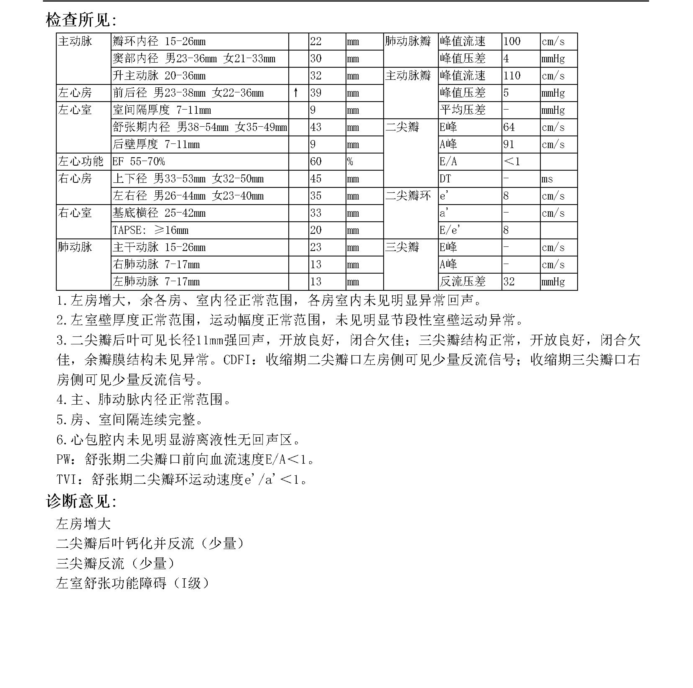

Supplement: Supplementary file 3 — Supplementary Data 2 [file 41746_2026_2648_MOESM3_ESM.zip › echocardiography_reports/288.png]

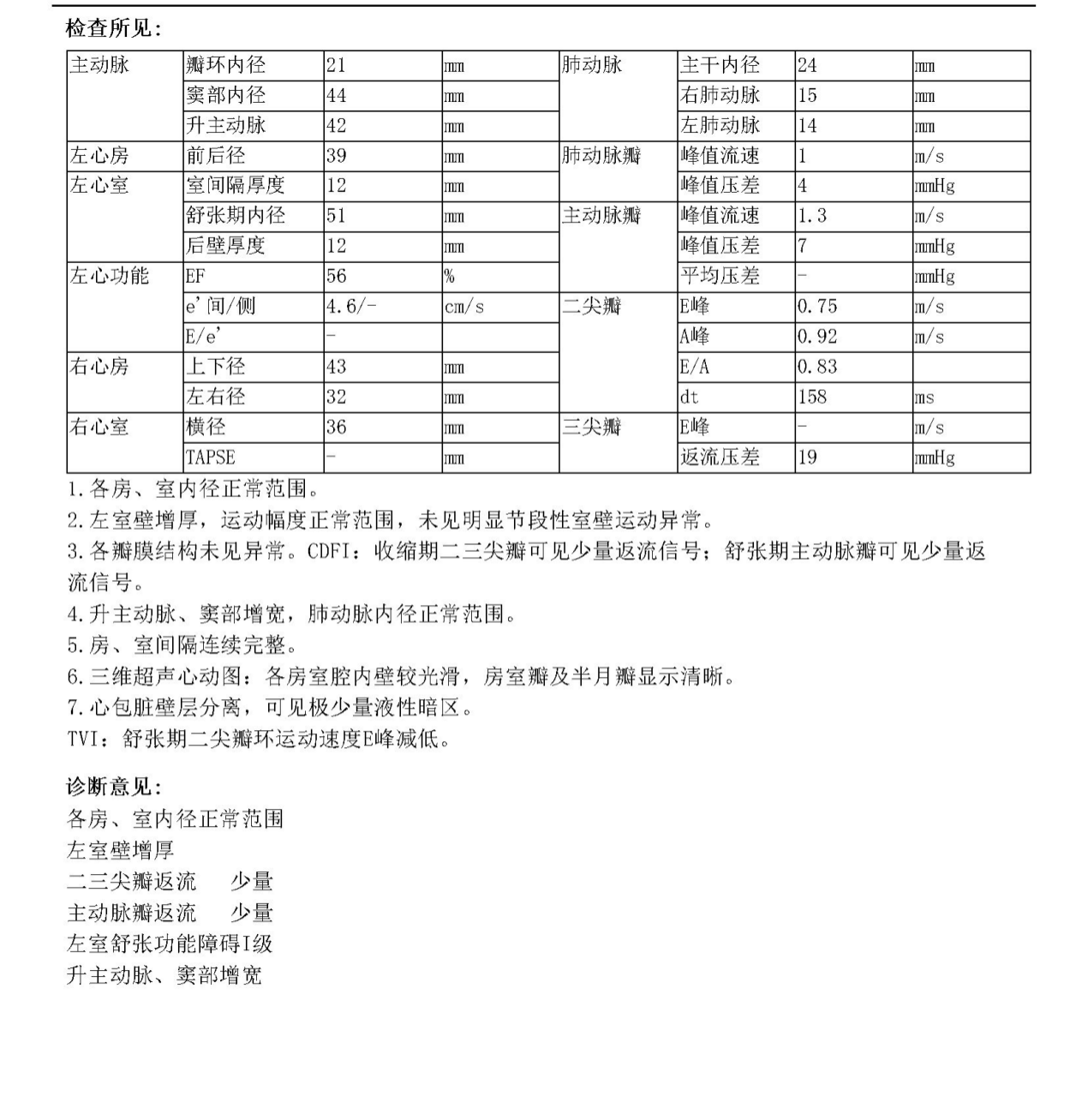

Supplement: Supplementary file 3 — Supplementary Data 2 [file 41746_2026_2648_MOESM3_ESM.zip › echocardiography_reports/289.png]

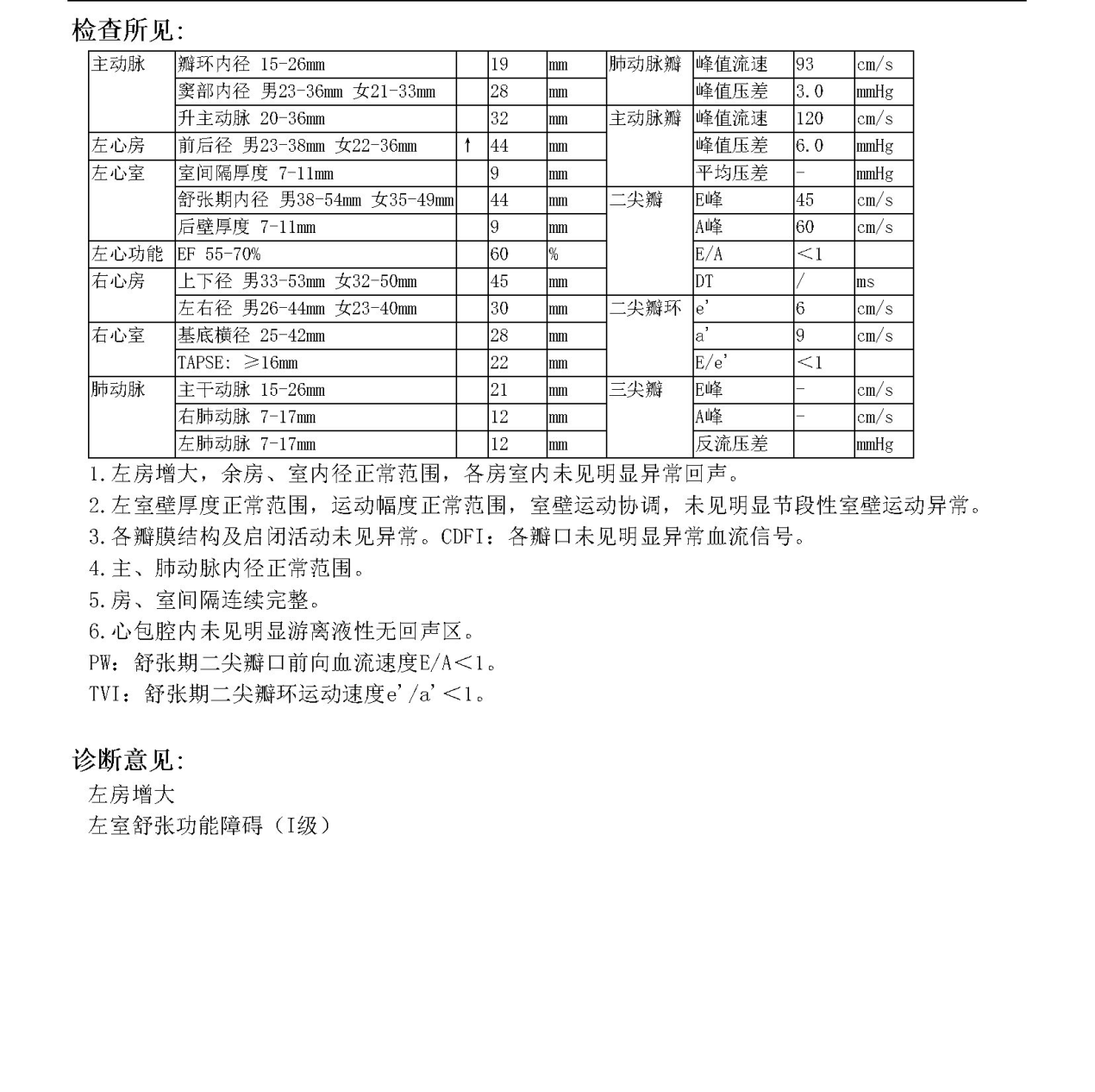

Supplement: Supplementary file 3 — Supplementary Data 2 [file 41746_2026_2648_MOESM3_ESM.zip › echocardiography_reports/290.png]

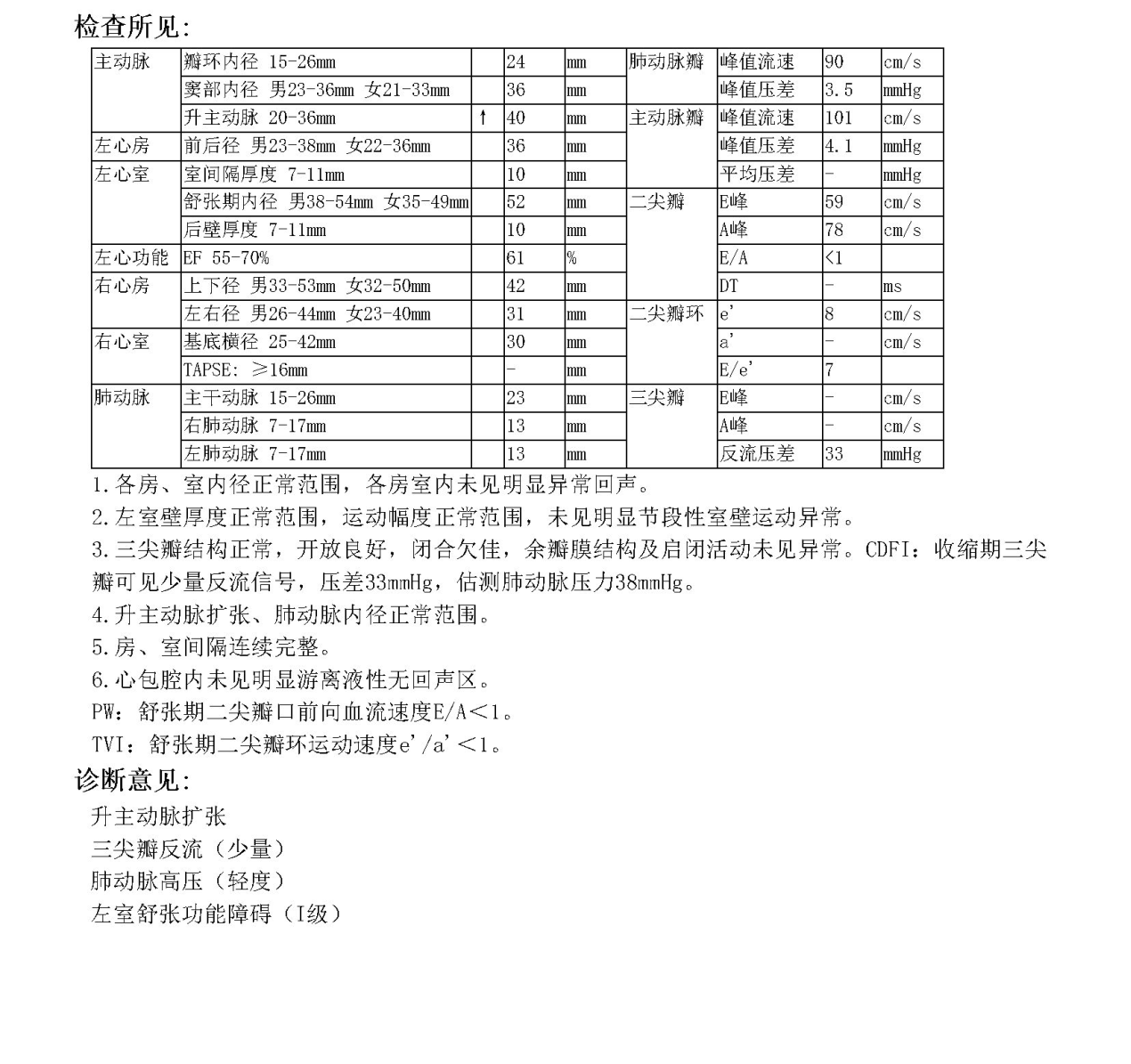

Supplement: Supplementary file 3 — Supplementary Data 2 [file 41746_2026_2648_MOESM3_ESM.zip › echocardiography_reports/291.png]

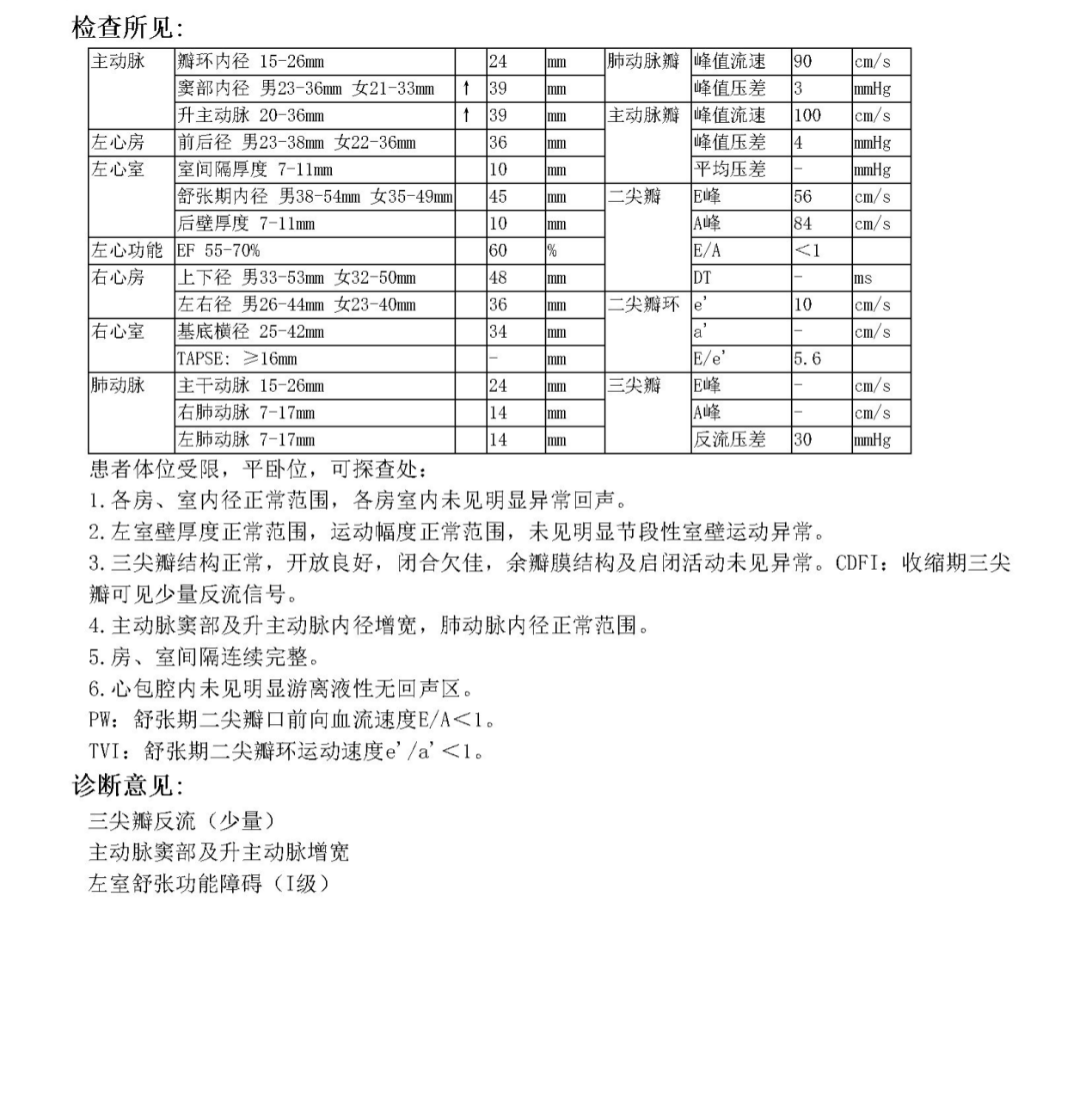

Supplement: Supplementary file 3 — Supplementary Data 2 [file 41746_2026_2648_MOESM3_ESM.zip › echocardiography_reports/292.png]

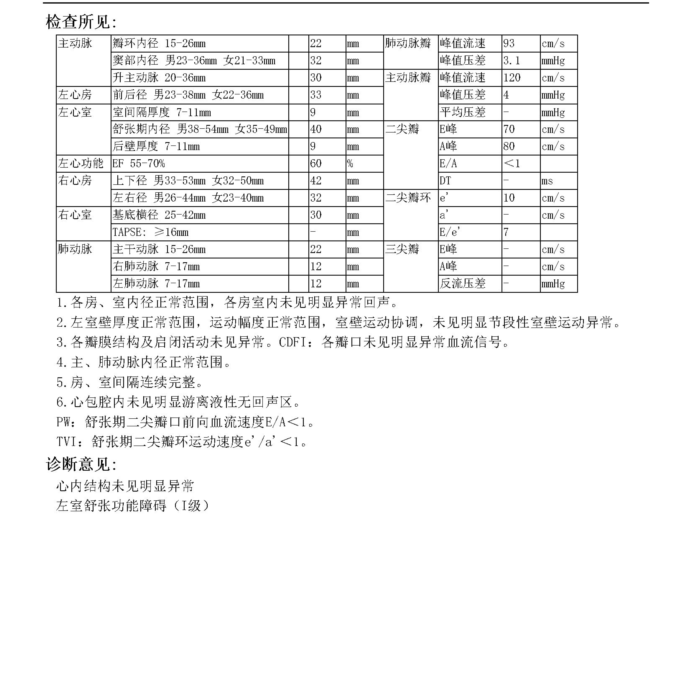

Supplement: Supplementary file 3 — Supplementary Data 2 [file 41746_2026_2648_MOESM3_ESM.zip › echocardiography_reports/293.png]

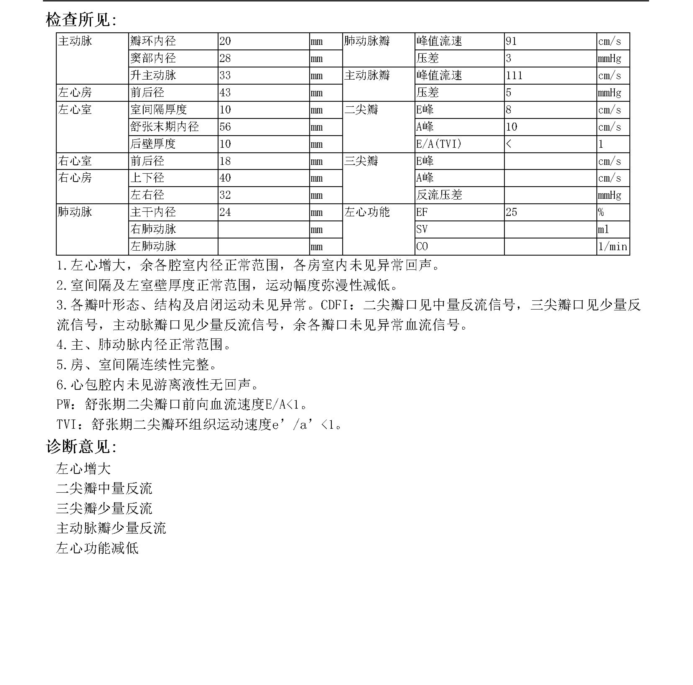

Supplement: Supplementary file 3 — Supplementary Data 2 [file 41746_2026_2648_MOESM3_ESM.zip › echocardiography_reports/294.png]

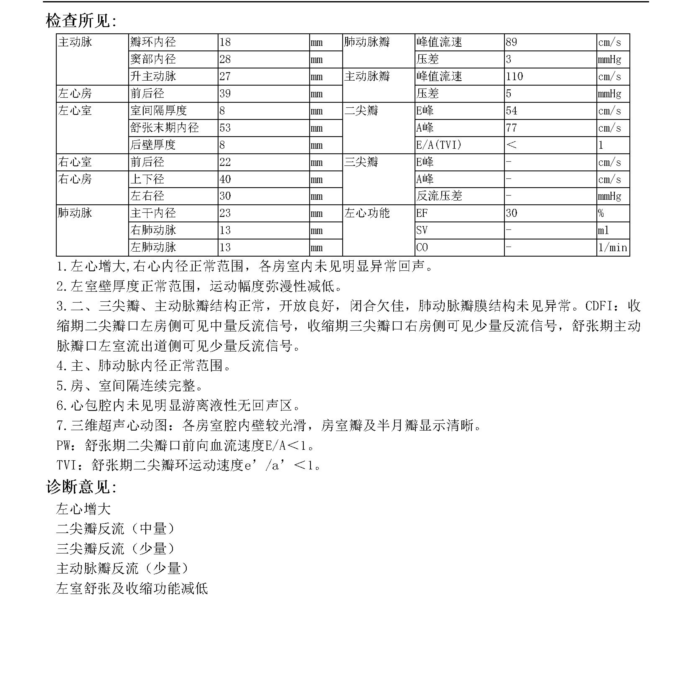

Supplement: Supplementary file 3 — Supplementary Data 2 [file 41746_2026_2648_MOESM3_ESM.zip › echocardiography_reports/295.png]

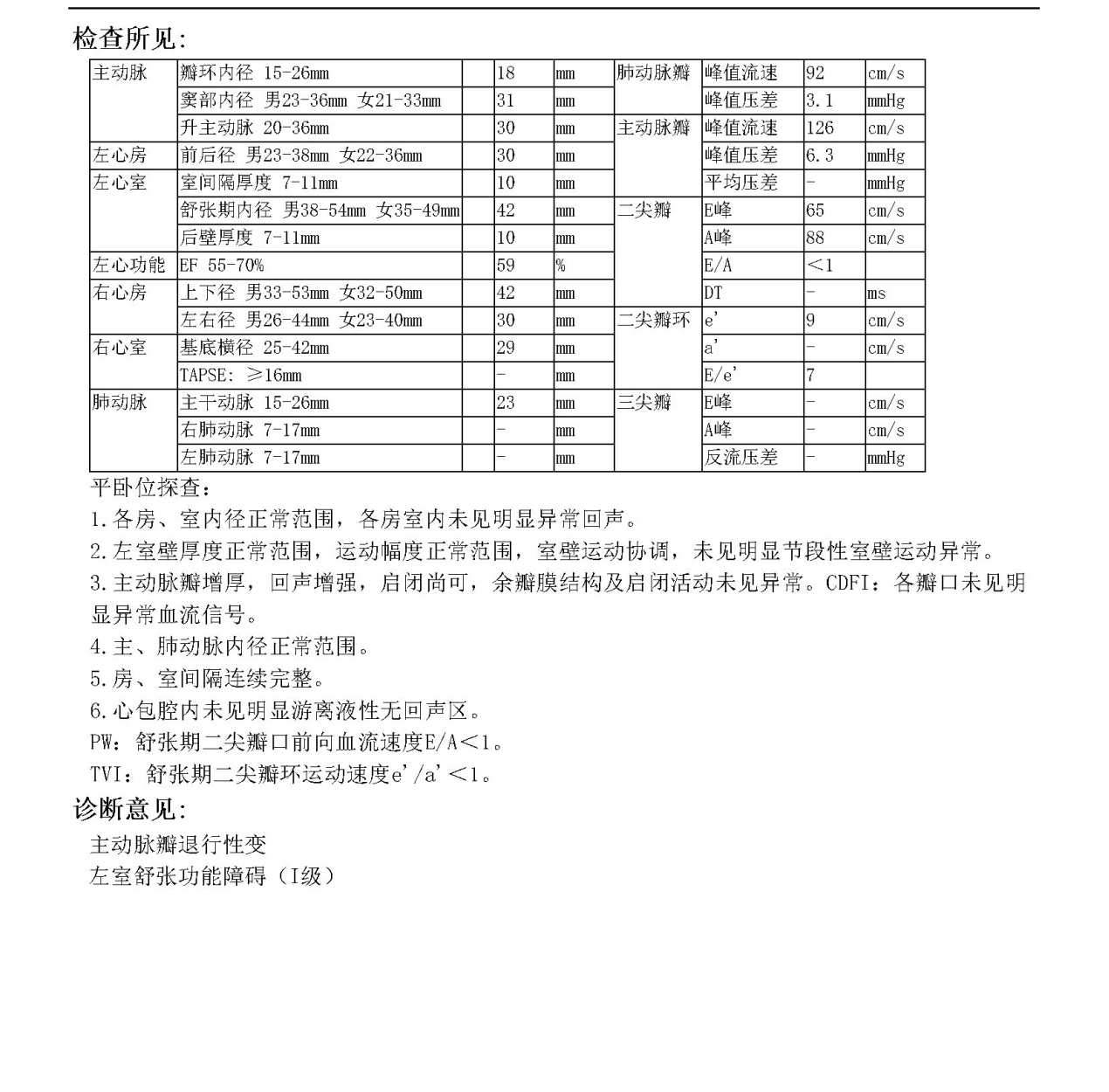

Supplement: Supplementary file 3 — Supplementary Data 2 [file 41746_2026_2648_MOESM3_ESM.zip › echocardiography_reports/296.png]

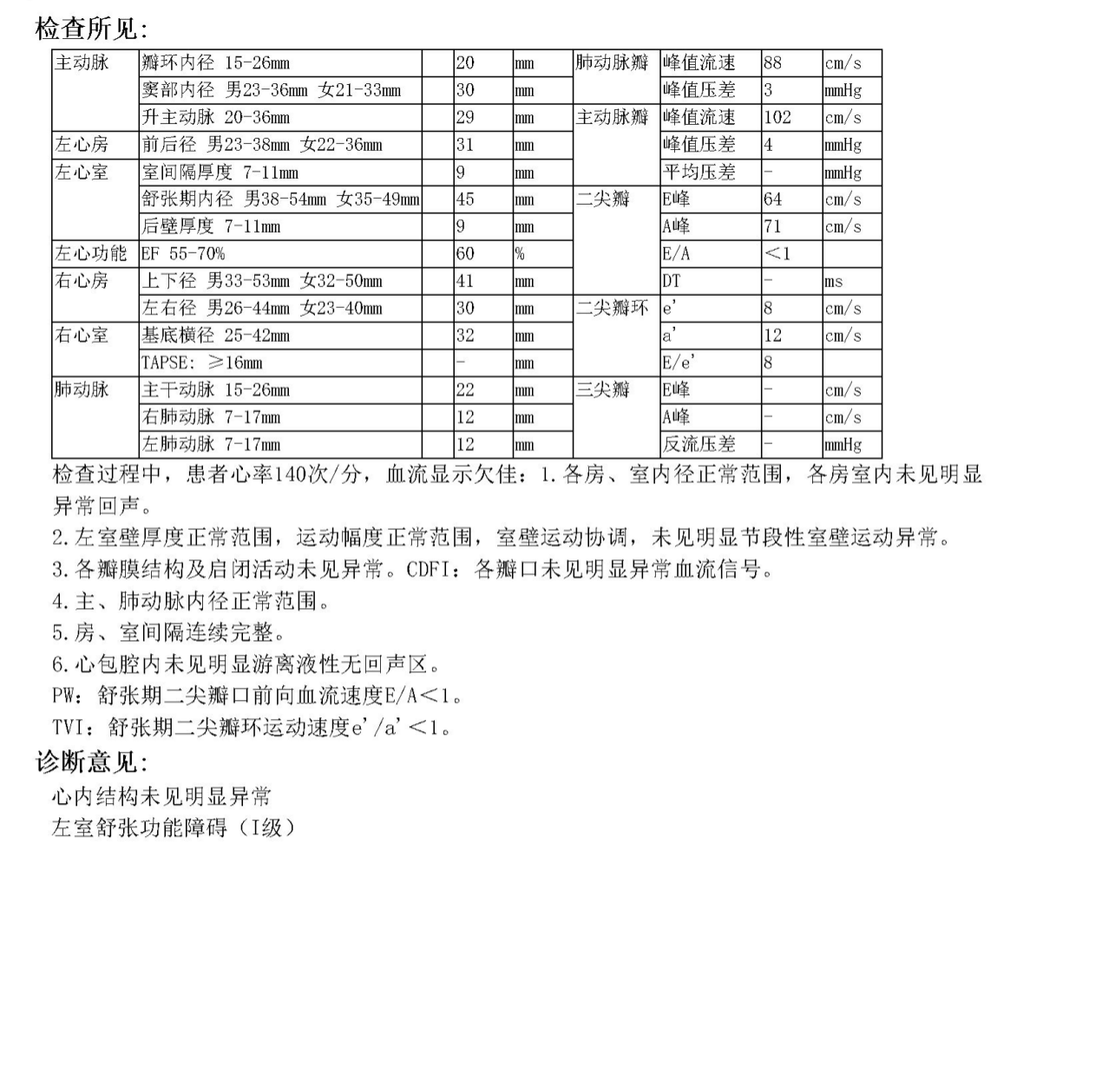

Supplement: Supplementary file 3 — Supplementary Data 2 [file 41746_2026_2648_MOESM3_ESM.zip › echocardiography_reports/297.png]

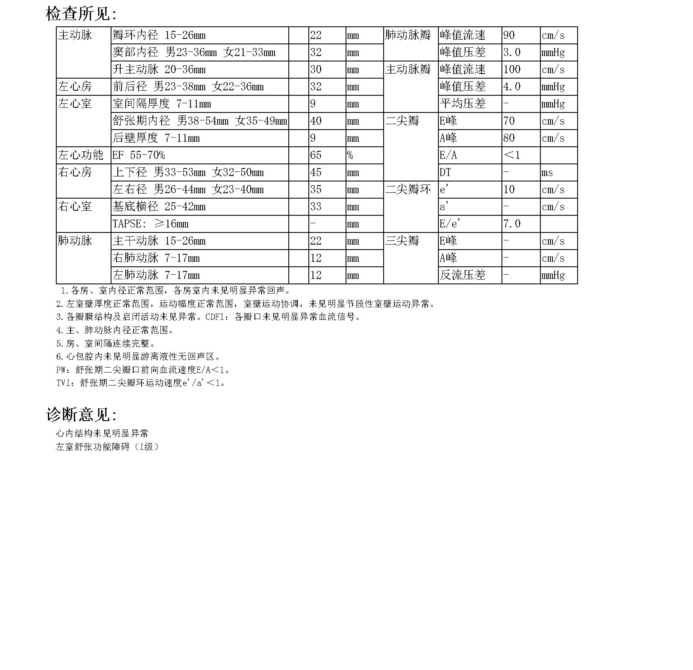

Supplement: Supplementary file 3 — Supplementary Data 2 [file 41746_2026_2648_MOESM3_ESM.zip › echocardiography_reports/298.png]

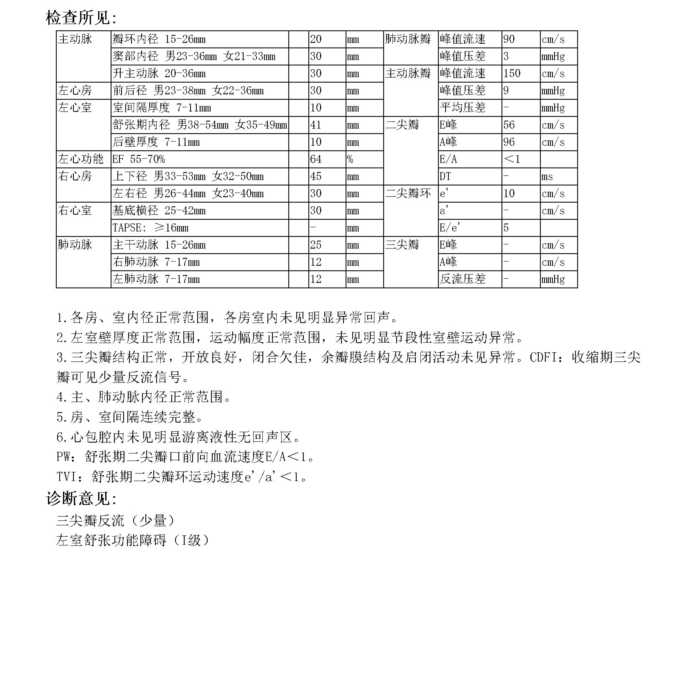

Supplement: Supplementary file 3 — Supplementary Data 2 [file 41746_2026_2648_MOESM3_ESM.zip › echocardiography_reports/299.png]

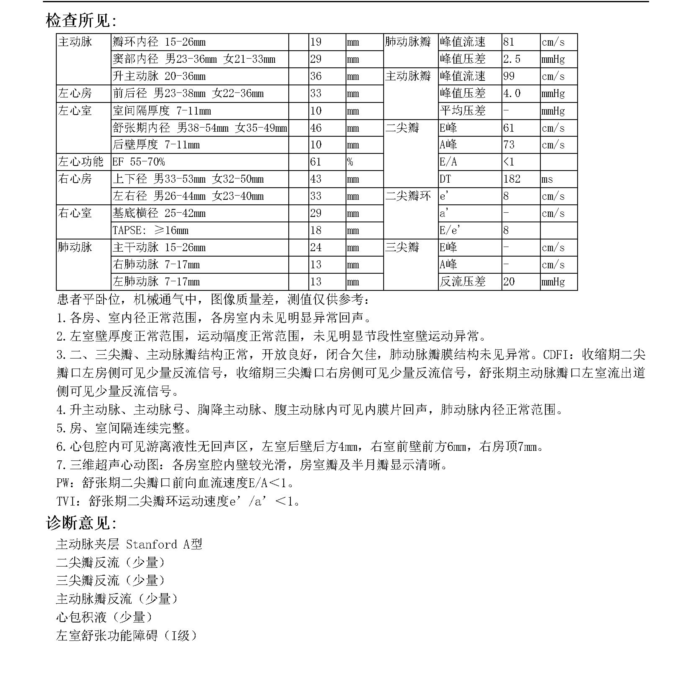

Supplement: Supplementary file 3 — Supplementary Data 2 [file 41746_2026_2648_MOESM3_ESM.zip › echocardiography_reports/300.png]

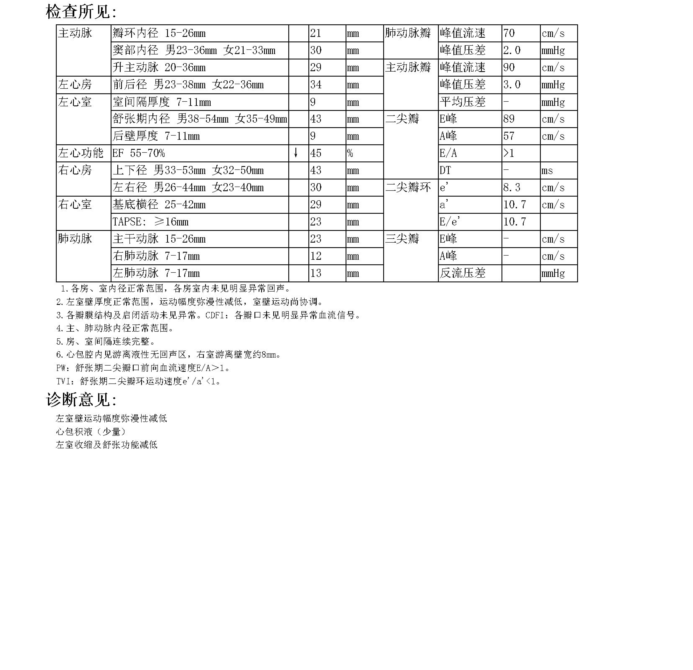

Supplement: Supplementary file 3 — Supplementary Data 2 [file 41746_2026_2648_MOESM3_ESM.zip › echocardiography_reports/301.png]

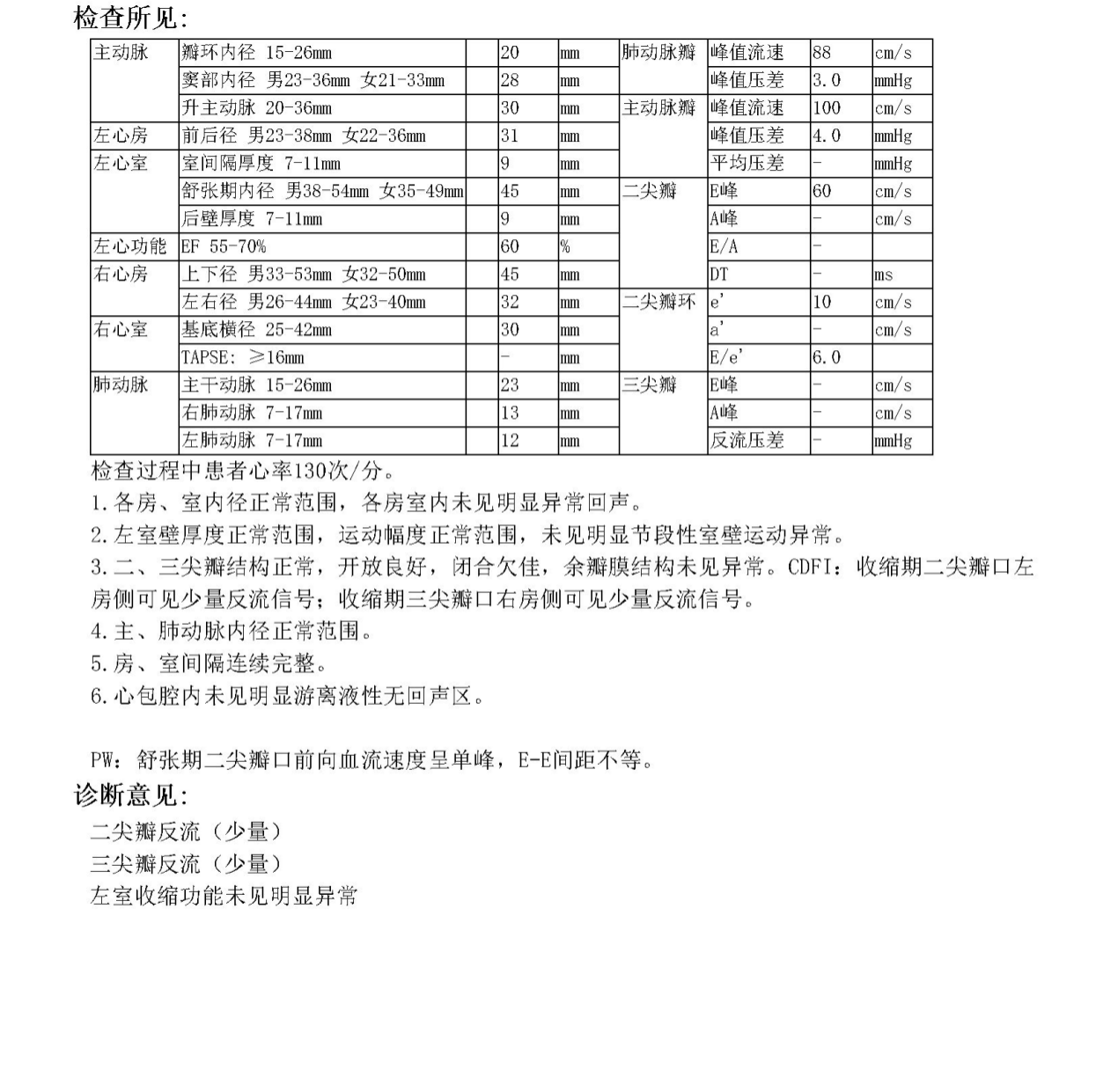

Supplement: Supplementary file 3 — Supplementary Data 2 [file 41746_2026_2648_MOESM3_ESM.zip › echocardiography_reports/302.png]

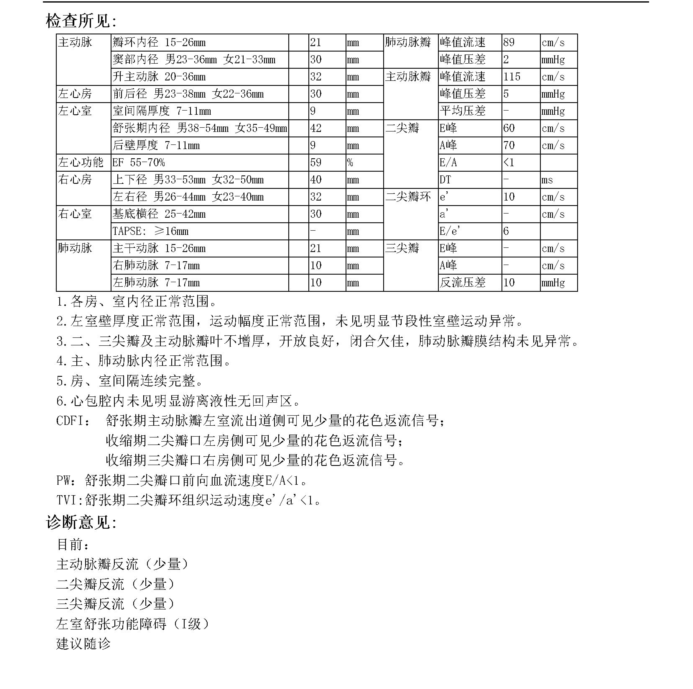

Supplement: Supplementary file 3 — Supplementary Data 2 [file 41746_2026_2648_MOESM3_ESM.zip › echocardiography_reports/303.png]

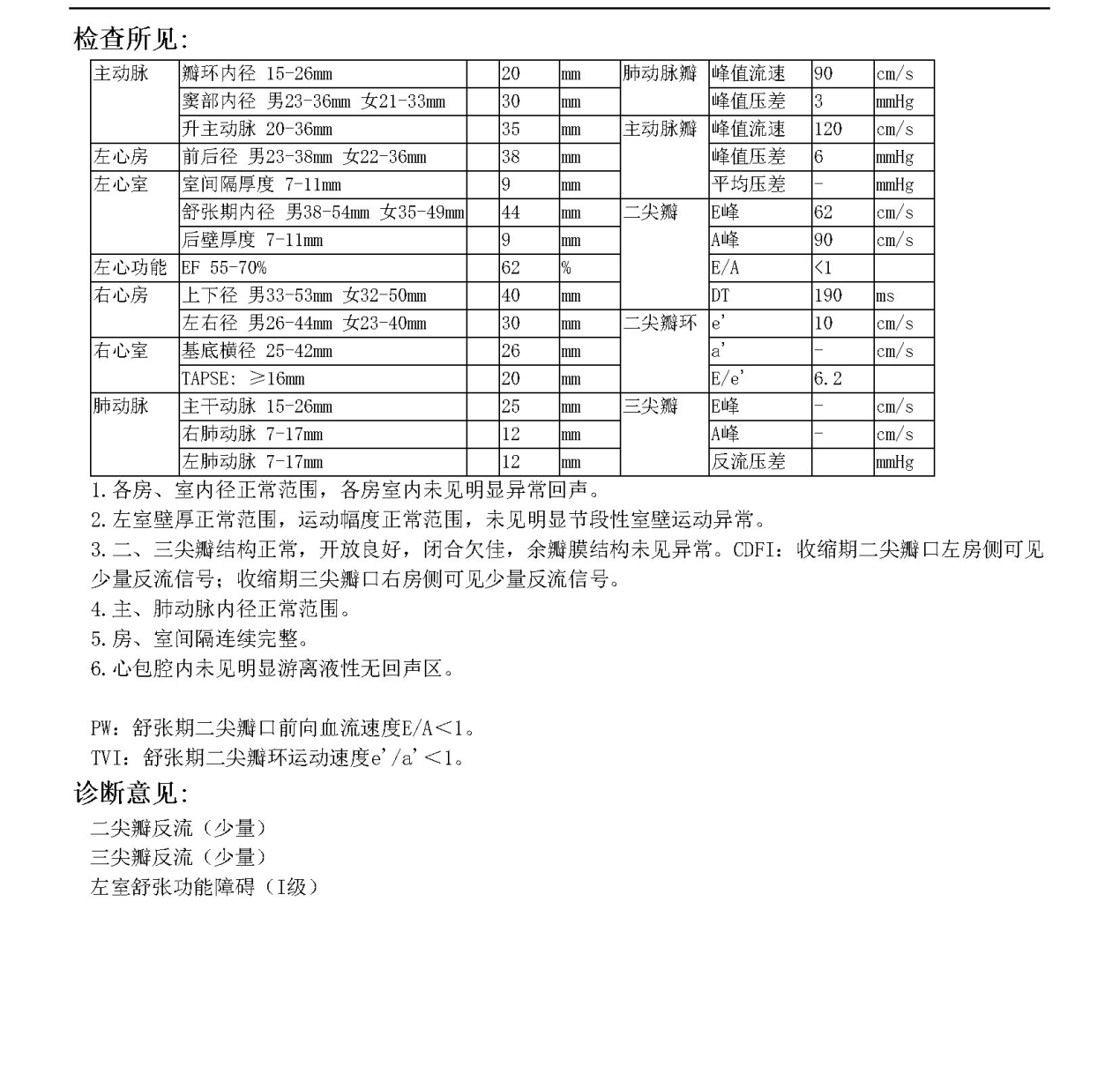

Supplement: Supplementary file 3 — Supplementary Data 2 [file 41746_2026_2648_MOESM3_ESM.zip › echocardiography_reports/304.png]

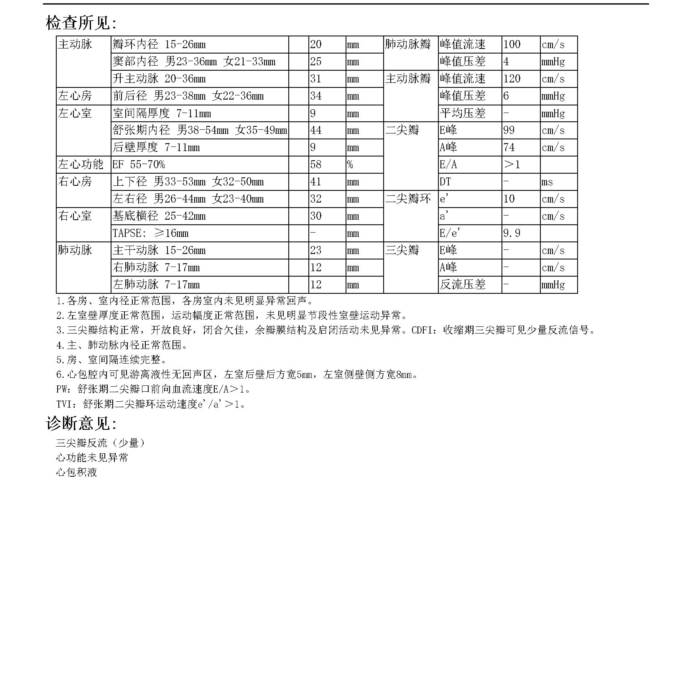

Supplement: Supplementary file 3 — Supplementary Data 2 [file 41746_2026_2648_MOESM3_ESM.zip › echocardiography_reports/305.png]

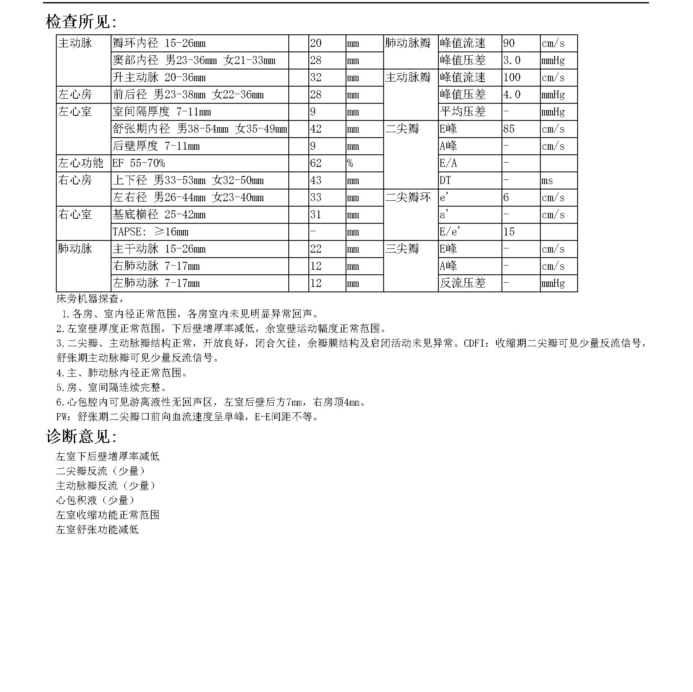

Supplement: Supplementary file 3 — Supplementary Data 2 [file 41746_2026_2648_MOESM3_ESM.zip › echocardiography_reports/306.png]

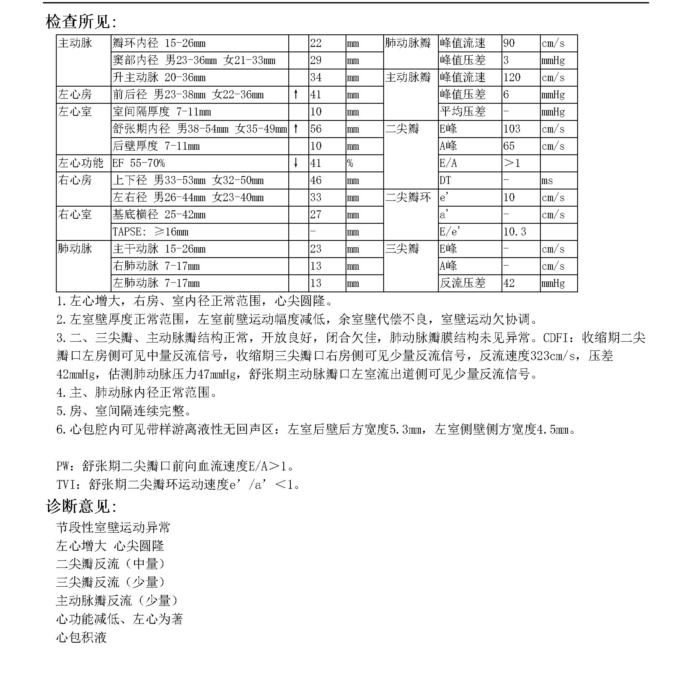

Supplement: Supplementary file 3 — Supplementary Data 2 [file 41746_2026_2648_MOESM3_ESM.zip › echocardiography_reports/307.png]

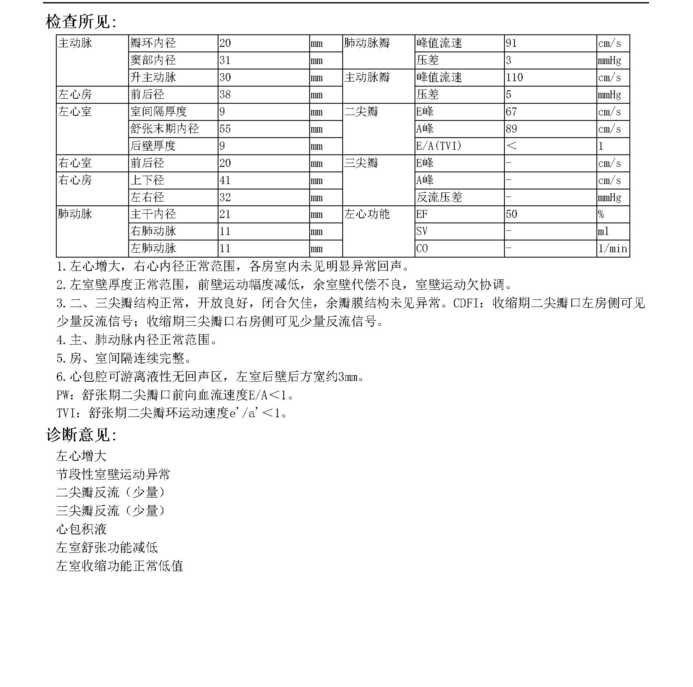

Supplement: Supplementary file 3 — Supplementary Data 2 [file 41746_2026_2648_MOESM3_ESM.zip › echocardiography_reports/308.png]

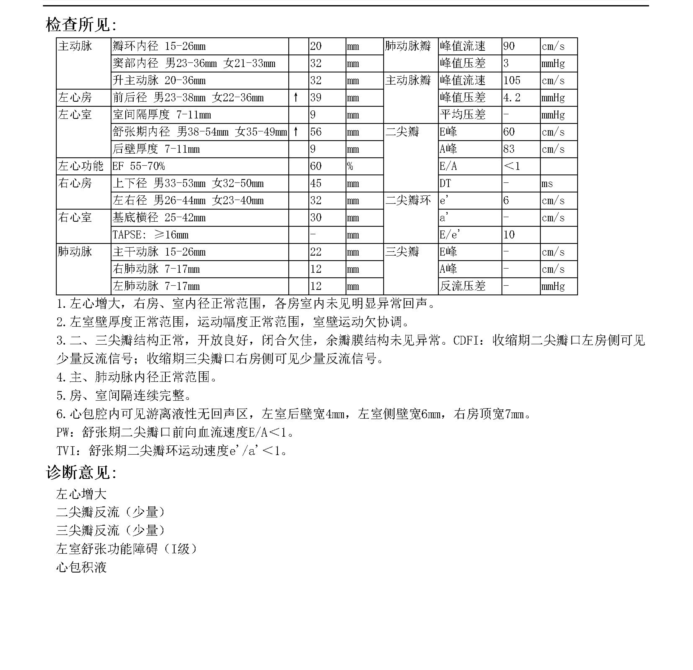

Supplement: Supplementary file 3 — Supplementary Data 2 [file 41746_2026_2648_MOESM3_ESM.zip › echocardiography_reports/309.png]

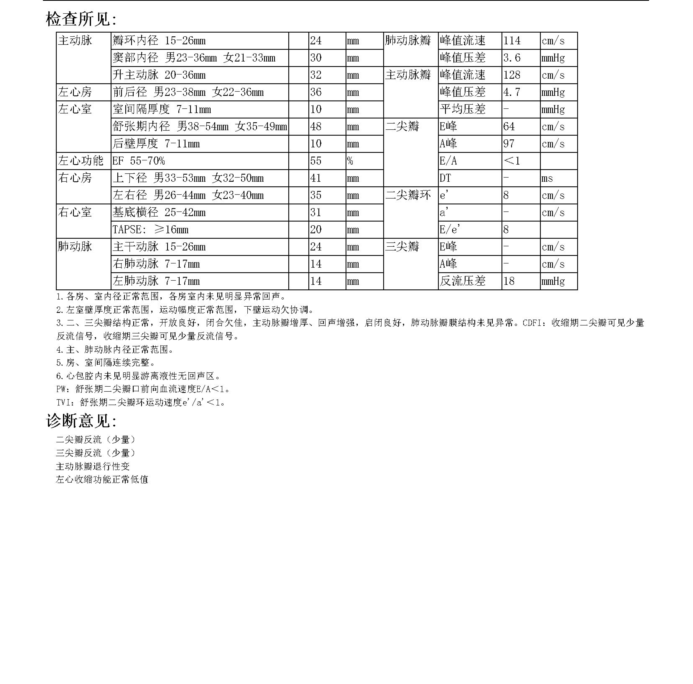

Supplement: Supplementary file 3 — Supplementary Data 2 [file 41746_2026_2648_MOESM3_ESM.zip › echocardiography_reports/310.png]

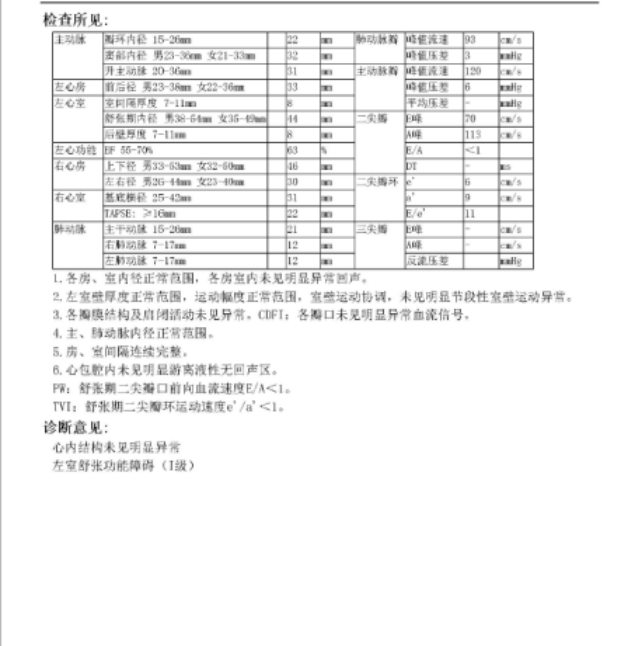

Supplement: Supplementary file 3 — Supplementary Data 2 [file 41746_2026_2648_MOESM3_ESM.zip › echocardiography_reports/311.png]

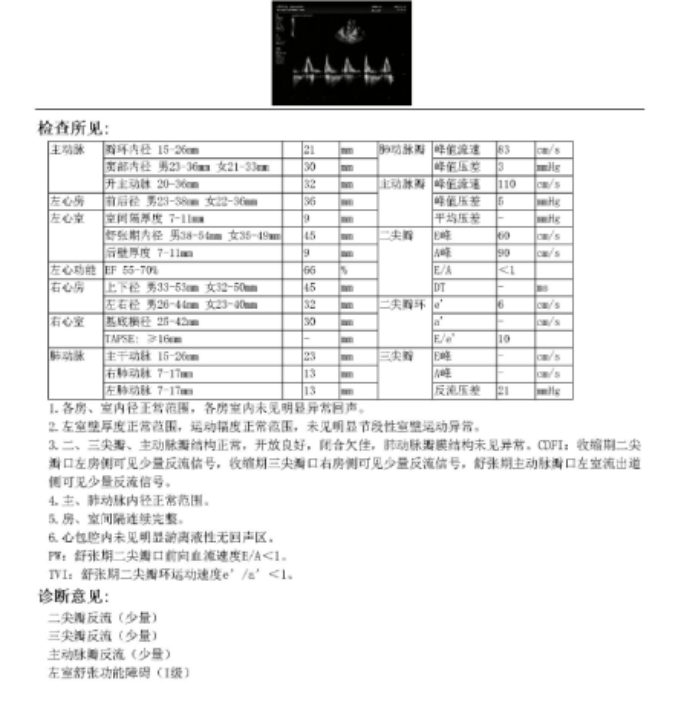

Supplement: Supplementary file 3 — Supplementary Data 2 [file 41746_2026_2648_MOESM3_ESM.zip › echocardiography_reports/312.png]

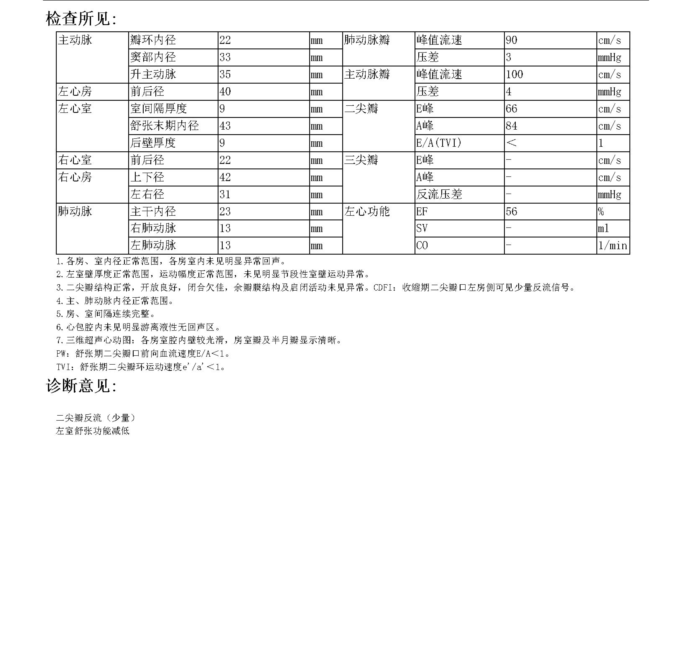

Supplement: Supplementary file 3 — Supplementary Data 2 [file 41746_2026_2648_MOESM3_ESM.zip › echocardiography_reports/313.png]

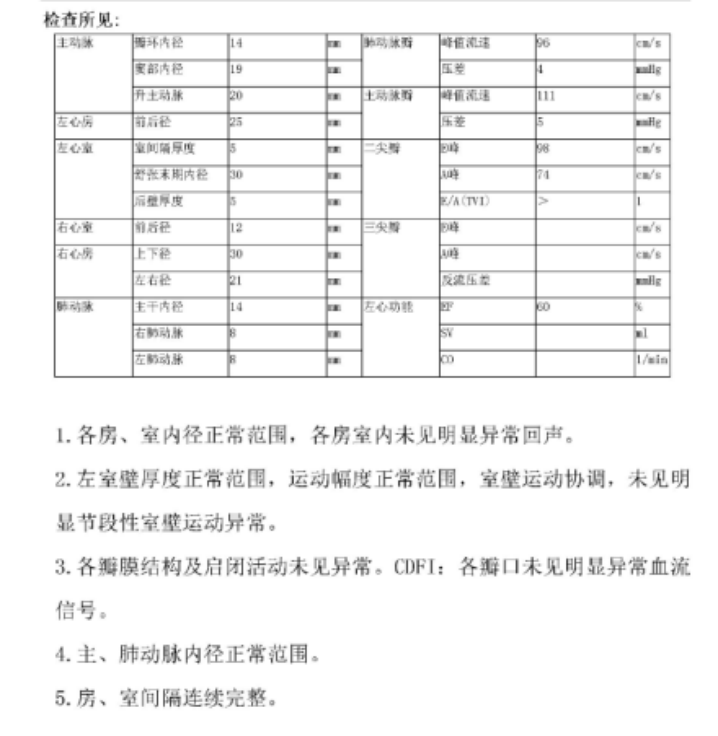

Supplement: Supplementary file 3 — Supplementary Data 2 [file 41746_2026_2648_MOESM3_ESM.zip › echocardiography_reports/314.png]

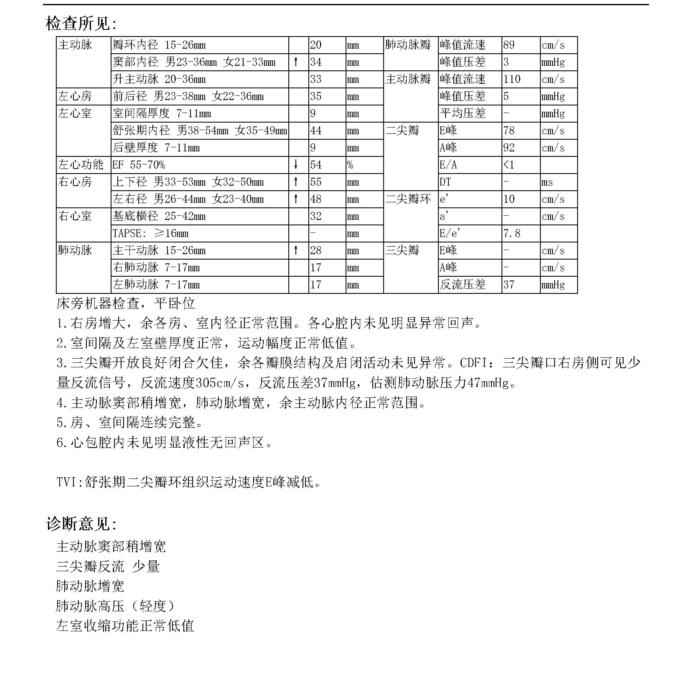

Supplement: Supplementary file 3 — Supplementary Data 2 [file 41746_2026_2648_MOESM3_ESM.zip › echocardiography_reports/315.png]

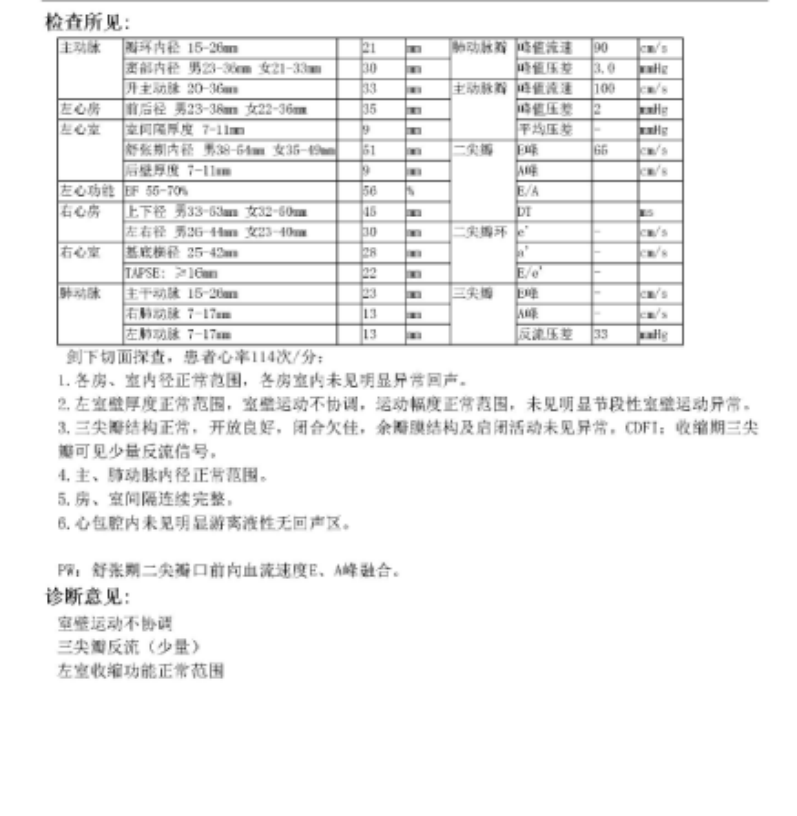

Supplement: Supplementary file 3 — Supplementary Data 2 [file 41746_2026_2648_MOESM3_ESM.zip › echocardiography_reports/316.png]

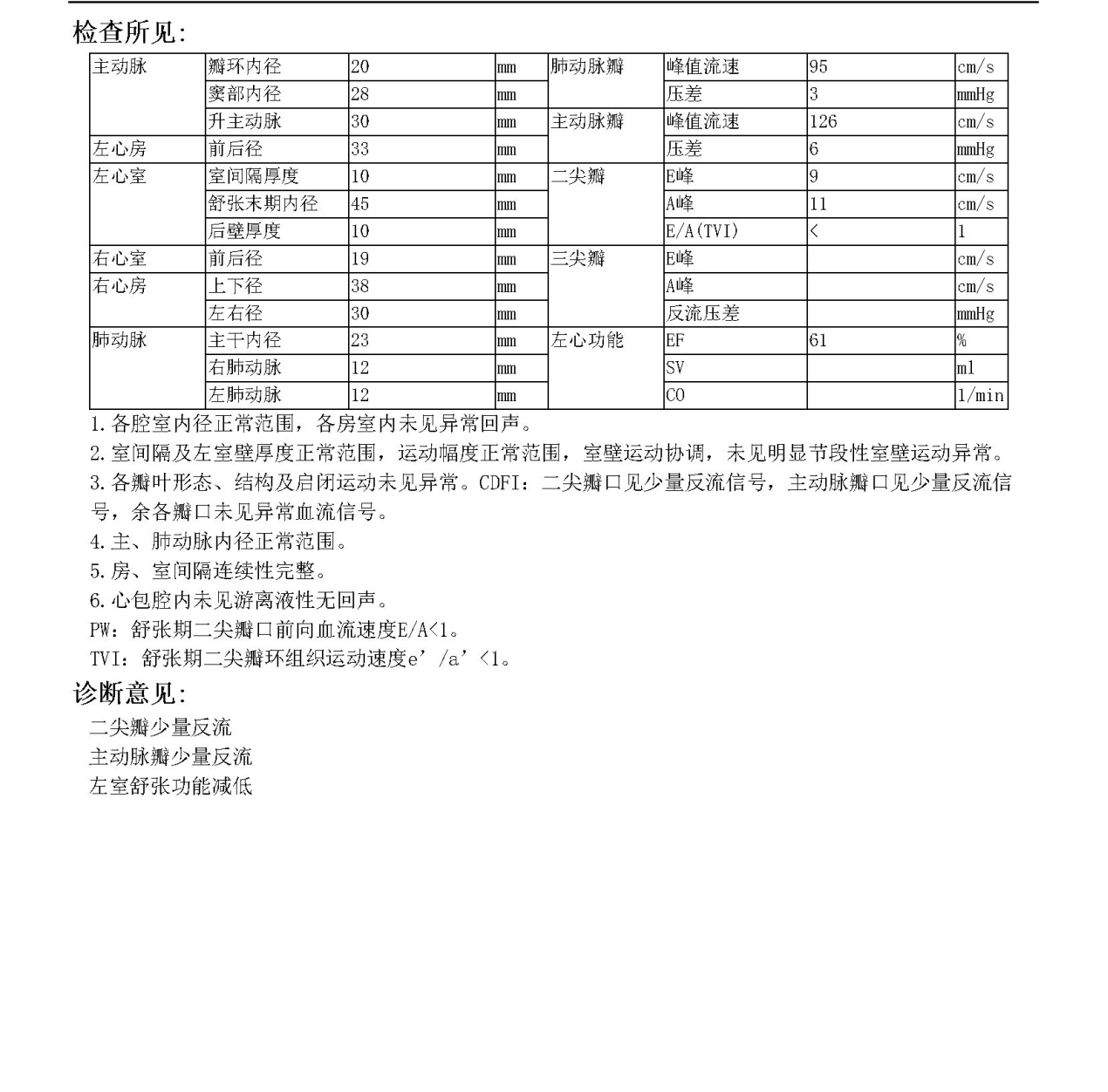

Supplement: Supplementary file 3 — Supplementary Data 2 [file 41746_2026_2648_MOESM3_ESM.zip › echocardiography_reports/317.png]

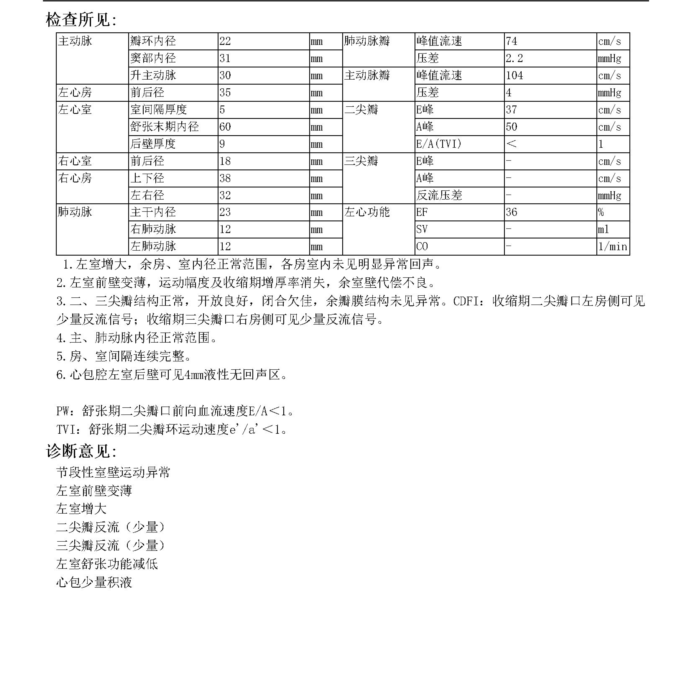

Supplement: Supplementary file 3 — Supplementary Data 2 [file 41746_2026_2648_MOESM3_ESM.zip › echocardiography_reports/318.png]

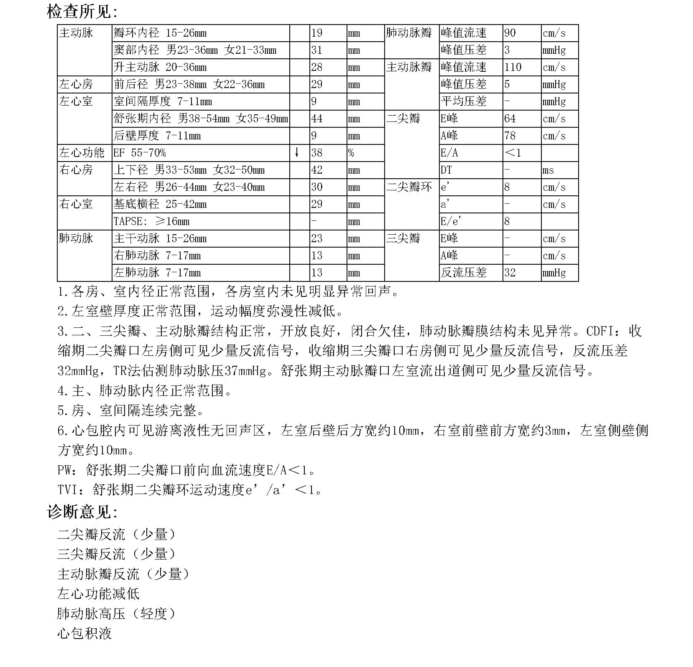

Supplement: Supplementary file 3 — Supplementary Data 2 [file 41746_2026_2648_MOESM3_ESM.zip › echocardiography_reports/319.png]
