# Supplementary figures and images for: CT-based AI system for quantitative and integrated management of acute respiratory distress syndrome in critical care (part 1 of 2)
Source: NPJ Digit Med. 2026 Apr 24;9:493. doi: 10.1038/s41746-026-02648-9 (PMC13315241; doi:10.1038/s41746-026-02648-9)

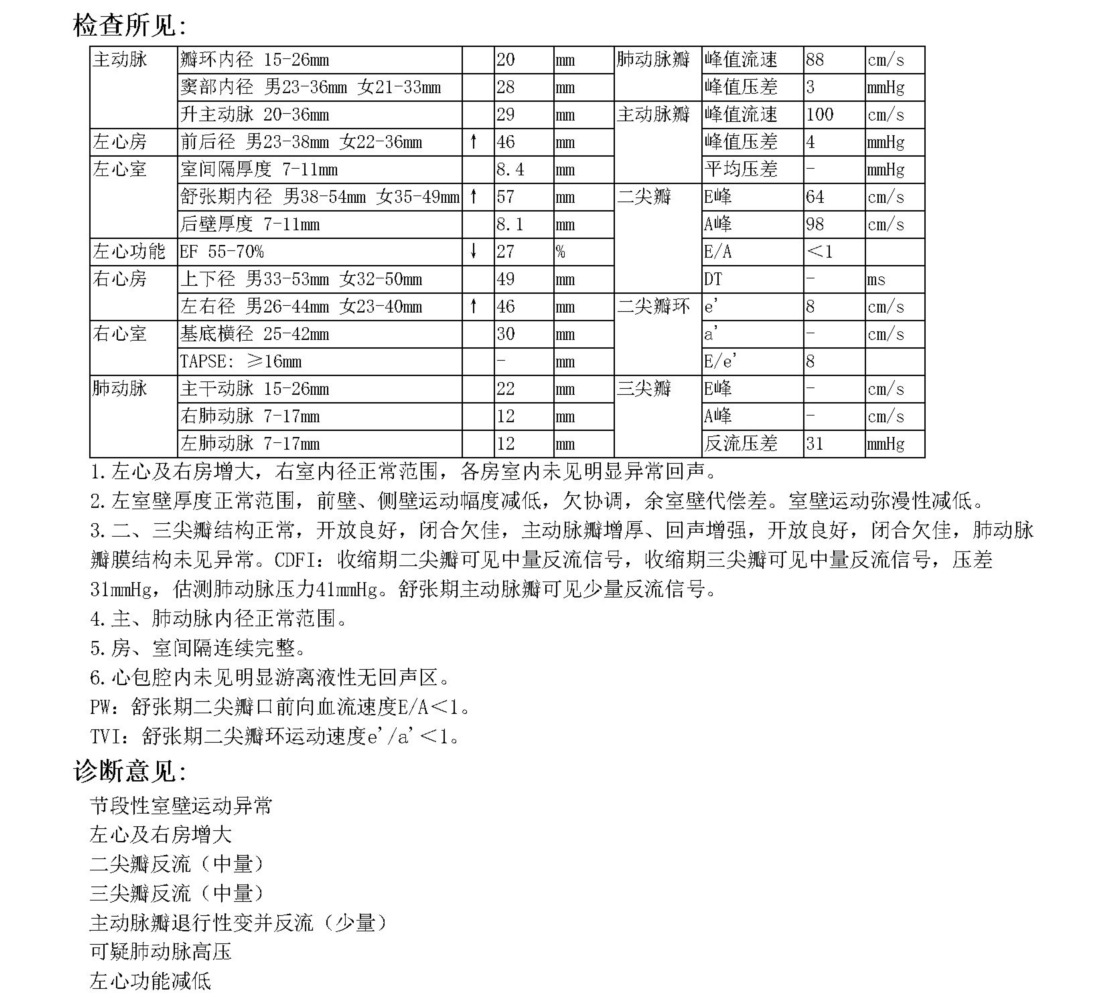

Supplement: Supplementary file 3 — Supplementary Data 2 [file 41746_2026_2648_MOESM3_ESM.zip › echocardiography_reports/120.png]

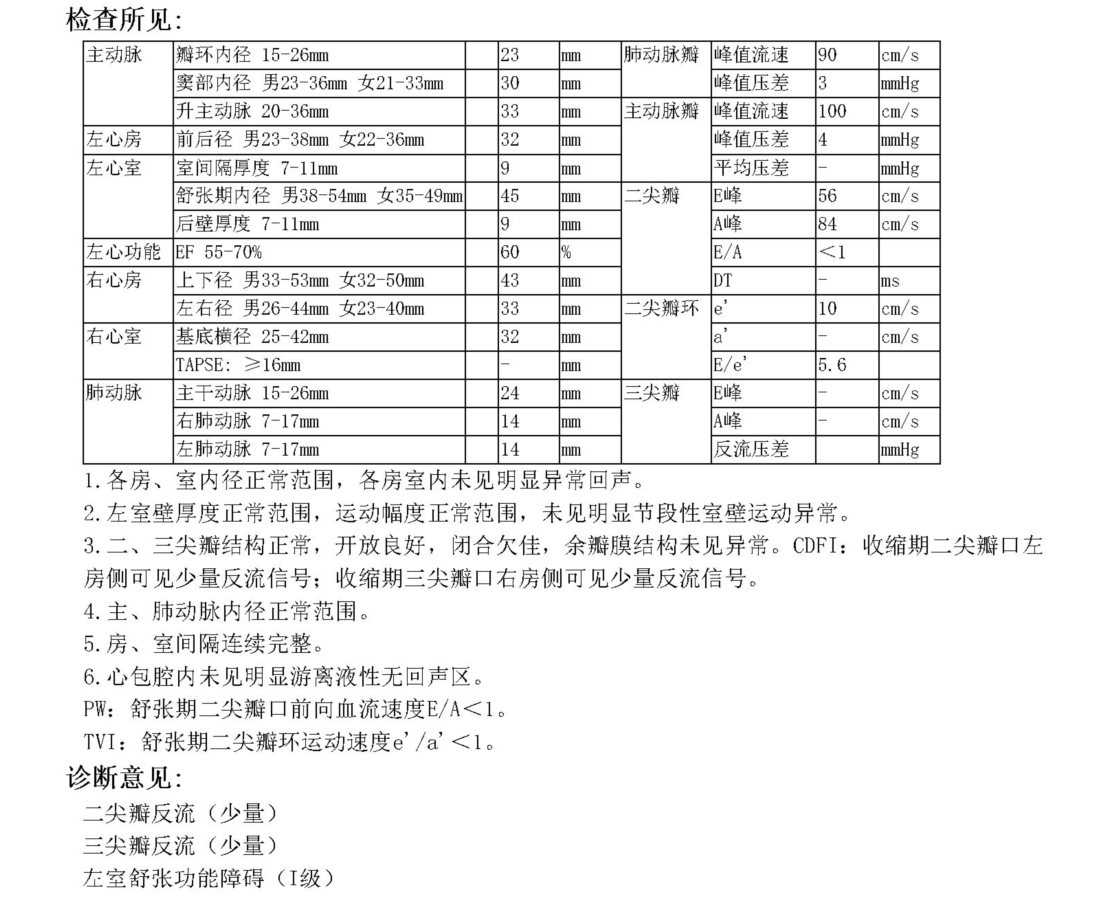

Supplement: Supplementary file 3 — Supplementary Data 2 [file 41746_2026_2648_MOESM3_ESM.zip › echocardiography_reports/121.png]

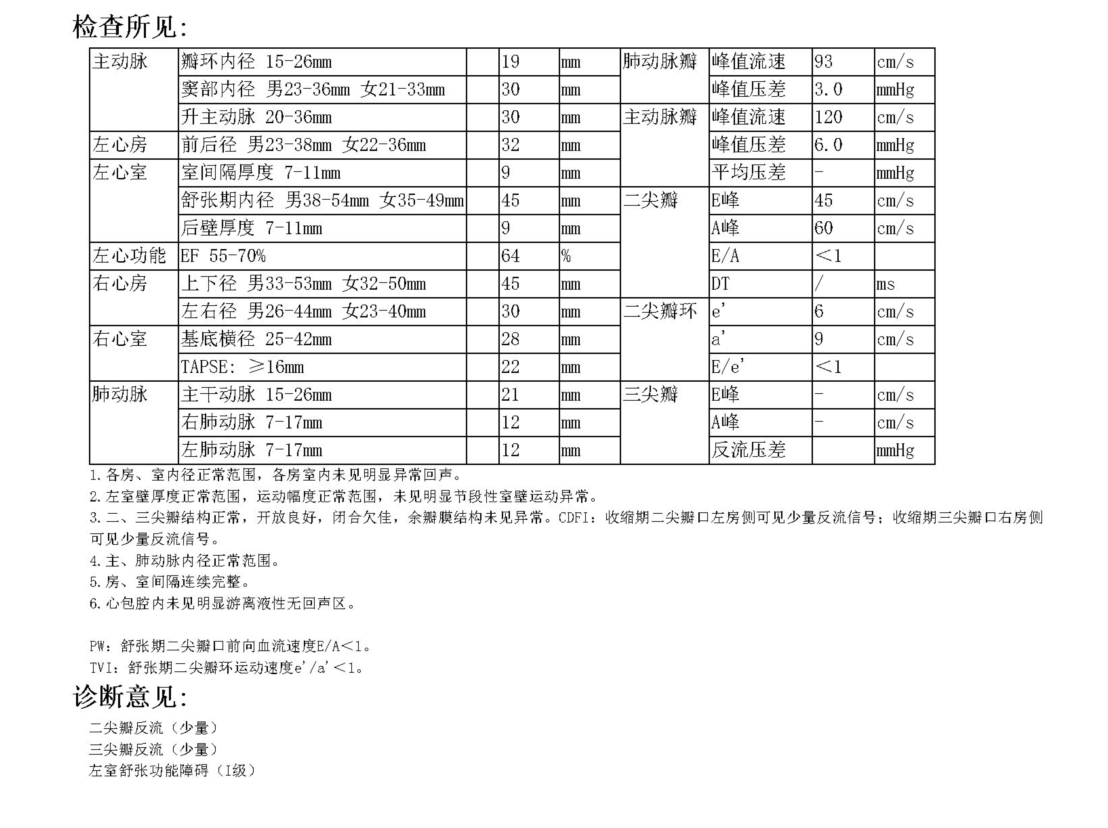

Supplement: Supplementary file 3 — Supplementary Data 2 [file 41746_2026_2648_MOESM3_ESM.zip › echocardiography_reports/122.png]

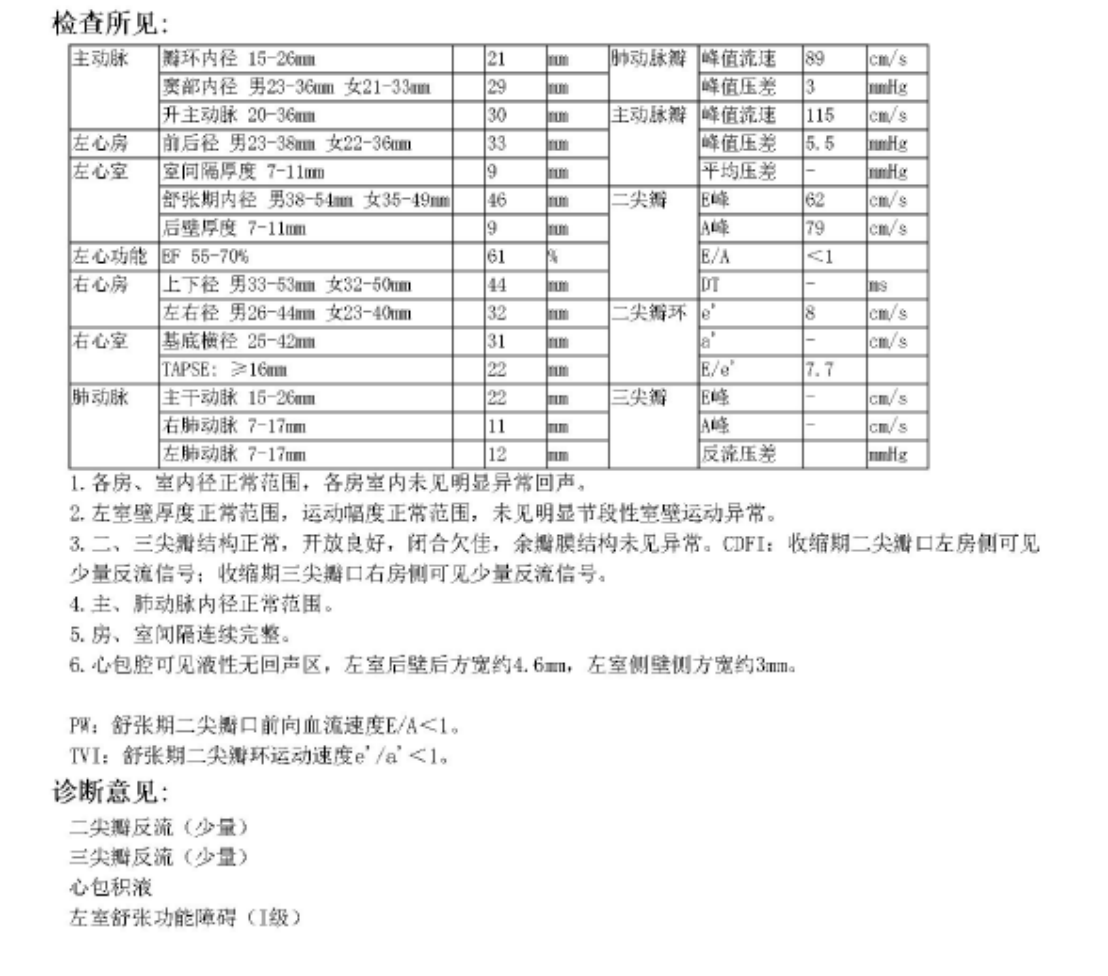

Supplement: Supplementary file 3 — Supplementary Data 2 [file 41746_2026_2648_MOESM3_ESM.zip › echocardiography_reports/123.png]

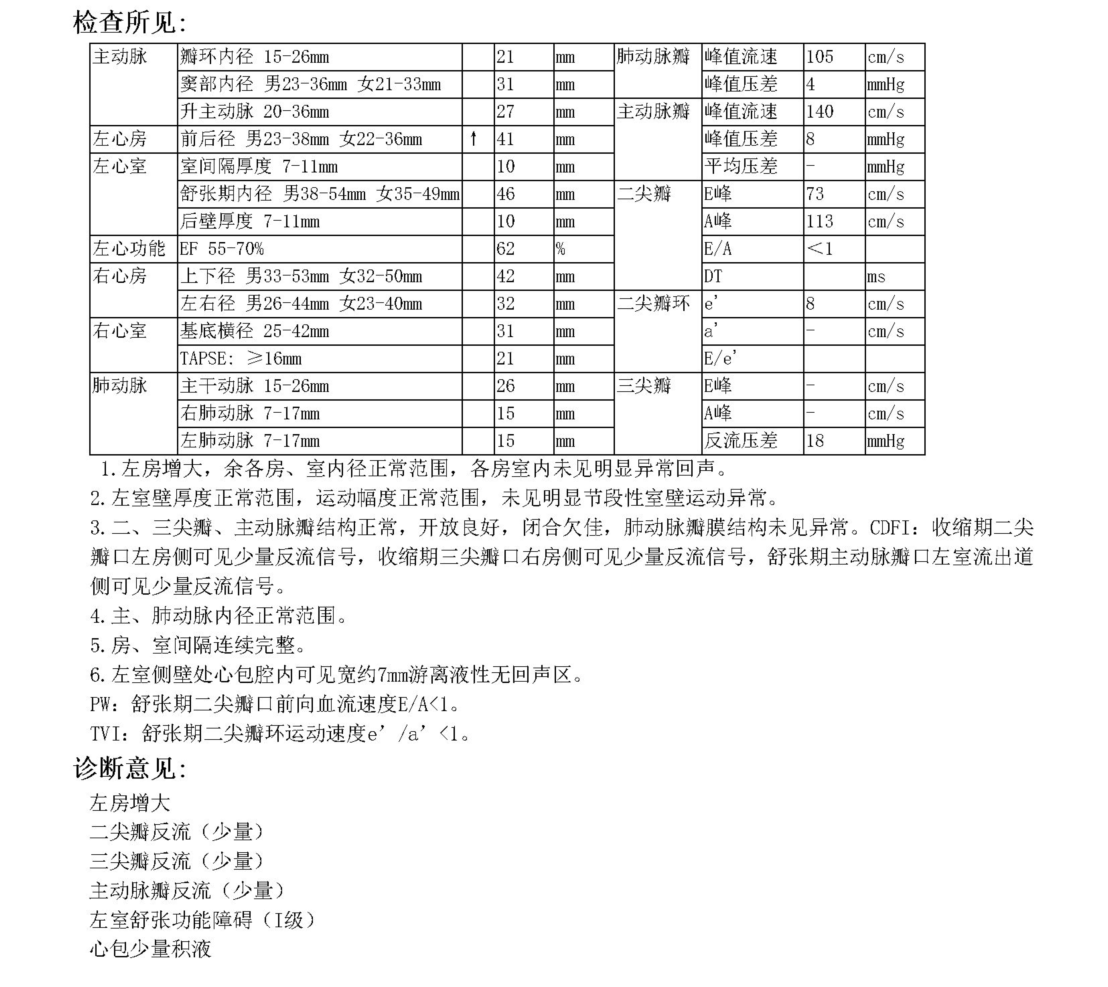

Supplement: Supplementary file 3 — Supplementary Data 2 [file 41746_2026_2648_MOESM3_ESM.zip › echocardiography_reports/124.png]

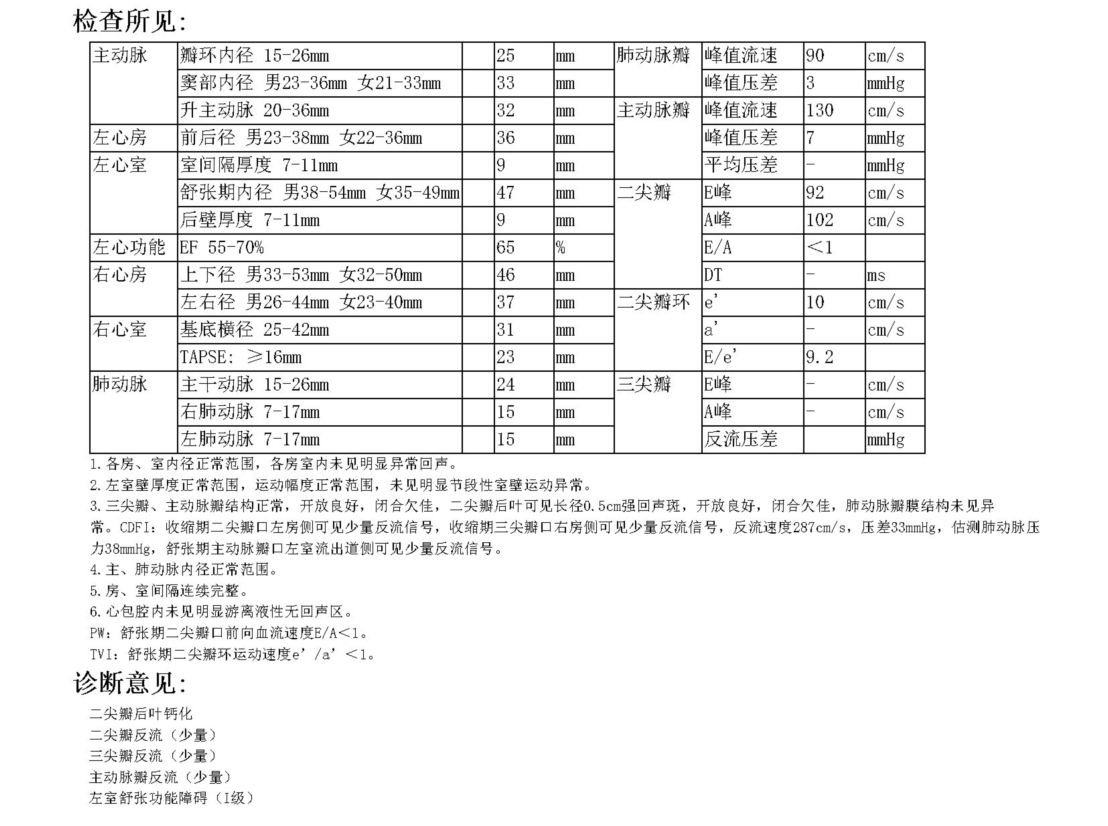

Supplement: Supplementary file 3 — Supplementary Data 2 [file 41746_2026_2648_MOESM3_ESM.zip › echocardiography_reports/125.png]

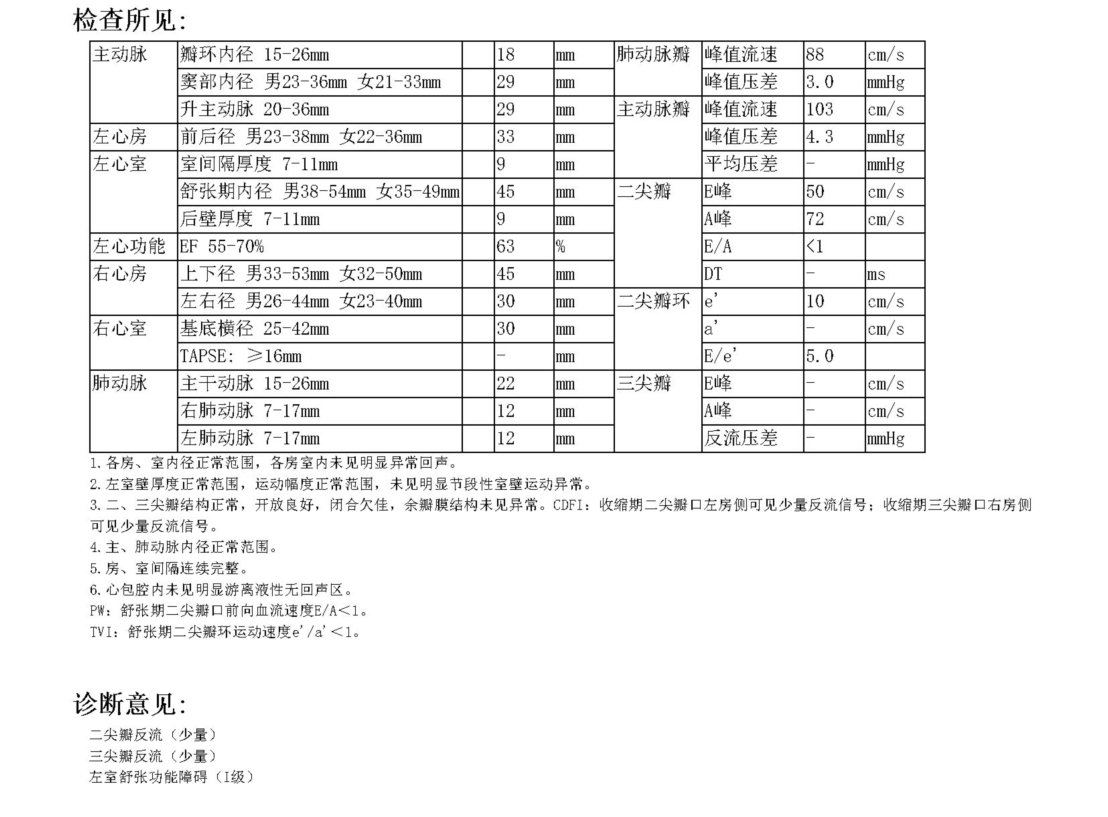

Supplement: Supplementary file 3 — Supplementary Data 2 [file 41746_2026_2648_MOESM3_ESM.zip › echocardiography_reports/126.png]

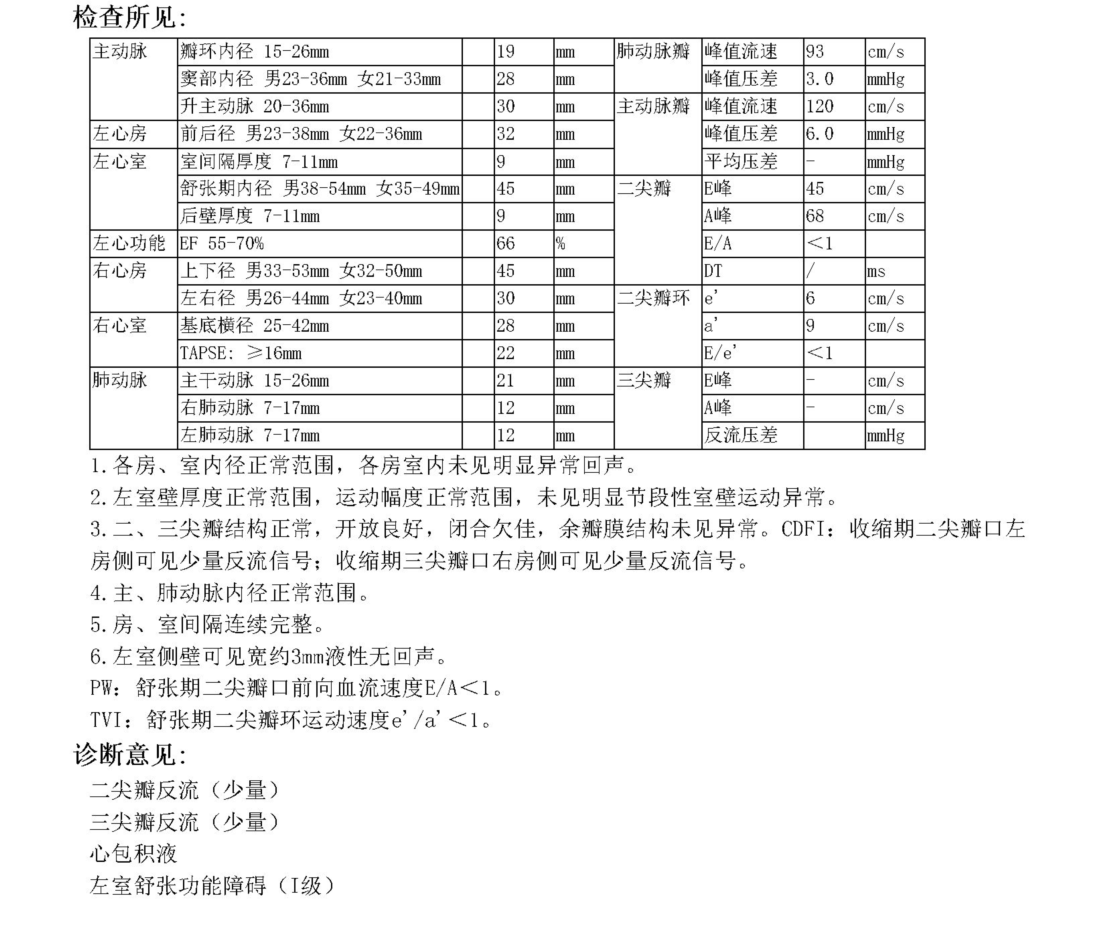

Supplement: Supplementary file 3 — Supplementary Data 2 [file 41746_2026_2648_MOESM3_ESM.zip › echocardiography_reports/127.png]

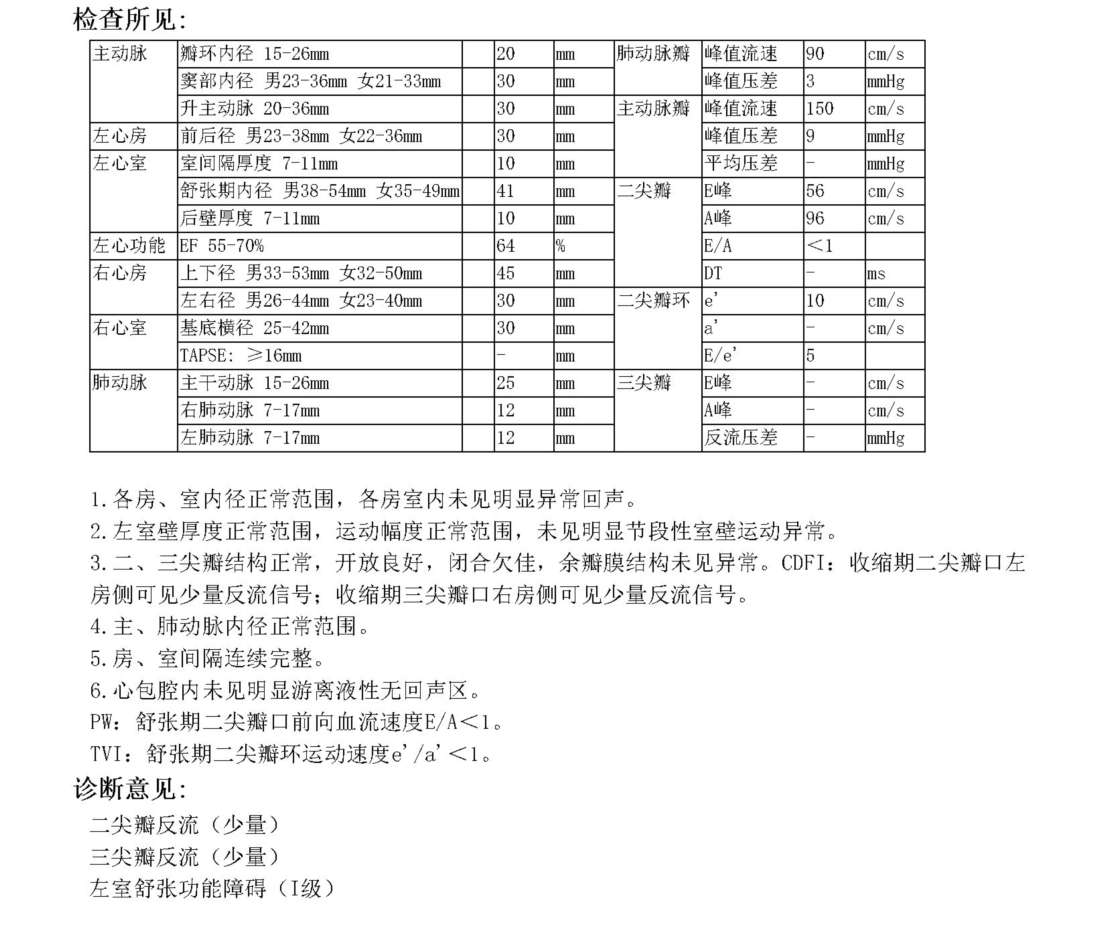

Supplement: Supplementary file 3 — Supplementary Data 2 [file 41746_2026_2648_MOESM3_ESM.zip › echocardiography_reports/128.png]

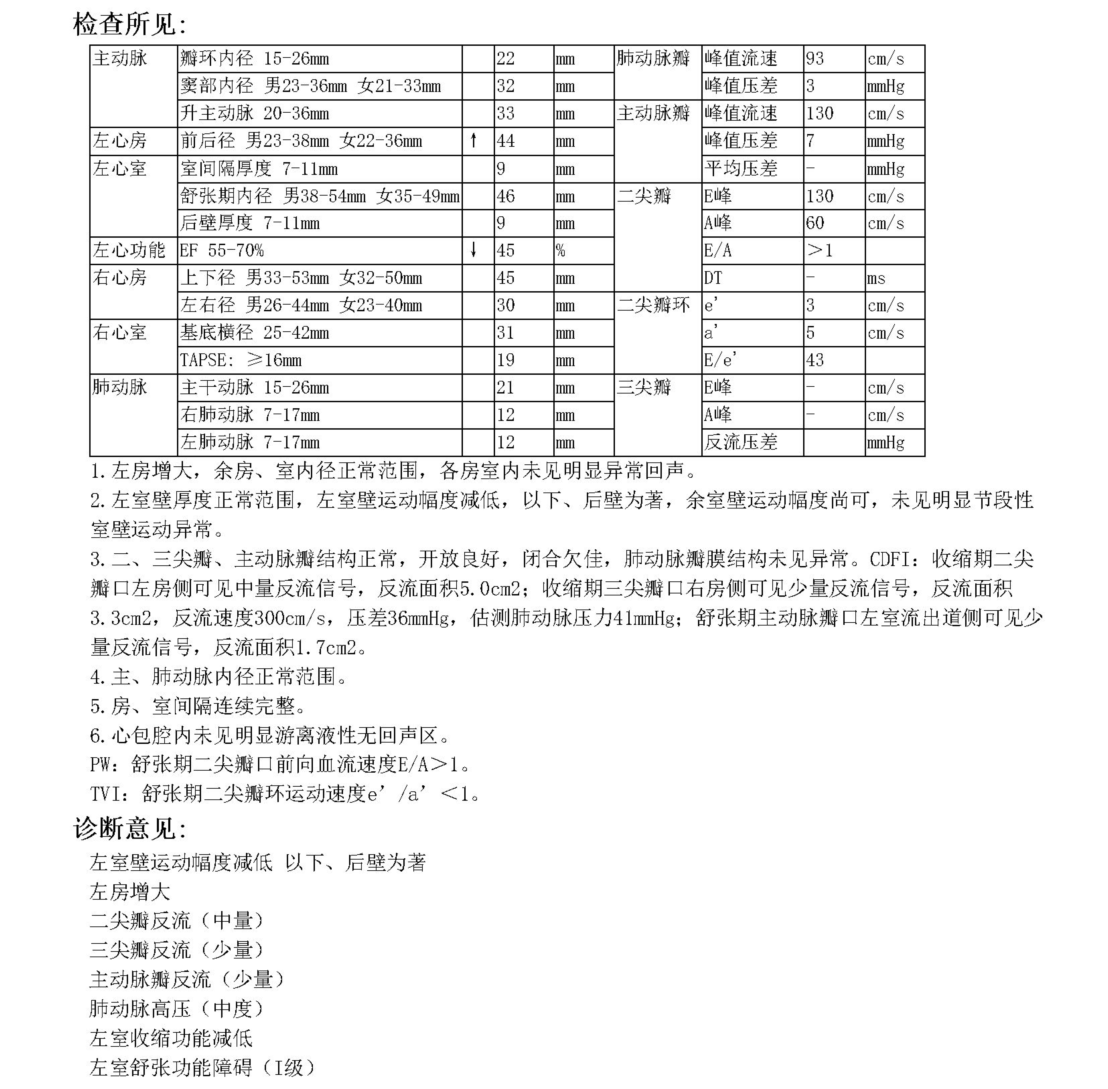

Supplement: Supplementary file 3 — Supplementary Data 2 [file 41746_2026_2648_MOESM3_ESM.zip › echocardiography_reports/129.png]

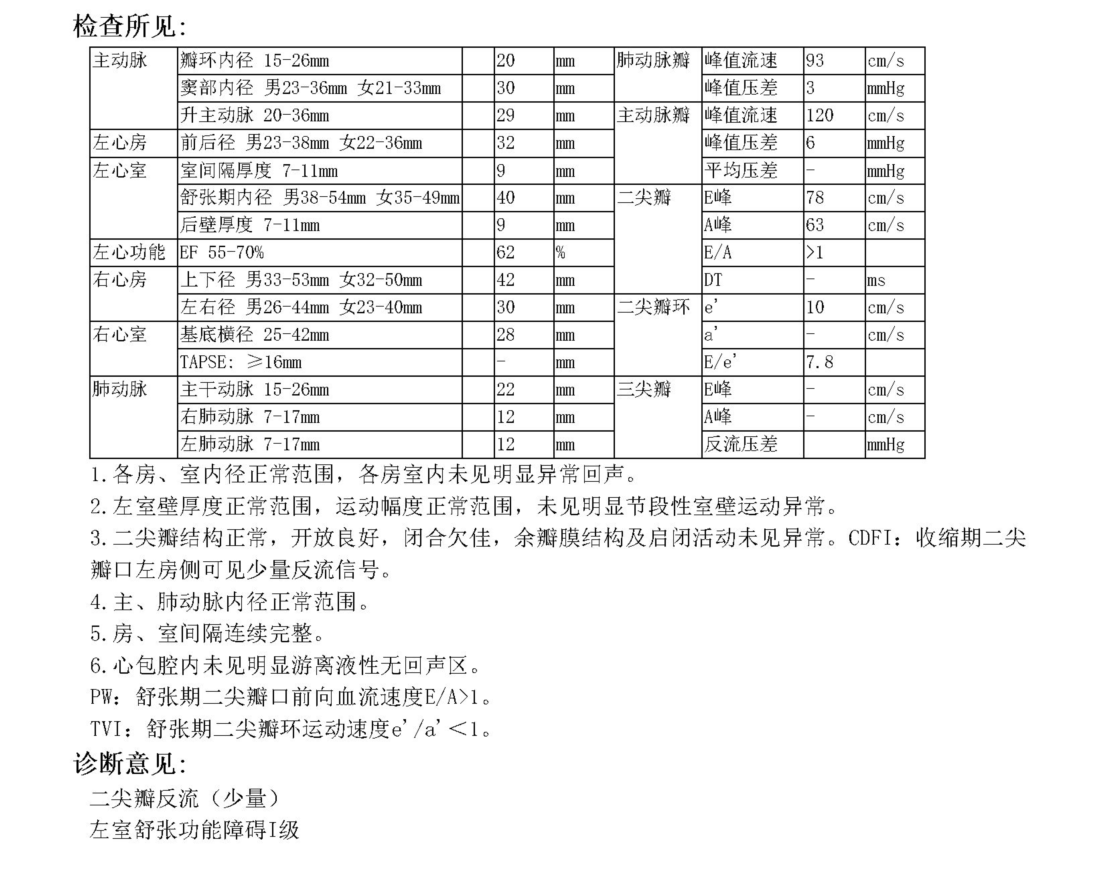

Supplement: Supplementary file 3 — Supplementary Data 2 [file 41746_2026_2648_MOESM3_ESM.zip › echocardiography_reports/130.png]

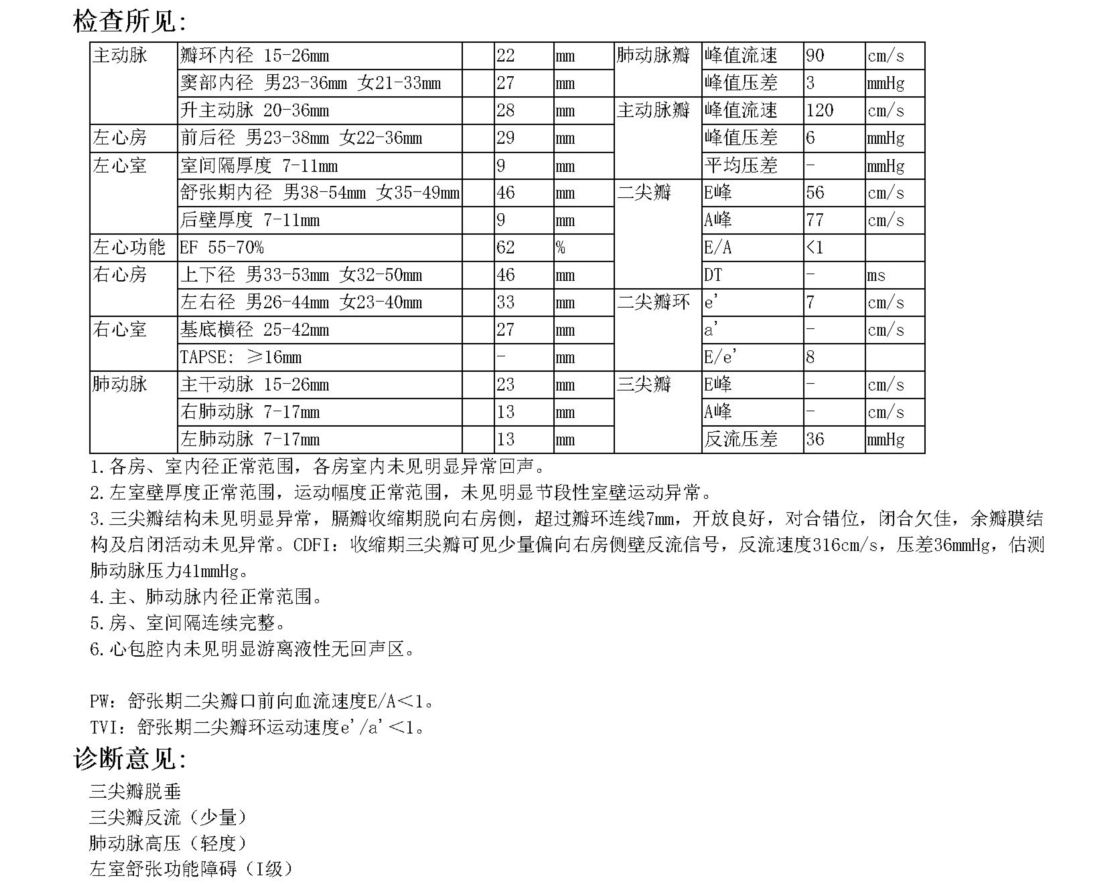

Supplement: Supplementary file 3 — Supplementary Data 2 [file 41746_2026_2648_MOESM3_ESM.zip › echocardiography_reports/131.png]

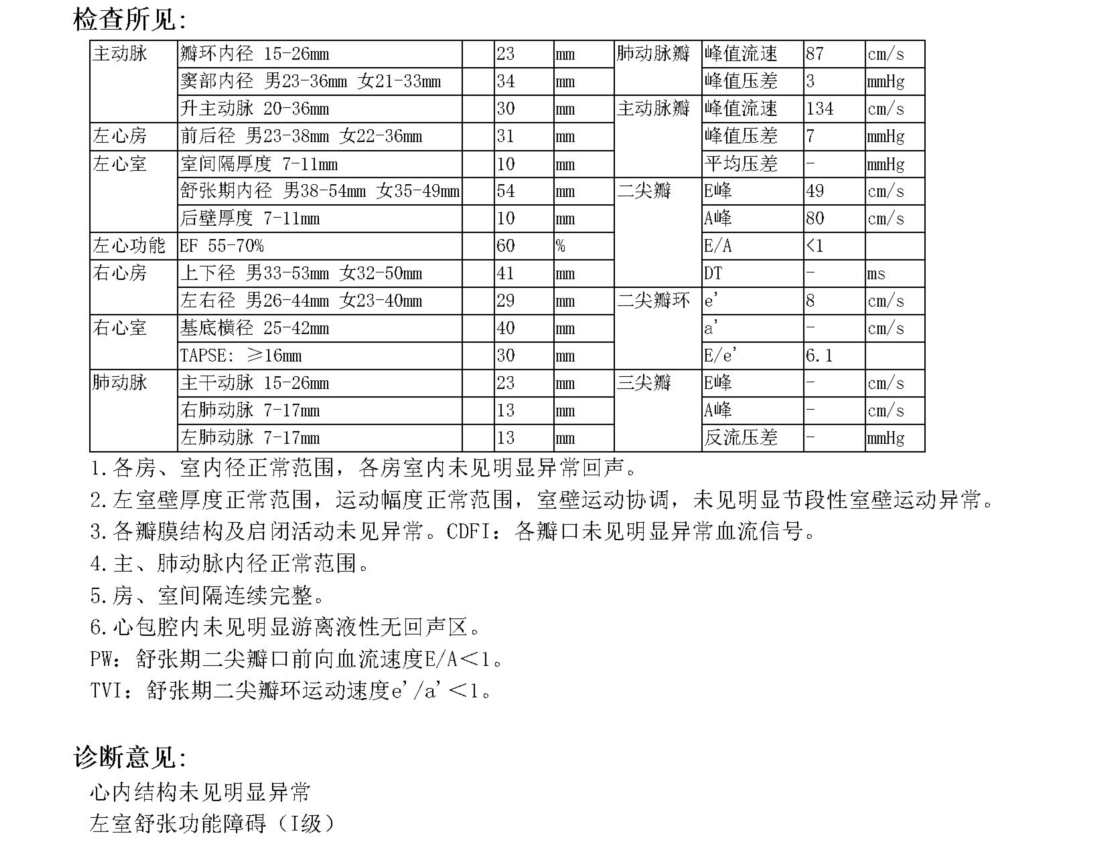

Supplement: Supplementary file 3 — Supplementary Data 2 [file 41746_2026_2648_MOESM3_ESM.zip › echocardiography_reports/132.png]

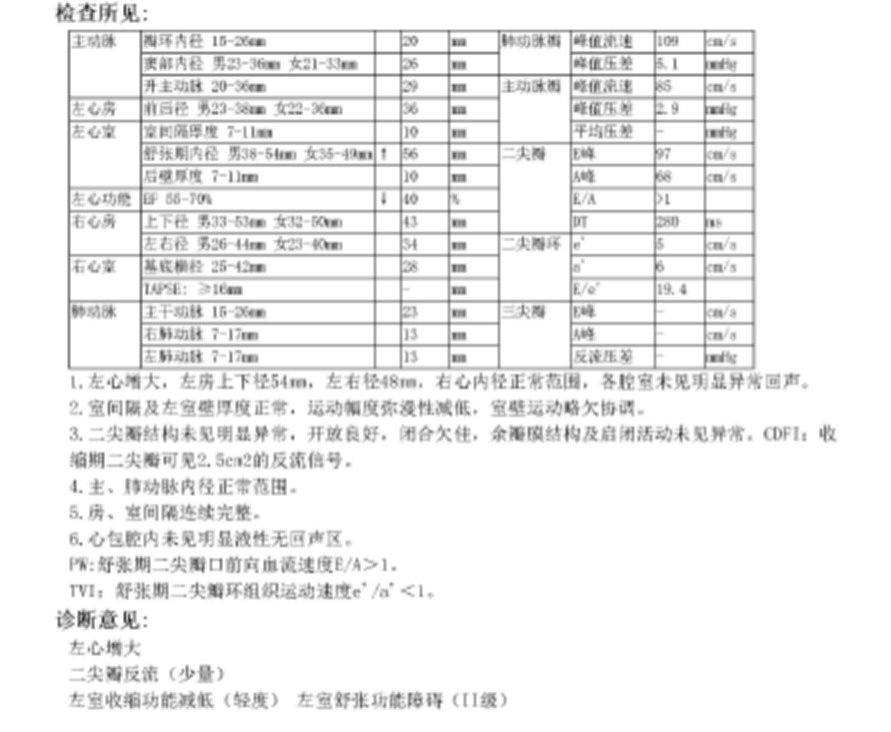

Supplement: Supplementary file 3 — Supplementary Data 2 [file 41746_2026_2648_MOESM3_ESM.zip › echocardiography_reports/133.png]

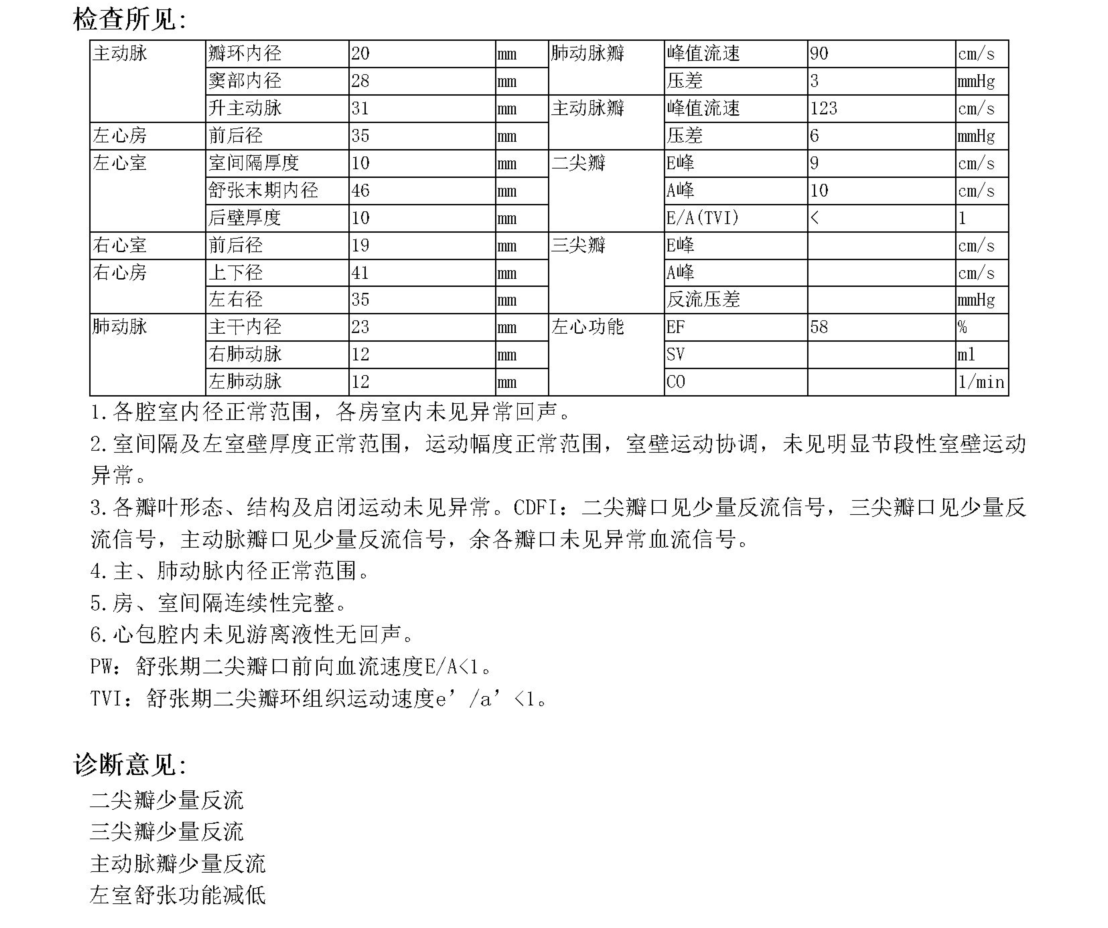

Supplement: Supplementary file 3 — Supplementary Data 2 [file 41746_2026_2648_MOESM3_ESM.zip › echocardiography_reports/134.png]

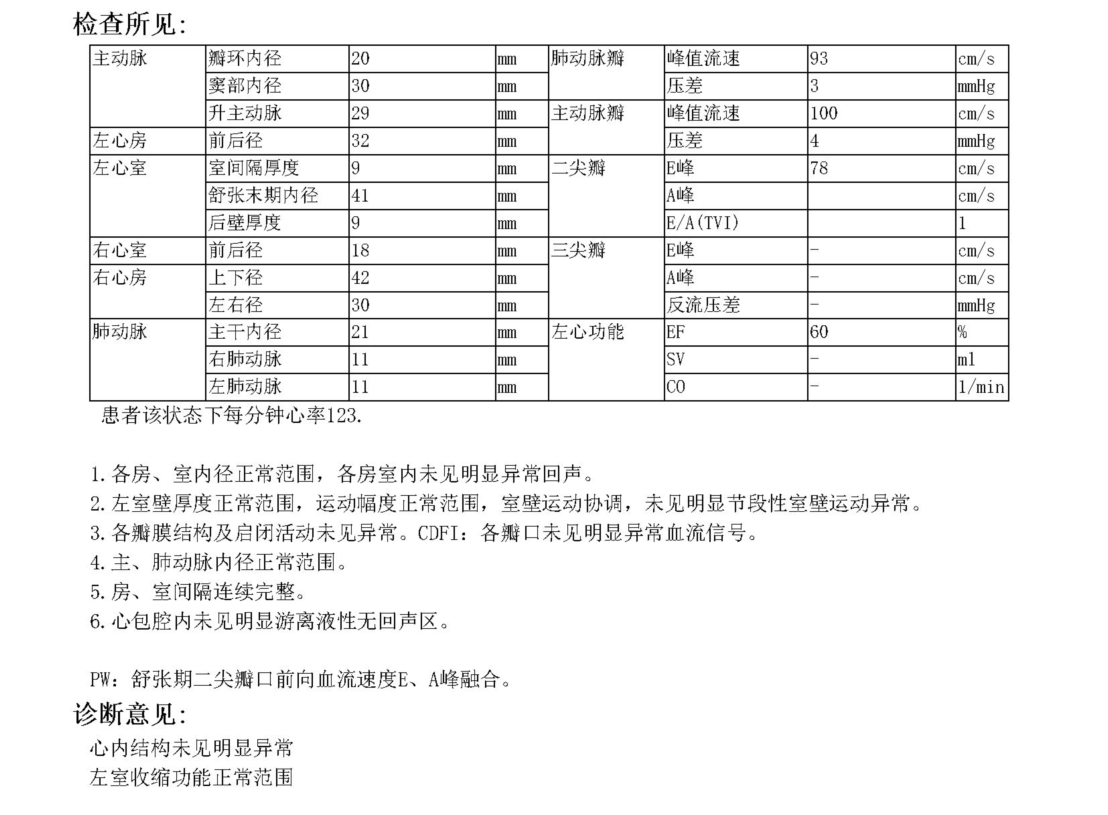

Supplement: Supplementary file 3 — Supplementary Data 2 [file 41746_2026_2648_MOESM3_ESM.zip › echocardiography_reports/135.png]

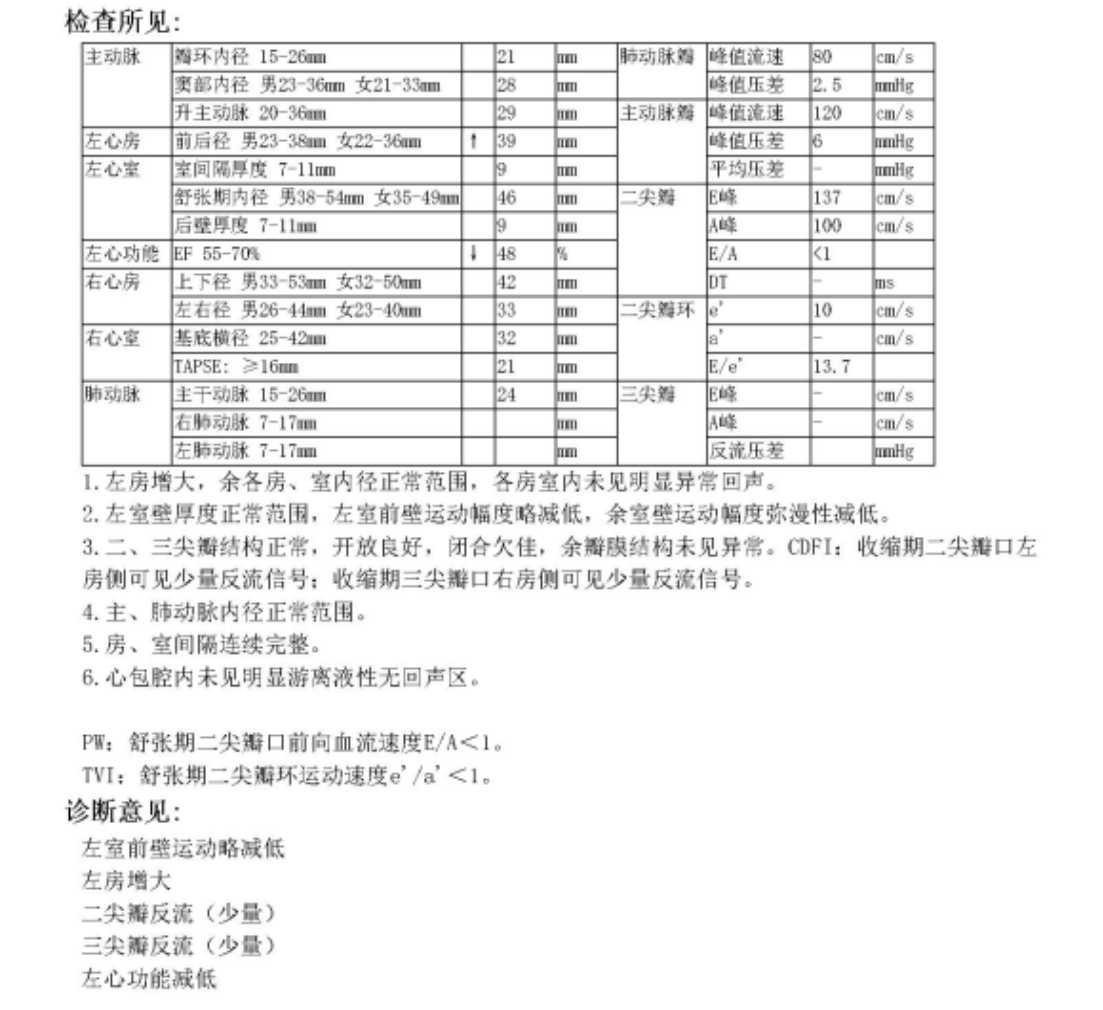

Supplement: Supplementary file 3 — Supplementary Data 2 [file 41746_2026_2648_MOESM3_ESM.zip › echocardiography_reports/136.png]

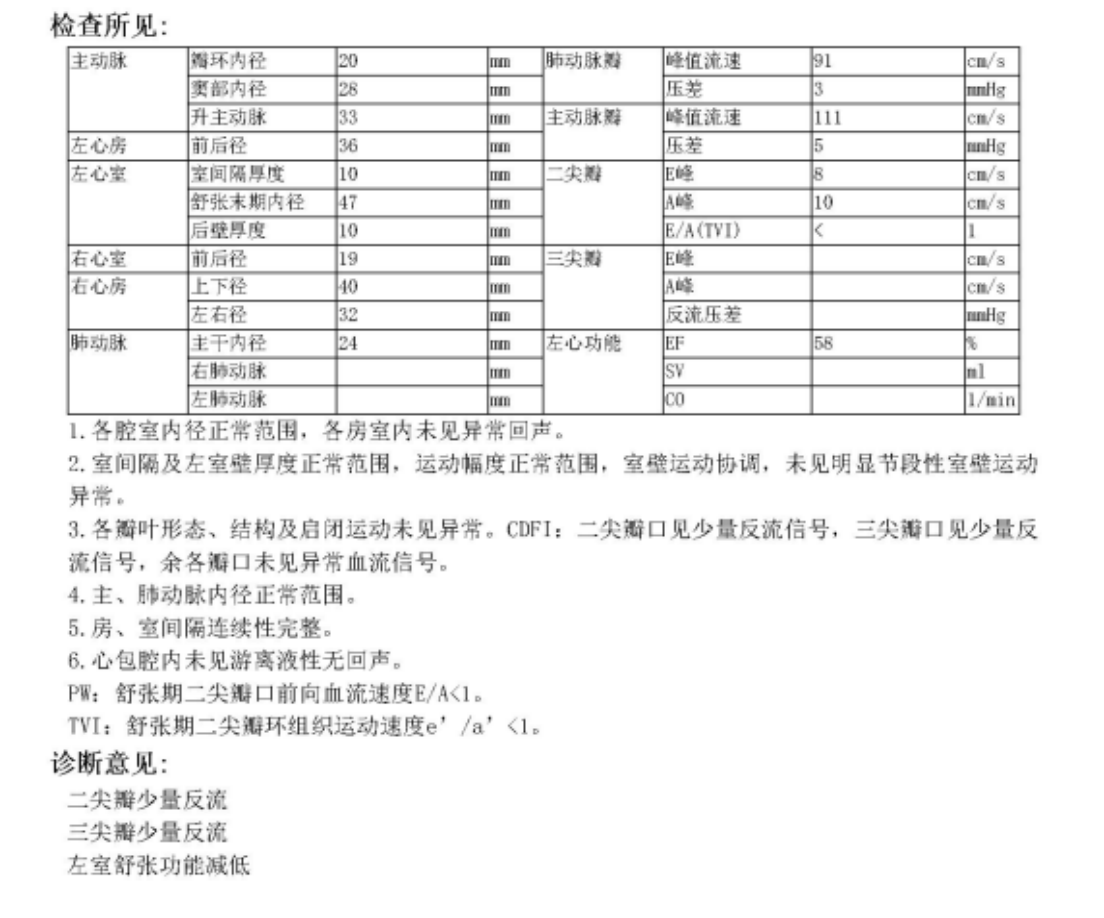

Supplement: Supplementary file 3 — Supplementary Data 2 [file 41746_2026_2648_MOESM3_ESM.zip › echocardiography_reports/137.png]

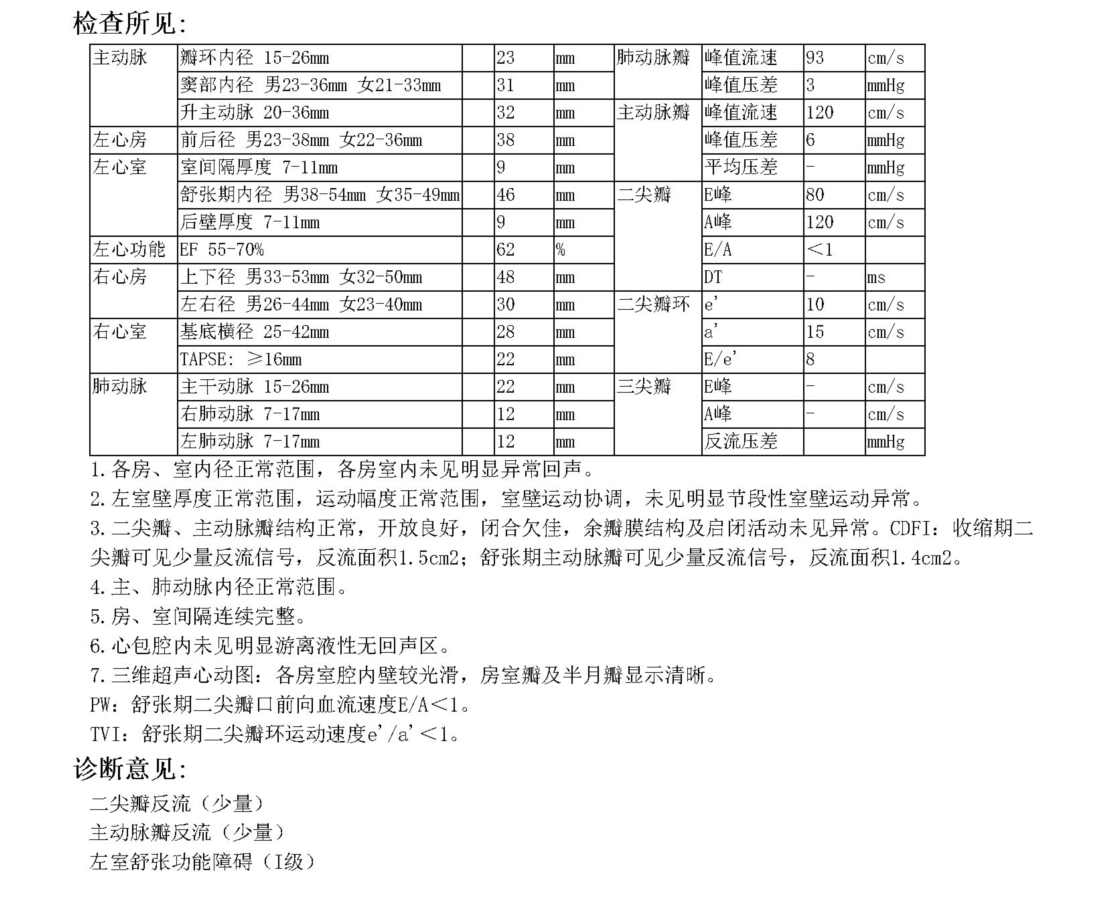

Supplement: Supplementary file 3 — Supplementary Data 2 [file 41746_2026_2648_MOESM3_ESM.zip › echocardiography_reports/138.png]

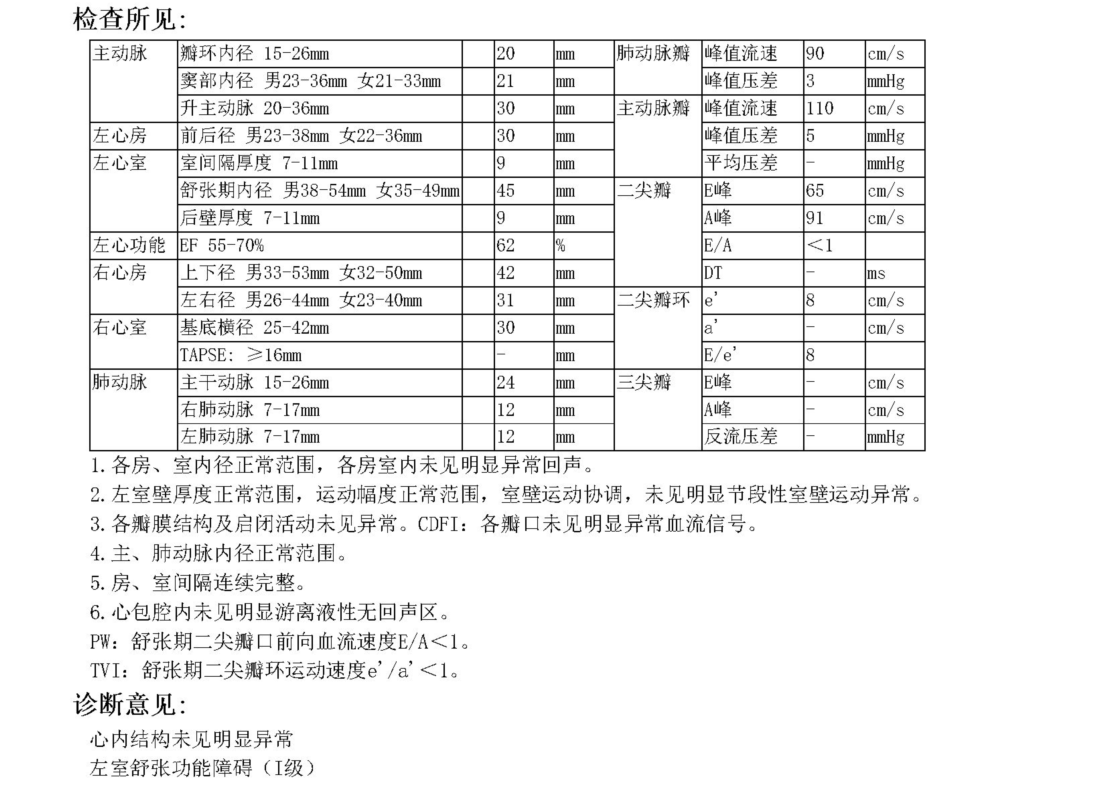

Supplement: Supplementary file 3 — Supplementary Data 2 [file 41746_2026_2648_MOESM3_ESM.zip › echocardiography_reports/139.png]

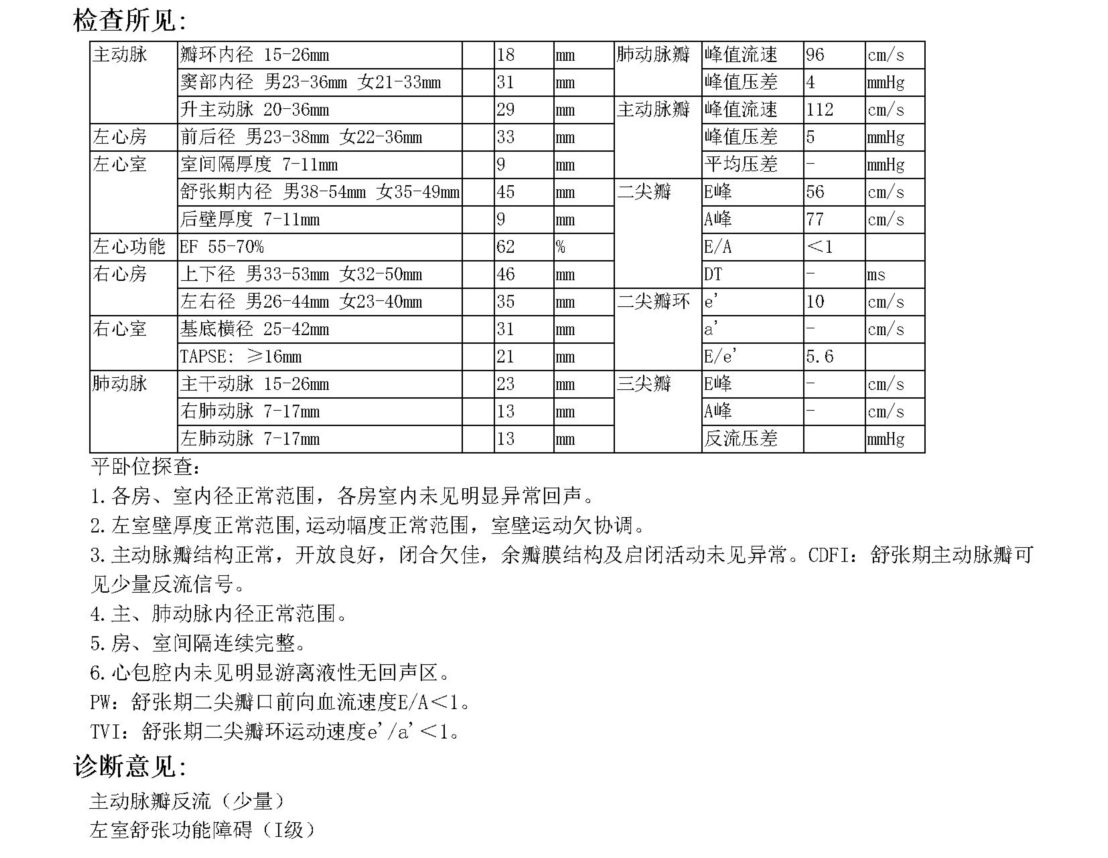

Supplement: Supplementary file 3 — Supplementary Data 2 [file 41746_2026_2648_MOESM3_ESM.zip › echocardiography_reports/140.png]

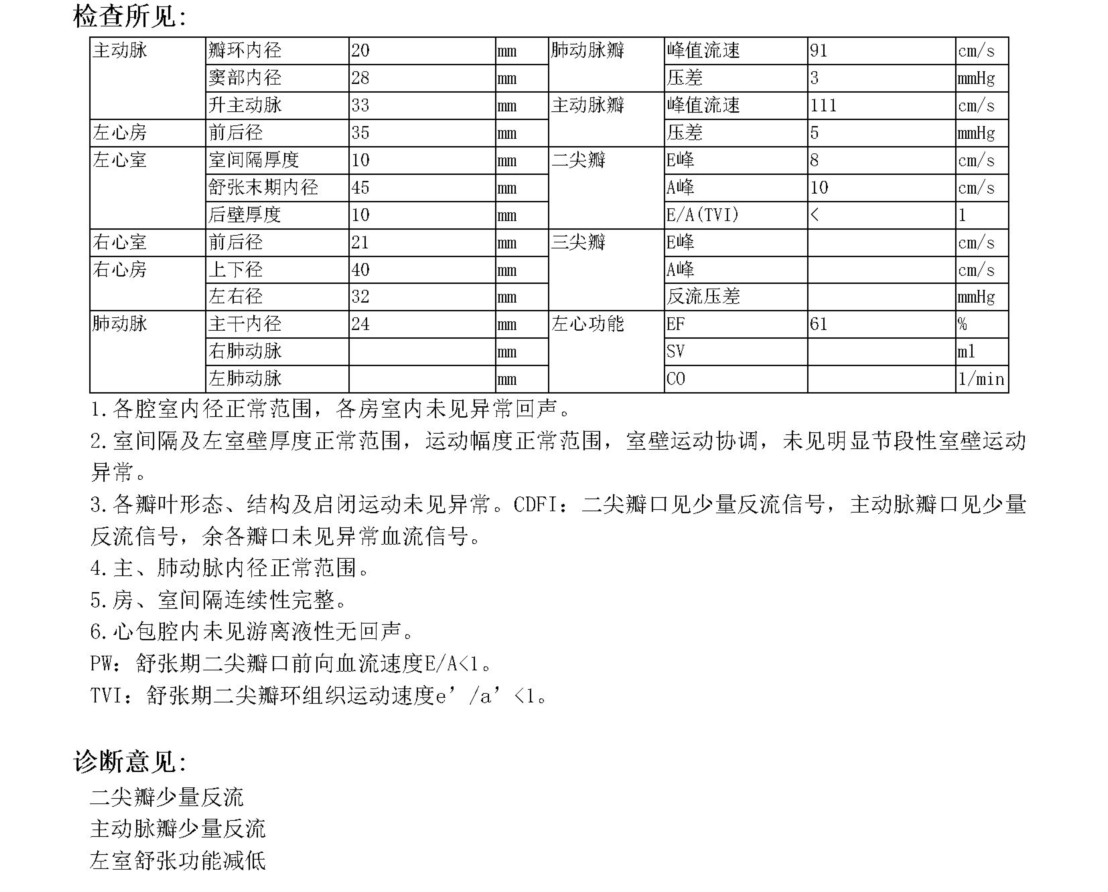

Supplement: Supplementary file 3 — Supplementary Data 2 [file 41746_2026_2648_MOESM3_ESM.zip › echocardiography_reports/141.png]

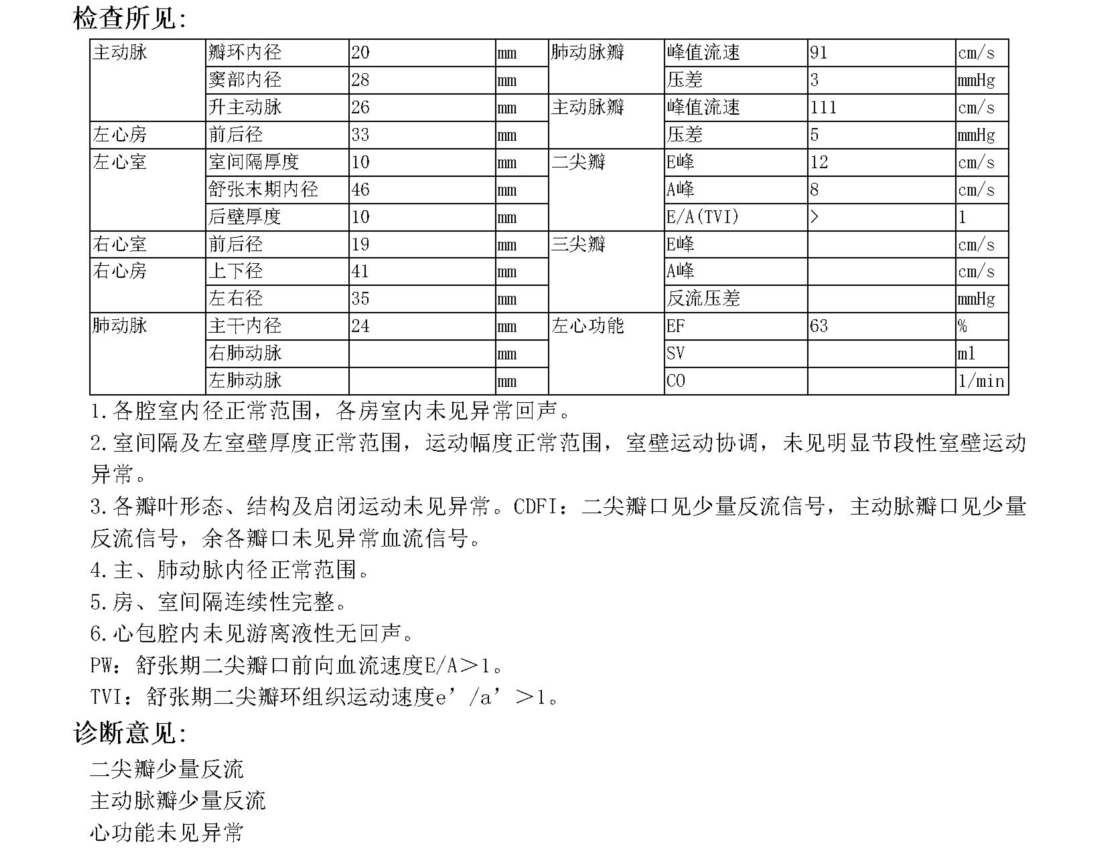

Supplement: Supplementary file 3 — Supplementary Data 2 [file 41746_2026_2648_MOESM3_ESM.zip › echocardiography_reports/142.png]

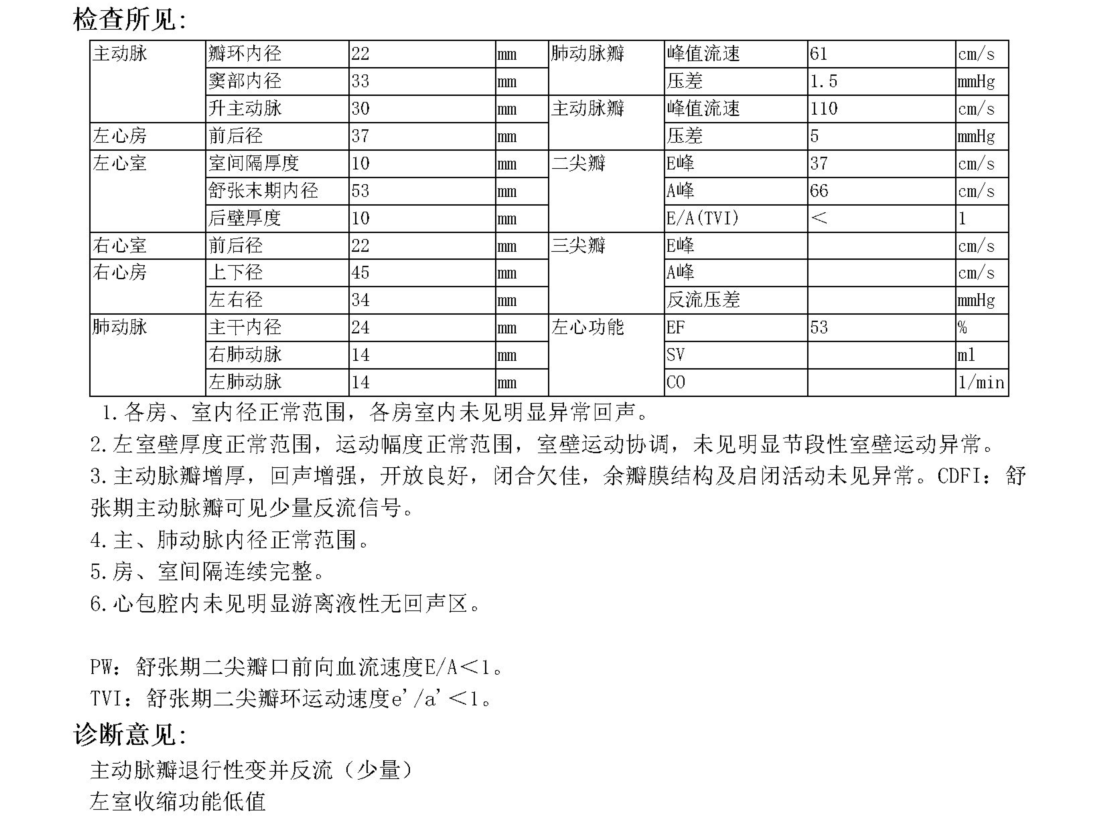

Supplement: Supplementary file 3 — Supplementary Data 2 [file 41746_2026_2648_MOESM3_ESM.zip › echocardiography_reports/143.png]

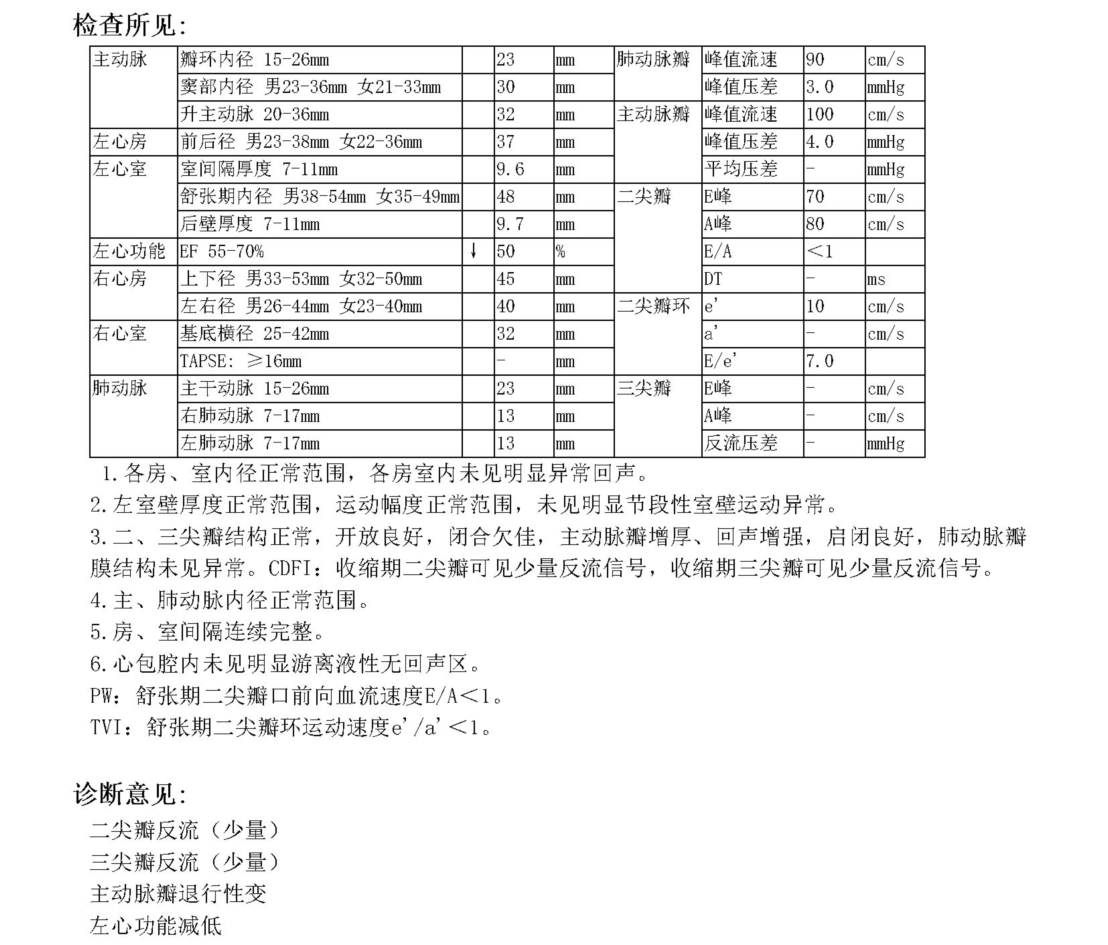

Supplement: Supplementary file 3 — Supplementary Data 2 [file 41746_2026_2648_MOESM3_ESM.zip › echocardiography_reports/144.png]

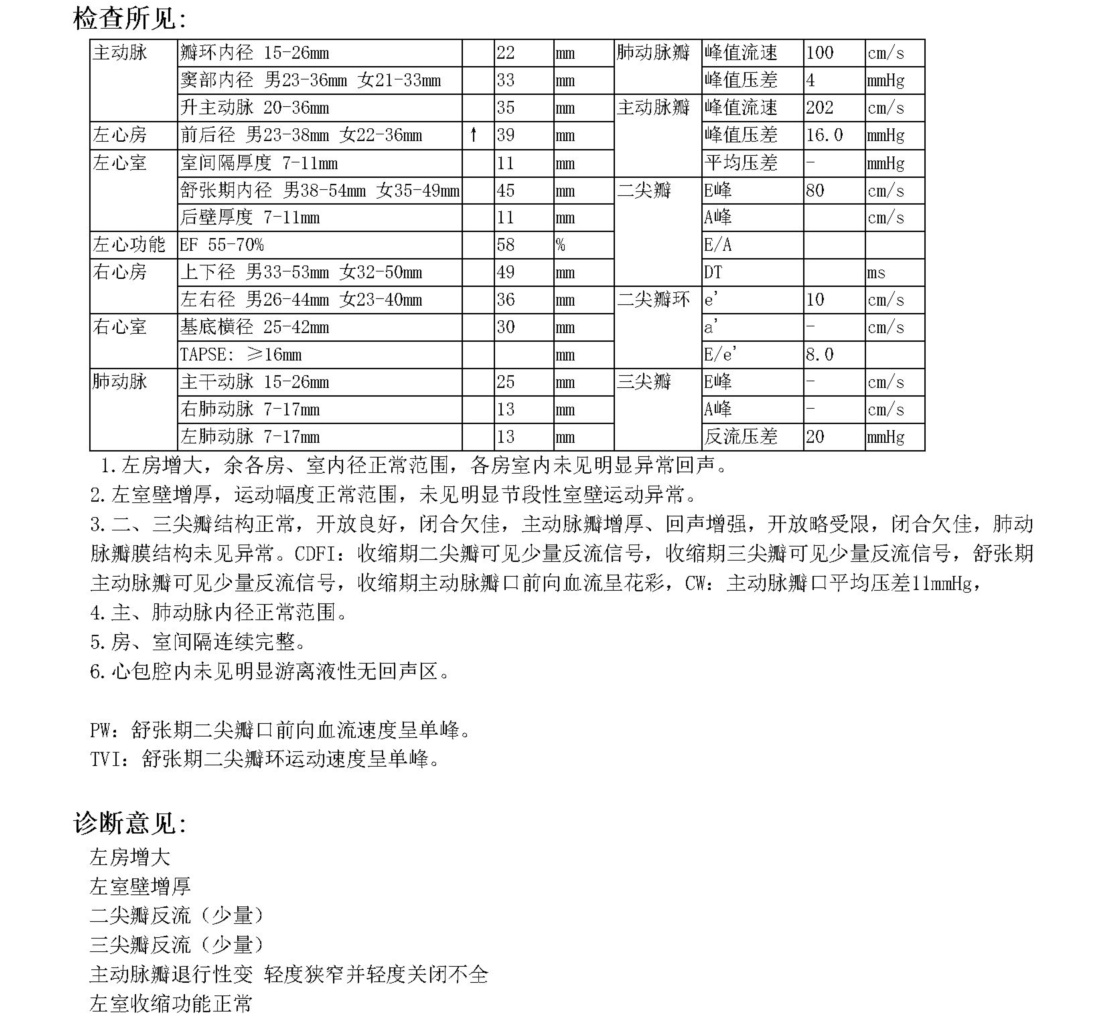

Supplement: Supplementary file 3 — Supplementary Data 2 [file 41746_2026_2648_MOESM3_ESM.zip › echocardiography_reports/145.png]

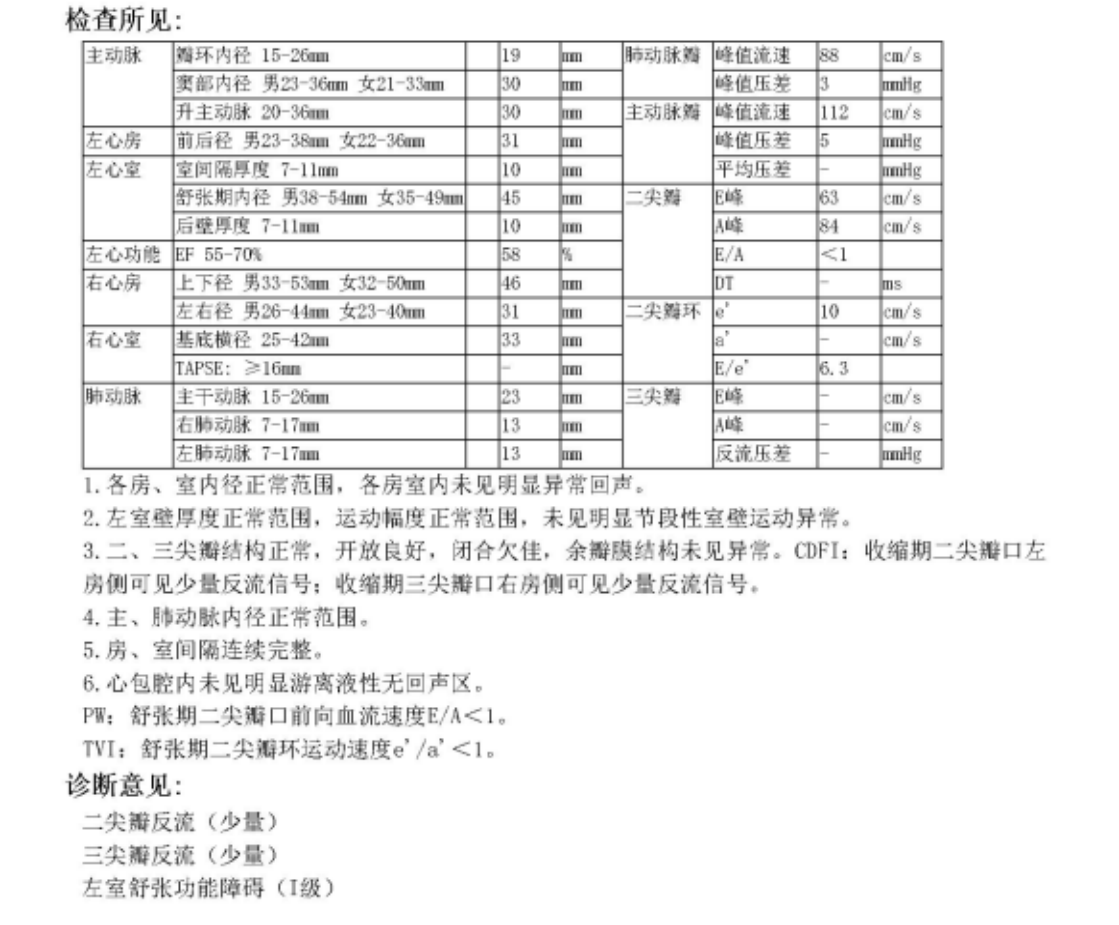

Supplement: Supplementary file 3 — Supplementary Data 2 [file 41746_2026_2648_MOESM3_ESM.zip › echocardiography_reports/146.png]

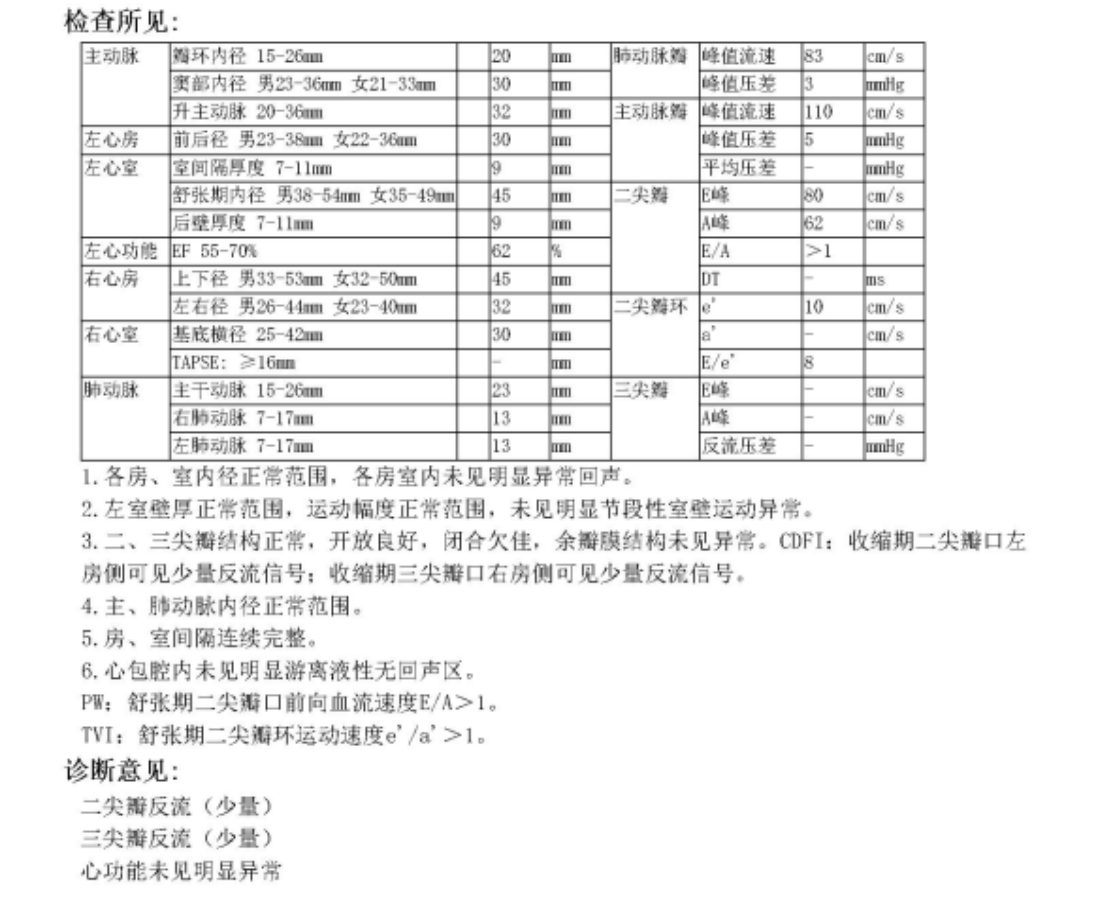

Supplement: Supplementary file 3 — Supplementary Data 2 [file 41746_2026_2648_MOESM3_ESM.zip › echocardiography_reports/147.png]

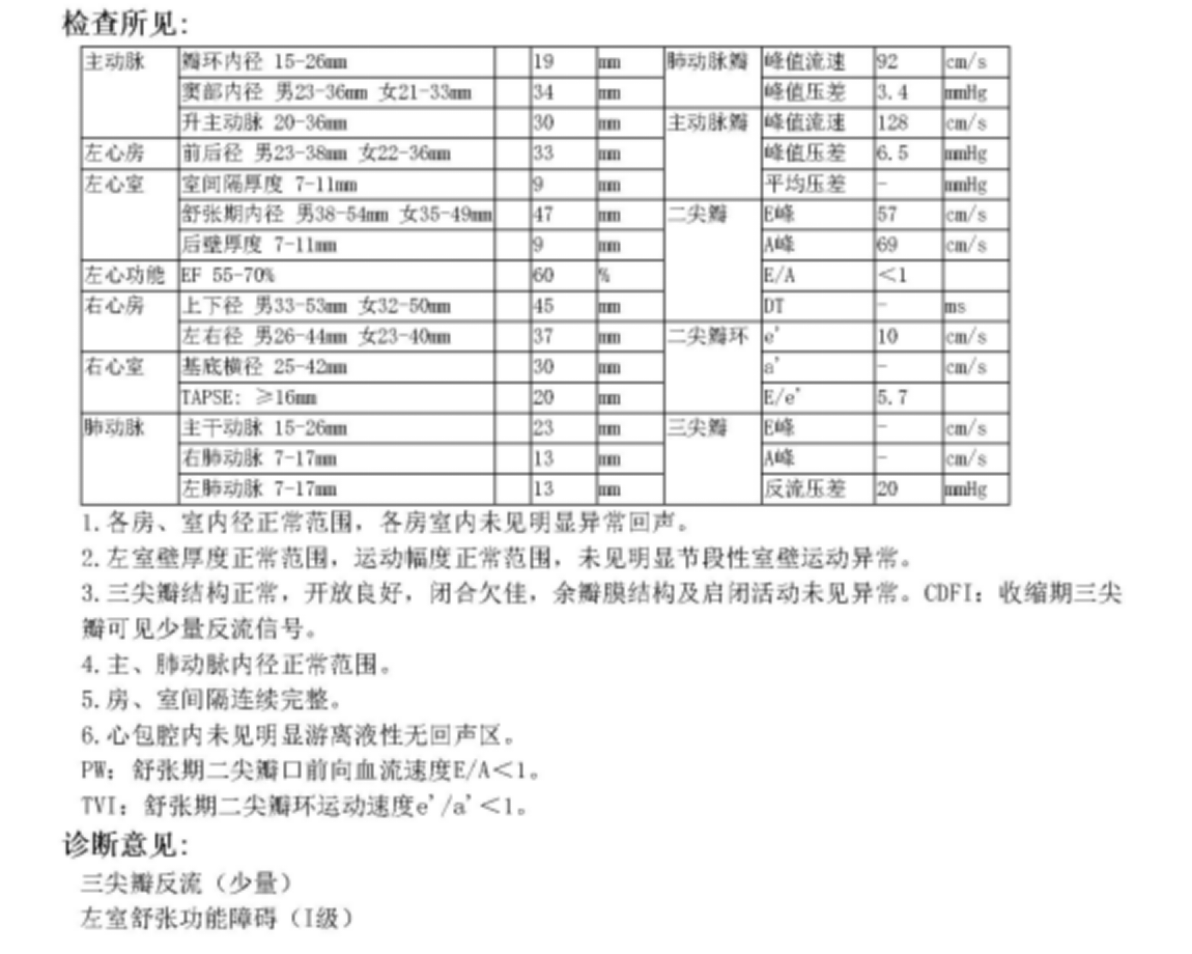

Supplement: Supplementary file 3 — Supplementary Data 2 [file 41746_2026_2648_MOESM3_ESM.zip › echocardiography_reports/148.png]

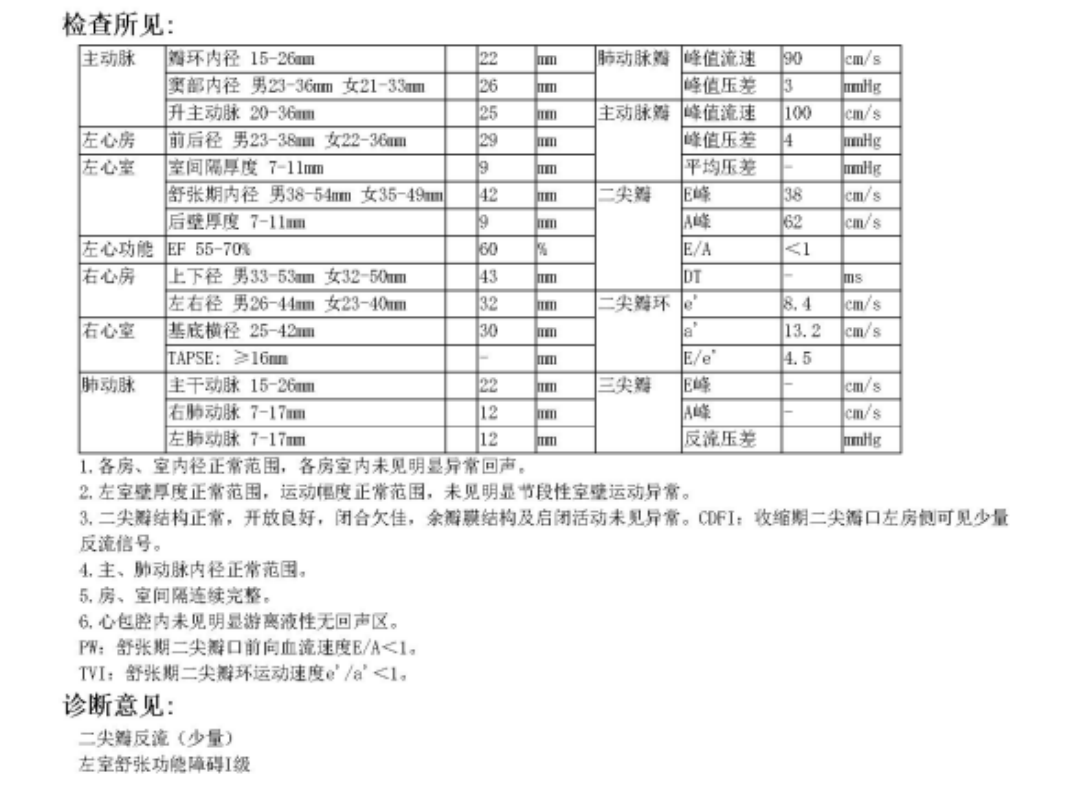

Supplement: Supplementary file 3 — Supplementary Data 2 [file 41746_2026_2648_MOESM3_ESM.zip › echocardiography_reports/149.png]

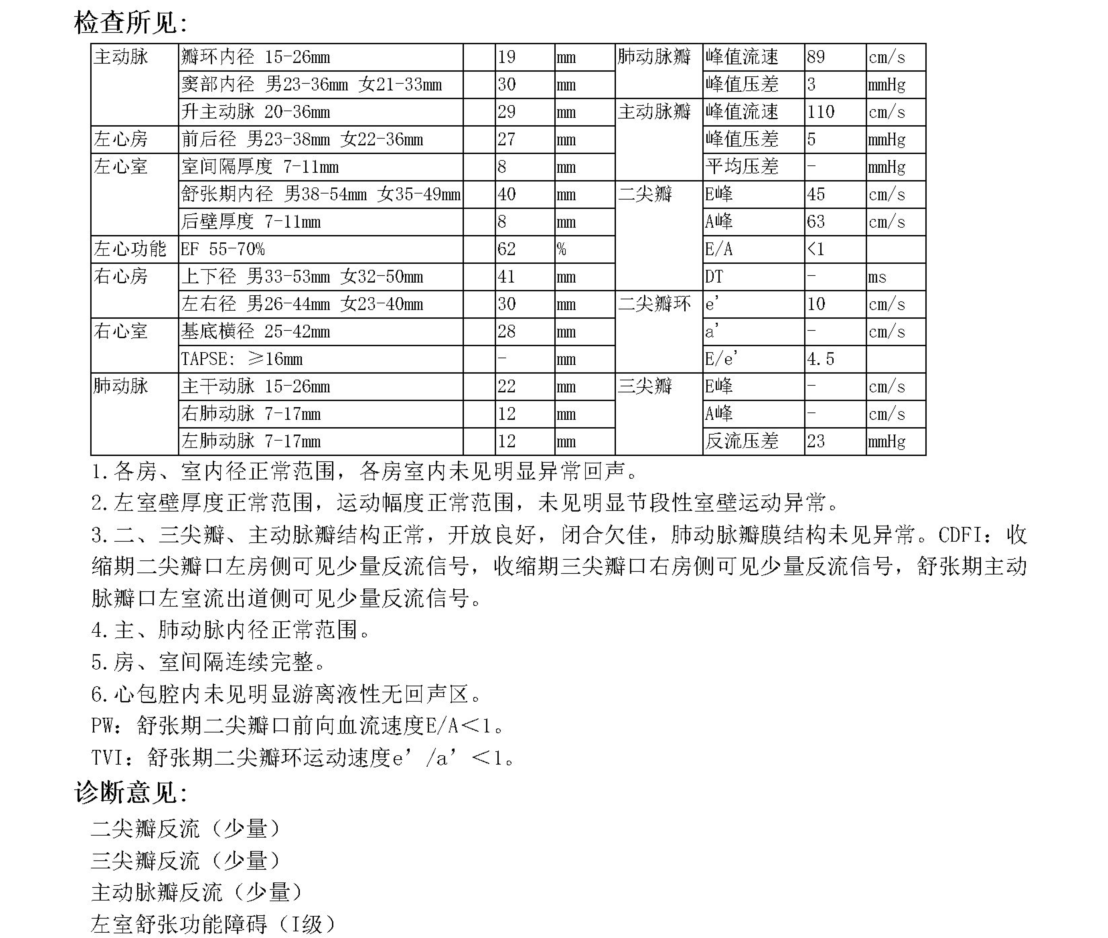

Supplement: Supplementary file 3 — Supplementary Data 2 [file 41746_2026_2648_MOESM3_ESM.zip › echocardiography_reports/150.png]

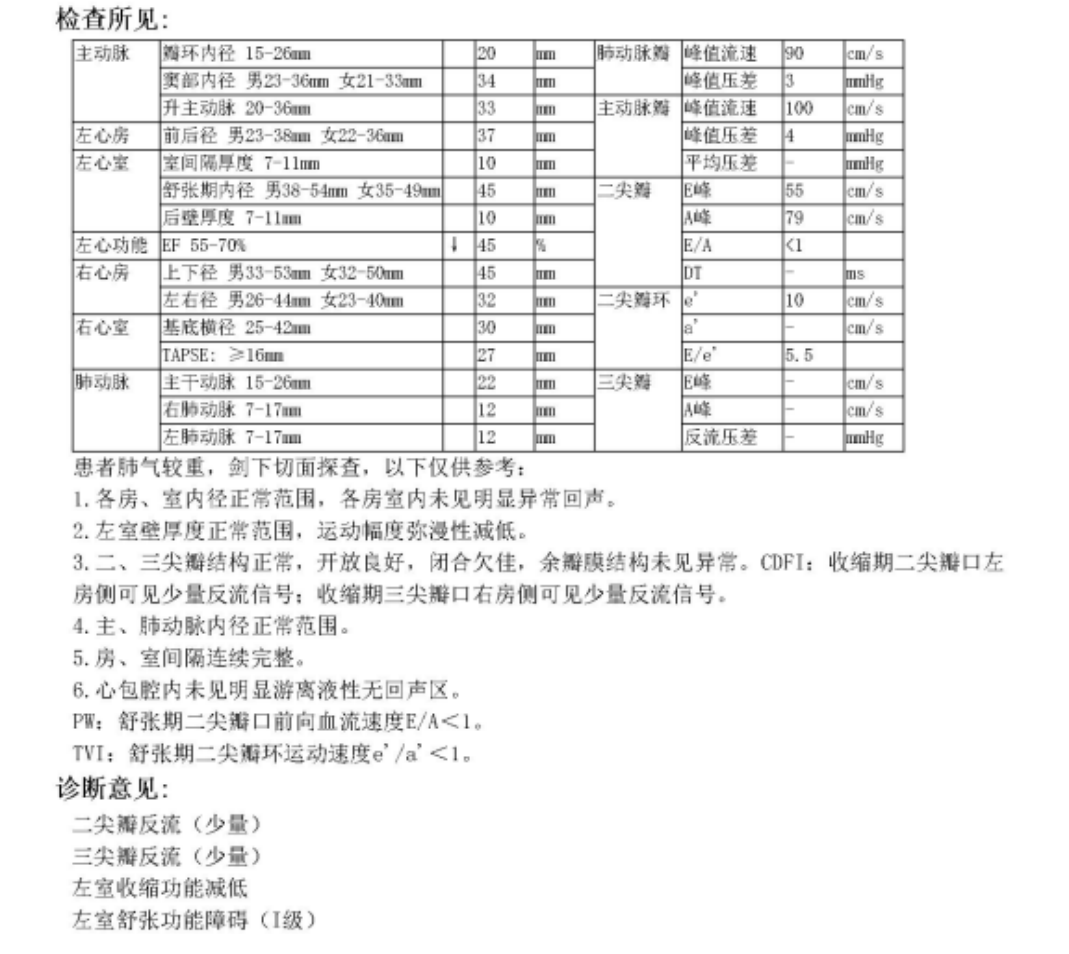

Supplement: Supplementary file 3 — Supplementary Data 2 [file 41746_2026_2648_MOESM3_ESM.zip › echocardiography_reports/151.png]

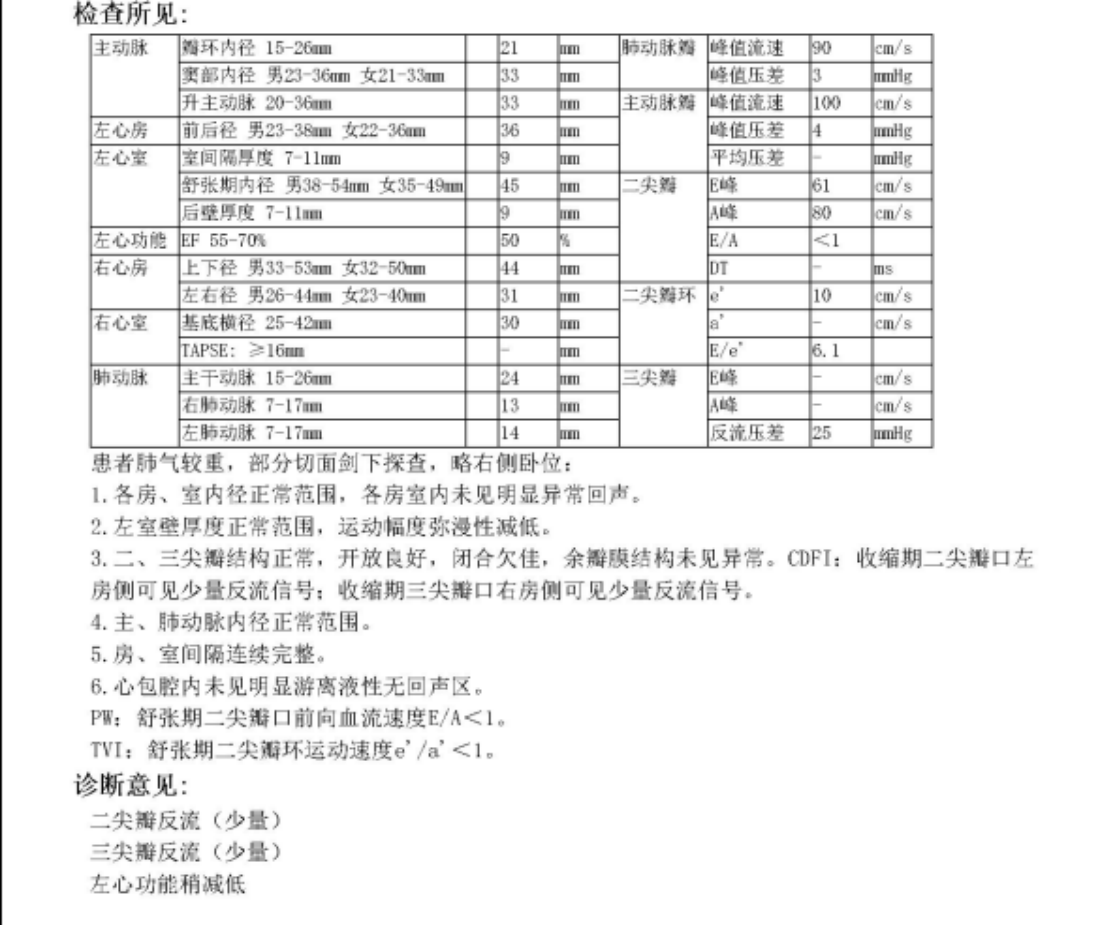

Supplement: Supplementary file 3 — Supplementary Data 2 [file 41746_2026_2648_MOESM3_ESM.zip › echocardiography_reports/152.png]

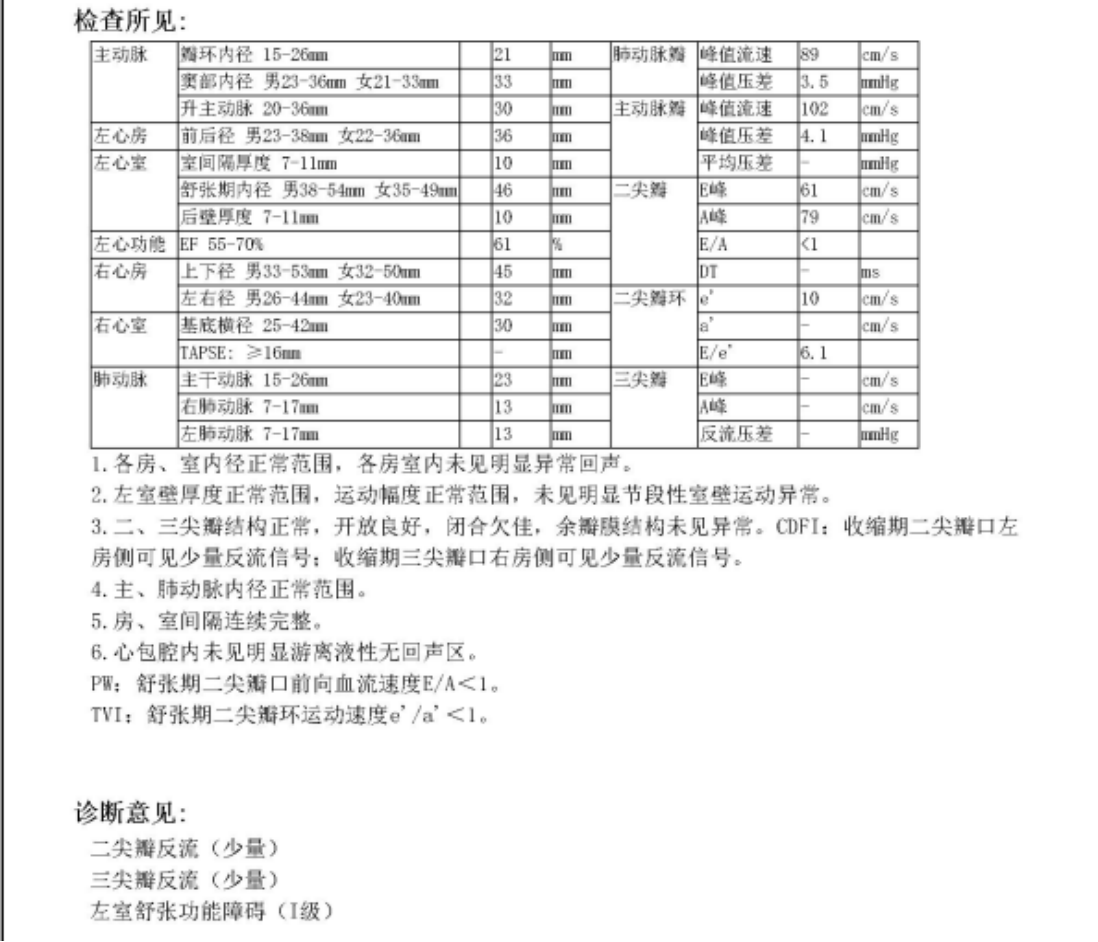

Supplement: Supplementary file 3 — Supplementary Data 2 [file 41746_2026_2648_MOESM3_ESM.zip › echocardiography_reports/153.png]

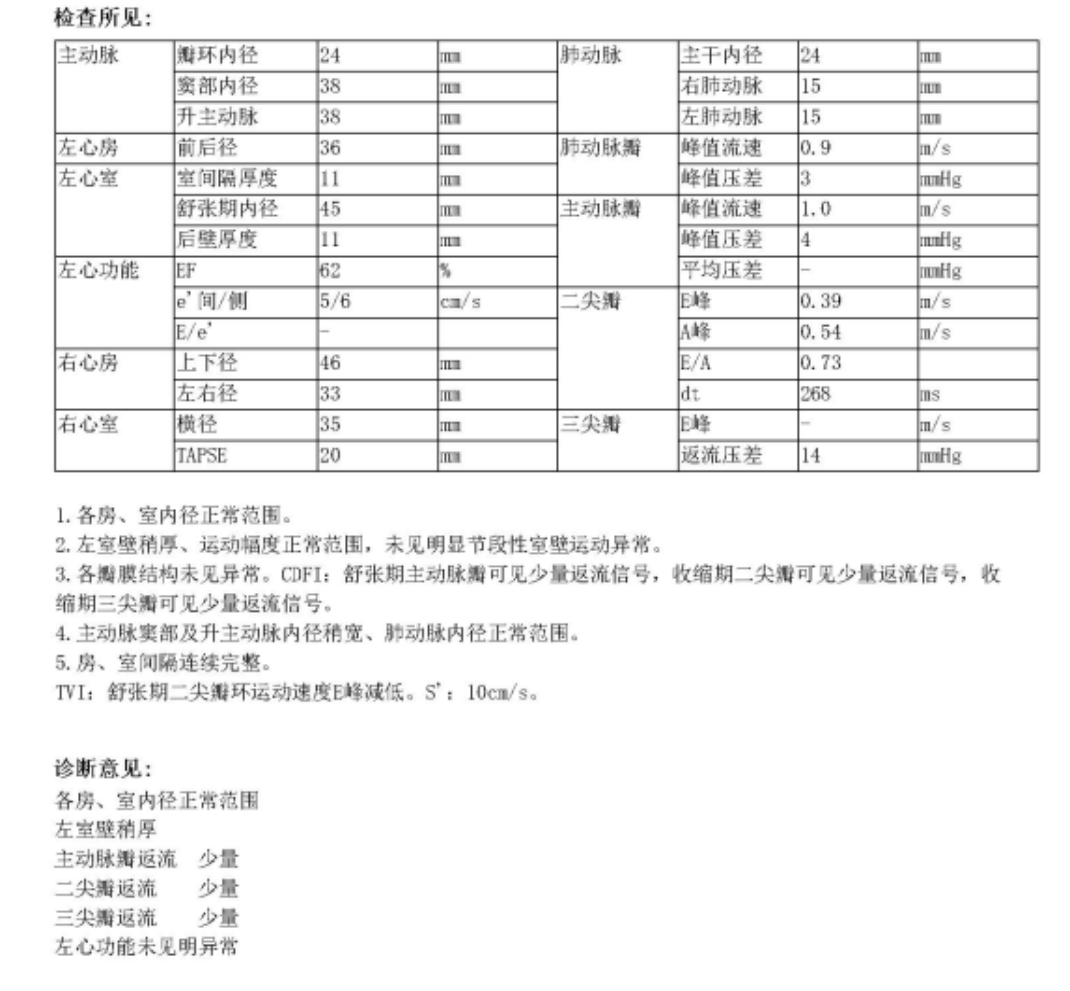

Supplement: Supplementary file 3 — Supplementary Data 2 [file 41746_2026_2648_MOESM3_ESM.zip › echocardiography_reports/154.png]

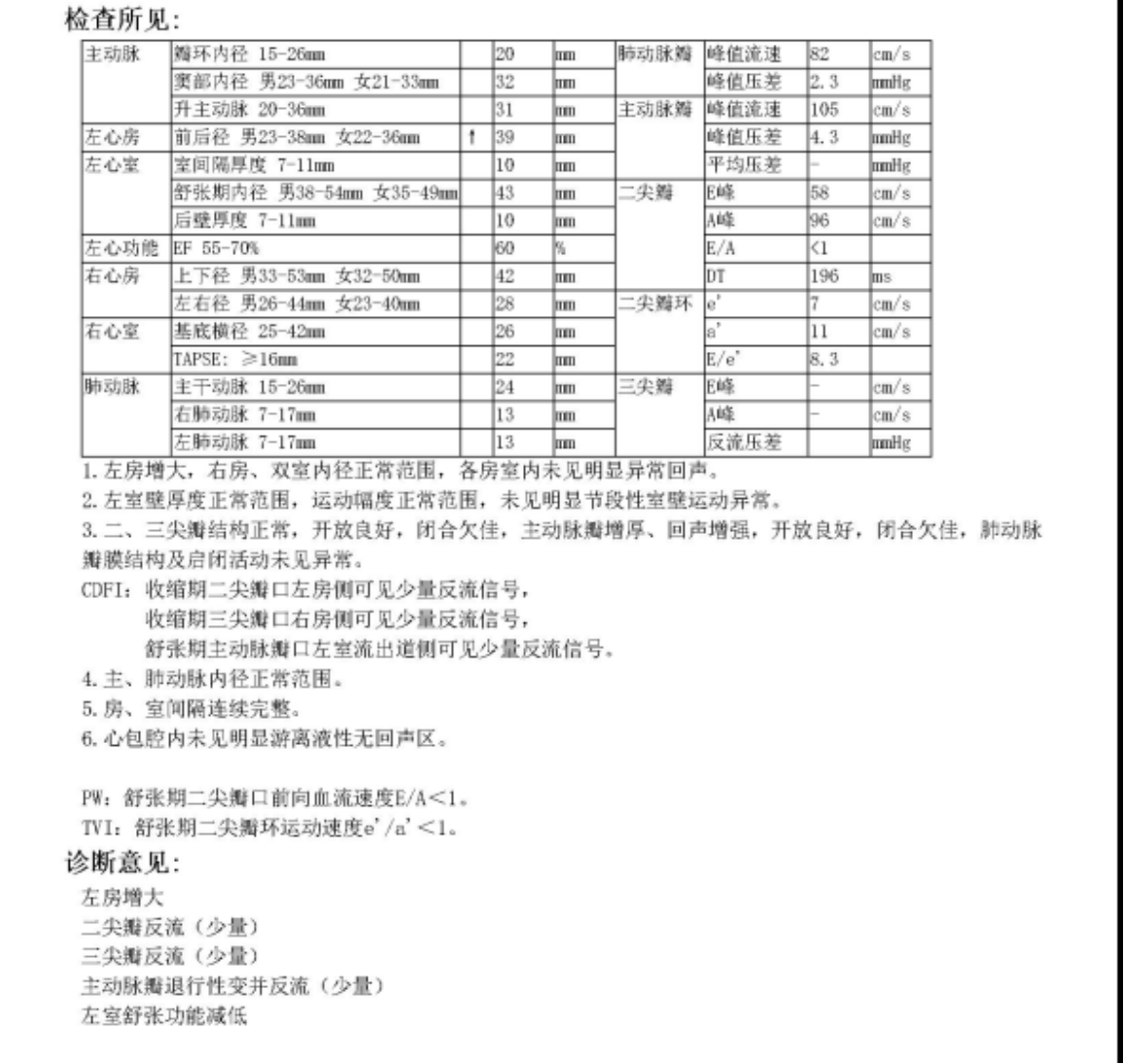

Supplement: Supplementary file 3 — Supplementary Data 2 [file 41746_2026_2648_MOESM3_ESM.zip › echocardiography_reports/155.png]

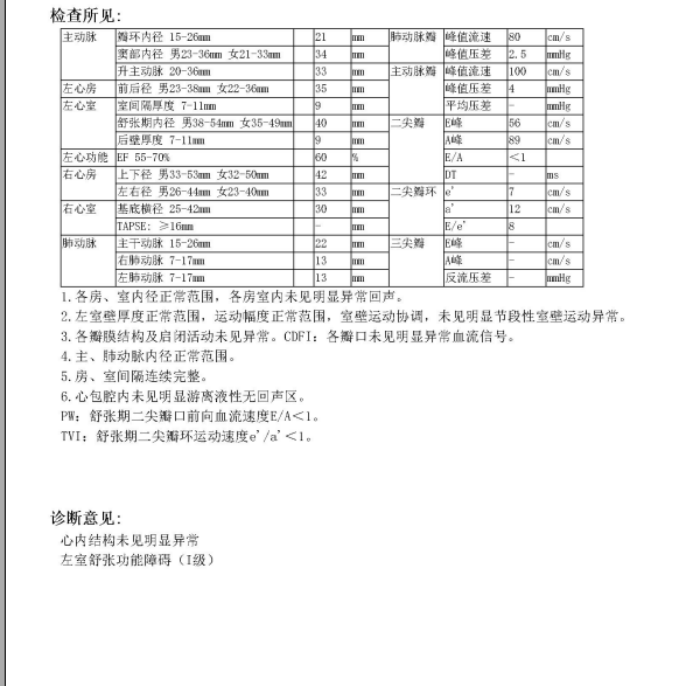

Supplement: Supplementary file 3 — Supplementary Data 2 [file 41746_2026_2648_MOESM3_ESM.zip › echocardiography_reports/156.png]

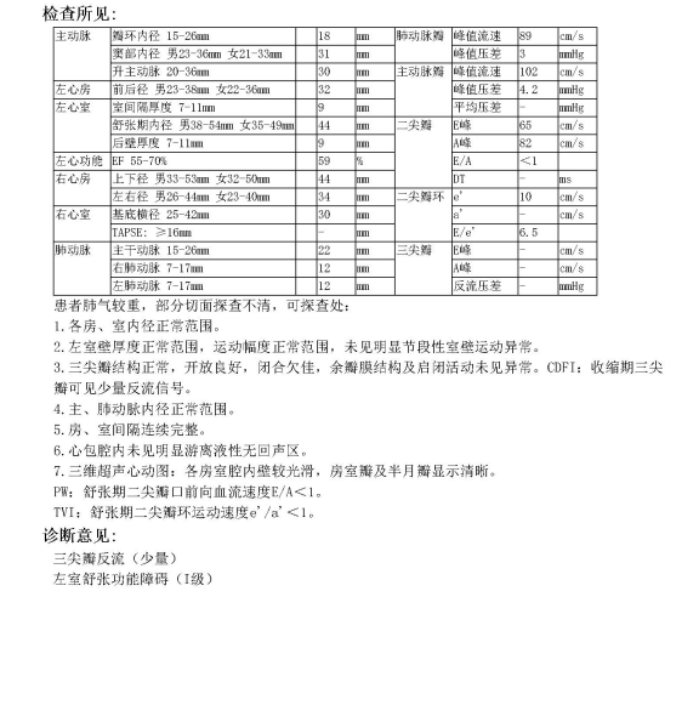

Supplement: Supplementary file 3 — Supplementary Data 2 [file 41746_2026_2648_MOESM3_ESM.zip › echocardiography_reports/157.png]

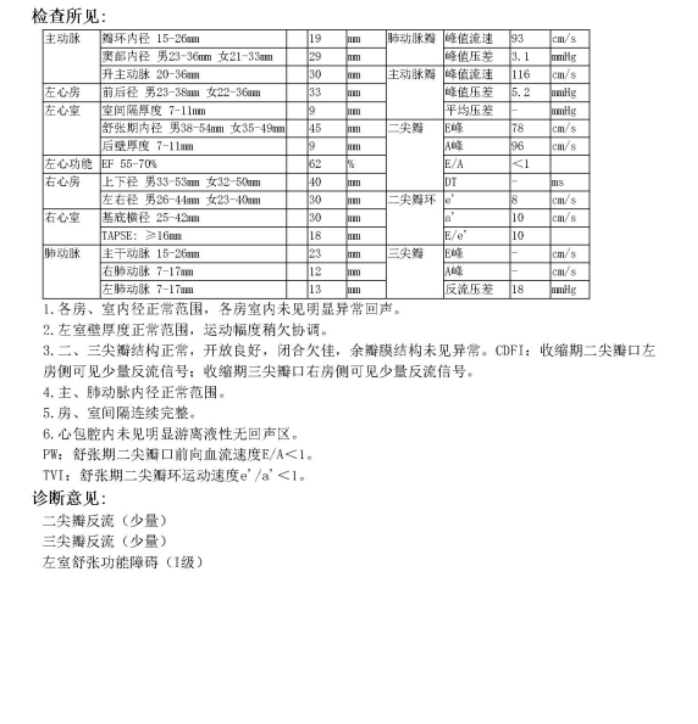

Supplement: Supplementary file 3 — Supplementary Data 2 [file 41746_2026_2648_MOESM3_ESM.zip › echocardiography_reports/158.png]

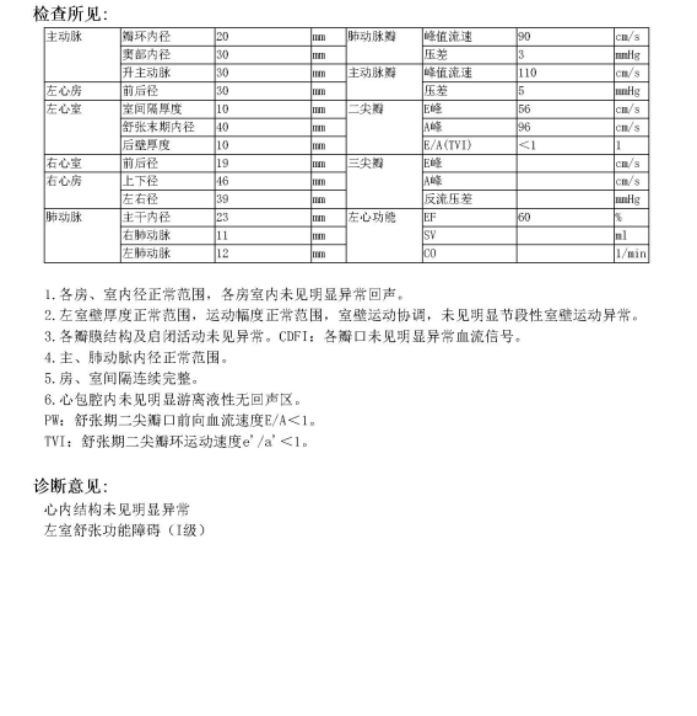

Supplement: Supplementary file 3 — Supplementary Data 2 [file 41746_2026_2648_MOESM3_ESM.zip › echocardiography_reports/159.png]

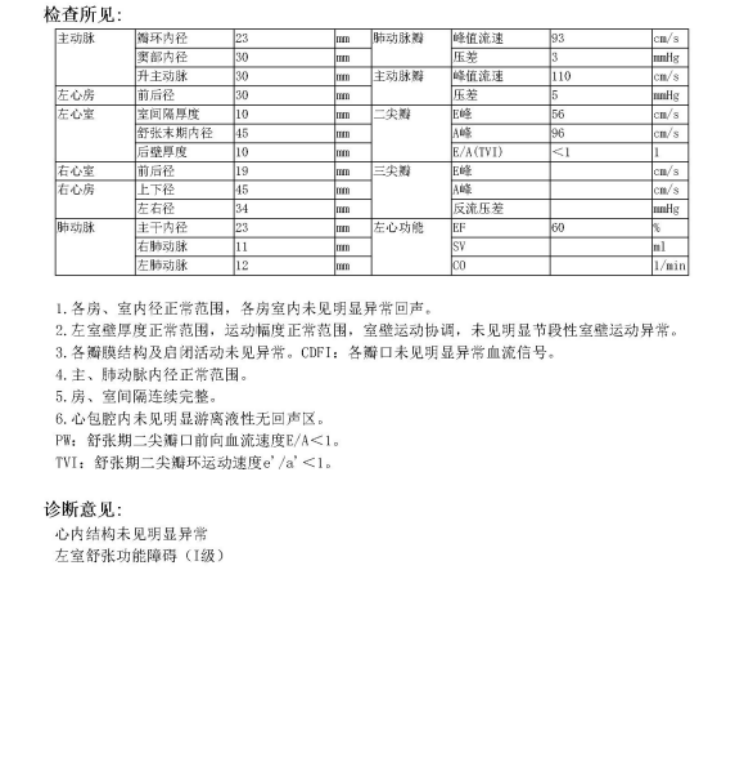

Supplement: Supplementary file 3 — Supplementary Data 2 [file 41746_2026_2648_MOESM3_ESM.zip › echocardiography_reports/160.png]

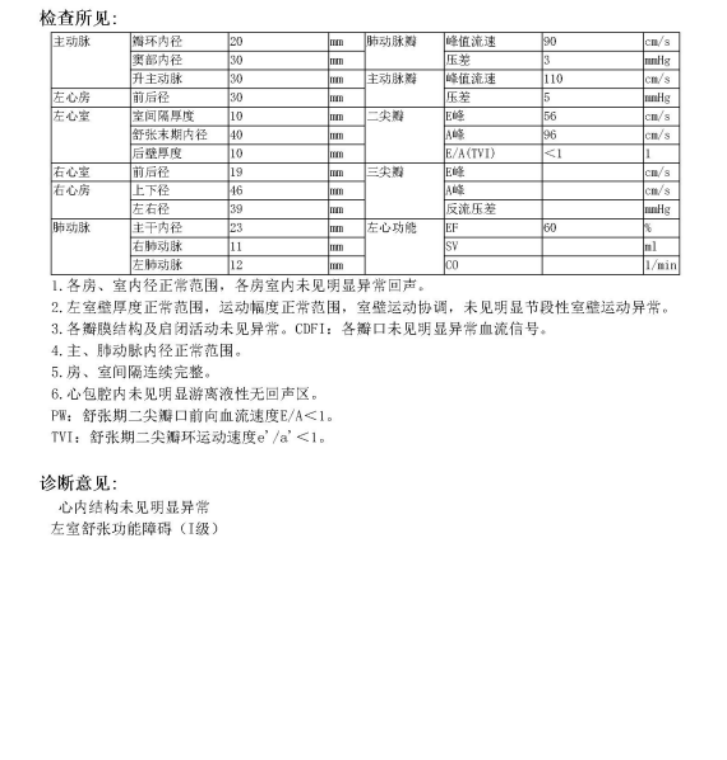

Supplement: Supplementary file 3 — Supplementary Data 2 [file 41746_2026_2648_MOESM3_ESM.zip › echocardiography_reports/161.png]

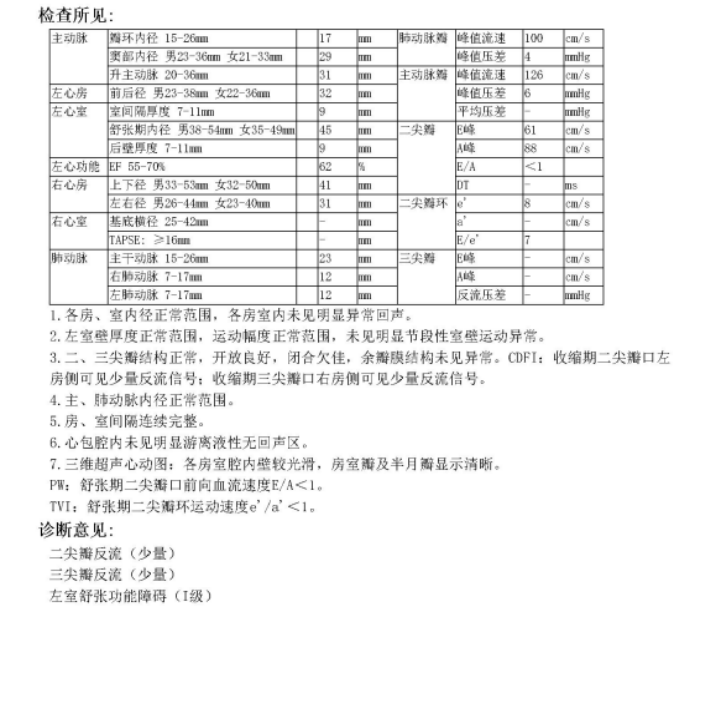

Supplement: Supplementary file 3 — Supplementary Data 2 [file 41746_2026_2648_MOESM3_ESM.zip › echocardiography_reports/162.png]

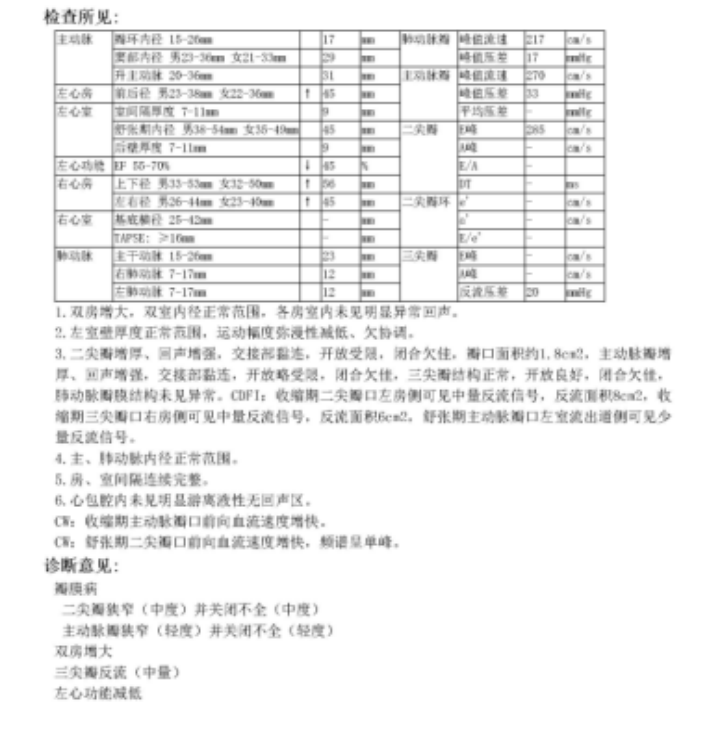

Supplement: Supplementary file 3 — Supplementary Data 2 [file 41746_2026_2648_MOESM3_ESM.zip › echocardiography_reports/163.png]

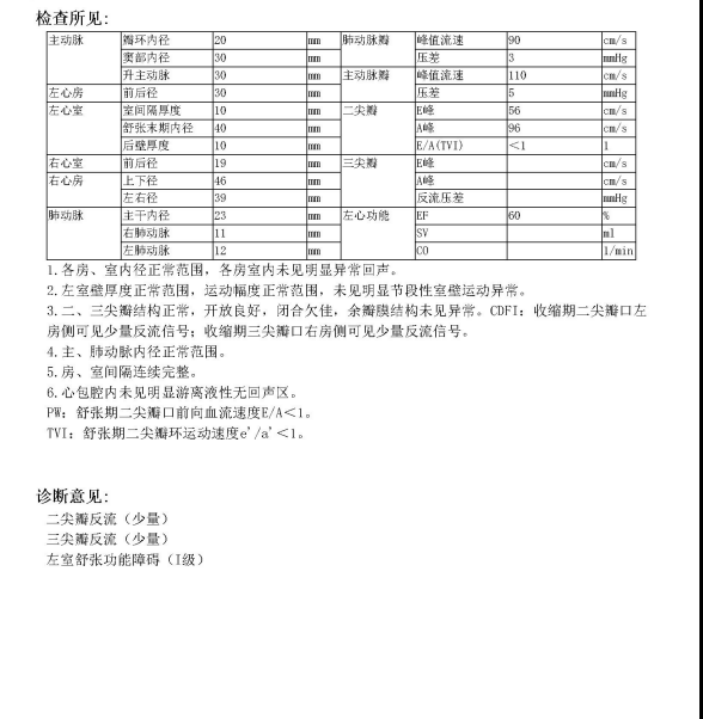

Supplement: Supplementary file 3 — Supplementary Data 2 [file 41746_2026_2648_MOESM3_ESM.zip › echocardiography_reports/164.png]

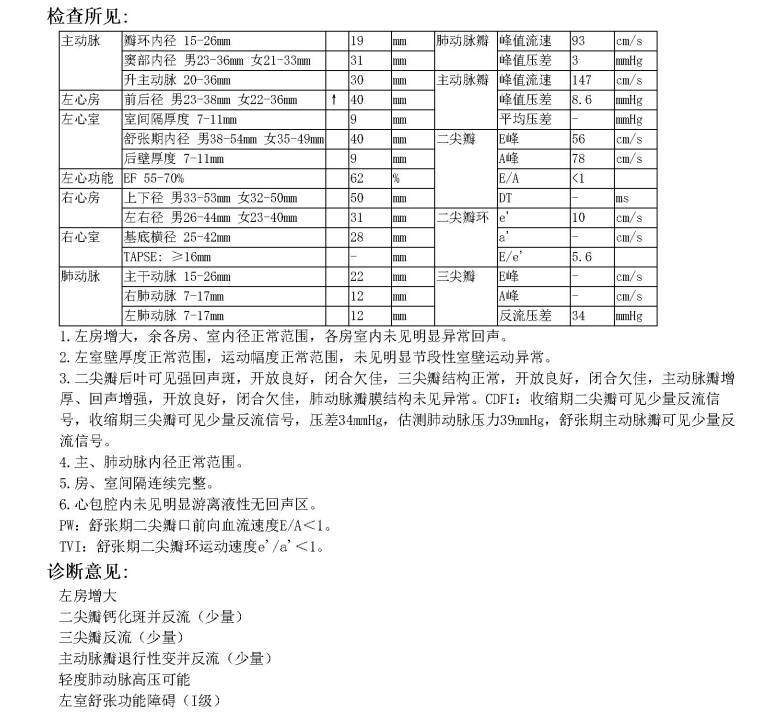

Supplement: Supplementary file 3 — Supplementary Data 2 [file 41746_2026_2648_MOESM3_ESM.zip › echocardiography_reports/165.png]

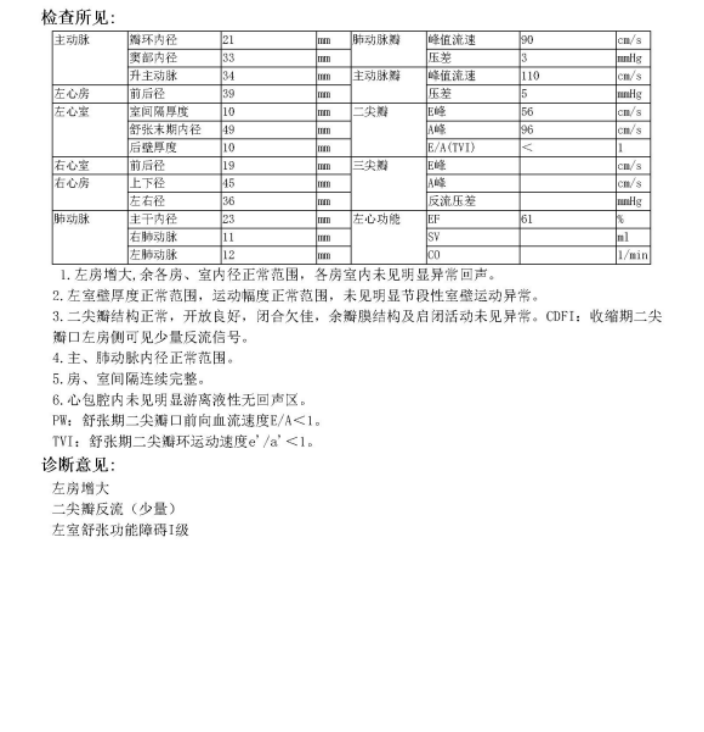

Supplement: Supplementary file 3 — Supplementary Data 2 [file 41746_2026_2648_MOESM3_ESM.zip › echocardiography_reports/166.png]

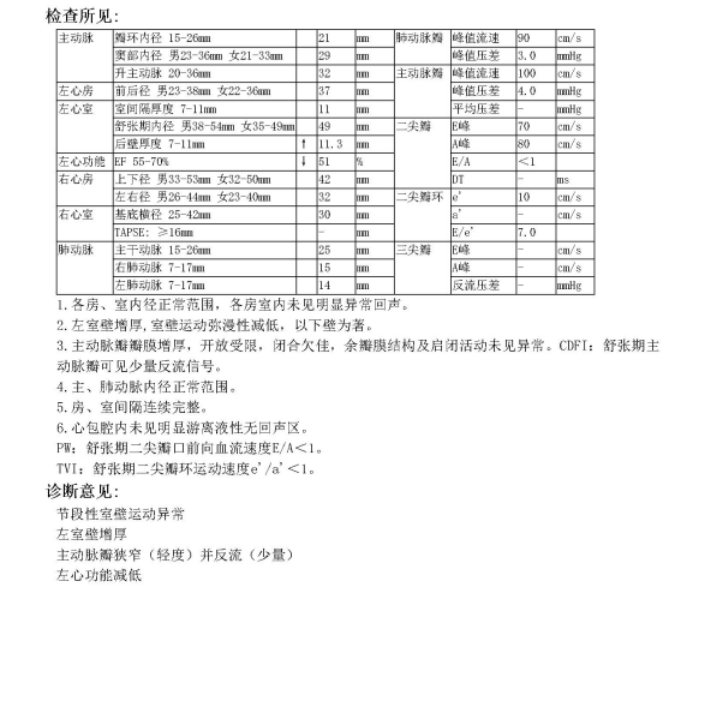

Supplement: Supplementary file 3 — Supplementary Data 2 [file 41746_2026_2648_MOESM3_ESM.zip › echocardiography_reports/167.png]

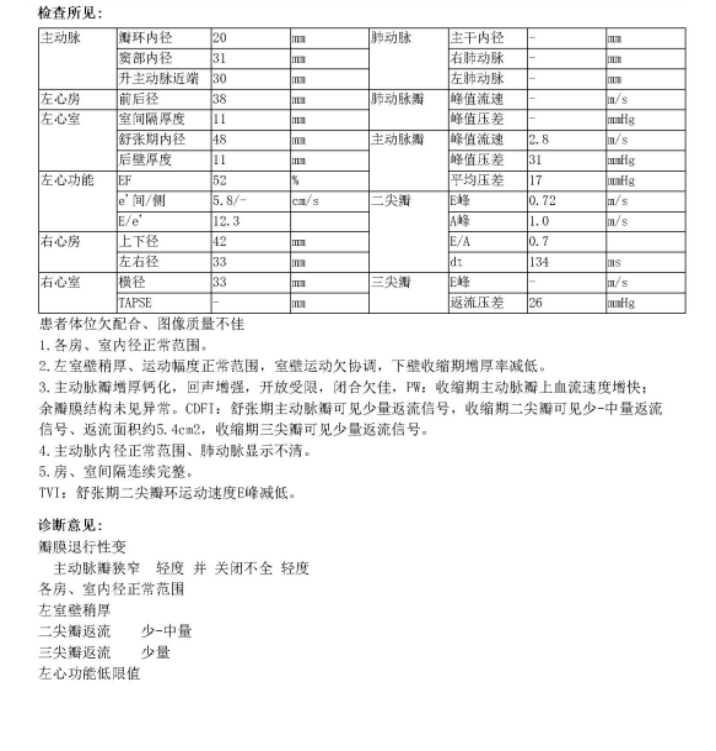

Supplement: Supplementary file 3 — Supplementary Data 2 [file 41746_2026_2648_MOESM3_ESM.zip › echocardiography_reports/168.png]

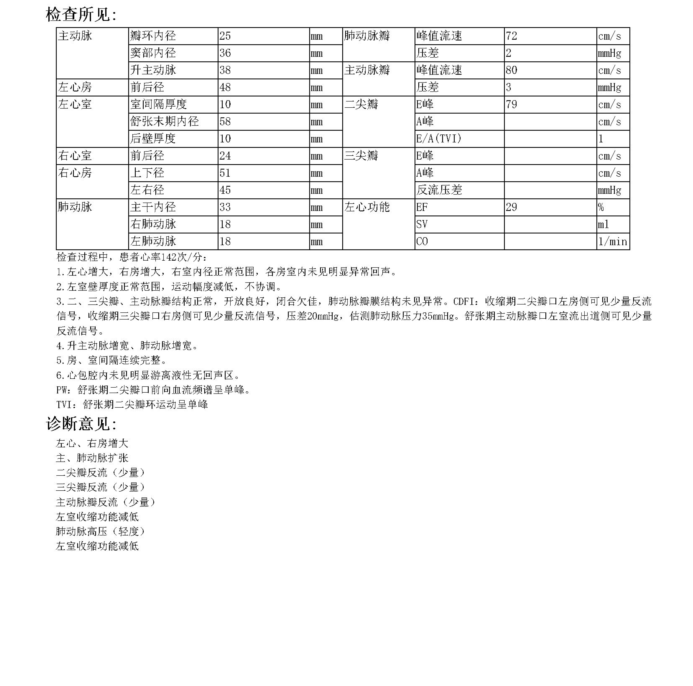

Supplement: Supplementary file 3 — Supplementary Data 2 [file 41746_2026_2648_MOESM3_ESM.zip › echocardiography_reports/169.png]

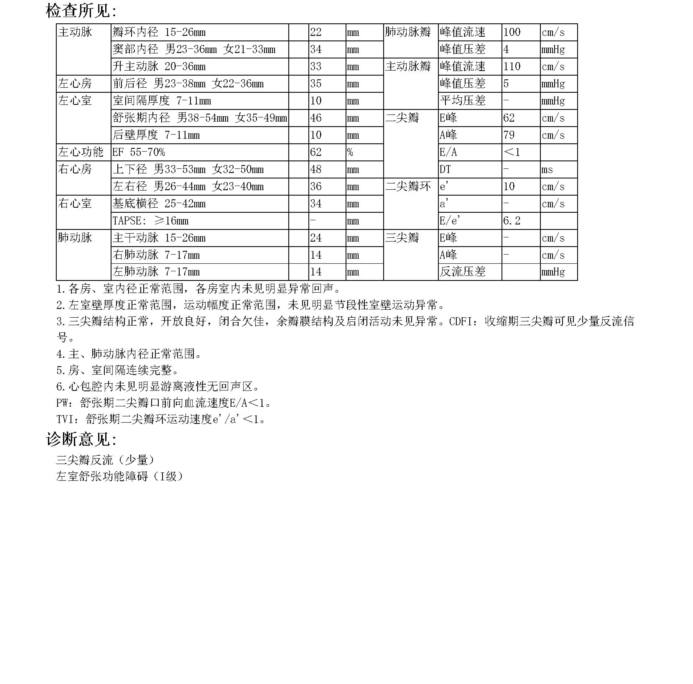

Supplement: Supplementary file 3 — Supplementary Data 2 [file 41746_2026_2648_MOESM3_ESM.zip › echocardiography_reports/170.png]

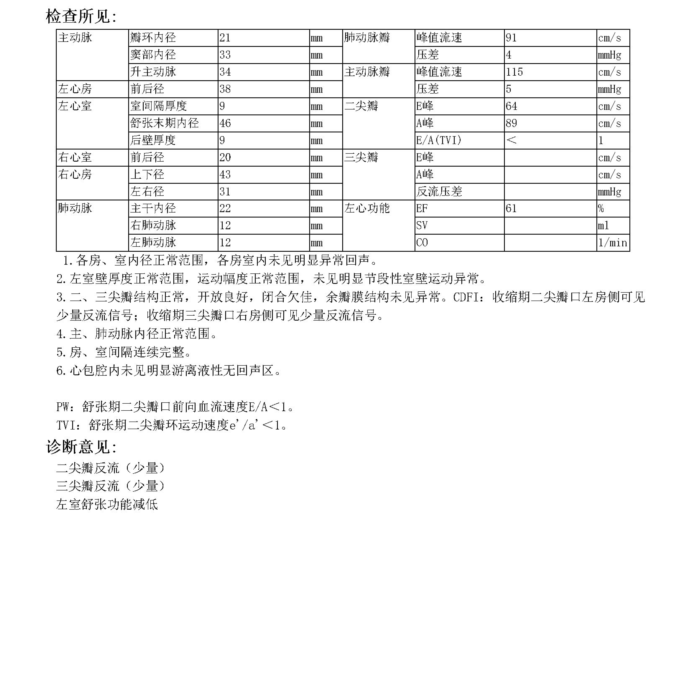

Supplement: Supplementary file 3 — Supplementary Data 2 [file 41746_2026_2648_MOESM3_ESM.zip › echocardiography_reports/171.png]

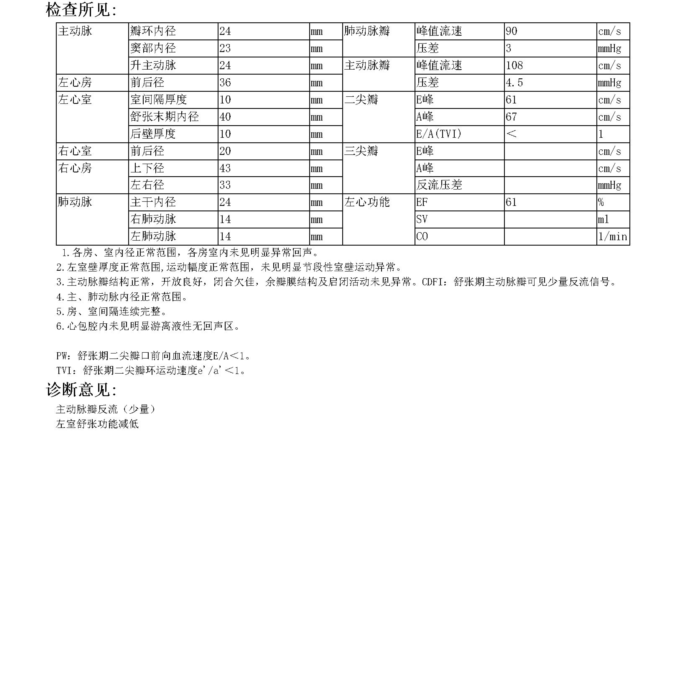

Supplement: Supplementary file 3 — Supplementary Data 2 [file 41746_2026_2648_MOESM3_ESM.zip › echocardiography_reports/172.png]

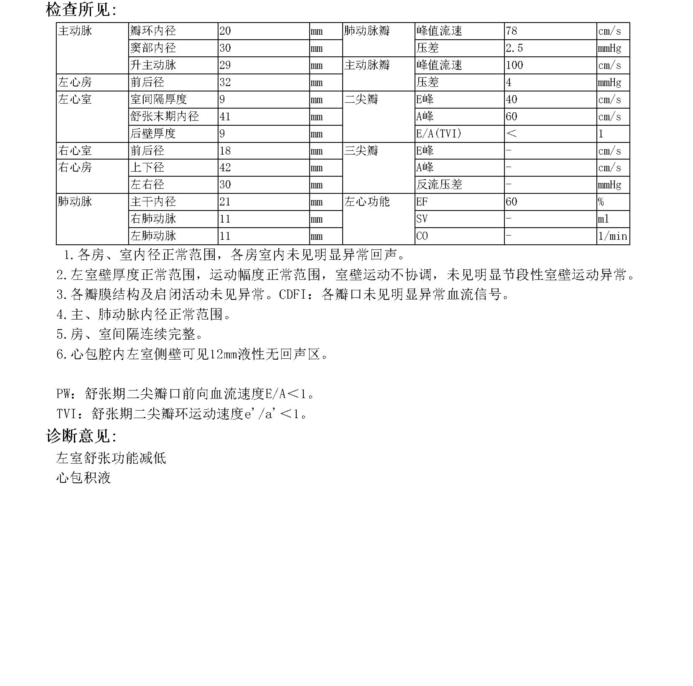

Supplement: Supplementary file 3 — Supplementary Data 2 [file 41746_2026_2648_MOESM3_ESM.zip › echocardiography_reports/173.png]

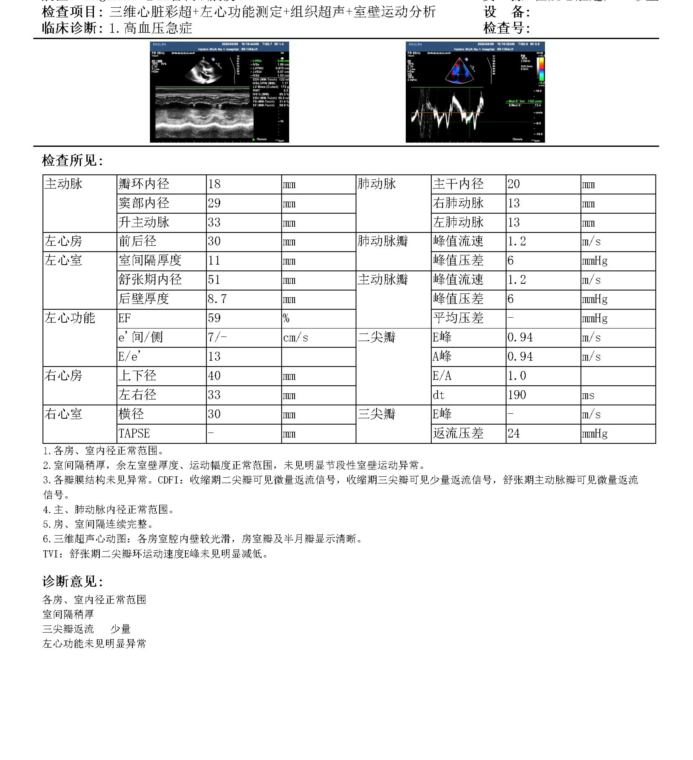

Supplement: Supplementary file 3 — Supplementary Data 2 [file 41746_2026_2648_MOESM3_ESM.zip › echocardiography_reports/174.png]

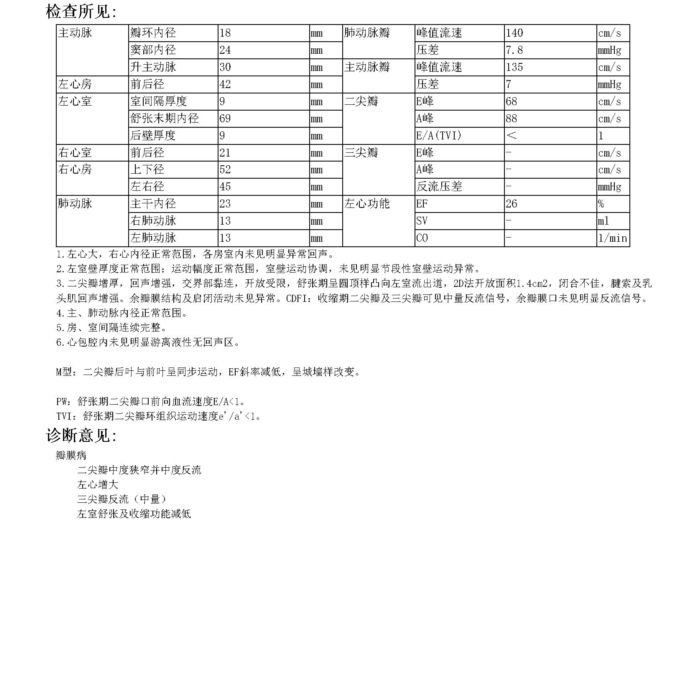

Supplement: Supplementary file 3 — Supplementary Data 2 [file 41746_2026_2648_MOESM3_ESM.zip › echocardiography_reports/175.png]

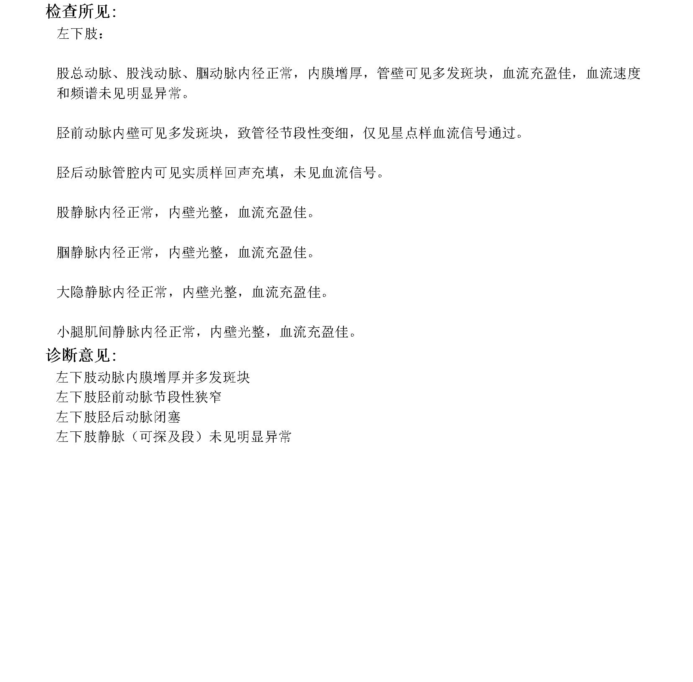

Supplement: Supplementary file 3 — Supplementary Data 2 [file 41746_2026_2648_MOESM3_ESM.zip › echocardiography_reports/176.png]

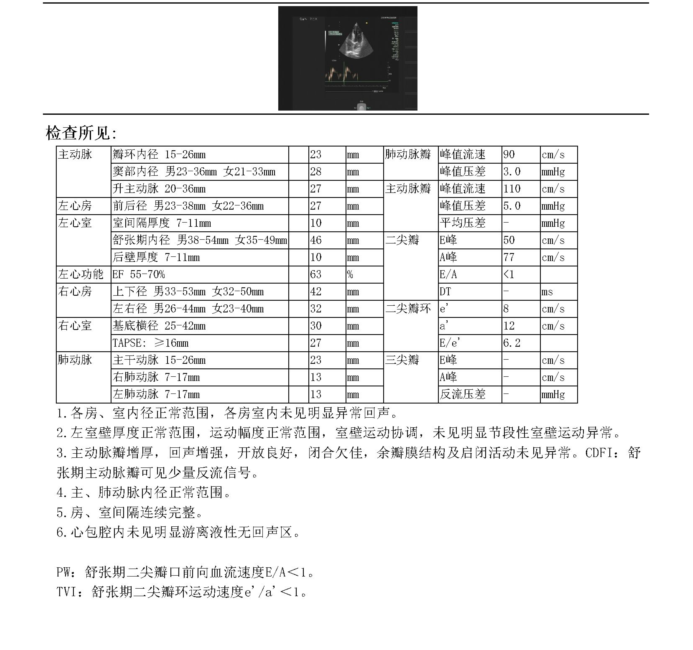

Supplement: Supplementary file 3 — Supplementary Data 2 [file 41746_2026_2648_MOESM3_ESM.zip › echocardiography_reports/177.png]

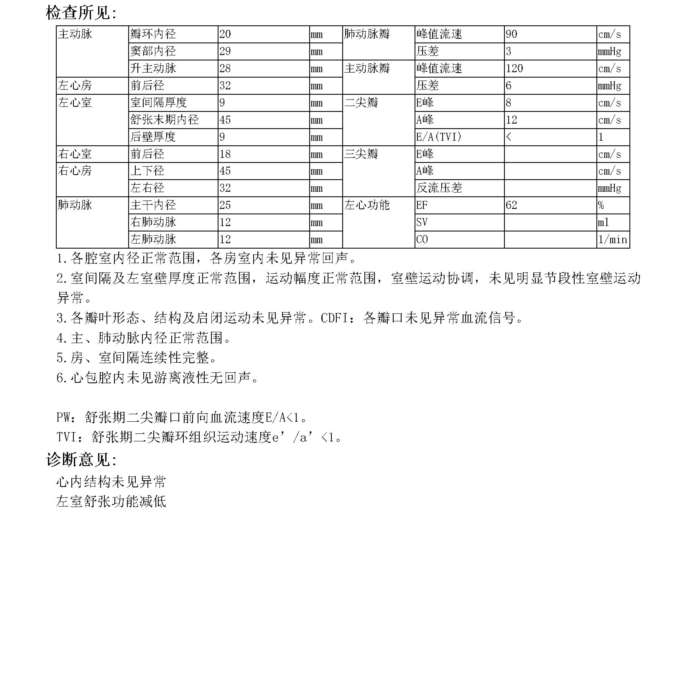

Supplement: Supplementary file 3 — Supplementary Data 2 [file 41746_2026_2648_MOESM3_ESM.zip › echocardiography_reports/178.png]

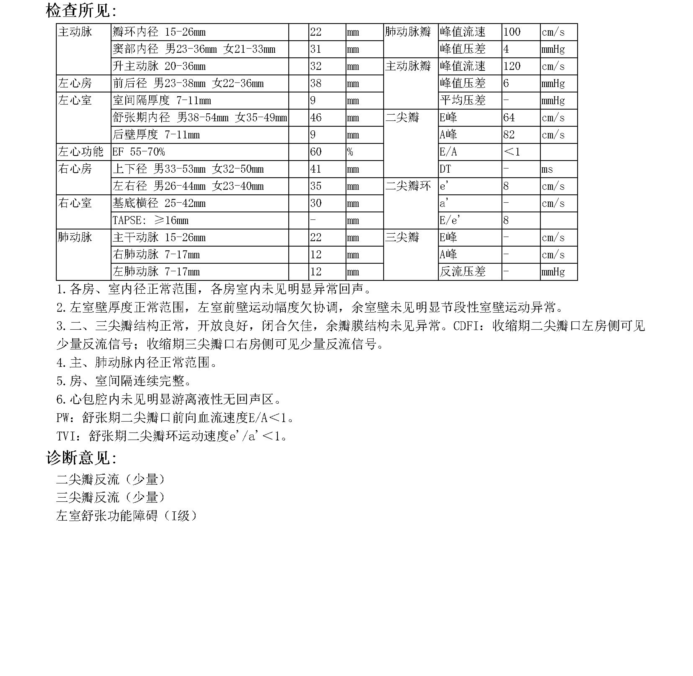

Supplement: Supplementary file 3 — Supplementary Data 2 [file 41746_2026_2648_MOESM3_ESM.zip › echocardiography_reports/179.png]

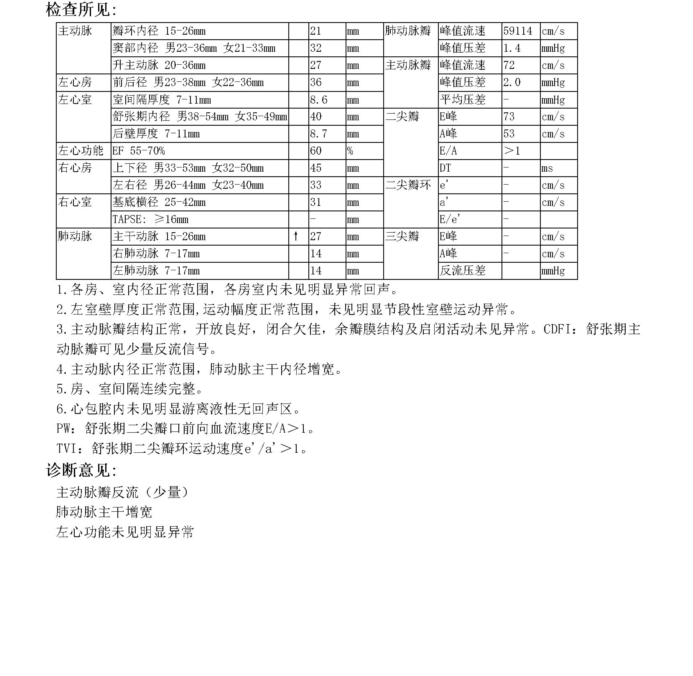

Supplement: Supplementary file 3 — Supplementary Data 2 [file 41746_2026_2648_MOESM3_ESM.zip › echocardiography_reports/180.png]

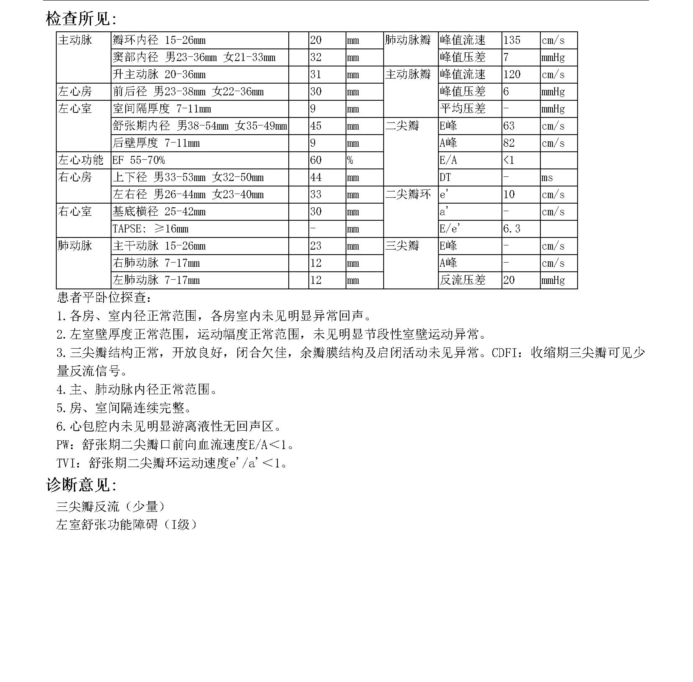

Supplement: Supplementary file 3 — Supplementary Data 2 [file 41746_2026_2648_MOESM3_ESM.zip › echocardiography_reports/181.png]

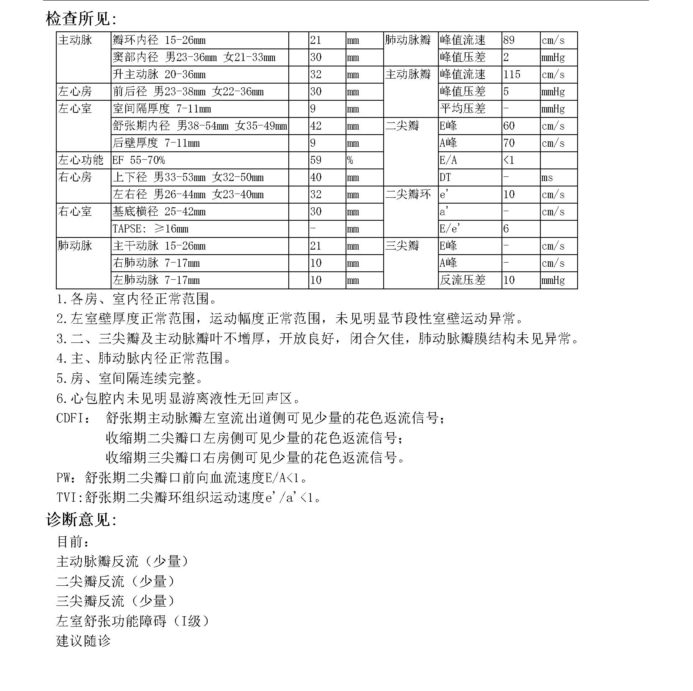

Supplement: Supplementary file 3 — Supplementary Data 2 [file 41746_2026_2648_MOESM3_ESM.zip › echocardiography_reports/182.png]

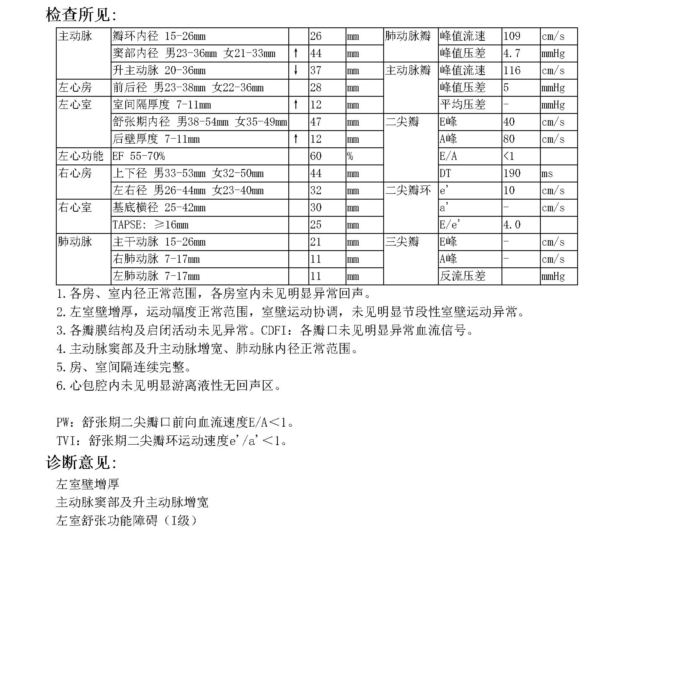

Supplement: Supplementary file 3 — Supplementary Data 2 [file 41746_2026_2648_MOESM3_ESM.zip › echocardiography_reports/183.png]

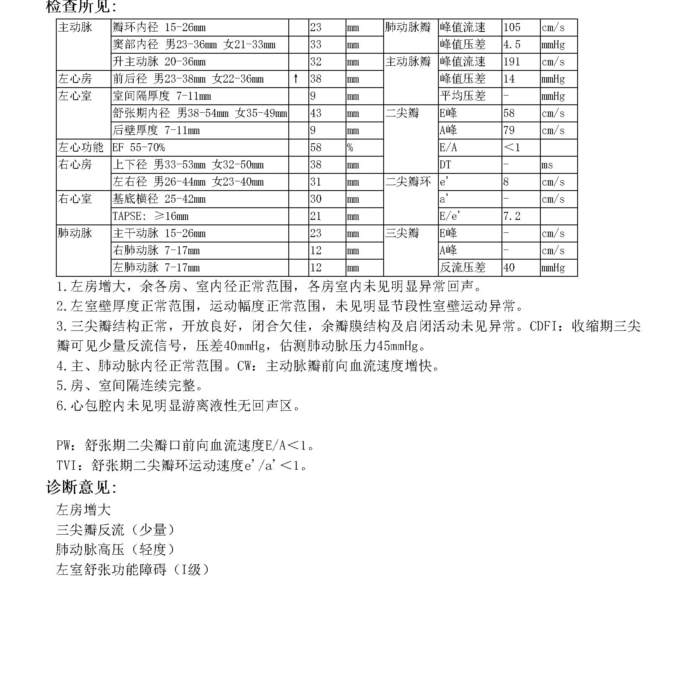

Supplement: Supplementary file 3 — Supplementary Data 2 [file 41746_2026_2648_MOESM3_ESM.zip › echocardiography_reports/184.png]

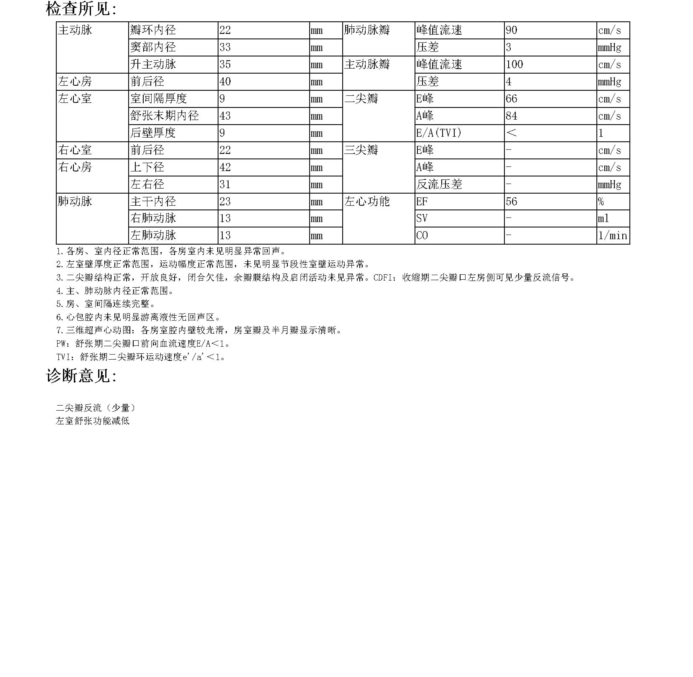

Supplement: Supplementary file 3 — Supplementary Data 2 [file 41746_2026_2648_MOESM3_ESM.zip › echocardiography_reports/185.png]

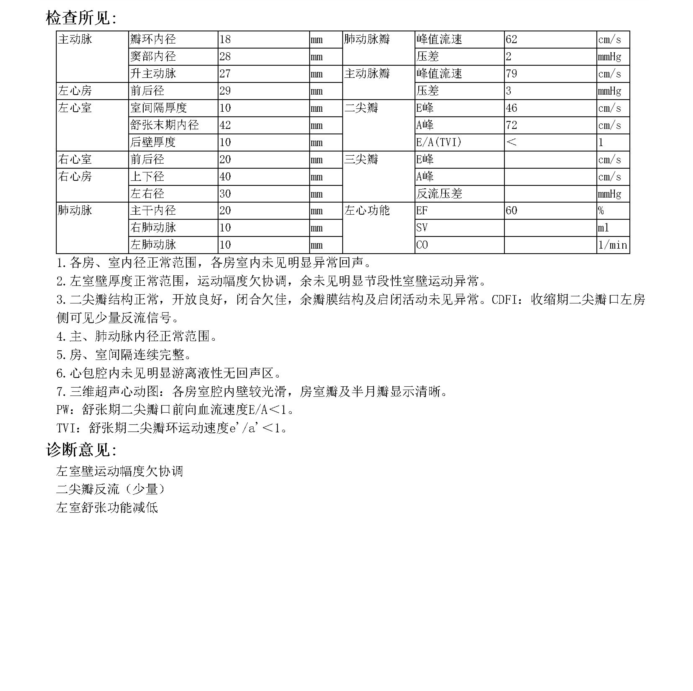

Supplement: Supplementary file 3 — Supplementary Data 2 [file 41746_2026_2648_MOESM3_ESM.zip › echocardiography_reports/186.png]

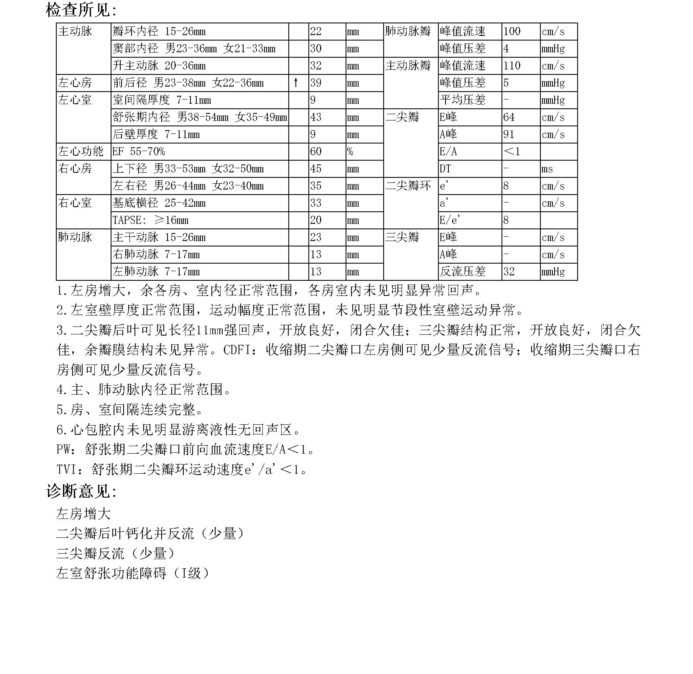

Supplement: Supplementary file 3 — Supplementary Data 2 [file 41746_2026_2648_MOESM3_ESM.zip › echocardiography_reports/187.png]

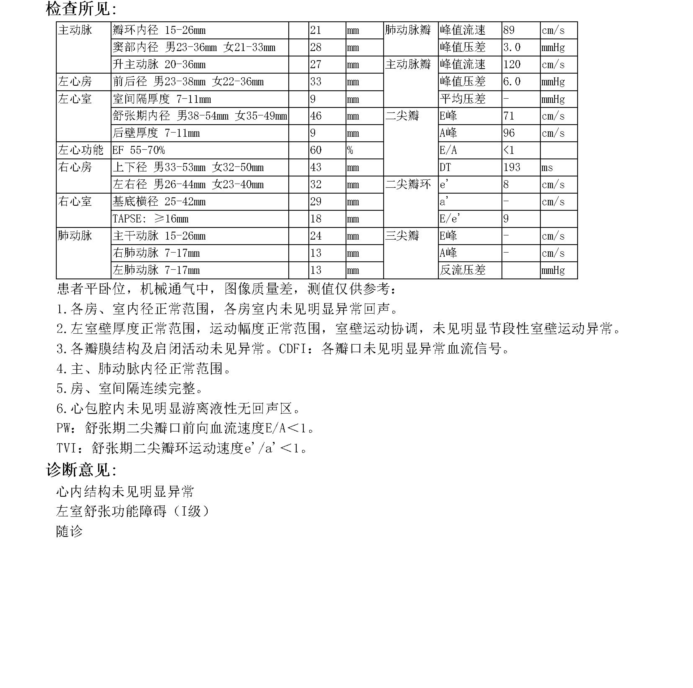

Supplement: Supplementary file 3 — Supplementary Data 2 [file 41746_2026_2648_MOESM3_ESM.zip › echocardiography_reports/188.png]

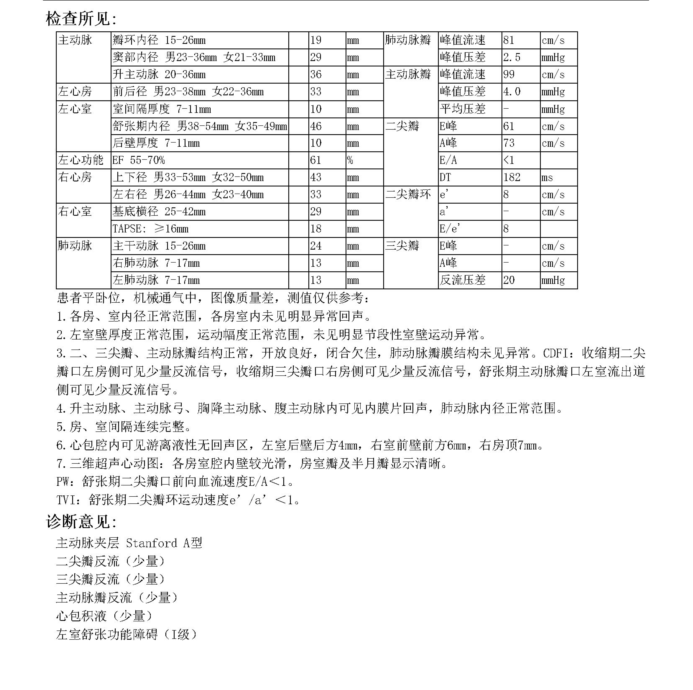

Supplement: Supplementary file 3 — Supplementary Data 2 [file 41746_2026_2648_MOESM3_ESM.zip › echocardiography_reports/189.png]

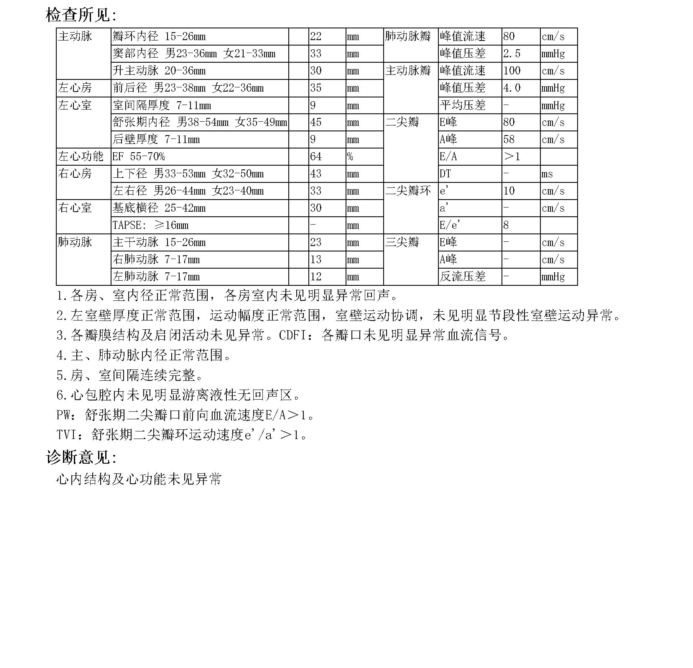

Supplement: Supplementary file 3 — Supplementary Data 2 [file 41746_2026_2648_MOESM3_ESM.zip › echocardiography_reports/190.png]

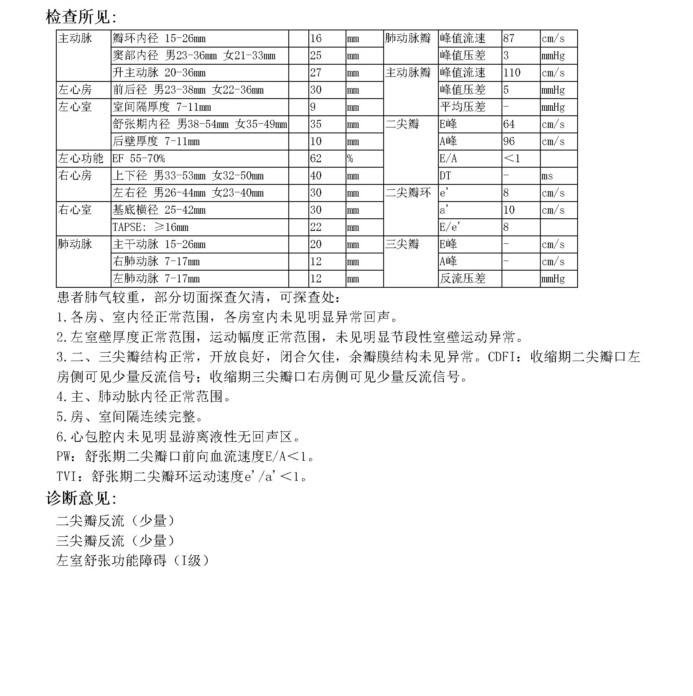

Supplement: Supplementary file 3 — Supplementary Data 2 [file 41746_2026_2648_MOESM3_ESM.zip › echocardiography_reports/191.png]

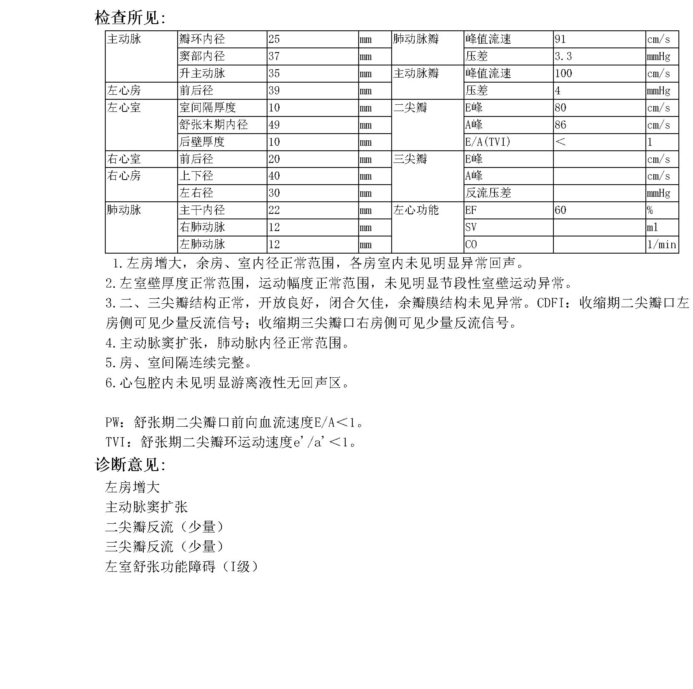

Supplement: Supplementary file 3 — Supplementary Data 2 [file 41746_2026_2648_MOESM3_ESM.zip › echocardiography_reports/192.png]

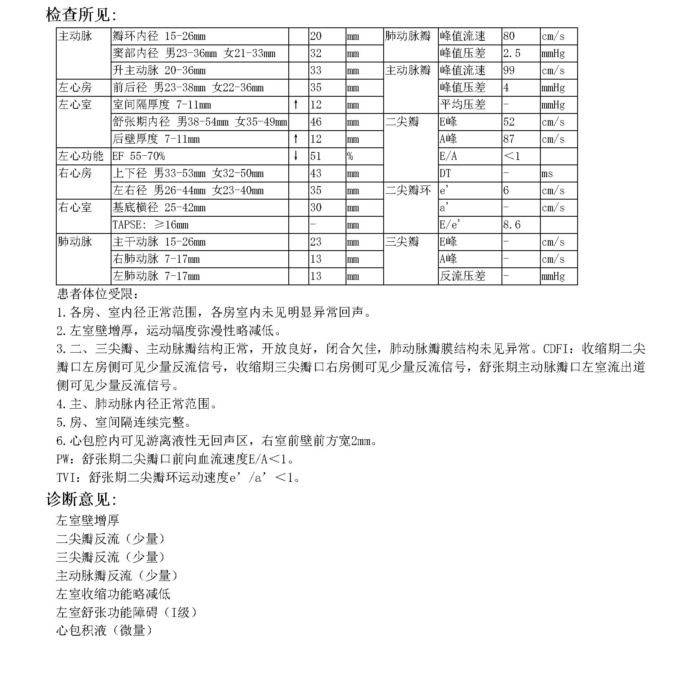

Supplement: Supplementary file 3 — Supplementary Data 2 [file 41746_2026_2648_MOESM3_ESM.zip › echocardiography_reports/193.png]

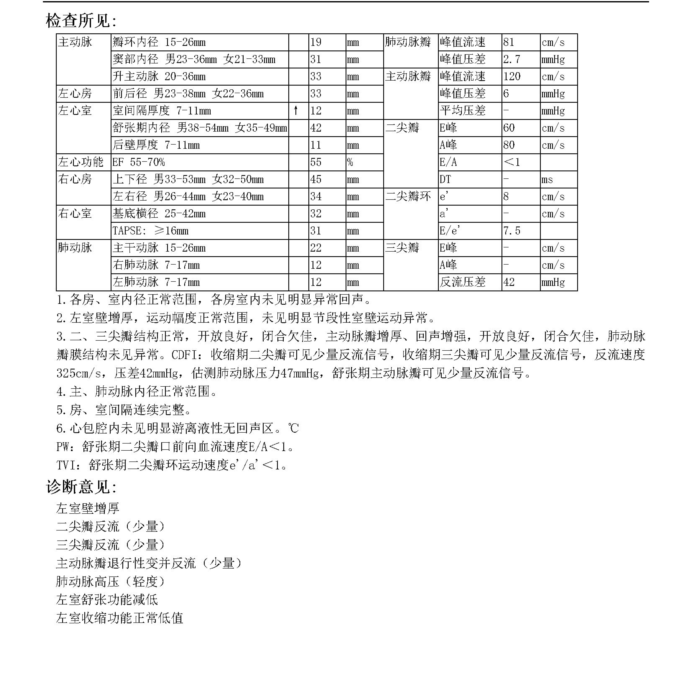

Supplement: Supplementary file 3 — Supplementary Data 2 [file 41746_2026_2648_MOESM3_ESM.zip › echocardiography_reports/194.png]

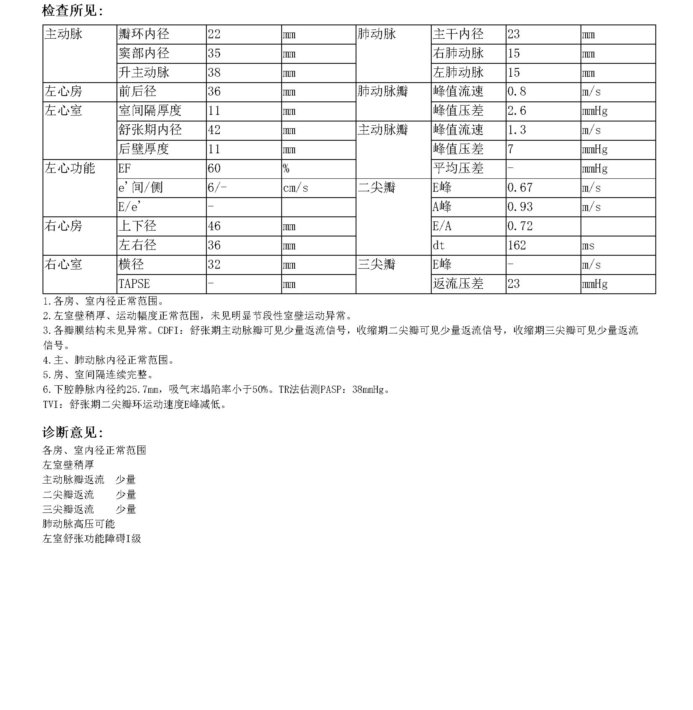

Supplement: Supplementary file 3 — Supplementary Data 2 [file 41746_2026_2648_MOESM3_ESM.zip › echocardiography_reports/195.png]

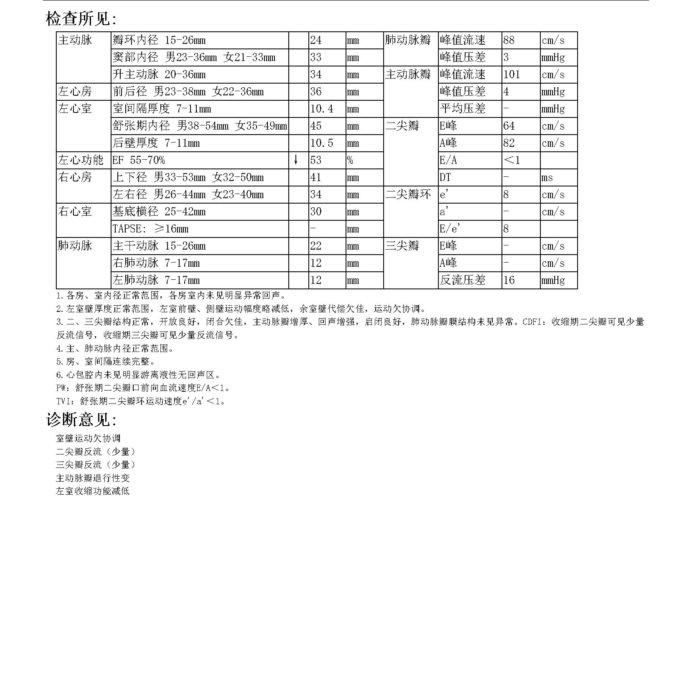

Supplement: Supplementary file 3 — Supplementary Data 2 [file 41746_2026_2648_MOESM3_ESM.zip › echocardiography_reports/196.png]

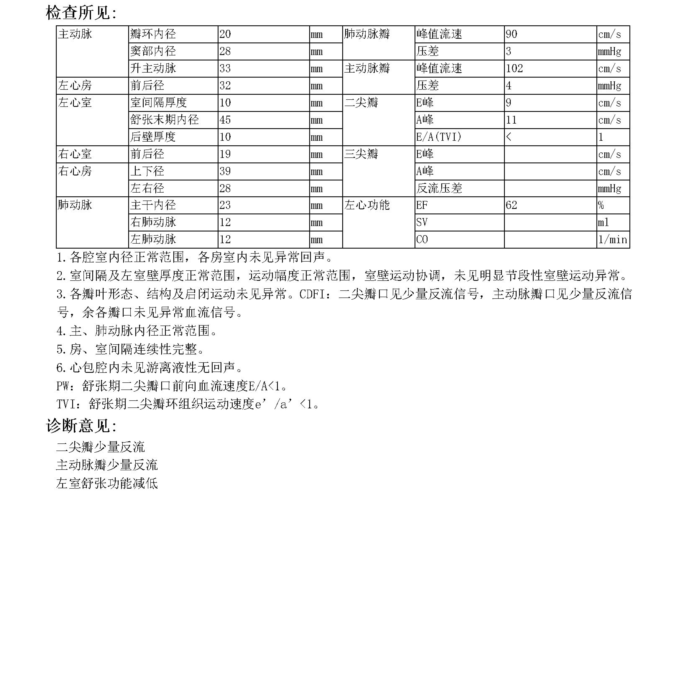

Supplement: Supplementary file 3 — Supplementary Data 2 [file 41746_2026_2648_MOESM3_ESM.zip › echocardiography_reports/197.png]

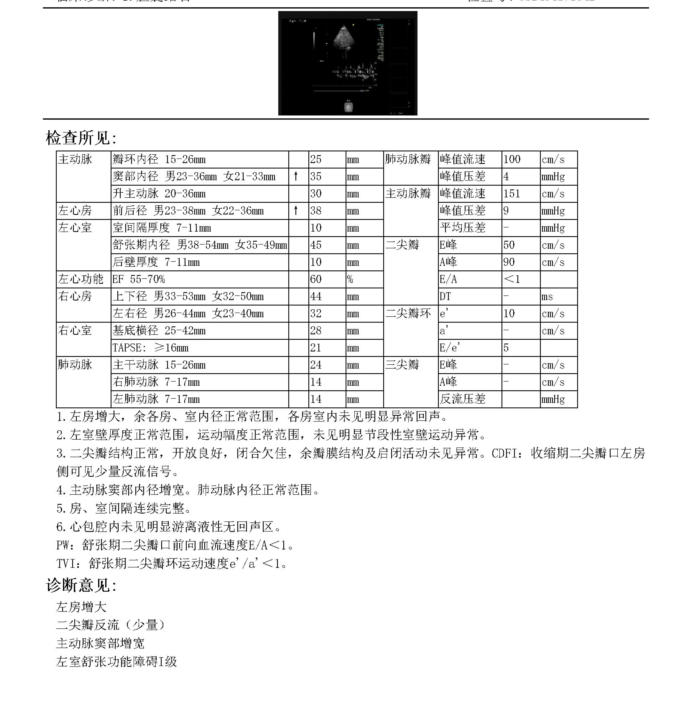

Supplement: Supplementary file 3 — Supplementary Data 2 [file 41746_2026_2648_MOESM3_ESM.zip › echocardiography_reports/198.png]

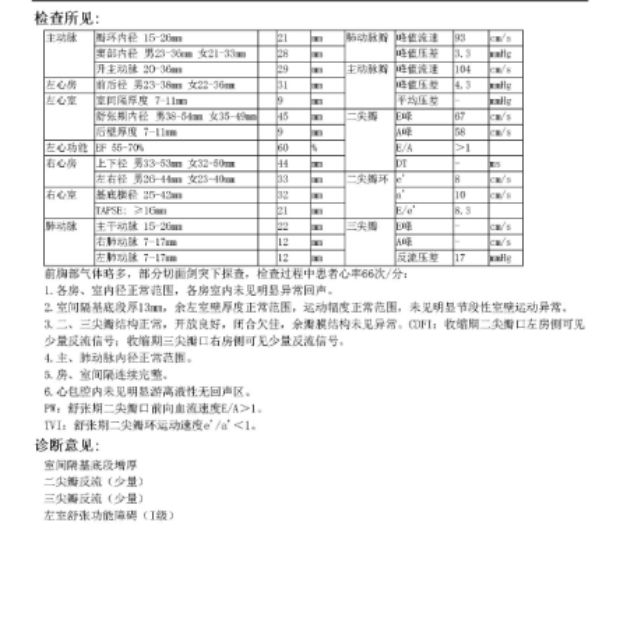

Supplement: Supplementary file 3 — Supplementary Data 2 [file 41746_2026_2648_MOESM3_ESM.zip › echocardiography_reports/199.png]

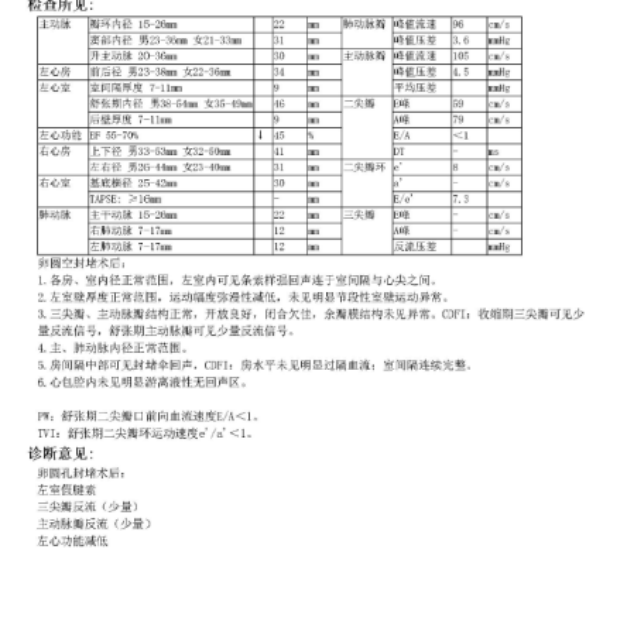

Supplement: Supplementary file 3 — Supplementary Data 2 [file 41746_2026_2648_MOESM3_ESM.zip › echocardiography_reports/200.png]

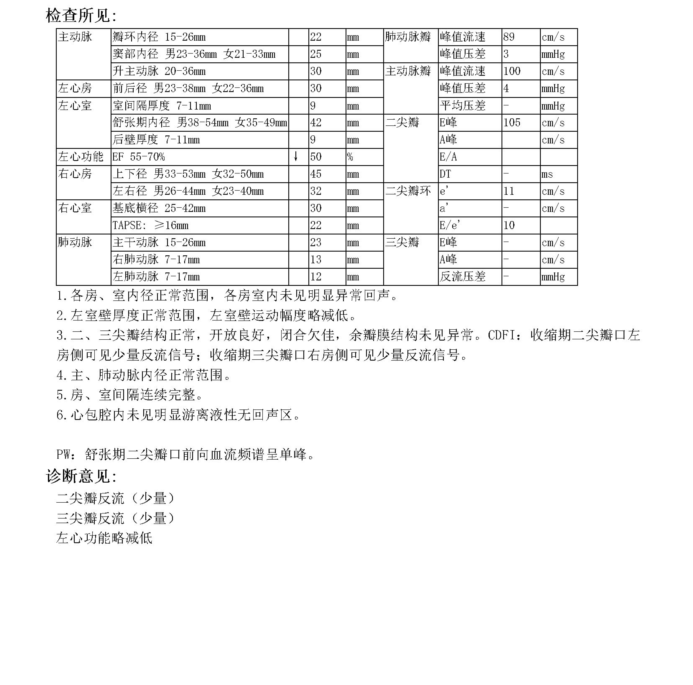

Supplement: Supplementary file 3 — Supplementary Data 2 [file 41746_2026_2648_MOESM3_ESM.zip › echocardiography_reports/201.png]

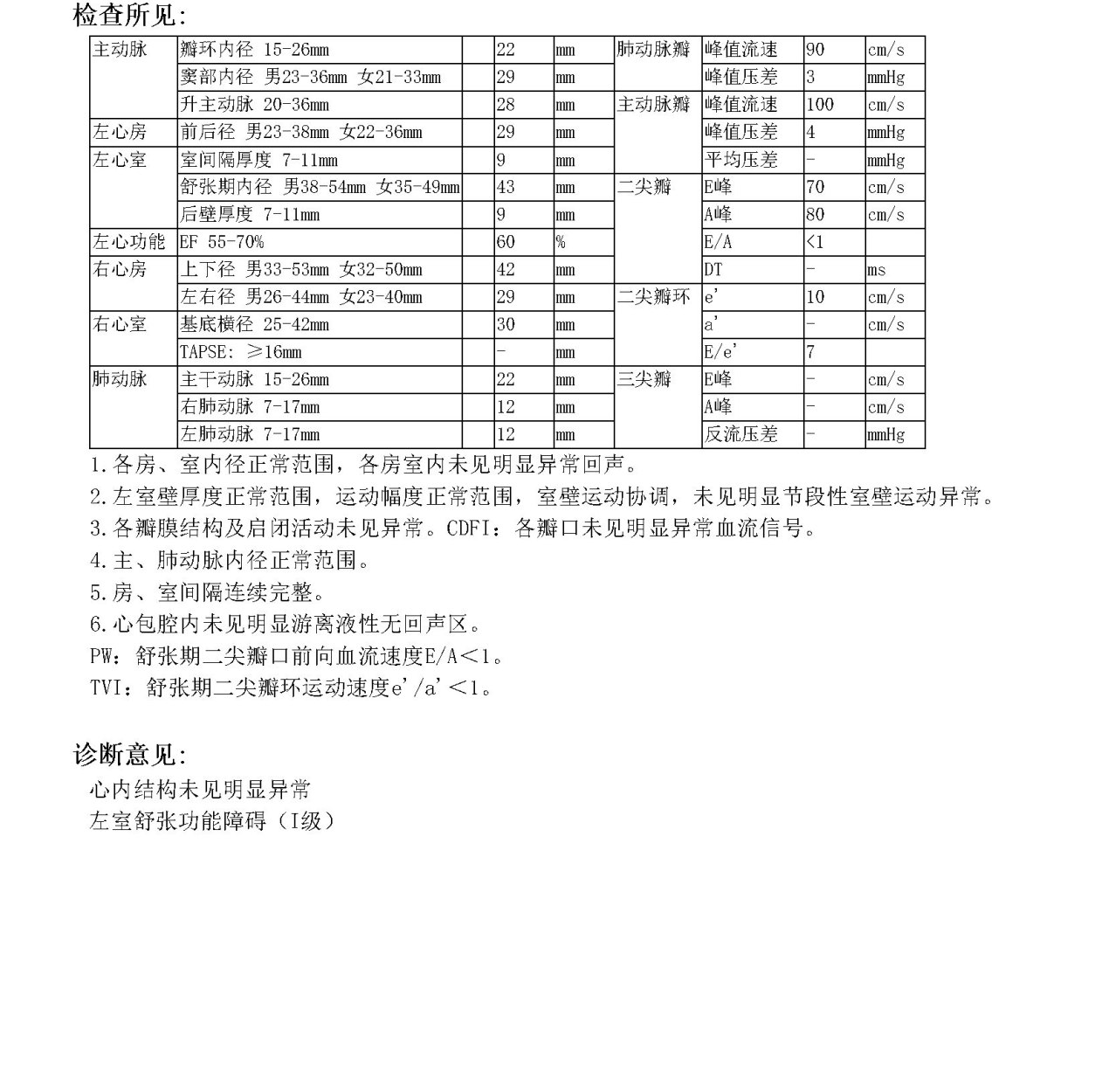

Supplement: Supplementary file 3 — Supplementary Data 2 [file 41746_2026_2648_MOESM3_ESM.zip › echocardiography_reports/202.png]

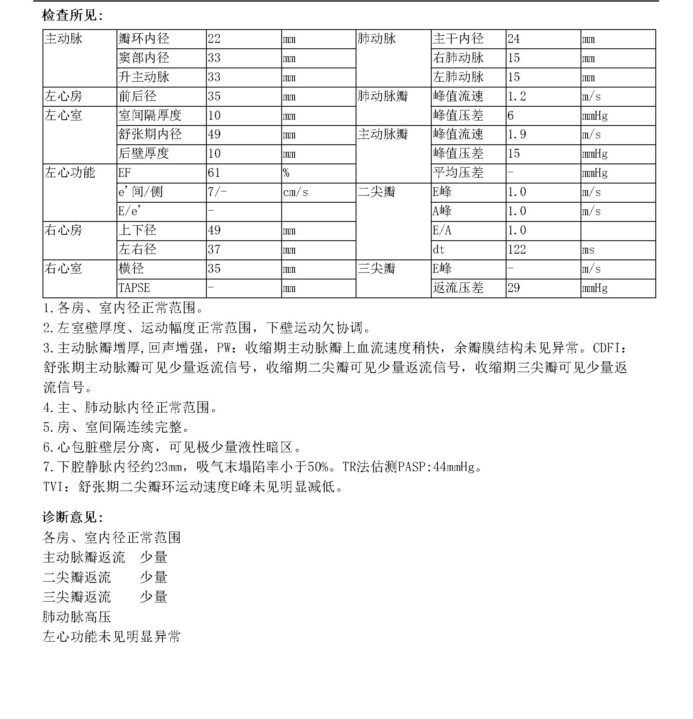

Supplement: Supplementary file 3 — Supplementary Data 2 [file 41746_2026_2648_MOESM3_ESM.zip › echocardiography_reports/203.png]

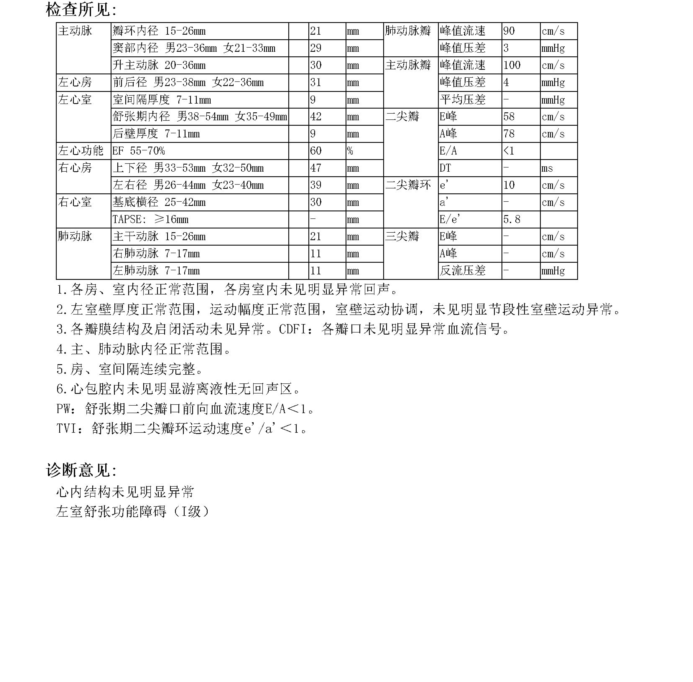

Supplement: Supplementary file 3 — Supplementary Data 2 [file 41746_2026_2648_MOESM3_ESM.zip › echocardiography_reports/204.png]

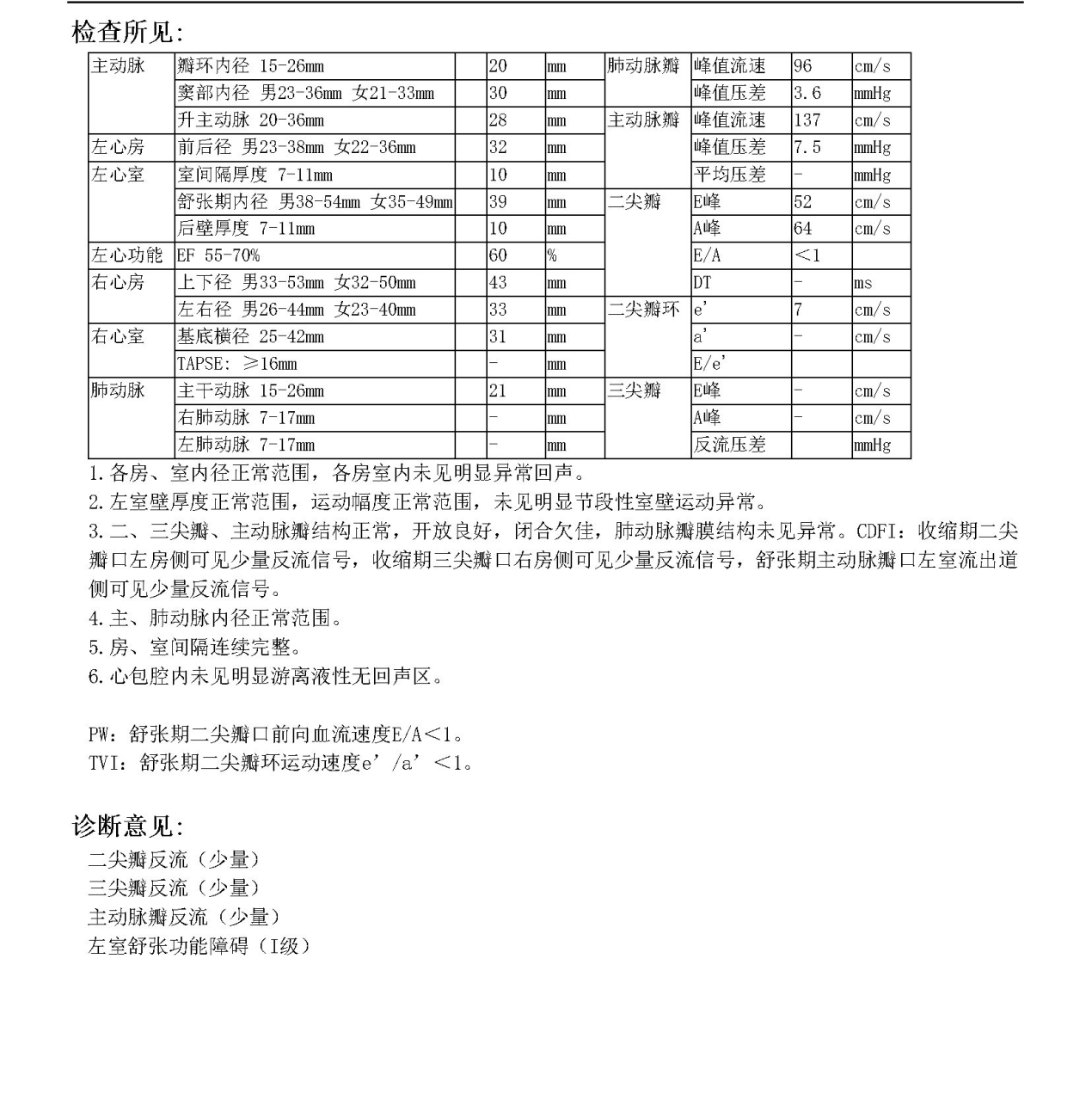

Supplement: Supplementary file 3 — Supplementary Data 2 [file 41746_2026_2648_MOESM3_ESM.zip › echocardiography_reports/205.png]

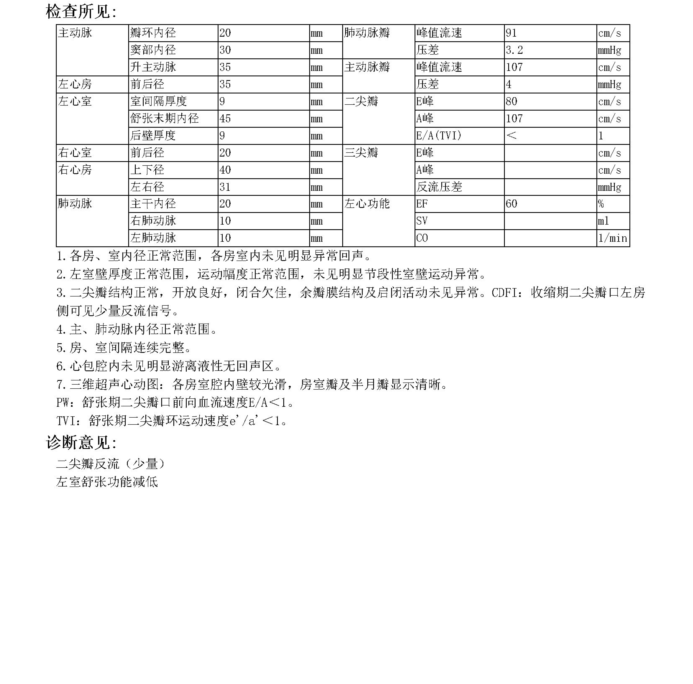

Supplement: Supplementary file 3 — Supplementary Data 2 [file 41746_2026_2648_MOESM3_ESM.zip › echocardiography_reports/206.png]

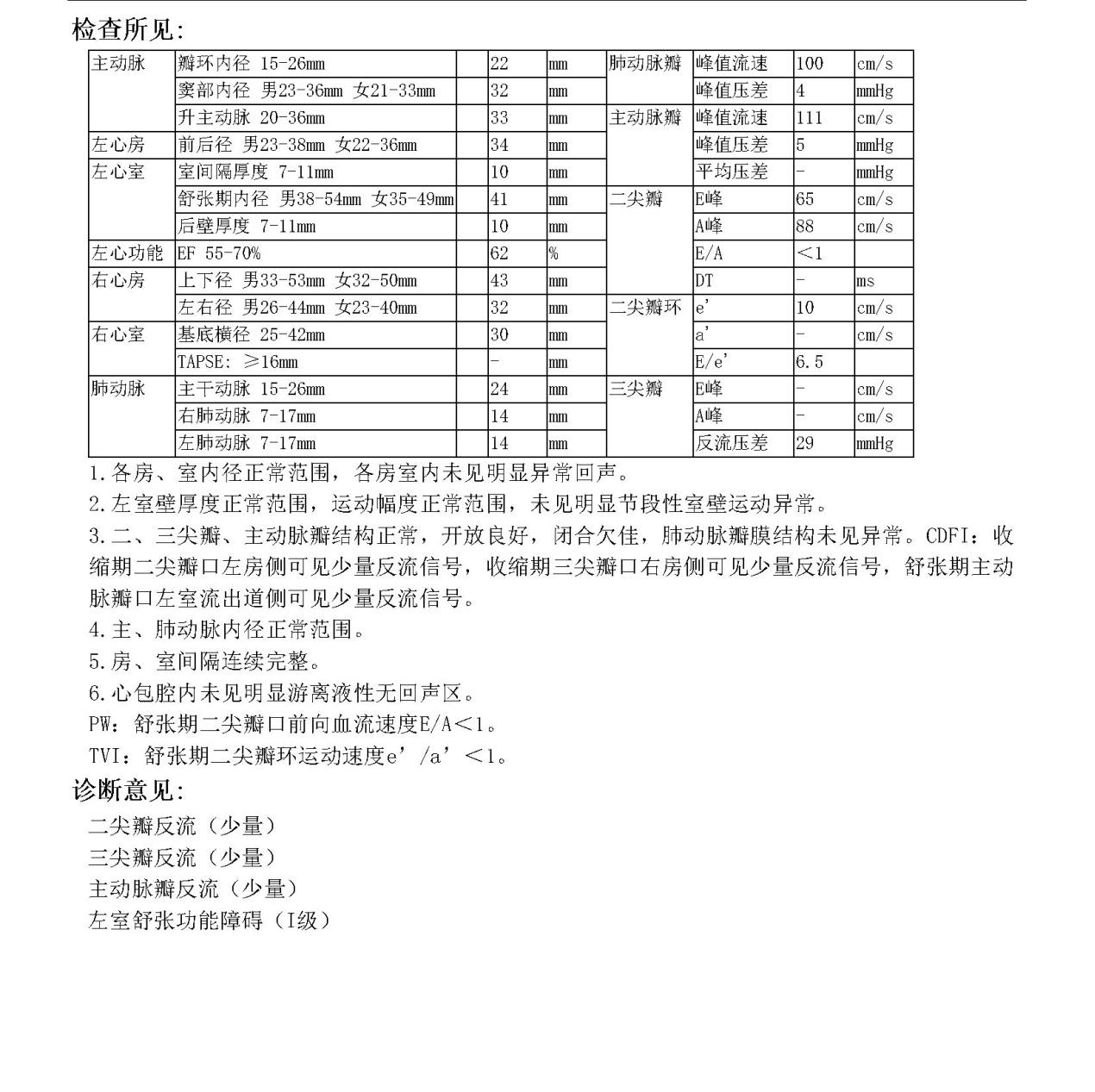

Supplement: Supplementary file 3 — Supplementary Data 2 [file 41746_2026_2648_MOESM3_ESM.zip › echocardiography_reports/207.png]

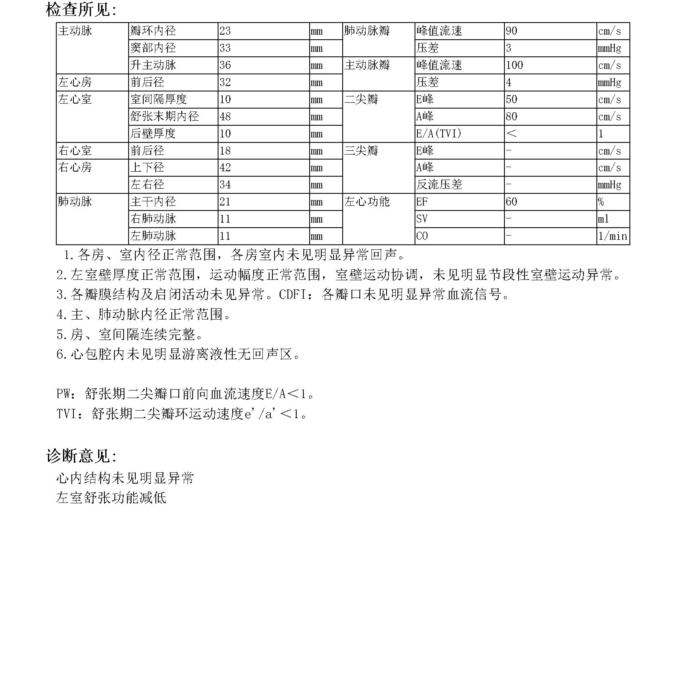

Supplement: Supplementary file 3 — Supplementary Data 2 [file 41746_2026_2648_MOESM3_ESM.zip › echocardiography_reports/208.png]

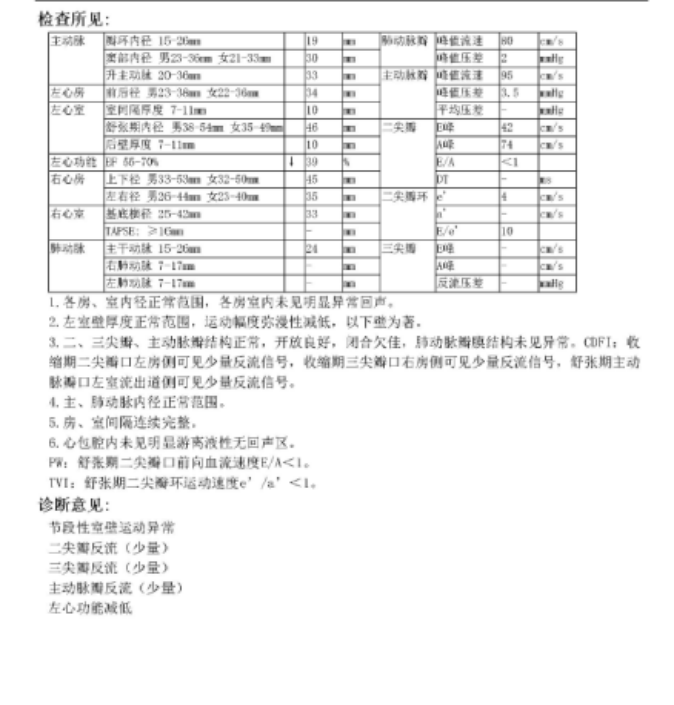

Supplement: Supplementary file 3 — Supplementary Data 2 [file 41746_2026_2648_MOESM3_ESM.zip › echocardiography_reports/209.png]

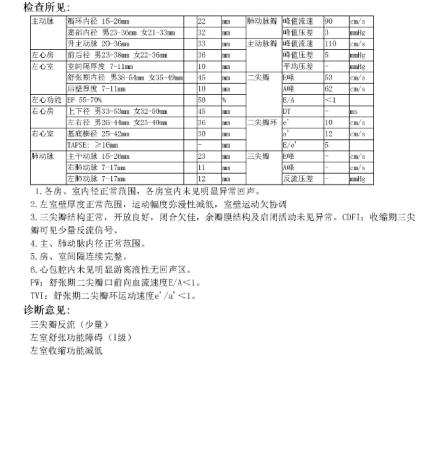

Supplement: Supplementary file 3 — Supplementary Data 2 [file 41746_2026_2648_MOESM3_ESM.zip › echocardiography_reports/210.png]

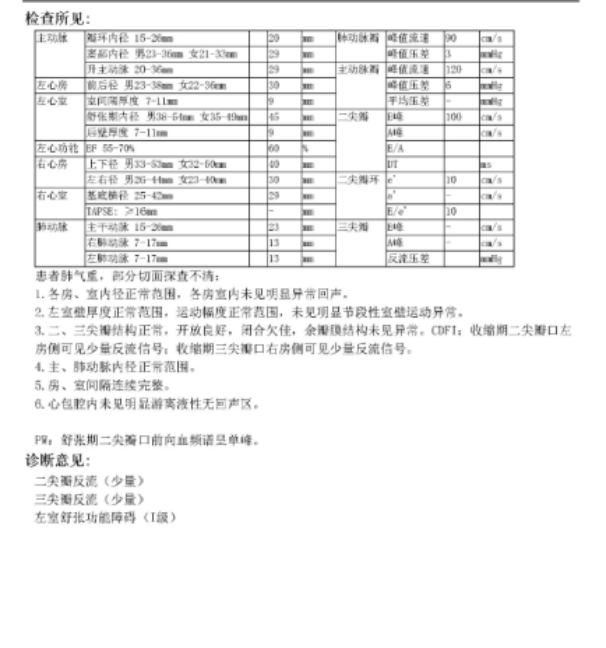

Supplement: Supplementary file 3 — Supplementary Data 2 [file 41746_2026_2648_MOESM3_ESM.zip › echocardiography_reports/211.png]

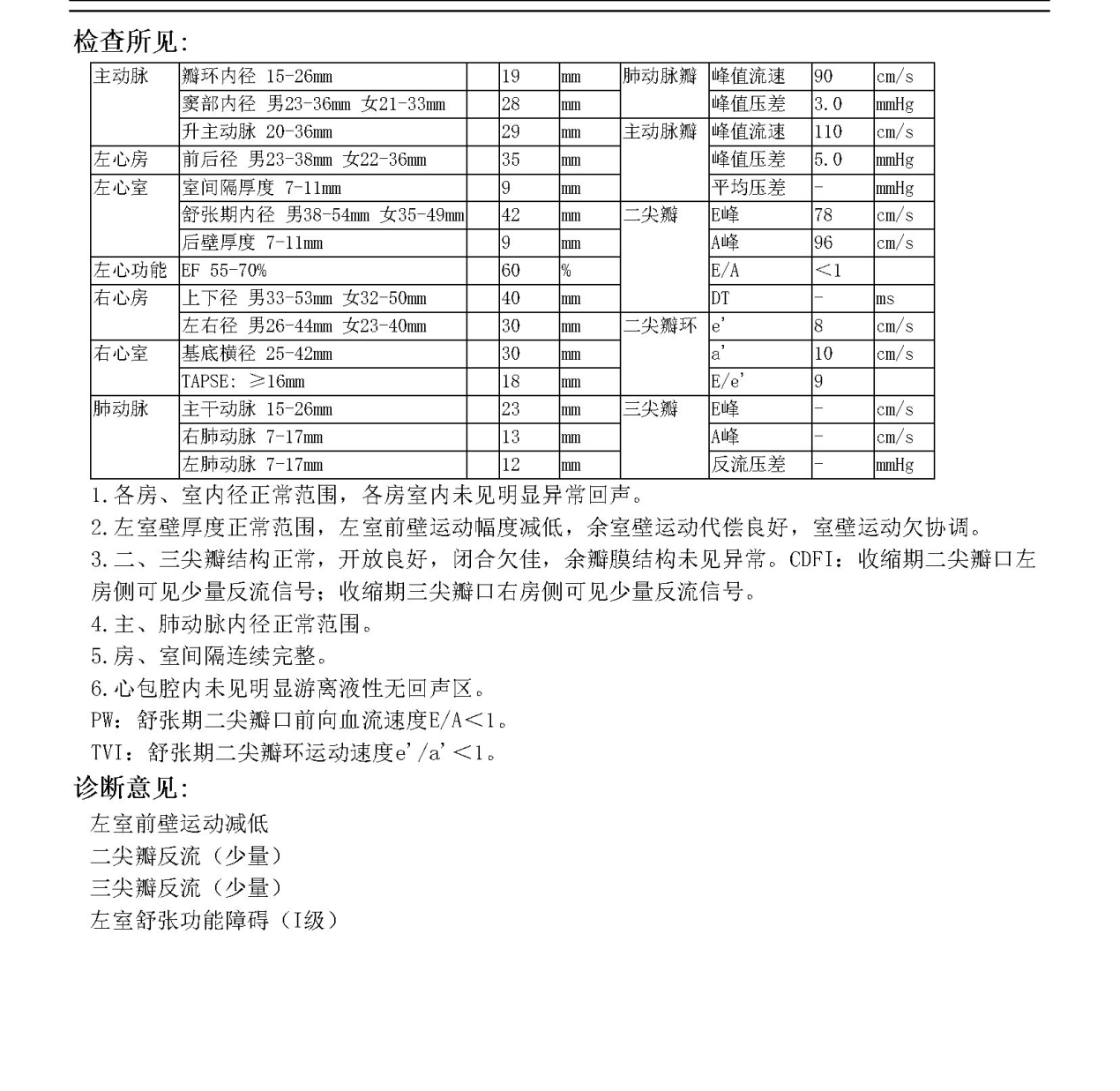

Supplement: Supplementary file 3 — Supplementary Data 2 [file 41746_2026_2648_MOESM3_ESM.zip › echocardiography_reports/212.png]

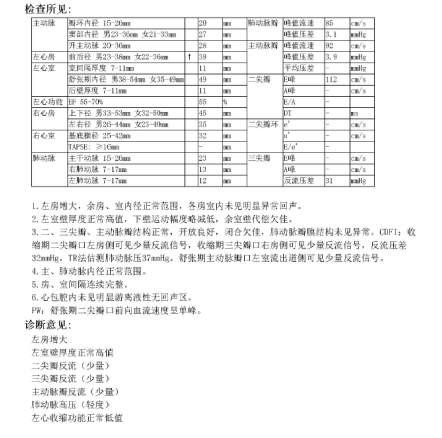

Supplement: Supplementary file 3 — Supplementary Data 2 [file 41746_2026_2648_MOESM3_ESM.zip › echocardiography_reports/213.png]

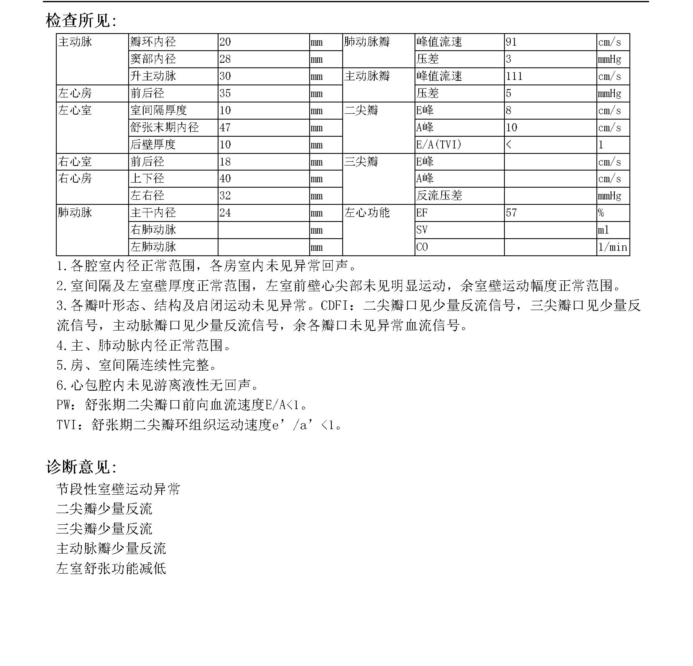

Supplement: Supplementary file 3 — Supplementary Data 2 [file 41746_2026_2648_MOESM3_ESM.zip › echocardiography_reports/214.png]

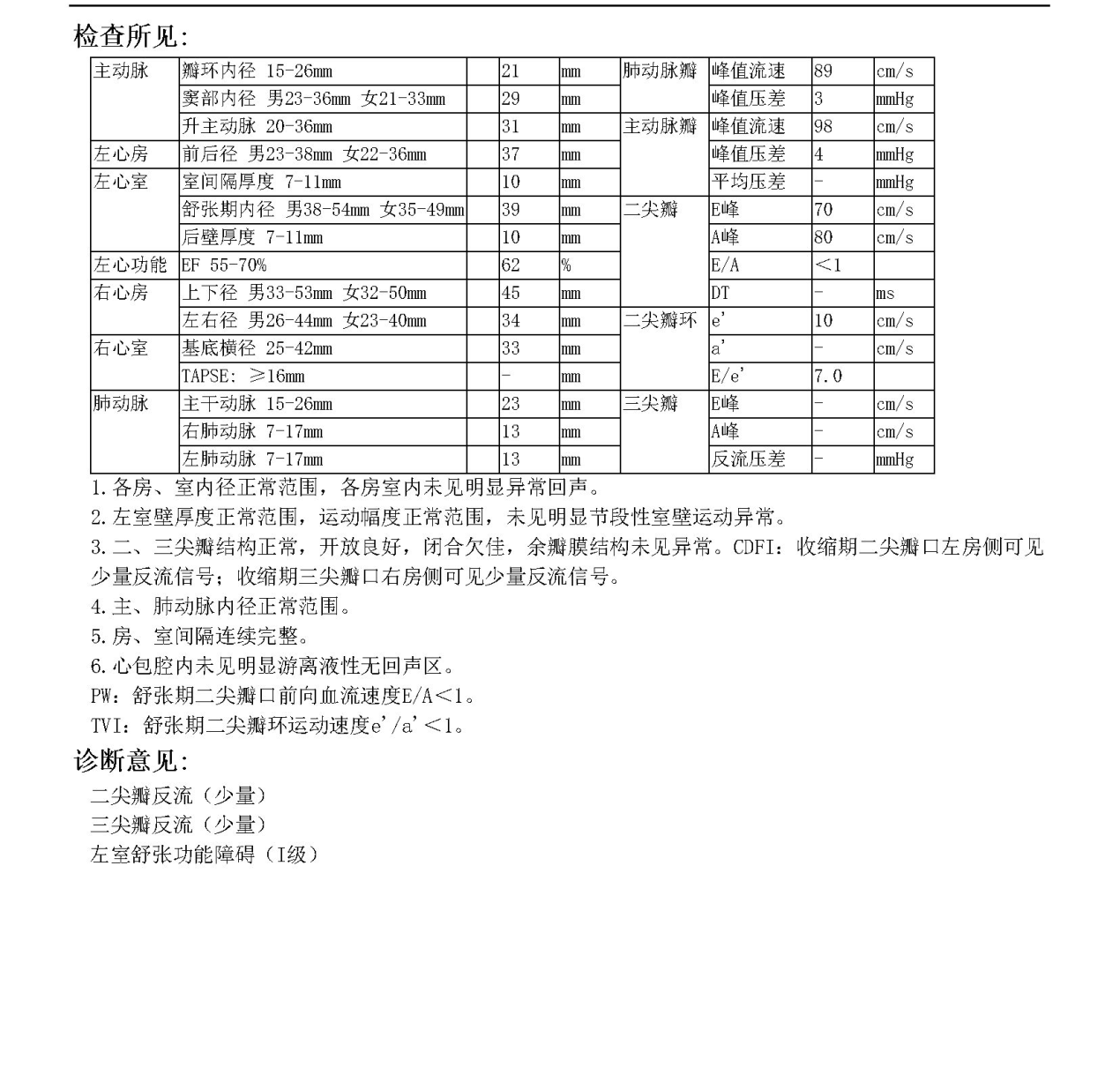

Supplement: Supplementary file 3 — Supplementary Data 2 [file 41746_2026_2648_MOESM3_ESM.zip › echocardiography_reports/215.png]

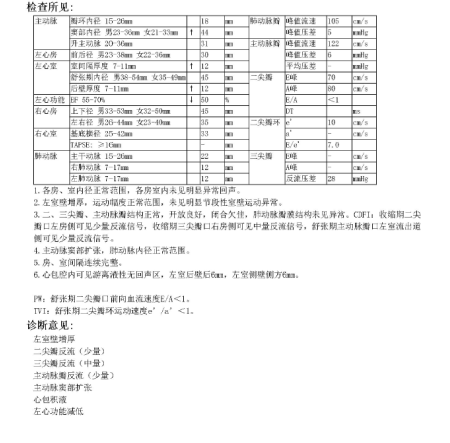

Supplement: Supplementary file 3 — Supplementary Data 2 [file 41746_2026_2648_MOESM3_ESM.zip › echocardiography_reports/216.png]

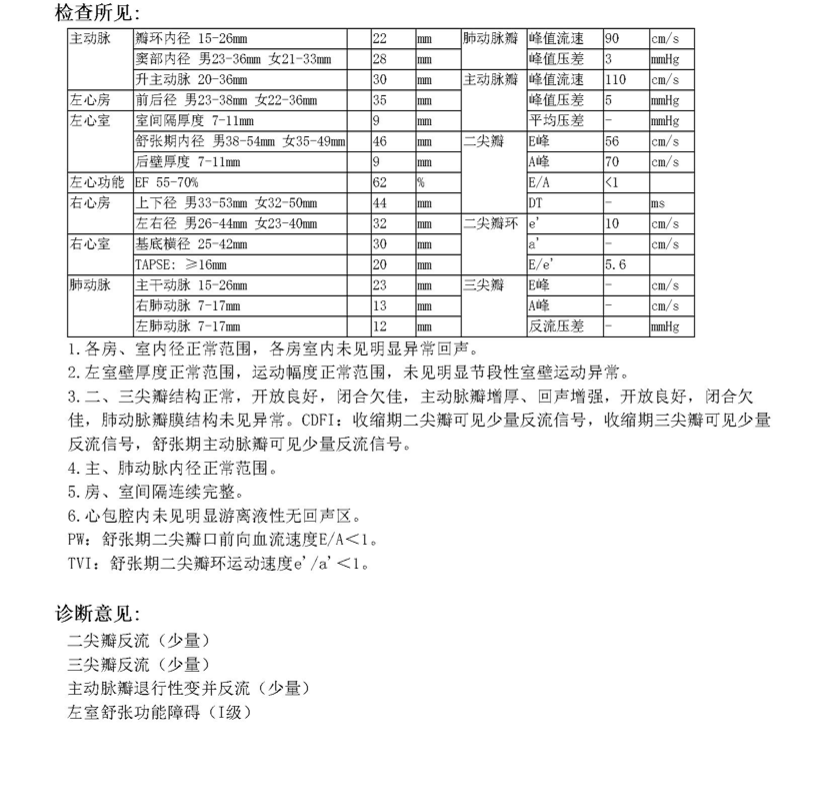

Supplement: Supplementary file 3 — Supplementary Data 2 [file 41746_2026_2648_MOESM3_ESM.zip › echocardiography_reports/217.png]

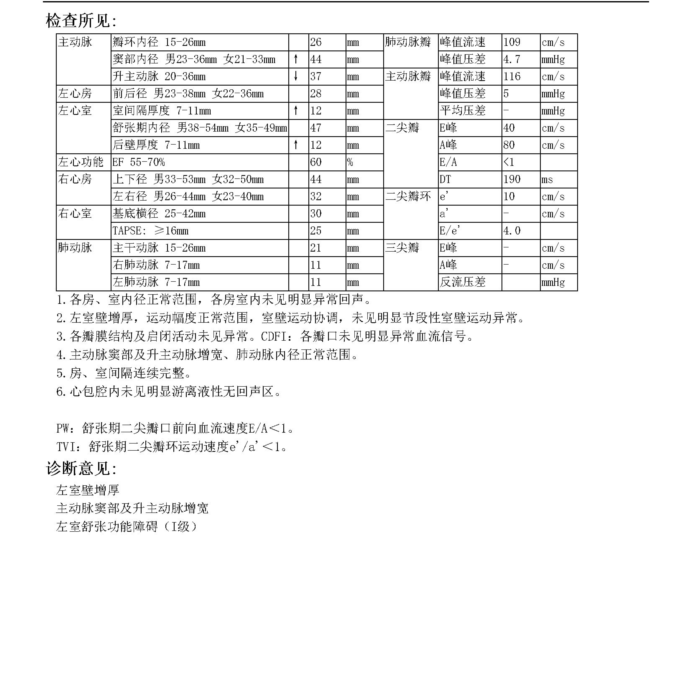

Supplement: Supplementary file 3 — Supplementary Data 2 [file 41746_2026_2648_MOESM3_ESM.zip › echocardiography_reports/218.png]

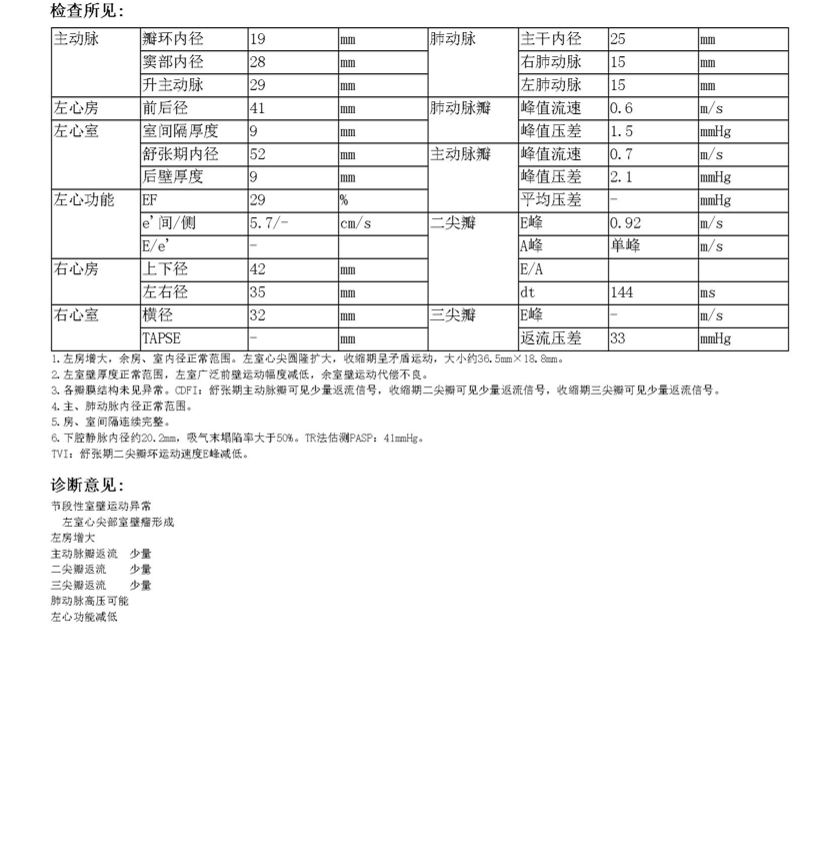

Supplement: Supplementary file 3 — Supplementary Data 2 [file 41746_2026_2648_MOESM3_ESM.zip › echocardiography_reports/219.png]
